# Regulation of the cardiomyocyte transcriptome *vs *translatome by endothelin-1 and insulin: translational regulation of 5' terminal oligopyrimidine tract (TOP) mRNAs by insulin

**DOI:** 10.1186/1471-2164-11-343

**Published:** 2010-05-29

**Authors:** Thomais Markou, Andrew K Marshall, Timothy E Cullingford, El L Tham, Peter H Sugden, Angela Clerk

**Affiliations:** 1 National Heart and Lung Institute (Cardiovascular Sciences), Faculty of Medicine, Imperial College London, London, UK

## Abstract

**Background:**

Changes in cellular phenotype result from underlying changes in mRNA transcription and translation. Endothelin-1 stimulates cardiomyocyte hypertrophy with associated changes in mRNA/protein expression and an increase in the rate of protein synthesis. Insulin also increases the rate of translation but does not promote overt cardiomyocyte hypertrophy. One mechanism of translational regulation is through 5' terminal oligopyrimidine tracts (TOPs) that, in response to growth stimuli, promote mRNA recruitment to polysomes for increased translation. TOP mRNAs include those encoding ribosomal proteins, but the full panoply remains to be established. Here, we used microarrays to compare the effects of endothelin-1 and insulin on the global transcriptome of neonatal rat cardiomyocytes, and on mRNA recruitment to polysomes (i.e. the translatome).

**Results:**

Globally, endothelin-1 and insulin (1 h) promoted >1.5-fold significant (false discovery rate < 0.05) changes in expression of 341 and 38 RNAs, respectively. For these transcripts with this level of change there was little evidence of translational regulation. However, 1336 and 712 RNAs had >1.25-fold significant changes in expression in total and/or polysomal RNA induced by endothelin-1 or insulin, respectively, of which ~35% of endothelin-1-responsive and ~56% of insulin-responsive transcripts were translationally regulated. Of mRNAs for established proteins recruited to polysomes in response to insulin, 49 were known TOP mRNAs with a further 15 probable/possible TOP mRNAs, but 49 had no identifiable TOP sequences or other consistent features in the 5' untranslated region.

**Conclusions:**

Endothelin-1, rather than insulin, substantially affects global transcript expression to promote cardiomyocyte hypertrophy. Effects on RNA recruitment to polysomes are subtle, with differential effects of endothelin-1 and insulin on specific transcripts. Furthermore, although insulin promotes recruitment of TOP mRNAs to cardiomyocyte polysomes, not all recruited mRNAs are TOP mRNAs.

## Background

Changes in the phenotype of cells (e.g. proliferation, differentiation, hypertrophic growth) result from changes in gene expression. Emphasis is often placed on RNA expression, and the availability of microarray technology has enabled studies of the global transcriptome. However, gene expression is also influenced by the rate of translation into protein. The global rate of protein synthesis relates to the capacity for and efficiency of translation [1,2]. Capacity is increased by synthesis of ribosomal subunits and other translational components, whereas efficiency is regulated by the rate of translational initiation (assembly of initiation factors, "unwinding" of RNA secondary structures, scanning and recognition of the initiation codon), and the rate of peptide chain elongation. Individual mRNAs are subject to additional levels of translational regulation, and elements in their 5' and 3' untranslated regions (UTRs) may interact with regulatory RNAs (e.g. antisense sequences, microRNAs) or RNA binding proteins to modulate ribosomal association [2]. The 5' UTR particularly influences the rate of initiation via 5' terminal oligopyrimidine tracts (TOPs), inclusion of short upstream open reading frames (uORFs), GC content and UTR length [2,3].

TOP mRNAs possess 5-15 pyrimidines at the 5' end, usually starting with C [4]. They are subject to growth-associated translational regulation, and stimulation with serum increases their polysomal association. mRNAs encoding ribosomal proteins, elongation factors and some subunits of Eif3e initiation factor are all TOP mRNAs [4,5]. Recruitment to polysomes increases their rate of translation, thus increasing translational capacity. Several studies have used microarrays to analyse RNA recruitment to polysomes [6-10], and bioinformatics approaches have been used to identify potential TOP mRNAs [11]. However, the full panoply of TOP mRNAs is not known and the extent to which translational regulation is mediated through TOP mRNAs relative to other mechanisms (e.g. uORFs) remains to be established. Phosphoinositide 3' kinase (PI3K), signaling through protein kinase B (PKB, also known as Akt) and mammalian target of rapamycin (mTOR), is particularly implicated in translational regulation [1,12]. mTOR complex 1 (mTORC1) promotes phosphorylation (activation) of p70 S6 kinases (p70S6Ks) that phosphorylate the small ribosomal subunit protein S6 (Rps6), and this was proposed to promote translation of TOP mRNAs. However, protein synthesis and recruitment of TOP mRNAs to polysomes in the presence of serum is not inhibited in p70S6K-null cells [13], and alternative mechanisms and signaling pathways may operate. For example, p90 ribosomal S6 kinases (p90RSKs), activated by extracellular signal-regulated kinases 1/2 (ERK1/2), also phosphorylate Eif4b and Eef2k [1]. Additionally, the pathways are integrated and ERK1/2 can activate mTORC1 independently of PI3K [1,12]. In a global context, PI3K signaling also influences the global rate of translation by promoting phosphorylation of 4E-BP1 [1,12]. This promotes dissociation of 4E-BP1 from Eif4e, allowing Eif4e to bind to the 7-methylGTP cap of mRNAs and increase the rate of initiation.

Cardiomyocytes, the contractile cells of the heart, withdraw from the cell cycle perinatally. Maturational growth of the heart results from an increase in cell size, but cardiomyocytes also hypertrophy in response to physiological stresses (e.g. hypertension) [14]. Cardiomyocyte hypertrophy is manifested in increased cell size and sarcomeric content. This reflects underlying changes in gene/protein expression, resulting from alterations in the transcriptome coupled with an increase in the rate of protein synthesis. Some would argue that the increased rate of protein synthesis is a crucial factor in facilitating hypertrophy [15]. Various neurohumoral factors promote cardiomyocyte hypertrophy including endothelin-1 (ET-1) and other agonists that potently activate ERK1/2, and ERK1/2 signaling is particularly implicated in promoting hypertrophy [16]. Insulin is associated with cardiomyocyte growth since it increases the rate of cardiac protein synthesis [17] and, as in other cells, insulin potently activates PKB/Akt via PI3K in cardiomyocytes [18]. Insulin activates ERK1/2 to a degree, but this is substantially less than that induced by ET-1 and, although ET-1 activates PKB/Akt to a minor degree, this is substantially less than insulin [19]. Notably, insulin does not induce the same hypertrophic phenotype as, for example, ET-1, and inhibition of ERK1/2 signaling, but not PI3K, attenuates cardiomyocyte hypertrophy induced by hypertrophic agonists [19]. In hearts *in vivo*, pressure overload increases recruitment of Rpl32 mRNA (an example of a TOP mRNA) rather than non-TOP mRNAs (β-myosin heavy chain) suggesting that the TOP mRNA mechanism is an integral part of cardiac hypertrophy [20]. Similar effects are seen in feline cardiomyocytes with ET-1 and insulin, both of which activate mTOR. However, only a single TOP mRNA was examined and whether this extends to other established TOP mRNAs, or if there are additional TOP mRNAs in the cardiac transcriptome is unknown.

Previously, we reported the acute effects of ET-1 on the cardiomyocyte transcriptome [21], identifying 1306 RNAs as temporally-regulated over the first 4 h of stimulation. Most of the protein-coding RNAs are approximately equally changed in total and polysomal RNA pools, suggesting that there is little translational regulation of these transcripts. Here, we compare the changes induced by ET-1 and insulin in the global cardiomyocyte transcriptome and in transcript recruitment to polysomes. Unlike ET-1, insulin did not have a substantial effect on global transcript expression, but both agonists differentially affected RNA recruitment to polysomes. Furthermore, whilst insulin did promote recruitment of TOP RNAs to the polysomes, not all recruited mRNAs possessed a TOP sequence.

## Results

### Signaling through to protein synthesis by ET-1 and insulin

**Figure 1:**
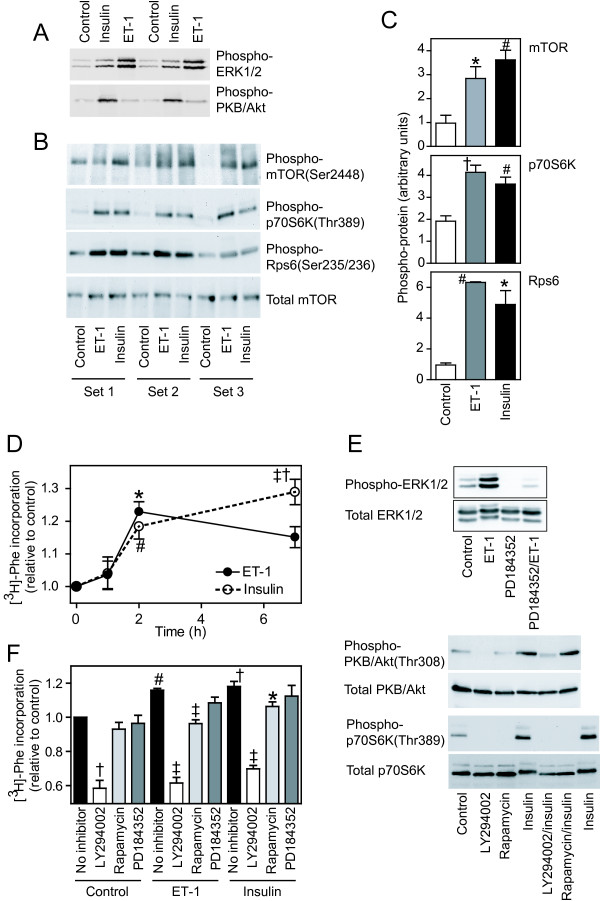
**Protein kinase signaling and regulation of protein synthesis by ET-1 and insulin**. Cardiomyocytes were exposed to 100 nM ET-1 or 50 mU/ml insulin or left unstimulated. A-C, Cardiomyocytes were exposed to agonists for 5 min (A) or 15 min (B and C). Protein extracts were immunoblotted with antibodies to phosphorylated ERK1/2 (A, upper panel), phosphorylated PKB/Akt (A, lower panel), phosphorylated mTOR (B, top panel), phosphorylated p70S6k (B, upper center panel), phosphorylated Rps6 (B, lower center panel) or total mTOR (B, lower panel). Each set of samples was prepared from a separate preparation of cardiomyocytes. C, Bands from images in panel B were quantified by scanning densitometry. Results are means ± SEM (n = 3). * p < 0.05, # p < 0.01, † p < 0.001 relative to controls (one-way ANOVA with Tukey post-test). D, Cardiomyocytes were exposed to agonists for the total time indicated and rate of protein synthesis was measured over the last 1 h of incubation using [^3^H]-Phe. Results are means ± SEM (n = 5 independent preparations of cardiomyocytes). * p < 0.05, # p < 0.01, † p < 0.001 relative to controls, ‡ p < 0.05 relative to 2 h (one-way ANOVA with Tukey post-test). E, Cardiomyocytes were unstimulated (Control) or exposed to agonists/inhibitors as follows: ET-1 (5 min), PD184352 (2 μM, 15 min), PD184352/ET-1 (10 min pretreatment with PD184352 before addition of ET-1 for 5 min), insulin (10 min), LY294002 (50 μM, 20 min), rapamycin (1 μM, 20 min), LY294002/insulin or rapamycin/insulin (10 min pretreatment with inhibitor before addition of insulin for 10 min). Extracts were immunoblotted for phosphorylated and total ERK1/2, PKB/Akt and p70S6K as indicated. Experiments were repeated with similar results. F, Cardiomyocytes were exposed for 2 h to 50 μM LY294005, 1 μM rapamycin, 2 μM PD184352, 100 nM ET-1 or 50 mU/ml insulin, or ET-1 or insulin in the presence of each inhibitor. The rate of protein synthesis was measured over the last 1 h of incubation using [^3^H]-Phe. Results are means ± SEM (n = 5 independent preparations of cardiomyocytes). # p < 0.01, † p < 0.001 relative to controls, * p < 0.05, ‡ p < 0.001 relative to ET-1 or insulin alone (one-way ANOVA with Tukey post-test).

To compare the effects of ET-1 and insulin on activation of intracellular signaling pathways in cardiomyocytes, extracts were immunoblotted with antibodies selective for phosphorylated (activated) forms of ERK1/2, PKB/Akt, mTOR, p70S6K and Rps6. Consistent with previous studies [19], ET-1 (100 nM, 5 min) potently activated ERK1/2 with little effect on PKB/Akt whereas insulin (50 mU/ml, 5 min) had little effect on ERK1/2 phosphorylation, but potently activated PKB/Akt (Figure 1A). Both agonists promoted phosphorylation of mTOR(Ser2448), p70S6K(Thr389) and Rps6(Ser235/Ser236) (Figure 1, B and 1C). Although insulin consistently stimulated a greater increase in phospho-mTOR(Ser2448) than ET-1, this was not reflected in the degree of phosphorylation of p70S6K(Thr389) and Rps6(Ser235/236). Our previous studies demonstrated that 4E-BP1 exhibits significant phosphorylation in unstimulated cardiomyocytes, and the degree of phosphorylation is increased in response to insulin [18]. Furthermore, insulin increases the association of Eif4e with 7-methylGTP-sepharose and would thus be predicted to increase the rate of initiation of translation for the 7-methylGTP cap structure.

Insulin or ET-1 increases the rate of protein synthesis over the first 4 h of stimulation [18]. By pulse labelling with [^3^H]-Phe for the final 1 h for periods up to 7 h, we established that there was little increase in the rate of protein synthesis over the first hour, but the rate increased significantly over the second hour with either insulin or ET-1 and this was sustained for up to 7 h (Figure 1D). Inhibiting ERK1/2 signaling with PD184352 (2 μM, a concentration that gives specific inhibition of this pathway [22]; Figure 1E) had no significant effect on baseline protein synthesis or the increase in the rate of protein synthesis induced by ET-1 or insulin between 1 and 2 h (Figure 1F). Inhibiting PI3K signaling with 50 μM LY294002 [23] (Figure 1E) inhibited baseline protein synthesis to ~60% of control levels and inhibited the increase in protein synthesis induced by ET-1 or insulin (Figure 1F). Consistent with our previous study over 4 h [18], this suggests that PI3K plays a significant role in cardiomyocyte protein synthesis. Rapamycin inhibited the phosphorylation of p70S6K (Figure 1E), but had no significant effect on baseline protein synthesis. However, it did inhibit the increase induced by ET-1 and attenuated the increase induced by insulin (Figure 1F).

### Regulation of the global transcriptome by ET-1 or insulin

**Figure 2:**
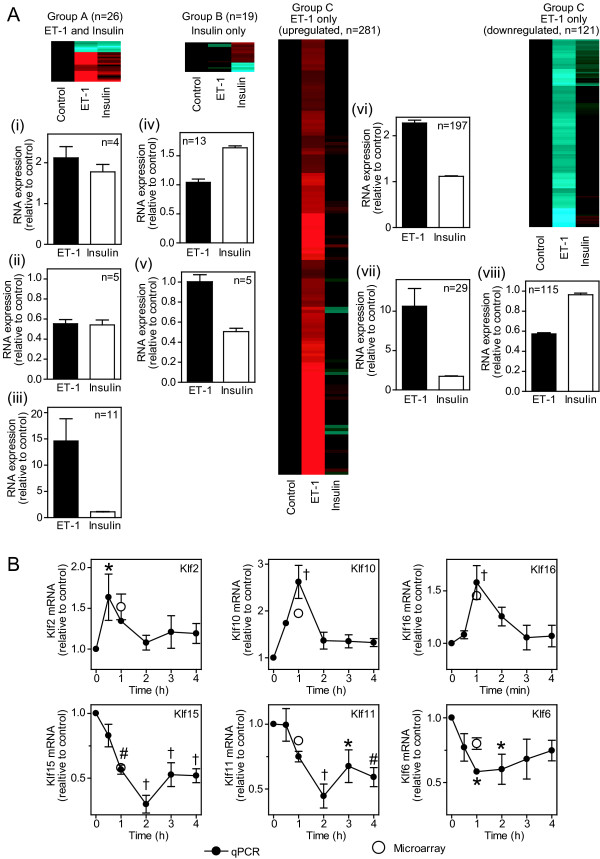
**Effects of ET-1 and insulin on the global cardiomyocyte transcriptome**. A, Cardiomyocytes were exposed to 100 nM ET-1 or 50 mU/ml insulin (1 h) or left unstimulated (Control). Total RNA was extracted and the global transcriptome analysed using microarrays. Transcripts with significant changes in expression (FDR < 0.05, >1.5-fold change) induced by either ET-1 or insulin relative to controls were clustered according to responsiveness to both agonists [Group A: (i) similar upregulation with both, (ii) similar downregulation with both, (iii) greater response to ET-1 than insulin], responsiveness to insulin alone [Group B: (iv) upregulated by insulin, (v) downregulated by insulin] or responsiveness to ET-1 alone [Group C: (vi) and (vii) upregulated by ET-1, (viii) downregulated by ET-1. Heatmaps represent the mean values of all probesets in each group with normalisation per gene to control values [Log_2 _scale; -2.0 (cyan) through 0 (black) to 2.0 (red)]. Histograms are means ± SEM for the RNAs in each group (numbers of transcripts in parentheses). B, Cardiomyocytes were exposed to insulin for the times indicated and mRNA expression of Klf2, Klf6, Klf10, Klf11, Klf15 and Klf16 measured by qPCR. Data were normalised to Gapdh. Solid circles represent qPCR data. Results are means ± SEM (n = 3 independent preparations of cardiomyocytes). * p < 0.05, # p < 0.01, † p < 0.001 relative to controls (one-way ANOVA with Tukey post-test). For comparison, microarray data are shown as open circles (means ± SEM, n = 4).

Previous studies demonstrated that almost all the changes induced by ET-1 in the cardiomyocyte transcriptome at 1 h (470 RNAs, n = 8) represent immediate early genes [21]. Here, we compared the effects of ET-1 (100 nM) with insulin (50 mU/ml) on the global transcriptome at 1 h. Using the same criteria (FDR < 0.05, >1.5-fold change), expression of 428 and 45 probesets was significantly changed by ET-1 or insulin, respectively (Figure 2A; Additional file 1). Of RNAs responsive to both agonists (20 RNAs, 26 probesets), 4 were similarly upregulated and 5 similarly downregulated (Group A, clusters (i) and (ii)), whereas 11 were upregulated to a greater extent by ET-1 (Group A, cluster (iii)). Only 18 RNAs (19 probesets) were selectively regulated by insulin (13 upregulated and 5 downregulated; Group B, clusters (iv) and (v)), whereas 341 RNAs (402 probesets) were selectively regulated by ET-1 [226 upregulated and 115 downregulated; Group C, clusters (vi), (vii) and (viii)]. We previously focused on Krüppel-like factors (Klfs) to validate ET-1-responsive changes [21,24]. We therefore validated the insulin microarray data for Klfs by qPCR (Figure 2B). Consistent with the microarray data, insulin promoted transient increases in expression of Klf2, Klf10 and Klf16 mRNAs (maximal expression at 0.5 - 1 h), with downregulation of Klf15, Klf11 and Klf6 mRNAs (maximal downregulation by 2 h). These data indicate that, although insulin does affect the global transcriptome, the response to ET-1 is substantially greater with respect to relative levels and numbers of changes.

### RNA recruitment to cardiomyocyte polysomes

**Figure 3:**
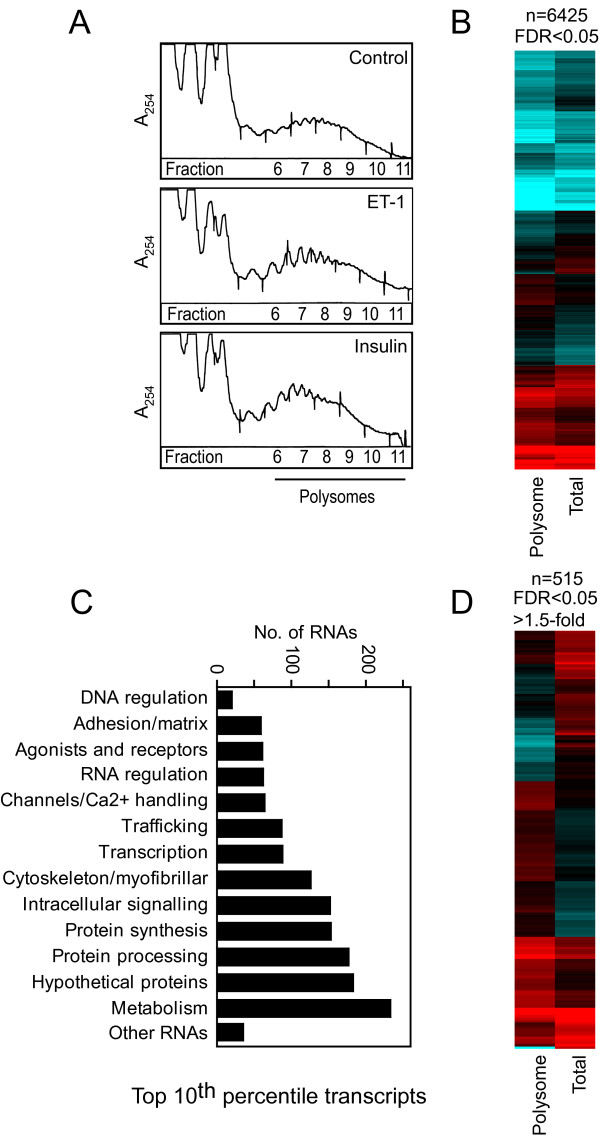
**Differential recruitment of cardiomyocyte RNAs to polysomes**.Cardiomyocytes were serum-starved (20 h). Polysomes prepared by sucrose density centrifugation. Total and polysomal RNA were extracted and analysed using microarrays. A, A_254 _profiles for sucrose density centrifugation. B, Heatmap of the mean raw fluorescence values for the 6425 probesets with significantly different expression in polysomal and total RNA pools in unstimulated cells [Log_2 _scale; 7 (cyan) through 10 (black) to 13 (red)]. C, Functional classification of top 10^th ^percentile transcripts detected in cardiomyocytes. D, Heatmap for the 515 probesets of the top 10^th ^percentile with significantly different expression (FDR < 0.05, >1.5-fold difference) in polysomal and total RNA pools in unstimulated cells [Log_2 _scale; 9.5 (cyan) through 11.5 (black) to 13.5 (red)].

To study translational regulation, cardiomyocyte polysomes were prepared by sucrose density centrifugation (Figure 3A) and RNA extracted. Equal amounts of labelled cRNA from total or polysomal pools were analysed using microarrays. Initially, we studied the baseline differences between the total and polysomal RNA profiles. Polysomal RNA profiles of unstimulated cells (serum-starved for 24 h) were notably different from total RNA profiles and, using raw fluorescence values, expression of 6425 of the 15,446 probesets detected was significantly different between the RNA pools (FDR < 0.05; Figure 3B). Given the range of non-protein coding RNAs that exist, this may not necessarily be considered as surprising. However, of the top 10^th ^percentile of detected probesets (1655 probesets) that predominantly encode mRNAs (Figure 3C, Additional file 2), 715 probesets (~43%) were similarly expressed in total and polysomal RNA pools (FDR > 0.05 and/or <1.25-fold difference), but 515 protein-coding mRNAs (~31%) exhibited >1.5-fold difference (FDR < 0.05) in expression (Figure 3D). Differential expression did not correlate with raw fluorescence values (Additional file 2), so the differences do not simply reflect excessive levels of expression or saturation of the microarray system. The data suggest that a significant proportion of constitutively expressed mRNAs in cardiomyocytes are translationally regulated and, in serum-starved cells, there appears to be a reservoir in the total RNA pool available for polysomal recruitment.

To identify RNAs with the greatest degree of differential expression between total and polysomal RNA pools, the data were normalised to the gene median. Of RNAs with substantial and significant differential expression (FDR < 0.05, >3-fold difference; Additional file 3), 157 were non-protein coding RNAs for sequences in introns, potential antisense sequences or probable microRNA precursors (given the proximity to established microRNAs on the mouse genome) with a further 52 probesets for RNAs associated with no established gene. As might be expected, virtually all (there are 2 exceptions) were preferentially detected in the total RNA pool. Of the 221 probesets encoding mRNAs, only 12 were preferentially recruited to the polysomes. Interestingly, the most disproportionately expressed mRNAs encoded α-myosin heavy chain or β-myosin heavy chain, key myofibrillar proteins, both of which were preferentially detected in total RNA. These data further suggest that cardiomyocytes possess a reservoir of mRNAs.

### Translational regulation in cardiomyocytes by insulin or ET-1

Consistent with the effects on protein synthesis (Figure 1D), insulin or ET-1 (1 h) increased cardiomyocyte polysome content, as seen by the increase in A_254 _in heavier sedimenting fractions (6 -11) and decrease in A_254 _in fractions 1-4 (Figure 3A). Polysomal and total RNA profiles were generated by hybridisation of equal amounts of labelled cRNA to microarrays. To identify RNAs subject to translational regulation by insulin or ET-1, the data for each RNA pool were normalised to unstimulated controls and the relative change in each pool (total and polysomal) was assessed independently. We subsequently compared the degree of change in total vs polysomal RNA. Thus, probesets were selected with >1.25-fold change in total or polysomal RNA relative to controls, and filtered on statistical significance (FDR < 0.05). Potential translationally-regulated RNAs were then selected with statistically-significant changes only in polysomal RNA (Group I RNAs) or only in total RNA (Group II RNAs) and with expression ratio (fold change in polysomal RNA:fold change in total RNA or vice versa) >1.2. We considered there was insufficient evidence for translational regulation of remaining RNAs (Group III, FDR > 0.05 and/or expression ratio < 1.2).

**Figure 4:**
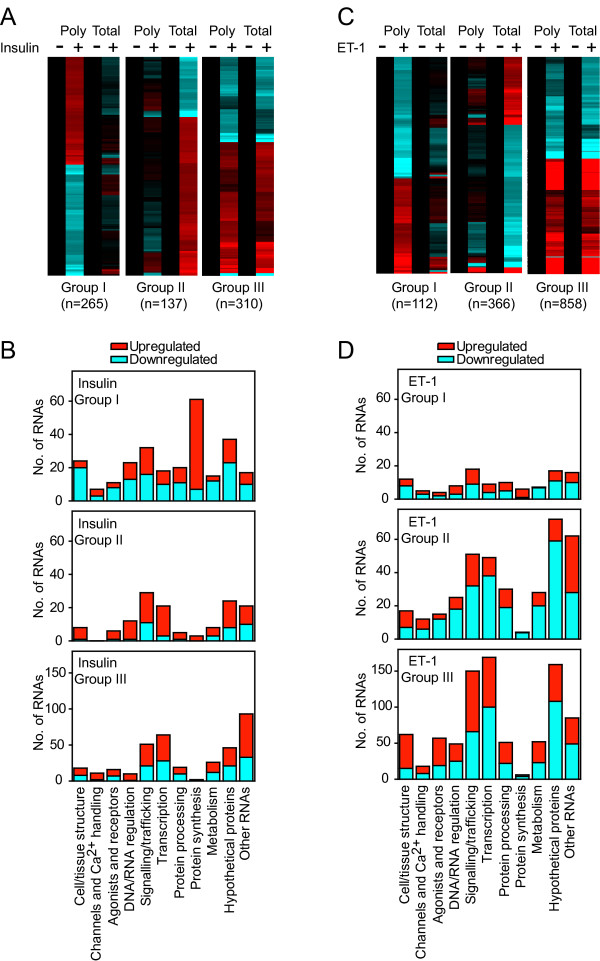
**Translational regulation of cardiomyocyte transcripts by insulin or ET-1**.Cardiomyocytes were exposed to 100 nM ET-1 or 50 mU/ml insulin (1 h) or were left unstimulated. Polysomes were prepared by sucrose density centrifugation. Total and polysomal RNA were extracted and analysed using microarrays. RNAs with significant changes in expression (FDR < 0.05, >1.25-fold change) induced by insulin (A and B) or ET-1 (C and D) in either total or polysomal RNA pools were identified and clustered according to regulation in polysomal RNA only (Group I; FDR < 0.05 in polysomal RNA only and expression ratio >1.2 for polysomal RNA:total RNA), regulation in total RNA only (Group II; FDR < 0.05 in total RNA only and expression ratio >1.2 for total RNA:polysomal RNA) or similar regulation in both (Group III; FDR < 0.05 in both polysomal and total RNA, or expression ratio <1.2). A, Heatmap for insulin-regulated probesets showing the mean values of all probesets in each group with normalisation per gene to control values [Log_2 _scale; -0.7 (cyan) through 0 (black) to 0.7 (red)]. C, Heatmap for ET-1-regulated probesets showing the mean values of all probesets in each group with normalisation per gene to control values [Log_2 _scale; -1.5 (cyan) through 0 (black) to 1.5 (red)]. B and D, Functional classification of the RNAs illustrated in panels A and C, respectively, showing the numbers of RNAs in each group (numbers of upregulated and downregulated RNAs are represented in red and cyan, respectively).

**Figure 5:**
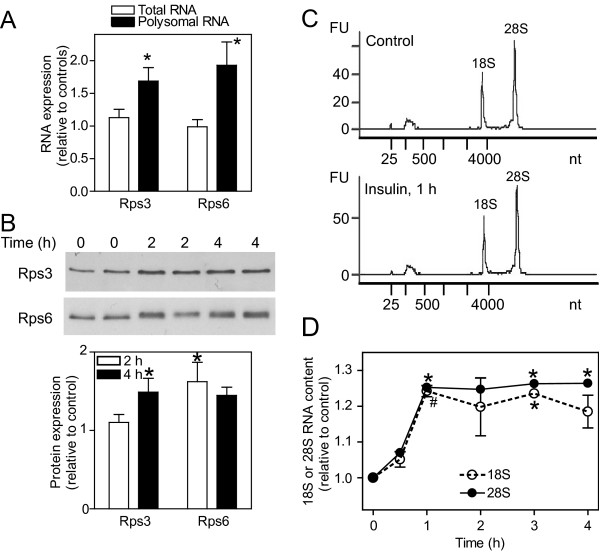
**Insulin increases ribosomal content in cardiomyocytes**. Cardiomyocytes (4 × 10^6 ^cells per sample) were exposed to 50 mU/ml insulin or were left unstimulated. A, RNA was extracted from cardiomyocytes exposed to insulin for 1 h. Rps3 and Rps6 mRNA expression was measured by qPCR using the absolute quantification protocol. Results are expressed relative to unstimulated controls and are means ± SEM (n = 8 independent preparations of cardiomyocytes). * p < 0.05 relative to controls (Student's t-test). B, Protein extracts from cardiomyocytes exposed to insulin for 0, 2 or 4 h were immunoblotted with antibodies to Rps3 (upper panel) or Rps6 (center panel). * p < 0.05 relative to controls (Student's t-test). C and D, RNA was extracted and analysed for 18S and 28S ribosomal RNA content using an Agilent Bioanalyser. Representative traces are shown in panel C for unstimulated (Control) cells and cells exposed to insulin for 1 h. The areas under the 18S and 28S peaks were integrated and the results are shown in panel D as means ± SEM (n = 3 independent preparations of cardiomyocytes). * p < 0.05, # p < 0.01 relative to controls (one-way ANOVA with Tukey post-test).

Approximately 56% of insulin-responsive and ~35% of ET-1-responsive transcripts were classed as Group I RNAs (265 RNAs with insulin; 112 RNAs with ET-1) or Group II RNAs (137 RNAs with insulin; 366 RNAs with ET-1) (Figure 4; Additional files 4 and 5). With respect to function, proteins associated with signaling/trafficking or transcriptional regulation, or RNAs for hypothetical proteins or transcripts of no known function were prevalent in all groups (Figure 4, B and 4D). However, insulin particularly increased polysomal association of mRNAs for proteins associated with protein synthesis (Figure 4, A and 4B; Group I RNAs), including TOP mRNAs for 47 cytosolic ribosomal proteins, Eif3f and Eef1b2 (Additional files 4 and 6). The TOP mRNA for Rpl39 was the only one to be decreased in cardiomyocyte polysomes in response to insulin. We validated the changes for two ribosomal protein mRNAs, Rps3 and Rps6 (Figure 5A) by qPCR, demonstrating selective upregulation in the polysomes in response to insulin. Consistent with this, absolute levels of Rps3 and Rps6 proteins (Figure 5B), and 18S and 28S RNAs (Figure 5C and 5D) were increased.

We identified all probesets for established TOP mRNAs encoding ribosomal proteins, elongation factors and Eif3e and Eif3h [5], and analysed the response to insulin. Of 79 ribosomal protein mRNAs, all but 10 were significantly increased in the insulin-responsive translatome and only 3 (Rpl36, Rpl39 and Rpl7l1) were not upregulated to any degree (Additional file 6). Eif3e and Eif3h mRNAs were also significantly upregulated in the insulin-responsive translatome and, of the elongation factors, Eef1b2 mRNA was the only elongation factor mRNA to be significantly upregulated (Additional file 6). Previous work suggests that ET-1 also increases translation of TOP mRNAs in feline cardiomyocytes as demonstrated by an increase in Rpl32 mRNA recruitment to polysomes [20]. Our data indicated that ET-1 did increase recruitment of mRNAs for Rpl32 and other ribosomal proteins to rat neonatal cardiomyocyte polysomes, but this was not as great as that induced by insulin, nor was it statistically significant (Additional file 6).

### Informatics analysis of 5' UTRs of mRNAs recruited to polysomes in response to insulin

113 of 132 RNAs significantly upregulated by insulin in the translatome but not the transcriptome encoded established proteins, 49 of which were established TOP mRNAs (see above). This raises the question of how many other mRNAs in this group also contain a TOP sequence. Since few transcriptional start sites (TSSs) have been mapped for the rat genome, we identified 5' UTR sequences for mRNAs for the mouse orthologs using the FANTOM3 CAGE (cap analysis of gene expression) database with support from NCBI databases and the Database of TSSs (DBTSS). We then identified 5' sequences of rat mRNAs (often predicted) from the NCBI rat genome database to confirm sequence conservation between species. With this approach we identified 5' UTR sequences for all but 6 mRNAs (Hnrph3, N6amt2, Ptrh1, Tac2, Thap6, Tmed5) in the insulin-responsive cardiomyocyte translatome. All ribosomal protein mRNAs identified were TOP mRNAs (Additional file 6), with a single exception, Rpl7l1. Interestingly, even though Rpl39 mRNA was downregulated in the translatome the 5'UTR contained a TOP sequence. Of other established mRNAs recruited to cardiomyocyte polysomes in response to insulin, Eif3e, Eif3f, Eif3h, Eef1b2, Gnb2l1 (also known as RACK1) and Npm1 were verified as TOP mRNAs (Additional file 6).

We examined 5' UTRs for other mRNAs selectively recruited to cardiomyocyte polysomes by insulin. Atg12, Atp5g2, Ccnj, Cnih, Gltscr2, Naca, Pfdn5 and Rbms2 were identified as probable TOP mRNAs on the basis of the TSS for mouse transcripts and homology with the rat genome (Table 1). Brpf1, Ilk, Plekhh3, Snrpd2 and Zfp110 mRNAs were identified as potential TOP RNAs although mouse and rat 5' UTRs have less identity and not all start with C (Additional file 6). We could not identify TOP sequences for 49 mRNAs and, although most contain polypyrimidine tracts, uORFs, GC tracts or untranslated 5' exons within the 5' UTR, there were no consistent features (Additional file 7). A recent bioinformatics study identified an additional 167 potential TOP mRNAs [11]. We mined our data for these, but only Impdh2 and Eif3e were significantly changed with another 28 being increased >10% albeit in the absence of statistical significance (Additional file 8). The majority showed no change in expression in cardiomyocyte polysomes in response to insulin although, given the recruitment of ribosomal protein mRNAs to cardiomyocyte polysomes, we are confident that a signaling pathway(s) for regulation of TOP mRNAs was operative.

**Table 1:** Identification of novel TOP mRNAs.

Gene Symbol	Polysome RNA	Total RNA	5' UTR
Atg12	1.28	1.03	Mouse:***CTTCC***GCCGCCGCCTCTCAGCAAGCAAAGATG Rat: ***CTTCC***GCCGTCGACGCTCAGCAAGCAAGATG

Atp5g2	1.26	1.00	Mouse:***CCCTCTCT***GTCTTCTCTGCCCTGGGAGCAGCCCTCCTGCCTCGGCCCCTCACCCCTGAAAATG Rat: ***CCCTCTCT***GTCTTCTCTACCCTGGGAGCAGCTCTTCTGCCTTTGCCCCTCACCCCTGAAAAATG

Ccnj	1.52	1.20	Mouse:***CTTCC***AGACTGAAGTTGCGGCGTCTGGCCGGGCGCACCTCCGGCTTCCATG Rat: ***CTTCC***AGACTGAAGTTGCGGCGTCTGGCTGGGCGCGCCTCCGGCTTCCATG

Cnih	1.25	1.02	Mouse:***CTTTCTCC***GCTGGCCCCGGCGCGCCCGGCAGCTCCTCCCCGGCCATG Rat: ***CTTTCTCC***GCTGGCCCCGGCGCGCCCGGCAGCTCCTCCCCGGCCATG

Gltscr2	1.28	0.91	Mouse:***CTTCCTTT***AAGAAGATG Rat: ***CTTCCTTT***AAGAAGATG

Naca	1.25	0.96	Mouse:***CTCTTTCT***GCCGCCATCTTGGTTCCGTGATCTCCGCACAAAATG Rat: ***CTCTTTCC***ACCGCCATCTTGGTTCCGTGTTCTCCGCACAAAATG

Pfdn5	1.28	0.90	Mouse:***CTTCCTCTTC***GGCAGTCCTCCTTCCCAACATG Rat: ***CTTCC***GCTTCGGCATTCCTCCTTCCCAACATG

Rbms2	1.27	0.96	Mouse:***CTCCCTCCCTTTCCTTC***ACTCTCTTCTTTCTCTCTCTCAGCTCCGTAACAGTAAAGAAAAAATG Rat: ***CTCCCTCCCTTTCCTTC***ACTCTCTTCTTTCTCTCTCTCAGCTCCGTAACAGTAAAGAAAAAATG

Total and polysomal RNA from cardiomyocytes exposed to insulin (50 mU/ml, 1 h) or unstimulated cardiomyocytes (controls) were analysed using microarrays. Probesets corresponding to mRNAs with >1.25-fold increase in expression (FDR < 0.05) were identified. Results are the mean fold change in insulin stimulated samples relative to controls. Mouse and rat 5' UTR sequences were identified. The TOP sequence is in bold italics. Accession numbers are provided in Additional data file 6.

ET-1 promoted some increase in recruitment to cardiomyocyte polysomes of mRNAs encoding ribosomal proteins (Additional file 6), suggesting there may be an effect on TOP mRNAs. We therefore examined the 5' UTR sequences of mRNAs with significant and selective recruitment to cardiomyocyte polysomes in response to ET-1 (Additional file 5). Of the 30 mRNAs for which we could determine the potential TSS, 6 were also regulated by insulin (Afg3l2, Azi2, Ccnl2, Eef2k, Sfrs5, Slc25a33) but did not possess a TOP sequence, and there were no consistent features for the remaining 24 mRNAs (Additional file 9).

## Discussion

### Transcription *vs *translation in cardiomyocytes

Most transcriptomics studies focus on global changes in RNA expression in response to specific stimuli or in specific disease states. However, analysis of mRNAs associated with polysomes provides further insight into translational regulation. Studies in yeast [25,26], transformed cancerous cells [8-10] and primary T cells [6,7] all suggest that, in steady-state (usually starved) cells, there is disparity between global transcript expression and translation of specific mRNAs. Our data with unstimulated (serum-starved) cardiomyocytes (Figure 3, B - D) are consistent with this and, although many RNAs in the global transcriptome are non-protein coding and may not necessarily be expected to be recruited to polysomes, over 30% of the most abundant cardiomyocyte mRNAs were differentially expressed in global and polysomal pools (Figure 3, B - D). Many of the most disproportionately expressed mRNAs in the global RNA pool encoded cytoskeletal/myofibrillar proteins. In response to hypertrophic stimulation, these may be rapidly recruited to polysomes for translation, thus initiating hypertrophic growth in the absence of further increases in transcript levels.

A further question relates to the degree of translational regulation following exposure to growth stimuli. Since the relative effect on transcriptional *vs *translational regulation is likely to depend on the intracellular signaling pathways activated by specific stimuli, we compared ET-1 (potent activation of ERK1/2 with minimal activation of PI3K and PKB/Akt) and insulin (potent activation of PI3K and PKB/Akt with minimal activation of ERK1/2) [19]. Whereas ET-1 drives a substantial response in the global transcriptome (Figures 2 and 4), a large proportion of which requires ERK1/2 signaling [21], insulin had a relatively minor effect on global RNA expression, suggesting that PI3K signaling through PKB/Akt does not substantially affect transcription even though PKB/Akt phosphorylates, for example, Forkhead transcription factors that are then inactivated [17]. To gain insight into the insulin response and regulation of translation, it was necessary to examine smaller relative changes (>1.25-fold; Figure 4). This suggests that effects on translation of individual transcripts and of insulin generally are relatively subtle. Interestingly, insulin had a greater translational effect on recruiting transcripts to polysomes rather than a bias away from the polysomes (Figure 4, A and 4B, Group I transcripts), whereas ET-1 had a greater effect on downregulated transcripts that were preserved in the polysomal fraction (Figure 4, C and 4D, Group II transcripts). In interpreting the data from this study, it should be noted that (as is usual for microarray experiments) equal amounts of RNA from total and polysomal pools were hybridized to the microarrays. Given that the data normalization was to the values of unstimulated cells and that insulin and ET-1 each increased the absolute amount of polysomal RNA (Figure 3A), it may be viewed that a greater number of cells were represented from the unstimulated samples compared with those from samples stimulated with insulin or ET-1. As a consequence, the degree by which transcripts were increased in polysomal RNA by insulin or ET-1 and the number of transcripts decreased in polysomal RNA may have been underestimated. Our analysis is therefore likely to be conservative in relation to both these aspects. Nevertheless, since the criteria for analysis were identical for both agonists (using a relatively low level for relative fold change in combination with statistical evaluation) and both promoted a similar increase in cardiomyocyte polysomes, the data reliably highlight the differential effects of insulin and ET-1 on translational regulation in cardiomyocytes.

### Translational regulation by insulin and identification of TOP mRNAs

It is almost 20 years since the TOP motif was identified in mammalian mRNAs for ribosomal proteins [27], and this is probably the most well-established feature of mRNAs that are translationally regulated in response to nutrients or growth stimuli [28]. The best characterized TOP mRNAs encode ribosomal proteins and, although others have been identified, most are associated with the translational apparatus [5]. The prevalence of TOP mRNAs and the extent to which this mechanism accounts for translational regulation is still debated. Since PI3K signaling and mTOR are particularly implicated in the TOP mRNA response [12], we focused on the cardiomyocyte response to insulin. As expected, insulin increased recruitment of established TOP mRNAs to cardiomyocyte polysomes. Although we identified a few probable/possible TOP mRNAs, we could find no evidence for TOP sequences for ~43% of mRNAs we identified as significantly increased in the insulin-responsive cardiomyocyte translatome. There are problems in identifying TSSs and many genes are not well-studied, so we cannot be certain that the latter group are not subject to alternative splicing or possess alternative promoters that may produce a 5' TOP sequence. However, the data suggest that there may not be many more TOP mRNAs to be identified. The mRNAs with no apparent TOP sequence possess a variety of features in the 5' UTR that indicate potential for translational regulation. With no consistent features, further studies will need to focus on individual mRNAs, first to confirm the 5' UTR sequence and, assuming TOP sequences are not identified, to establish the mechanisms of regulation.

With the vast amount of genome sequence data available, it might be expected that identification of TOP mRNAs should be relatively trivial but, even for human and mouse genomes, the TSS is often not well-defined, partly because of alternative promoters and alternative splicing of non-protein-coding first exons. The bioinformatics approach of Yamashita et al. [11] utilised a database of TSSs to define all potential TOP mRNAs for the human genome. Surprisingly, we found very few of these mRNAs in our insulin-responsive pool of transcripts recruited to cardiomyocyte polysomes (Additional files 6 and 8). Yamashita et al. used a phorbol ester for experimental validation and demonstrated polysomal recruitment of 41 of 83 candidate TOP RNAs tested. Phorbol esters, like ET-1, potently activate ERK1/2 signaling in cardiomyocytes [16], but we detected no preferential recruitment of the candidate TOP transcripts to cardiomyocyte polysomes in response to ET-1 either (Additional files 8 and 9). This perhaps highlights the necessity (currently) for an experimentally driven approach.

### Protein kinase signaling and translational regulation

PI3K signaling and mTOR are implicated in the regulation of TOP mRNAs and mRNA translation in general [1]. Our data are consistent with this. Thus, basal cardiomyocyte protein synthesis and PKB/Akt phosphorylation [18], was attenuated by inhibiting PI3K with LY294002 (Figure 1, E and 1F) indicating that, even in the basal state, turnover of phosphatidylinositol 3,4,5 *tris*phosphate plays an important maintenance role. Furthermore, the increase in protein synthesis induced by ET-1 or insulin was attenuated by LY294002 or rapamycin (Figure 1F). Our aim was to highlight differential signaling by ET-1 and insulin in relation to protein synthesis rather than dissect the involvement of signaling pathways in translational regulation *per se*. Thus, ET-1 potently activated ERK1/2 rather than PKB/Akt whereas insulin activated PKB/Akt rather than ERK1/2 (Figure 1A), but the pathways appear to converge and either stimulus promoted phosphorylation of mTOR, p70S6Ks and Rps6 (Figure 1B). The data are consistent with studies in other cells that implicate ERK1/2 signaling in the regulation of mTOR, p70S6Ks and Rps6 in addition to PI3K (see, for example, [29-31]), and both ET-1 and insulin promote phosphorylation of mTOR in feline cardiomyocytes [20]. However, the significance of this for translational regulation of TOP mRNAs or, indeed, translational regulation by any other mechanism is not clear.

Regardless of the signaling mechanism, there was little similarity in the responses to ET-1 and insulin, both with respect to the global transcriptome and the translatome (Figure 4, Additional files 4 and 5). This contrasts with a study in feline cardiomyocytes suggesting that either stimulus promotes recruitment of TOP mRNAs to polysomes for translation [20]. It should perhaps be considered that only Rpl32 mRNA was studied, as an example of a TOP mRNA, and the global picture was not defined. In our study, ET-1 did promote some increase in recruitment of TOP mRNAs for ribosomal proteins to cardiomyocyte polysomes (Additional file 6) but, overall, the response was less than that of insulin. Potentially, the degree of the response correlates with the lesser activation of mTOR by ET-1 than insulin (Figure 1, B and 1C), but we have little understanding of the mTOR signaling pathway in cardiomyocytes on which to base such a claim.

Though outside the scope of this study, further work is clearly required to establish the mechanisms by which insulin or ET-1 influence mRNA recruitment to cardiomyocyte polysomes. Given the likely importance of mTOR and the discrepancies in the literature with respect to mTOR regulation and sensitivity or otherwise to rapamycin [1,32,33], perhaps the most immediate focus should be the rapamycin sensitivity (or otherwise) of polysomal recruitment of TOP *vs *non-TOP mRNAs in the cardiomyocyte response to insulin or ET-1. However, more fundamental studies are also required simply to understand the PI3K signaling pathway in cardiomyocytes. Some of the important questions to be addressed relate to compartmentalisation of signaling events to establish whether, for example, activation of PI3K and/or mTOR by insulin is confined to a specific location and whether mTOR activated by ET-1 constitutes a distinct pool. Such spatial organisation could explain differential effects of the two stimuli despite a similar global level of mTOR activation. Even more fundamentally, perhaps it is worth noting that although PKB/Akt and mTOR are well-established effectors of PI3K signalling and most studies use PKB/Akt as an indicator of PI3K activation, PI3K is a family of enzymes with many lipid (and potentially protein) substrates that seem likely to signal through a range of effectors. This area is largely uninvestigated in cardiomyocytes and is underinvestigated in other cells, but future studies such as these may prove essential in understanding the role of PI3K in translational regulation.

## Conclusions

In summary, ET-1 substantially affects global transcript expression whereas insulin has a more subtle effect both in terms of relative changes and breadth of the response, and effects of either ET-1 or insulin on RNA recruitment to polysomes are more subtle with differential effects of the two agonists on specific transcripts. Notably, insulin stimulates recruitment of established TOP mRNAs to cardiomyocyte polysomes. Although we identified some novel TOP mRNAs, we could not identify a TOP sequence in the 5' UTRs of ~43% of the mRNAs for established proteins with selective upregulation in the insulin-responsive cardiomyocyte translatome. This suggests that the TOP mRNA response is largely confined to mRNAs encoding components of the translational apparatus, and other mechanisms of translational regulation operate in parallel. We suggest that these differences result from activation of different signaling pathways and may account for the overt hypertrophy induced by ET-1 compared with a "maintenance" effect of insulin. However, with the complexities of the signaling and transcriptional pathways with signal integration at specific nodes, further studies are clearly required to dissect the underlying mechanisms.

## Methods

### Primary cultures of neonatal rat ventricular myocytes and preparation of cardiomyocyte polysomes

Cardiomyocytes were cultured from 1-3 day neonatal rat hearts as previously described [21]. Cells were plated at confluence on Primaria tissue culture dishes in 15% (v/v) fetal calf serum (18 h) and serum was withdrawn (24 h) prior to stimulation. Cardiomyocytes were left unstimulated (Controls), exposed to 100 nM ET-1 (Bachem), 50 mU/ml insulin (Actrapid^® ^Novo Nordisk A/S, Denmark), 1 μM rapamycin (Calbiochem^®^, Merck Chemicals), 50 μM LY294002 (Enzo Life Sciences) or 2 μM PD184352 (Enzo Life Sciences) or exposed to ET-1 or insulin following pre-treatment (10 min) with rapamycin, LY294002 or PD184352. Cardiomyocyte polysomes (16 × 10^6 ^cells per sample) were prepared by sucrose density centrifugation as previously described [21]. Fractions (12 in total) were collected by upward displacement whilst monitoring absorbance at 254 nm. Fractions 6-11 were pooled for the preparation of polysomal RNA.

### Immunoblotting

Immunoblotting of cardiomyocyte nuclear extracts was performed essentially as described [34]. For all analyses other than total/phosphorylated mTOR, proteins (4 × 10^5 ^cells for phosphorylated and total ERK1/2 and PKBAkt blots; 2 × 10^5 ^cells for phosphorylated and/or total p70S6K, Rps3 and Rps6) were separated on 10% (w/v) polyacrylamide gels with electrophoresis for 45 min at 200 V. Proteins were transferred to nitrocellulose using standard transfer buffer (25 mM Tris, 192 mM glycine, 20% (v/v) methanol). For mTOR, proteins (2 × 10^5 ^cells) were separated on 8% (w/v) polyacrylamide gels with electrophoresis at 100 V until the dye front reached the bottom of the gel followed by 200 V for 2 h. Proteins were transferred to nitrocellulose using transfer buffer for high molecular weight proteins (48 mM Tris, 39 mM glycine, 1.3 mM SDS). Primary antibodies [phospho-ERK1/2 (cat. no. 4377), phospho(Ser473)-PKB/Akt and phospho(Thr308)-PKB/Akt (cat. nos. 9271 and 9275, respectively; these antibodies were used in combination), phospho(Ser2448)-mTOR (cat. no. 2971), total mTOR (cat. no. 2983), phospho(Ser235/236)-Rps6 (cat. no. 4858), Rps6, total Rps3 (cat. nol. 2579)] were from Cell Signaling Inc. and were used at 1/1000 dilution. Secondary antibodies conjugated to horseradish peroxidase were from Dako and used at 1/5000 dilution.

### **Protein synthesis assays**

To study the rate of protein synthesis at specific times, myocytes (2 × 10^6 ^cells per 35 mm dish) were incubated 100 nM ET-1 or 50 mU/ml insulin for a total of 1, 2 or 7 h with addition of L-[2,3,4,5,6-^3^H]-Phe (American Radiolabeled Chemicals Inc.) for the last 1 h of the incubation. For inhibitor studies, myocytes were exposed to each inhibitor alone (50 μM LY294002, 1 μM rapamycin, or 2 μM PD184352), ET-1 or insulin, or ET-1 or insulin in the presence of each inhibitor for a total of 2 h with addition of L-[2,3,4,5,6-^3^H]-Phe for the last 1 h of the incubation. Myocytes were washed (PBS, 1 ml, 4°C) and scraped into 1 ml 0.2 mM NaOH. A sample (15 μl) was taken to determine total protein (Biorad Bradford assay [35]). Bovine serum albumin (0.1 ml, 100 mg/ml) was added to the remaining sample, proteins were precipitated with 10% (w/v) trichloroacetic acid (6 ml) and samples were centrifuged. Precipitates were washed [10% (w/v) trichloroacetic acid (3 × 5 ml)], NaOH added (10 μl, 10 M), and the pellets were dissolved in 1.8 ml Soluene (Perkin Elmer UK) before scintillation counting using Ultima Gold scintillation fluid (Perkin Elmer UK). Experiments were performed in duplicate, the mean values were taken and corrected according to total protein. The data are presented as means ± SEM of these values.

### RNA preparation and microarray analysis

The total and polysomal RNA samples for controls and ET-1 (1 h) from our previous study [21] were hybridised to Affymetrix rat genome 230 2.0 microarrays simultaneously with samples treated with insulin (50 mU/ml, 1 h). Here, the full dataset was analysed using GeneSpring GX 10.0.2 (previously, we used GeneSpring GX 7.3.1).

Total RNAs (4 × 10^6 ^cells per sample) and polysomal RNAs (16 × 10^6 ^cells per sample) were prepared from unstimulated myocytes and myocytes exposed to ET-1 (100 nM, 1 h) or insulin (50 mU/ml, 1 h). RNAs were prepared from 12 separate myocyte preparations, individual samples were generated by combining equal amounts of RNA from three myocyte preparations and four sets of samples were hybridised to individual microarrays. RNA preparation and microarray hybridizations were performed as previously described [21]. The data are available from ArrayExpress (accession numbers: E-MIMR-681 for ET-1 data; E-MEXP-2527 for insulin data; the controls are included in each dataset). The .CEL files were imported into GeneSpring GX 10.0.2 for analysis using MAS 5.0 summarisation with normalisation to unstimulated control samples (analysis of changes induced by ET-1 or insulin), no baseline normalisation (comparison of raw expression values in total RNA *vs *polysomal RNA in control cells) or normalisation to gene median (identification of differential representation of transcripts in total and polysomal RNA pools in control cells). A confidence filter was applied (P/M in 100% of any condition; >50 raw value), and probesets selected according to relative level of expression followed by statistical testing (one-way ANOVA with Tukey post-test or t-test as indicated) with FDR < 0.05 (Benjamini and Hochberg correction for multiple testing). For agonist-responsive RNAs, supervised clustering was performed according to relative changes in expression (upregulation or downregulation by ET-1 and/or insulin). Heatmaps were generated with GeneSpring GX 10.0.2 using a Euclidean complete correlation. To confirm statistical significance for cluster sets, normalised values were exported and analysed by GraphPad Prism 4.

### Bioinformatics

All gene identities were confirmed by BLAST search of the probeset sequences using the Entrez nucleotide databases [36]. BLAST searches for unassigned sequences were performed against the rat genome [37] and, since the rat genome is still less well annotated, the mouse genome [38] (cross-species megaBLAST). Genes were classified as far as possible using GeneOntology classifications associated with rat, mouse and human orthologs [39], taking into account both probable Function and Process. For genes with conflicting potential functions, further literature searches were performed to ascertain probable biochemical function. Genes were grouped according to their biochemical function in the cell. Transcriptional start sites for mouse orthologs were determined using the FANTOM3 database [40], selecting for RIKEN clones with confirmation of a complete 5' UTR. For mouse and human orthologs, we also used the Database of Transcriptional Start Sites [41]. The predicted minimum fold energy of 5'UTR sequences was determined using the Vienna RNAfold WebServer [42]. Because of the large number of pseudogenes for ribosomal proteins in mammalian genomes [43], in some cases the gene is not correctly identified on the rat genome. In these cases, we identified the correct gene by its exon structure and position relative to adjacent genes identified on the mouse and human genomes.

### Quantitative reverse transcription-polymerase chain reaction (qPCR)

Primers for qPCR were designed for established genes using published rat sequences and PrimerExpress software for Real-Time PCR (version 3.0; Applied Biosystems) (Table 2). Where possible, these were designed across an exon boundary. cDNA was prepared by reverse transcription of RNA samples and qPCR performed using a 7500 Real-Time PCR System (Applied Biosystems). A master-mix with (per reaction) 12.5 μl Sybr-Green Jump Start Taq Readymix (Sigma-Aldrich) and 5 μl oligonucleotides (5 pmol each of forward and reverse primers) was aliquoted into Optical 96-well reaction-plates (Applied Biosystems), and cDNA template added (7.5 μl, 1/15 dilution in water). PCR conditions were 50°C for 2 min, 95°C for 10 min, followed by 40 cycles of 95°C for 15 s and 59°C for 60 s. Dissociation curve analysis was performed to check for aberrant amplification products. An absolute quantitation protocol was used and data were normalised to Gapdh where indicated.

**Table 2:** Primers used for qPCR.

Gene	Accession no.	Size (bp)	Forward primer	Reverse primer
Klf2	NM_001007684	96	CACACAGGTGAGAAGCCTTATCAT (874-897)	CCGTGTGCTTGCGGTAGTG (952-970)
Klf6	NM_031642	89	GCTCCCACTTGAAAGCACATC (641-661)	TTCTTGCAAAACGCCACTCA (710-729)
Klf10	NM_031135	149	CCATGAGCTGCGACTGGAA (183-201)	TAAGGTGGAGTCAAACAGAATGCT (307-331)
Klf11	NM_001037354	85	CCTGATCTACCAAAGGACTTCCA (311-333)	CTCATGGAGCCAACAGGGA (377-395)
Klf15	NM_053536	65	TGCGGCTGGAGGTTTTCA (1347-1364)	TTCACACCCGAGTGAGATCGT (1391-1411)
Klf16	NM_001127604	79	TCACACCTGCGGACTCACA (516-534)	CGGAACGGGCGAACTTCT (577-594)
Rps3	NM_001009239	94	AAAGTGGCCACAAGAGGTCTGT (280-301)	CATAGCAGGCCCTTCGAACT (354-373)
Rps6	NM_017160	82	AAGGTAAGAAGCCCAGGACCAA (509-530)	ATACGTCGGCGTTTGTGTTG (571-590)

mRNA sequences (accession numbers provided) for established genes were obtained from the Rat Genome Database (http://rgd.mcw.edu, viewed at http://www.ncbi.nlm.nih.gov/entrez). Nucleotide positions are shown in parentheses for each primer.

### 18S/28S rRNA quantification by Agilent Bioanalyser

Quantitative analysis of 18S/28S ribosomal RNA was performed with an Agilent 2100 Bioanalyser using the RNA 6000 Nano kit (Agilent Technologies, Palo Alto, CA, USA). Total RNA was prepared according to the Agilent 2100 Bioanalyser protocol, loaded into the NanoChip and processed for 30 min. Electropherograms were analyzed according to the manufacturer's protocol and the 18S and 28S ribosomal peak areas were integrated.

### Statistical analysis

Statistical analysis of microarray data was performed using GeneSpring GX 10.0.2 (one-way ANOVA with Tukey post-test or t-test as indicated) using FDR < 0.05 (Benjamini and Hochberg correction for multiple testing). Statistical analysis of qPCR, protein synthesis and immunoblotting data was performed using GraphPad Prism 4.

## List of abbreviations

ERK: Extracellular signal-regulated kinase; mTOR: mammalian target of rapamycin; mTORC: mTOR complex; p70S6K: p70 ribosomal S6 kinase; p90RSK: p90 ribosomal S6 kinase; PI3K: phosphatidylinositol 3' kinase; TOP: terminal oligopyrimidine tract; uORF: upstream open reading frame; UTR: untranslated region

## Authors' contributions

TM prepared the cardiomyocyte polysomes, prepared the RNA for microarray hybridisation and conducted qPCR validation studies. AKM conducted qPCR validation studies. TEC assisted with the RNA preparation for microarray hybridisation. ELT prepared the cardiomyocytes and performed the immunoblotting studies. PHS and AC wrote participated in the design and coordination of the project and drafted the manuscript. AC was responsible for the microarray data analysis and conceived the project. All authors have read and approved the final manuscript.

## Supplementary Material

Additional file 1 **Regulation of the cardiomyocyte global transcriptome by ET-1 or insulin (Microsoft Word Table)**. Cardiomyocytes were unstimulated (control), or exposed to 100 nM ET-1 or 50 mU/ml insulin (1 h). Total RNA expression was determined using microarrays. Transcripts were identified with significant changes in expression (>1.5-fold change induced by ET-1 or insulin relative to controls; FDR < 0.05, * ET-1 *vs *control; # Insulin *vs *control; † ET-1 *vs *insulin, one-way ANOVA with Tukey post-test and Benjamini-Hochberg FDR correction). Mean raw values are given for controls with the mean change relative to controls for ET-1 or insulin (n = 4). For transcripts represented by more than one probeset, the probesets and corresponding raw values are listed with the average of the mean relative change. RNA responses are clustered according to up- or down-regulation, and the effect of ET-1 and/or insulin. Clusters (i) - (viii) correspond to the summarised data in Figure 2A of the associated manuscript. Clusters are colour-coded according to response to ET-1 and insulin (green), ET-1 only (yellow) or insulin only (cyan). AS, Antisense. Click here for file

Additional file 2 **Functional classifications of the top 10th percentile RNAs identified in neonatal rat cardiomyocytes (Microsoft Word Table)**. Total and polysomal RNA from unstimulated neonatal rat cardiomyocytes (serum-starved, 20 h) were analysed using microarrays. Results are the mean raw fluorescence values (n = 4). For transcripts represented by more than one probeset, the probesets and mean corresponding raw values are listed. Differential expression in polysome *vs *total RNA was analysed by t-test with Benjamini-Hochberg FDR correction (*p < 0.05). RNAs are grouped according to general functional categories (summarised in Figure 3C of the associated manuscript) with detailed functions within the Table. Click here for file

Additional file 3 **Differential expression of cardiomyocyte transcripts in polysomal and total RNA (Microsoft Word Table)**. Total and polysomal RNA from unstimulated neonatal rat cardiomyocytes were analysed using microarrays. The data were normalised to the gene median. Transcripts with differential expression in total and polysomal RNA pools were identified (t-test with Benjamini-Hochberg FDR correction p < 0.05; >3-fold difference). Mean raw fluorescence and normalised values are given (n = 4). For transcripts represented by more than one probeset, the probesets and mean corresponding raw values are listed. RNAs are listed according to relative expression in polysomal or total pools and whether they are protein-coding, non-protein-coding or associated with no known gene. AS = Antisense. Click here for file

Additional file 4 **Regulation of polysomal and total RNA expression in cardiomyocytes in response to insulin (Microsoft Word Table)**. Neonatal rat cardiomyocytes were exposed to insulin (50 mU/ml, 1 h) or left unstimulated (controls). Total and polysomal RNA were analysed using microarrays. The data were normalised to controls. Transcripts with differential expression in insulin-treated cells relative to controls were identified (>1.25-fold difference; * FDR < 0.05 insulin *vs *controls for polysomal RNA, # FDR < 0.05 insulin *vs *controls for total RNA, t-test with Benjamini and Hochberg false discovery rate correction). Mean raw fluorescence values are provided for controls, with mean expression relative to controls for insulin-treated cells (n = 4). For transcripts represented by more than one probeset, the probesets and corresponding raw values are listed. RNAs are listed according to translational regulation and in order of functional category then alphabetical order of the gene symbol. AS = Antisense. Click here for file

Additional file 5 **Regulation of polysomal and total RNA expression in cardiomyocytes in response to ET-1 (Microsoft Word Table)**. Neonatal rat cardiomyocytes were exposed to ET-1 (100 nM, 1 h) or left unstimulated (controls). Total and polysomal RNA were analysed using Affymetrix rat genome 230 2.0 microarrays. The data were normalised to controls. Transcripts with differential expression in ET-1-treated cells relative to controls were identified (>1.25-fold difference; * FDR < 0.05 ET-1 *vs *Controls for polysomal RNA, # FDR < 0.05 ET-1 *vs *Controls for total RNA, t-test with Benjamini and Hochberg false discovery rate correction). Mean raw fluorescence values are provided for controls, and mean expression relative to controls is provided for ET-1-treated cells (n = 4). For transcripts represented by more than one probeset, the probesets and mean corresponding raw values are listed. RNAs are listed according to translational regulation and in order of functional category then alphabetical order of the gene symbol. AS = Antisense. Click here for file

Additional file 6 **Recruitment of TOP mRNAs to polysomes in cardiomyocytes in response to insulin (Microsoft Excel Spreadsheet)**. Neonatal rat cardiomyocytes were exposed to insulin (50 mU/ml, 1 h) or left unstimulated (controls). Total and polysomal RNA were analysed using microarrays. Probesets corresponding to ribosomal protein mRNAs and other established TOP mRNAs were identified. The mean raw values are provided for controls with the mean fold change (n = 4) induced by insulin relative to controls. *FDR < 0.05 for polysomal RNA from insulin-treated cells *vs *controls (t-test with Benjamini and Hochberg false discovery rate correction). Mouse and rat 5' UTR sequences up to and including the initiation codon are provided. Sequence identification used FANTOM3 and NCBI databases and the database of transcriptional start sites (DBTSS). The TOP sequence is shown in red. [N.B. For some rat genes, a pseudogene was identified on the NCBI site; the correct gene was identified by comparison with the mouse genome on the basis of intron structure and location relative to adjacent genes (#). For other rat genes, the 5' UTR was short and the genomic sequence was used to identify regions of homology with the mouse genome (†)]. Click here for file

Additional file 7 **Recruitment of probable non-TOP mRNAs to polysomes in cardiomyocytes in response to insulin (Microsoft Excel Spreadsheet)**. Neonatal rat cardiomyocytes exposed to insulin (50 mU/ml, 1 h) or left unstimulated (controls). Total and polysomal RNA were analysed using microarrays. Probesets corresponding to mRNAs with >1.25-fold increase in expression in polysomal RNA (but not total RNA) in response to insulin (FDR < 0.05, t-test with Benjamini -Hochberg false discovery rate correction) were identified. Mean raw fluorescence values are given for the controls with the mean fold change (n = 4) induced by insulin relative to controls. Mouse 5' UTR sequences up to and including the initiation codon are provided. Sequence identification used FANTOM3 and NCBI databases in addition to the database of transcriptional start sites (DBTSS). Presence (Y) or absence (N) of polypyrimidine or GC tracts, upstream open reading frames (uORFs), or 5' untranslated exons are indicated, in addition to GC content (%), UTR length and predicted minimum free energy (Mfe). Click here for file

Additional file 8 **Response of putative TOP mRNAs to insulin stimulation in cardiomyocytes (Microsoft Excel Spreadsheet)**. Neonatal rat cardiomyocytes were exposed to insulin (50 mU/ml, 1 h) or ET-1 (100 nM, 1 h) or left unstimulated (controls). Total and polysomal RNA prepared were analysed using Affymetrix rat genome 230 2.0 microarrays. Probesets corresponding to TOP mRNAs identified by Yameshita et al. (Nuclei Acids Res. (2008) 36:3707-3715) were identified and the microarray data mined for the cardiomyocyte response. Results are the mean fold change (n = 4) in response to insulin or ET-1 relative to controls. Mean raw fluorescence values are given for the controls. Click here for file

Additional file 9 **Recruitment of probable non-TOP mRNAs to polysomes in cardiomyocytes in response to ET-1 (Microsoft Excel Spreadsheet)**. Neonatal rat cardiomyocytes exposed to ET-1 (100 nM, 1 h) or left unstimulated (controls). Total and polysomal RNA were analysed using microarrays. Probesets corresponding to mRNAs with >1.25-fold increase in expression in polysomal RNA (but not total RNA) in response to ET-1 (FDR < 0.05, t-test with Benjamini -Hochberg false discovery rate correction) were identified. Mean raw fluorescence values are given for the controls with the mean fold change (n = 4) induced by insulin relative to controls. Mouse 5' UTR sequences up to and including the initiation codon are provided. Sequence identification used FANTOM3 and NCBI databases in addition to the database of transcriptional start sites (DBTSS). Presence (Y) or absence (N) of polypyrimidine or GC tracts, upstream open reading frames (uORFs), or 5' untranslated exons are indicated, in addition to GC content (%), UTR length and predicted minimum free energy (Mfe). Click here for file
