# Supplementary material for: Regulation of the cardiomyocyte transcriptome vs translatome by endothelin-1 and insulin: translational regulation of 5' terminal oligopyrimidine tract (TOP) mRNAs by insulin
Source: BMC Genomics. 2010 May 29;11:343. doi: 10.1186/1471-2164-11-343 (PMC2900265; doi:10.1186/1471-2164-11-343)
Supplement: Additional file 1 — Regulation of the cardiomyocyte global transcriptome by ET-1 or insulin (Microsoft Word Table). Cardiomyocytes were unstimulated (control), or exposed to 100 nM ET-1 or 50 mU/ml insulin (1 h). Total RNA expression was determined using microarrays. Transcripts were identified with significant changes in expression (>1.5-fold change induced by ET-1 or insulin relative to controls; FDR < 0.05, * ET-1 vs control; # Insulin vs control; † ET-1 vs insulin, one-way ANOVA with Tukey post-test and Benjamini-Hochberg FDR correction). Mean raw values are given for controls with the mean change relative to controls for ET-1 or insulin (n = 4). For transcripts represented by more than one probeset, the probesets and corresponding raw values are listed with the average of the mean relative change. RNA responses are clustered according to up- or down-regulation, and the effect of ET-1 and/or insulin. Clusters (i) - (viii) correspond to the summarised data in Figure 2A of the associated manuscript. Clusters are colour-coded according to response to ET-1 and insulin (green), ET-1 only (yellow) or insulin only (cyan). AS, Antisense. [file 1471-2164-11-343-S1.DOC]

**Additional file 1. Regulation of the cardiomyocyte global transcriptome by ET-1 or insulin.**  Cardiomyocytes were unstimulated (control), or exposed to 100 nM ET-1 or 50 mU/ml insulin (1 h). Total RNA expression was determined using microarrays. Transcripts were identified with significant changes in expression (>1.5-fold change induced by ET-1 or insulin relative to controls; FDR<0.05, * ET-1 *vs* control; # Insulin *vs* control; † ET-1 *vs* insulin, one-way ANOVA with Tukey post-test and Benjamini-Hochberg FDR correction). Mean raw values are given for controls with the mean change relative to controls for ET-1 or insulin (n=4). For transcripts represented by more than one probeset, the probesets and corresponding raw values are listed with the average of the mean relative change. RNA responses are clustered according to up- or down-regulation, and the effect of ET-1 and/or insulin. Clusters (i) - (viii) correspond to the summarised data in Figure 2A of the associated manuscript. Clusters are colour-coded according to response to ET-1 and insulin (green), ET-1 only (yellow) or insulin only (cyan). AS, Antisense.

| **Probeset** | **Gene Symbol** | **Function** | **Control**  **(raw values)** | **ET-1** | **Insulin** | **Cluster** |  |
| --- | --- | --- | --- | --- | --- | --- | --- |
| 1389573_at | Chac1 | **Cation transport** | 764 | 2.94 | 2.32 | (i) | *# |
| 1375852_at | Hmgcr | **Lipid metabolism** | 2051 | 1.82 | 1.54 | (i) | *# |
| 1395129_at | Rbm15 | **RNA binding** | 270 | 1.90 | 1.62 | (i) | *# |
| 1386662_at | Sesn2 | **Redox/detoxification** | 341 | 1.82 | 1.62 | (i) | *# |
| 1371953_at | Ccng2 | **Cell cycle** | 904 | 0.58 | 0.35 | (ii) | *#† |
| 1390024_at | Clec2d/g | **Receptors** | 1901 | 0.62 | 0.57 | (ii) | *# |
| 1386460_x_at | Dnm1l | **Protein transport** | 253 | 0.67 | 0.61 | (ii) | *# |
| 1368249_at, 1381396_s_at | Klf15 | **Transcriptional regulation** | 941, 300 | 0.41 | 0.58 | (ii) | *# |
| 1397681_at | RGD1305793 | **Hypothetical proteins** | 649 | 0.52 | 0.57 | (ii) | *# |
| 1367862_at | Rrad | **G proteins** | 2283 | 0.64 | 0.63 | (ii) | *# |
| 1369268_at | Atf3 | **Transcriptional regulation** | 475 | 13.91 | 1.84 | (iii) | *#† |
| 1389402_at | Axud1 | **Apoptosis** | 314 | 8.97 | 1.58 | (iii) | *#† |
| 1368860_at | Dnaja1 | **Chaperone/protein folding** | 181 | 7.10 | 1.51 | (iii) | *#† |
| 1369545_at, 1392791_at | Egr3 | **Transcriptional regulation** | 179, 172 | 28.36 | 1.87 | (iii) | #† |
| 1368489_at | Fosl1 | **Transcriptional regulation** | 295 | 6.08 | 1.57 | (iii) | *#† |
| 1387548_at | Has2 | **Carbohydrate metabolism** | 413 | 9.38 | 1.56 | (iii) | *#† |
| 1375475_at | Intron: Dusp5 | **Sequences in introns** | 64 | 6.35 | 1.88 | (iii) | #† |
| 1376569_at, 1386041_a_at, 1394068_x_at | Klf2 | **Transcriptional regulation** | 673, 141, 137 | 3.18 | 1.52 | (iii) | *#† |
| 1393728_at | Lif | **Agonists** | 291 | 10.57 | 2.11 | (iii) | *#† |
| 1368519_at, 1392264_s_at | Serpine1 | **Proteolysis** | 480, 153 | 19.08 | 1.69 | (iii) | *#† |
| 1371754_at | Slc25a25 | **Phosphate transport** | 295 | 4.06 | 1.90 | (iii) | *#† |
| 1384162_at | Csnrp2 | **Apoptosis** | 305 | 1.07 | 1.62 | (iv) | #† |
| 1389404_at | Fkhl18 | **Transcriptional regulation** | 385 | 0.72 | 1.52 | (iv) | #† |
| 1385359_at | Fscn1 | **Actin structures** | 387 | 1.40 | 1.58 | (iv) | # |
| 1387028_a_at | Id1 | **Transcriptional regulation** | 3549 | 0.71 | 1.67 | (iv) | *#† |
| 1391710_at | Intron: Nme2 | **Sequences in introns** | 345 | 1.16 | 1.59 | (iv) | *#† |
| 1368650_at | Klf10 | **Transcriptional regulation** | 1399 | 0.90 | 1.95 | (iv) | #† |
| 1376581_at | RGD1309138 | **Hypothetical proteins** | 184 | 1.12 | 1.52 | (iv) | #† |
| 1383337_at | RGD1311055 | **Hypothetical proteins** | 210 | 0.96 | 1.56 | (iv) | #† |
| 1372316_at | RGD1311939 | **Hypothetical proteins** | 756 | 1.08 | 1.68 | (iv) | #† |
| 1383253_at | Slc30a1 | **Zinc transport** | 911 | 1.00 | 1.68 | (iv) | #† |
| 1385978_at | Sox9 | **Transcriptional regulation** | 422 | 1.01 | 1.77 | (iv) | #† |
| 1379488_at | Trp53rk | **Kinases/kinase regulators** | 271 | 1.17 | 1.55 | (iv) | #† |
| 1373583_at | Unknown | **No established gene** | 560 | 1.29 | 1.61 | (iv) | *#† |
| 1389230_at, 1394948_at | Arrdc3 | **Unknown function** | 2632, 666 | 0.83 | 0.58 | (v) | #† |
| 1384260_at | Frat2 | **Kinases/kinase regulators** | 914 | 1.21 | 0.39 | (v) | #† |
| 1381798_at | Lmo7 | **Cell-cell adhesion/interaction** | 492 | 1.07 | 0.55 | (v) | #† |
| 1378074_at | Pdk4 | **Energy metabolism** | 504 | 0.88 | 0.45 | (v) | #† |
| 1370381_at | Pnrc1 | **Transcriptional regulation** | 3415 | 0.97 | 0.49 | (v) | #† |
| 1390881_at | Abra | **G proteins** | 477 | 2.97 | 0.84 | (vi) | *#† |
| 1368223_at | Adamts1 | **Proteolysis** | 1415 | 2.52 | 0.87 | (vi) | #† |
| 1374610_at | Agpat9 | **Lipid metabolism** | 432 | 2.10 | 1.10 | (vi) | #† |
| 1373866_at, 1382206_a_at, 1385566_at | Akap2 | **Adaptors/scaffold proteins** | 1485, 790, 207 | 2.35 | 1.25 | (vi) | #† |
| 1372069_at | Ankrd15 | **Unknown function** | 2390 | 1.53 | 1.01 | (vi) | #† |
| 1386065_at | Ankrd57 | **Unknown function** | 269 | 2.09 | 1.33 | (vi) | *#† |
| 1367614_at | Anxa1 | **Unknown function** | 4962 | 1.67 | 0.93 | (vi) | #† |
| 1377750_at | Arhgef3 | **G proteins** | 359 | 3.28 | 1.05 | (vi) | #† |
| 1379311_at, 1380772_at, 1397437_at | Arid5a | **Transcriptional regulation** | 406, 149, 249 | 2.30 | 0.97 | (vi) | #† |
| 1372964_at | Arid5b | **Transcriptional regulation** | 1935 | 1.94 | 0.71 | (vi) | *#† |
| 1376275_at | Arl5b | **G proteins** | 643 | 1.74 | 1.12 | (vi) | #† |
| 1382778_at | AS: Dusp6 | **Potential AS sequences** | 685 | 2.46 | 1.07 | (vi) | #† |
| 1385961_at | AS: Klf5 | **Potential AS sequences** | 206 | 3.18 | 0.70 | (vi) | #† |
| 1375676_at | AS: Lin7c | **Potential AS sequences** | 370 | 1.60 | 1.40 | (vi) | *#† |
| 1374429_at | AS: Pim1 | **Potential AS sequences** | 1425 | 2.01 | 0.59 | (vi) | *#† |
| 1398037_at | AS: Plk2 | **Potential AS sequences** | 128 | 2.83 | 0.92 | (vi) | #† |
| 1379747_at | AS: Prss35 | **Potential AS sequences** | 425 | 1.53 | 1.09 | (vi) | #† |
| 1382171_at | AS: Tsc22d2 | **Potential AS sequences** | 1040 | 2.98 | 1.19 | (vi) | *#† |
| 1367624_at | Atf4 | **Transcriptional regulation** | 5811 | 1.50 | 1.32 | (vi) | *#† |
| 1373494_at, 1389465_at | Bcr | **Other signalling** | 996, 984 | 2.66 | 1.02 | (vi) | #† |
| 1398270_at | Bmp2 | **Agonists** | 491 | 2.36 | 1.17 | (vi) | #† |
| 1371719_at | Brd2 | **Kinases/kinase regulators** | 863 | 1.55 | 1.46 | (vi) | * |
| 1379935_at | Ccl7 | **Agonists** | 2730 | 1.61 | 1.19 | (vi) | #† |
| 1368050_at | Ccnl1 | **Cell cycle** | 943 | 1.89 | 1.00 | (vi) | #† |
| 1368921_a_at | Cd44 | **Cell-cell adhesion/interaction** | 994 | 1.51 | 1.09 | (vi) | #† |
| 1375910_at | Cdc42ep3 | **G proteins** | 3587 | 2.02 | 1.20 | (vi) | *#† |
| 1387391_at | Cdkn1a | **Kinases/kinase regulators** | 542 | 2.18 | 1.35 | (vi) | *#† |
| 1374139_at | Cdr2 | **Unknown function** | 609 | 2.11 | 1.49 | (vi) | *# |
| 1389119_at | Cmya1 | **Actin structures** | 2419 | 2.34 | 1.10 | (vi) | #† |
| 1389368_at | Cnksr3 | **Kinases/kinase regulators** | 1075 | 1.83 | 0.92 | (vi) | #† |
| 1378925_at | Crem | **Transcriptional regulation** | 318 | 2.01 | 0.90 | (vi) | #† |
| 1367631_at | Ctgf | **Agonists** | 12903 | 1.83 | 0.82 | (vi) | *#† |
| 1382873_at | Cttnbp2nl | **Unknown function** | 548 | 1.66 | 1.10 | (vi) | #† |
| 1387316_at | Cxcl1 | **Agonists** | 3361 | 1.53 | 0.86 | (vi) | #† |
| 1373525_at, 1379666_at | Dcun1d3 | **Unknown function** | 327, 296 | 2.09 | 1.26 | (vi) | #† |
| 1383302_at | Dnajb1 | **Chaperone/protein folding** | 1034 | 1.53 | 1.35 | (vi) | *# |
| 1372722_at | Dnajb4 | **Chaperone/protein folding** | 1516 | 2.35 | 0.89 | (vi) | #† |
| 1384511_at | Dnajb5 | **Chaperone/protein folding** | 354 | 1.57 | 1.14 | (vi) | #† |
| 1388589_at | Dot1l | **DNA structure and repair** | 602 | 2.78 | 1.19 | (vi) | #† |
| 1368146_at, 1368147_at | Dusp1 | **Phosphatases** | 2320, 237 | 2.68 | 0.73 | (vi) | *#† |
| 1394028_at | Dusp10 | **Phosphatases** | 247 | 2.08 | 0.90 | (vi) | #† |
| 1377023_at | Dusp2 | **Phosphatases** | 444 | 1.72 | 1.15 | (vi) | #† |
| 1393119_at | Dusp4 | **Phosphatases** | 1412 | 1.96 | 1.01 | (vi) | #† |
| 1368124_at | Dusp5 | **Phosphatases** | 681 | 4.80 | 1.08 | (vi) | #† |
| 1377064_at, 1387024_at | Dusp6 | **Phosphatases** | 1350, 2253 | 3.47 | 1.18 | (vi) | #† |
| 1372385_at | Dusp8 | **Phosphatases** | 411 | 1.56 | 1.17 | (vi) | * |
| 1371840_at | Edg1 | **Receptors** | 4796 | 1.58 | 1.02 | (vi) | #† |
| 1369519_at | Edn1 | **Agonists** | 276 | 1.64 | 0.93 | (vi) | #† |
| 1383353_at | Efnb2 | **Cell-cell adhesion/interaction** | 537 | 1.63 | 1.20 | (vi) | #† |
| 1369736_at, 1371527_at | Emp1 | **Other signalling** | 1390, 4530 | 1.72 | 1.05 | (vi) | #† |
| 1373535_at, 1396450_at | Enah | **Actin structures** | 2108, 371 | 1.69 | 1.11 | (vi) | #† |
| 1374143_at | Epha2 | **Cell-cell adhesion/interaction** | 288 | 2.15 | 1.38 | (vi) | #† |
| 1369587_at, 1385150_at | Ereg | **Agonists** | 160, 158 | 3.62 | 1.39 | (vi) | #† |
| 1369182_at | F3 | **Receptors** | 190 | 4.57 | 1.36 | (vi) | *#† |
| 1377938_at | Fam100a | **Hypothetical proteins** | 963 | 1.67 | 0.94 | (vi) | #† |
| 1380696_at | Fam102b | **Hypothetical proteins** | 520 | 1.53 | 1.09 | (vi) | #† |
| 1383295_at | Fam110a | **Hypothetical proteins** | 162 | 1.73 | 0.92 | (vi) | #† |
| 1385251_at | Fam110c | **Hypothetical proteins** | 76 | 3.82 | 1.41 | (vi) | *#† |
| 1394745_at | Fam46a | **Hypothetical proteins** | 263 | 1.60 | 1.08 | (vi) | #† |
| 1382059_at, 1392747_at | Fbxo30 | **Ubiquitin cycle** | 631, 395 | 1.70 | 1.13 | (vi) | #† |
| 1368336_at | Fdx1 | **Redox/detoxification** | 2354 | 1.82 | 0.99 | (vi) | #† |
| 1370623_at, 1383516_at, 1386637_at, 1392894_at | Fgl2 | **Extracellular matrix** | 451, 315, 268, 624 | 2.19 | 1.03 | (vi) | #† |
| 1392863_at | Flrt3 | **Cell-cell adhesion/interaction** | 172 | 1.72 | 1.41 | (vi) | *# |
| 1373035_at | Fosl2 | **Transcriptional regulation** | 1742 | 1.63 | 0.99 | (vi) | #† |
| 1372750_at, 1387843_at | Fst | **Agonists** | 496, 301 | 4.54 | 1.38 | (vi) | #† |
| 1368947_at | Gadd45a | **Cell cycle** | 2147 | 1.74 | 1.07 | (vi) | #† |
| 1372016_at | Gadd45b | **Cell cycle** | 563 | 1.64 | 0.82 | (vi) | *#† |
| 1388792_at | Gadd45g | **Cell cycle** | 1419 | 2.59 | 1.05 | (vi) | #† |
| 1373513_at | Gch1 | **Tetrahydrofolate metabolism** | 196 | 1.66 | 1.02 | (vi) | #† |
| 1372523_at | Gclc | **Redox/detoxification** | 1041 | 1.54 | 1.05 | (vi) | #† |
| 1382351_at | Gem | **G proteins** | 448 | 1.73 | 1.03 | (vi) | #† |
| 1369640_at | Gja1 | **Gap junction** | 2041 | 1.58 | 1.12 | (vi) | * |
| 1378113_at | Gm22 | **Unknown function** | 282 | 1.53 | 1.29 | (vi) | * |
| 1376828_at | Gprc5a | **Receptors** | 443 | 1.59 | 1.17 | (vi) | #† |
| 1368983_at | Hbegf | **Receptors** | 381 | 3.04 | 1.10 | (vi) | #† |
| 1369006_at | Hk2 | **Carbohydrate metabolism** | 546 | 1.66 | 0.93 | (vi) | #† |
| 1368247_at | Hspa1a/b | **Chaperone/protein folding** | 317 | 3.76 | 1.08 | (vi) | #† |
| 1370912_at | Hspa1b | **Chaperone/protein folding** | 443 | 2.63 | 1.04 | (vi) | #† |
| 1387202_at | Icam1 | **Cell-cell adhesion/interaction** | 1620 | 2.87 | 1.23 | (vi) | *#† |
| 1394022_at | Id4 | **Transcriptional regulation** | 711 | 1.70 | 1.43 | (vi) | *# |
| 1368878_at, 1388872_at | Idi1 | **Lipid metabolism** | 3111, 1817 | 1.60 | 1.24 | (vi) | *#† |
| 1372389_at | Ier2 | **Unknown function** | 637 | 3.92 | 1.36 | (vi) | *#† |
| 1388587_at | Ier3 | **Apoptosis** | 4246 | 1.66 | 1.21 | (vi) | *#† |
| 1367795_at | Ifrd1 | **Transcriptional regulation** | 1197 | 4.27 | 1.12 | (vi) | #† |
| 1387273_at | Il1rl1 | **Receptors** | 1198 | 3.86 | 1.05 | (vi) | #† |
| 1367894_at | Insig1 | **Other metabolism** | 4726 | 1.68 | 1.10 | (vi) | #† |
| 1391753_at | Intron: Ankrd1 | **Sequences in introns** | 556 | 2.14 | 1.09 | (vi) | #† |
| 1394451_at | Intron: Anxa1 | **Sequences in introns** | 325 | 2.61 | 0.81 | (vi) | #† |
| 1390723_at | Intron: Ctnna1 | **Sequences in introns** | 201 | 3.30 | 1.32 | (vi) | #† |
| 1391373_at | Intron: Enah | **Sequences in introns** | 169 | 1.52 | 1.02 | (vi) | #† |
| 1397449_at | Intron: Enah | **Sequences in introns** | 256 | 2.16 | 1.41 | (vi) | *#† |
| 1382942_at | Intron: Ext1 | **Sequences in introns** | 223 | 1.73 | 1.12 | (vi) | * |
| 1394750_at | Intron: Fhl1 | **Sequences in introns** | 119 | 3.72 | 1.20 | (vi) | #† |
| 1381343_at | Intron: Frmd6 | **Sequences in introns** | 211 | 1.52 | 0.94 | (vi) | † |
| 1385757_at | Intron: Homer1 | **Sequences in introns** | 228 | 1.59 | 1.16 | (vi) | *#† |
| 1396877_at | Intron: Lamc1 | **Sequences in introns** | 506 | 1.93 | 1.28 | (vi) | #† |
| 1379089_at, 1380155_at | Intron: Myh9 | **Sequences in introns** | 331, 755 | 2.25 | 1.33 | (vi) | #† |
| 1391215_at | Intron: Samd4 | **Sequences in introns** | 190 | 1.60 | 1.00 | (vi) | #† |
| 1382020_at | Intron: Spag9 | **Sequences in introns** | 346 | 1.64 | 1.25 | (vi) | *#† |
| 1380701_at | Intron: Ssfa2 | **Sequences in introns** | 255 | 1.83 | 1.23 | (vi) | * |
| 1378447_at | Intron: Thrap1 | **Sequences in introns** | 176 | 3.04 | 1.20 | (vi) | #† |
| 1395350_at | Intron: Tpm1 | **Sequences in introns** | 234 | 1.59 | 1.27 | (vi) | *#† |
| 1371091_at | Irs2 | **Adaptors/scaffold proteins** | 959 | 2.29 | 0.67 | (vi) | *#† |
| 1385649_at | Itga5 | **Cell-cell adhesion/interaction** | 1302 | 1.63 | 1.11 | (vi) | #† |
| 1369788_s_at, 1374404_at, 1389528_s_at | Jun | **Transcriptional regulation** | 919, 449, 1432 | 2.69 | 1.29 | (vi) | #† |
| 1387788_at | Junb | **Transcriptional regulation** | 1731 | 1.77 | 0.91 | (vi) | #† |
| 1387260_at | Klf4 | **Transcriptional regulation** | 1242 | 4.14 | 1.40 | (vi) | #† |
| 1368363_at, 1394039_at | Klf5 | **Transcriptional regulation** | 325, 391 | 3.24 | 0.89 | (vi) | #† |
| 1387060_at, 1388986_at, 1395557_at | Klf6 | **Transcriptional regulation** | 959, 1895, 755 | 4.30 | 0.80 | (vi) | #† |
| 1376632_at | Lmcd1 | **Transcriptional regulation** | 1212 | 3.39 | 1.02 | (vi) | #† |
| 1377610_at | Lmod2 | **Actin structures** | 2429 | 2.78 | 1.00 | (vi) | #† |
| 1380229_at | Maff | **Transcriptional regulation** | 235 | 2.36 | 0.84 | (vi) | #† |
| 1372211_at | Mafk | **Transcriptional regulation** | 967 | 1.87 | 0.85 | (vi) | *#† |
| 1387570_at | Manea | **Hydrolase** | 308 | 1.54 | 1.12 | (vi) | #† |
| 1388858_at | Map2k3 | **Kinases/kinase regulators** | 2245 | 1.62 | 1.14 | (vi) | *#† |
| 1371350_at, 1387737_at | Mat2a | **Amino acid metabolism** | 3333, 822 | 2.12 | 1.40 | (vi) | *#† |
| 1370141_at, 1372520_at | Mcl1 | **Apoptosis** | 2383, 5372 | 1.52 | 1.02 | (vi) | #† |
| 1383288_at, 1383485_at, 1384427_at | Mdm2 | **Ubiquitin cycle** | 1737, 361, 743 | 1.81 | 1.06 | (vi) | #† |
| 1394527_at | Mirn132 | **Non-protein-coding** | 136 | 1.87 | 1.37 | (vi) | * |
| 1374290_at, 1396435_at | Mirn145 | **Non-protein coding** | 286, 178 | 1.85 | 1.11 | (vi) | #† |
| 1371692_at | Mllt11 | **Unknown function** | 1400 | 1.86 | 1.29 | (vi) | * |
| 1372904_at, 1384900_at | Mobkl2b | **Kinases/kinase regulators** | 688, 255 | 1.94 | 1.18 | (vi) | #† |
| 1372808_at | Mthfd2 | **Tetrahydrofolate metabolism** | 664 | 1.51 | 1.43 | (vi) | * |
| 1368308_at | Myc | **Transcriptional regulation** | 634 | 4.75 | 1.02 | (vi) | #† |
| 1370174_at | Myd116 | **Unknown function** | 1132 | 2.41 | 0.96 | (vi) | #† |
| 1371595_at, 1397164_at | Neat1 | **Non-protein coding** | 1756, 662 | 2.42 | 0.89 | (vi) | #† |
| 1368488_at | Nfil3 | **Transcriptional regulation** | 559 | 3.27 | 1.16 | (vi) | #† |
| 1378032_at | Nfkbiz | **Transcriptional regulation** | 893 | 3.88 | 0.85 | (vi) | #† |
| 1373232_at | Nid2 | **Cell-cell adhesion/interaction** | 474 | 1.55 | 1.02 | (vi) | #† |
| 1368683_at | Oldlr1 | **Receptors** | 1316 | 1.85 | 1.12 | (vi) | #† |
| 1389918_at | Palld | **Actin structures** | 1084 | 1.51 | 0.88 | (vi) | #† |
| 1383224_at | Pard6b | **Cell-cell adhesion/interaction** | 276 | 4.07 | 1.34 | (vi) | *#† |
| 1390814_at, 1399081_at | Peli1 | **Unknown function** | 741, 522 | 1.69 | 1.04 | (vi) | #† |
| 1368303_at | Per2 | **Unknown function** | 361 | 1.80 | 1.29 | (vi) | #† |
| 1387269_s_at | Plaur | **Receptors** | 372 | 3.63 | 1.36 | (vi) | *#† |
| 1368106_at | Plk2 | **Kinases/kinase regulators** | 2643 | 3.78 | 1.14 | (vi) | #† |
| 1382642_at | Psmc6 | **Proteolysis** | 900 | 2.43 | 1.20 | (vi) | #† |
| 1370193_at | Ptp4a1 | **Phosphatases** | 3509 | 1.58 | 1.05 | (vi) | #† |
| 1370177_at | PVR | **Receptors** | 964 | 2.60 | 1.23 | (vi) | #† |
| 1383322_at | Rasl11b | **G proteins** | 1116 | 2.41 | 1.22 | (vi) | #† |
| 1373989_at | Rassf1 | **G proteins** | 537 | 1.57 | 1.35 | (vi) | * |
| 1388686_at | Rcan1 | **Phosphatases** | 5992 | 1.59 | 1.05 | (vi) | #† |
| 1388945_at | RGD1311307 | **Hypothetical proteins** | 511 | 2.03 | 1.09 | (vi) | #† |
| 1383001_at, 1383874_at, 1394278_at | RGD1560812 | **Hypothetical proteins** | 252. 397. 251 | 3.78 | 1.24 | (vi) | #† |
| 1381923_at | RGD1564664 | **Hypothetical proteins** | 277 | 1.69 | 1.02 | (vi) | #† |
| 1376937_at | RGD1565927 | **Hypothetical proteins** | 1035 | 1.78 | 1.07 | (vi) | #† |
| 1369958_at | Rhob | **G proteins** | 2376 | 3.18 | 1.50 | (vi) | *#† |
| 1381279_at | Ripk2 | **Kinases/kinase regulators** | 773 | 2.66 | 1.21 | (vi) | *#† |
| 1381533_at | Rnd1 | **G proteins** | 1026 | 3.77 | 1.27 | (vi) | #† |
| 1376066_at, 1377663_at, 1394077_at | Rnd3 | **G proteins** | 1203, 1483 | 2.12 | 1.20 | (vi) | #† |
| 1390364_at | Runx1 | **Transcriptional regulation** | 304 | 1.61 | 1.30 | (vi) | *# |
| 1371774_at | Sat | **Polyamine metabolism** | 3725 | 2.05 | 0.96 | (vi) | #† |
| 1372417_at | Sertad1 | **Transcriptional regulation** | 870 | 1.97 | 1.27 | (vi) | *#† |
| 1367802_at | Sgk | **Kinases/kinase regulators** | 2220 | 2.20 | 1.02 | (vi) | #† |
| 1372347_at | Skil | **Transcriptional regulation** | 2699 | 1.55 | 1.28 | (vi) | *#† |
| 1370314_at | Slc20a1 | **Phosphate transport** | 2847 | 1.58 | 1.19 | (vi) | *#† |
| 1368596_at | Snf1lk | **Kinases/kinase regulators** | 567 | 2.24 | 1.15 | (vi) | #† |
| 1383210_at | Sox11 | **Transcriptional regulation** | 264 | 1.51 | 1.35 | (vi) | *# |
| 1374864_at | Spry2 | **Kinases/kinase regulators** | 1551 | 2.40 | 1.08 | (vi) | #† |
| 1374829_at, 1388842_at, 1395378_at | Srf | **Transcriptional regulation** | 393, 2603, 126 | 2.31 | 1.21 | (vi) | #† |
| 1377404_at, 1386530_at, 1396101_at | Stc1 | **Agonists** | 1161, 627, 656 | 1.86 | 0.99 | (vi) | #† |
| 1367570_at | Tagln | **Actin structures** | 2778 | 1.69 | 1.11 | (vi) | #† |
| 1371554_at | Tcap | **Myofilaments** | 1229 | 1.59 | 1.09 | (vi) | * |
| 1377340_at | Tfpi2 | **Proteolysis** | 270 | 2.10 | 1.20 | (vi) | #† |
| 1367859_at | Tgfb3 | **Agonists** | 927 | 1.83 | 0.99 | (vi) | #† |
| 1375951_at | Thbd | **Receptors** | 242 | 1.91 | 1.05 | (vi) | #† |
| 1374529_at, 1394109_at | Thbs1 | **Cell-cell adhesion/interaction** | 10105, 5478 | 2.52 | 1.14 | (vi) | #† |
| 1372926_at, 1389836_a_at | Timp3 | **Proteolysis** | 590, 2343 | 1.74 | 1.06 | (vi) | #† |
| 1374446_at, 1385407_at | Tiparp | **Glycosylation** | 2600, 1629 | 1.55 | 1.16 | (vi) | #† |
| 1373401_at | Tnc | **Cell-cell adhesion/interaction** | 1073 | 1.83 | 0.87 | (vi) | #† |
| 1385641_at | Tnfaip3 | **Transcriptional regulation** | 1101 | 2.35 | 0.73 | (vi) | *#† |
| 1371194_at | Tnfaip6 | **Cell-cell adhesion/interaction** | 458 | 4.96 | 1.19 | (vi) | #† |
| 1369407_at | Tnfrsf11b | **Receptors** | 1400 | 2.02 | 1.18 | (vi) | #† |
| 1371785_at | Tnfrsf12a | **Receptors** | 3932 | 2.43 | 1.32 | (vi) | *#† |
| 1370287_a_at | Tpm1 | **Myofilaments** | 2268 | 1.51 | 1.12 | (vi) | #† |
| 1371019_at, 1391643_at | Trib1 | **Kinases/kinase regulators** | 385, 1036 | 2.94 | 1.06 | (vi) | #† |
| 1378165_at, 1387750_at | Twist1 | **Transcriptional regulation** | 456, 99 | 4.54 | 1.31 | (vi) | #† |
| 1379910_at | Uap1 | **Glycosaminoglycan metabolism** | 932 | 2.73 | 1.28 | (vi) | #† |
| 1373651_at | Unknown | **No established gene** | 388 | 1.80 | 1.15 | (vi) | * |
| 1392108_at | Unknown | **No established gene** | 94 | 2.75 | 0.82 | (vi) | #† |
| 1378754_at | Unknown | **No established gene** | 359 | 2.66 | 0.97 | (vi) | #† |
| 1373679_at | Unknown | **No established gene** | 664 | 1.96 | 0.98 | (vi) | #† |
| 1376403_at | Unknown | **No established gene** | 193 | 2.17 | 1.07 | (vi) | #† |
| 1395119_at | Unknown | **No established gene** | 221 | 2.06 | 1.08 | (vi) | #† |
| 1379584_at | Unknown | **No established gene** | 215 | 2.08 | 1.19 | (vi) | #† |
| 1381341_at | Unknown | **No established gene** | 180 | 4.93 | 1.21 | (vi) | #† |
| 1379714_at | Unknown | **No established gene** | 134 | 2.44 | 1.24 | (vi) | #† |
| 1372905_at, 1398476_at | Vcl | **Actin structures** | 4753, 885 | 1.70 | 1.14 | (vi) | #† |
| 1377778_at | Vof16 | **Unknown function** | 327 | 2.92 | 1.05 | (vi) | #† |
| 1373767_at | Zfand2a | **Unknown function** | 636 | 1.52 | 1.08 | (vi) | #† |
| 1388868_at | Zfand5 | **Unknown function** | 4425 | 1.80 | 0.92 | (vi) | *#† |
| 1385869_at | Zfp281 | **Transcriptional regulation** | 977 | 2.45 | 1.07 | (vi) | #† |
| 1387870_at | Zfp36 | **mRNA stability** | 1255 | 3.35 | 1.25 | (vi) | *#† |
| 1373106_at | Zfp36l2 | **mRNA stability** | 2154 | 1.61 | 0.98 | (vi) | #† |
| 1398536_at | Zfp655 | **Transcriptional regulation** | 281 | 1.74 | 1.07 | (vi) | #† |
| 1373680_at | Zfp697 | **Transcriptional regulation** | 426 | 2.26 | 1.26 | (vi) | #† |
| 1388130_at | Zyx | **Actin structures** | 4942 | 1.73 | 1.19 | (vi) | *#† |
| 1387068_at | Arc | **Unknown function** | 256 | 5.14 | 0.98 | (vii) | #† |
| 1369871_at | Areg | **Agonists** | 73 | 11.55 | 0.78 | (vii) | #† |
| 1386994_at, 1386995_at | Btg2 | **Transcriptional regulation** | 1101, 1127 | 7.67 | 1.25 | (vii) | #† |
| 1377869_at | Ccrn4l | **Transcriptional regulation** | 455 | 11.01 | 1.20 | (vii) | #† |
| 1380063_at | Ch25h | **Lipid metabolism** | 490 | 9.83 | 1.12 | (vii) | #† |
| 1367601_at, 1367602_at | Cited2 | **Transcriptional regulation** | 1314, 2091 | 5.03 | 0.75 | (vii) | #† |
| 1368290_at | Cyr61 | **Cell-cell adhesion/interaction** | 506 | 7.46 | 1.07 | (vii) | #† |
| 1368321_at | Egr1 | **Transcriptional regulation** | 3193 | 5.49 | 1.20 | (vii) | #† |
| 1387306_a_at, 1398266_a_at | Egr2 | **Transcriptional regulation** | 464, 107 | 6.69 | 1.33 | (vii) | #† |
| 1387442_at | Egr4 | **Transcriptional regulation** | 9 | 68.89 | 0.82 | (vii) | #† |
| 1373093_at | Errfi1 | **Kinases/kinase regulators** | 1963 | 5.23 | 1.12 | (vii) | #† |
| 1375043_at | Fos | **Transcriptional regulation** | 241 | 12.50 | 1.22 | (vii) | #† |
| 1373759_at | FosB | **Transcriptional regulation** | 41 | 113.08 | 0.70 | (vii) | #† |
| 1370454_at, 1370997_at | Homer1 | **Receptors** | 210, 34 | 5.32 | 1.02 | (vii) | #† |
| 1389355_at, 1389882_at | Ier5 | **Unknown function** | 2068, 99 | 5.07 | 1.21 | (vii) | #† |
| 1369191_at | Il6 | **Agonists** | 131 | 10.48 | 1.06 | (vii) | #† |
| 1369012_at, 1383486_at | Inhba | **Agonists** | 223, 290 | 5.34 | 1.14 | (vii) | #† |
| 1396539_at | Intron: Actn1 | **Sequences in introns** | 230 | 6.19 | 1.18 | (vii) | #† |
| 1383828_at | Intron: Klf14 | **Sequences in introns** | 76 | 5.11 | 1.23 | (vii) | #† |
| 1398691_at | Intron: TSC22d2 | **Sequences in introns** | 58 | 5.17 | 1.27 | (vii) | #† |
| 1378867_at | Mirn221 | **Non-protein coding** | 80 | 8.09 | 1.04 | (vii) | #† |
| 1386935_at | Nr4a1 | **Transcriptional regulation** | 635 | 18.48 | 1.08 | (vii) | #† |
| 1369007_at | Nr4a2 | **Transcriptional regulation** | 95 | 5.96 | 0.96 | (vii) | #† |
| 1369067_at, 1393389_at | Nr4a3 | **Transcriptional regulation** | 85, 136 | 30.72 | 1.31 | (vii) | #† |
| 1384254_at | Otud1 | **Unknown function** | 1602 | 6.08 | 0.93 | (vii) | #† |
| 1368527_at | Ptgs2 | **Lipid metabolism** | 427 | 14.36 | 1.13 | (vii) | #† |
| 1368144_at. 1387074_at | Rgs2 | **G proteins** | 1175. 1977 | 5.66 | 1.34 | (vii) | *#† |
| 1368487_at | Serpinb2 | **Proteolysis** | 354 | 17.02 | 1.16 | (vii) | #† |
| 1372510_at, 1384331_at | Srxn1 | **Redox/detoxification** | 615, 328 | 5.47 | 1.36 | (vii) | *#† |
| 1391791_at | Acer2 | **Lipid metabolism** | 285 | 0.64 | 0.95 | (viii) | * |
| 1390931_at | Adamts15 | **Proteolysis** | 898 | 0.55 | 1.08 | (viii) | #† |
| 1383848_at | Adrb1 | **Receptors** | 718 | 0.40 | 1.39 | (viii) | #† |
| 1367655_at | Ankrd1 | **Transcriptional regulation** | 9018 | 0.63 | 0.68 | (viii) | * |
| 1373302_at | Asah3l | **Lipid metabolism** | 455 | 0.66 | 0.75 | (viii) | *# |
| 1373287_at | Atoh8 | **Transcriptional regulation** | 935 | 0.39 | 1.01 | (viii) | #† |
| 1370823_at | Bambi | **Receptors** | 1061 | 0.52 | 1.02 | (viii) | #† |
| 1388742_at | Bcl2l11 | **Apoptosis** | 1016 | 0.63 | 0.74 | (viii) | *# |
| 1385627_at, 1398482_at | Bcl3 | **Transcriptional regulation** | 499, 455 | 0.48 | 1.27 | (viii) | #† |
| 1374493_at | Bmf | **Apoptosis** | 466 | 0.62 | 0.81 | (viii) | *#† |
| 1373885_at | Cbx5 | **Transcriptional regulation** | 354 | 0.62 | 0.75 | (viii) | * |
| 1368813_at, 1387343_at | Cebpd | **Transcriptional regulation** | 981, 2512 | 0.46 | 0.78 | (viii) | #† |
| 1374416_at | Chchd8 | **Unknown function** | 453 | 0.63 | 0.89 | (viii) | #† |
| 1398710_at | Cyp2u1 | **Redox/detoxification** | 403 | 0.57 | 0.87 | (viii) | *#† |
| 1368025_at | Ddit4 | **Apoptosis** | 2986 | 0.16 | 0.94 | (viii) | #† |
| 1391741_a_at | Fam78a | **Hypothetical proteins** | 344 | 0.58 | 1.26 | (viii) | * |
| 1372086_at | Fhdc1 | **Actin structures** | 355 | 0.53 | 0.81 | (viii) | #† |
| 1382774_at | Frat1 | **Other signalling** | 587 | 0.60 | 1.18 | (viii) | #† |
| 1373838_at | Fut4 | **Carbohydrate metabolism** | 640 | 0.65 | 1.03 | (viii) | #† |
| 1386818_at | Golph3l | **Protein transport** | 250 | 0.65 | 0.82 | (viii) | * |
| 1387036_at | Hes1 | **Transcriptional regulation** | 1854 | 0.48 | 1.14 | (viii) | #† |
| 1387270_at | Hhex | **Transcriptional regulation** | 315 | 0.65 | 0.78 | (viii) | * |
| 1389675_at, 1391026_at | Ier5l | **Unknown function** | 637, 1234 | 0.34 | 1.43 | (viii) | #† |
| 1378945_at | Intron: Aco2 | **Sequences in introns** | 199 | 0.58 | 1.40 | (viii) | #† |
| 1376522_at | Intron: Fabp3 | **Sequences in introns** | 302 | 0.38 | 1.02 | (viii) | #† |
| 1368073_at | Irf1 | **Transcriptional regulation** | 920 | 0.37 | 0.92 | (viii) | #† |
| 1374627_at | Irf9 | **Transcriptional regulation** | 430 | 0.64 | 0.88 | (viii) | #† |
| 1390776_at | Irx3 | **Transcriptional regulation** | 1685 | 0.56 | 1.24 | (viii) | #† |
| 1386823_at | Irx5 | **Transcriptional regulation** | 356 | 0.54 | 1.30 | (viii) | #† |
| 1390969_at | Kcne4 | **Potassium transport** | 1056 | 0.51 | 0.85 | (viii) | #† |
| 1387698_at, 1391007_s_at | Kcnj11 | **Potassium transport** | 377, 545 | 0.61 | 0.99 | (viii) | #† |
| 1389988_at | Kctd2 | **Potassium transport** | 1013 | 0.61 | 0.99 | (viii) | #† |
| 1379914_at | Klf11 | **Transcriptional regulation** | 522 | 0.59 | 0.87 | (viii) | #† |
| 1389479_at | Klf3 | **Transcriptional regulation** | 1924 | 0.61 | 1.04 | (viii) | #† |
| 1380449_at | Klhdc5 | **Unknown function** | 331 | 0.63 | 1.00 | (viii) | #† |
| 1383169_at | Lifr | **Receptors** | 1870 | 0.67 | 0.86 | (viii) | * |
| 1392057_at | LOC500893 | **Hypothetical proteins** | 431 | 0.64 | 0.87 | (viii) | #† |
| 1375988_at | LOC680262 | **Hypothetical proteins** | 261 | 0.63 | 0.88 | (viii) | #† |
| 1390907_at | LOC691170 | **Hypothetical proteins** | 392 | 0.65 | 0.73 | (viii) | *# |
| 1385299_at | Lpar4 | **Receptors** | 283 | 0.63 | 0.79 | (viii) | * |
| 1372060_at | Lysmd4 | **Unknown function** | 467 | 0.52 | 0.74 | (viii) | * |
| 1398804_at | Mak10 | **Unknown function** | 680 | 0.61 | 0.85 | (viii) | #† |
| 1368871_at | Map3k1 | **Kinases/kinase regulators** | 1127 | 0.60 | 0.78 | (viii) | *#† |
| 1376718_at | Mblac1 | **Unknown function** | 458 | 0.62 | 1.02 | (viii) | #† |
| 1379059_at | Micall2 | **Unknown function** | 367 | 0.64 | 0.93 | (viii) | #† |
| 1368376_at | Nr0b2 | **Transcriptional regulation** | 311 | 0.53 | 0.81 | (viii) | * |
| 1377955_at | Orai2 | **Unknown function** | 340 | 0.63 | 0.95 | (viii) | #† |
| 1374693_at | Parp16 | **Glycosylation** | 413 | 0.48 | 0.79 | (viii) | *#† |
| 1378143_at | Pdcl | **Unknown function** | 312 | 0.66 | 0.89 | (viii) | #† |
| 1394014_at | Phf12 | **Transcriptional regulation** | 360 | 0.66 | 1.17 | (viii) | #† |
| 1369655_at | Pik3c3 | **Kinases/kinase regulators** | 438 | 0.62 | 0.79 | (viii) | * |
| 1388525_at | Pik3ip1 | **Kinases/kinase regulators** | 1160 | 0.55 | 0.67 | (viii) | *# |
| 1373532_at | Plekhf1 | **Apoptosis** | 1082 | 0.61 | 0.78 | (viii) | *#† |
| 1377384_at | Plekhh3 | **Unknown function** | 237 | 0.53 | 1.15 | (viii) | #† |
| 1384262_at | Ppp1r3b | **Phosphatases** | 914 | 0.31 | 0.84 | (viii) | #† |
| 1395236_at | Ppp1r3c | **Phosphatases** | 389 | 0.65 | 0.83 | (viii) | #† |
| 1393231_at | Ppp4r2 | **Phosphatases** | 265 | 0.64 | 0.85 | (viii) | * |
| 1398508_at | Pqcp | **Unknown function** | 2082 | 0.64 | 1.01 | (viii) | #† |
| 1392593_a_at | Prr12 | **Unknown function** | 286 | 0.64 | 1.09 | (viii) | #† |
| 1378484_at | Rasl12 | **G proteins** | 442 | 0.61 | 1.00 | (viii) | #† |
| 1377187_at | Rbm12b | **RNA binding** | 294 | 0.61 | 0.90 | (viii) | #† |
| 1377793_at | Rbm33 | **RNA binding** | 324 | 0.65 | 0.91 | (viii) | #† |
| 1383641_at | RGD1559432 | **Hypothetical proteins** | 1887 | 0.64 | 0.82 | (viii) | #† |
| 1392588_at | Ripk5 | **Kinases/kinase regulators** | 614 | 0.66 | 0.75 | (viii) | *# |
| 1389790_at | Rnf169 | **Unknown function** | 465 | 0.66 | 0.89 | (viii) | #† |
| 1393193_at | Rpusd2 | **RNA binding** | 336 | 0.65 | 1.28 | (viii) | #† |
| 1389437_at | Sall2 | **Transcriptional regulation** | 423 | 0.50 | 1.05 | (viii) | #† |
| 1390695_at | Scyl3 | **Kinases/kinase regulators** | 277 | 0.64 | 1.10 | (viii) | #† |
| 1390249_at | Sept14 | **Cell cycle** | 560 | 0.56 | 1.27 | (viii) | *#† |
| 1372248_at | Sesn1 | **Unknown function** | 564 | 0.54 | 0.90 | (viii) | #† |
| 1394025_at | Smad6 | **Transcriptional regulation** | 1033 | 0.53 | 1.13 | (viii) | #† |
| 1368896_at | Smad7 | **Transcriptional regulation** | 845 | 0.59 | 1.08 | (viii) | #† |
| 1393262_at | Smad9 | **Transcriptional regulation** | 555 | 0.64 | 1.02 | (viii) | #† |
| 1373219_at | Snai1 | **Transcriptional regulation** | 1250 | 0.28 | 1.49 | (viii) | *#† |
| 1383589_at | Snai2 | **Transcriptional regulation** | 500 | 0.60 | 1.24 | (viii) | † |
| 1389460_at | Socs6 | **Kinases/kinase regulators** | 463 | 0.58 | 0.77 | (viii) | *#† |
| 1377458_at | Sorl1 | **Lipid transport** | 752 | 0.58 | 0.80 | (viii) | * |
| 1372633_at | Spg20 | **Microtubules** | 718 | 0.60 | 0.93 | (viii) | #† |
| 1388786_at | Synpo | **Actin structures** | 2541 | 0.58 | 1.02 | (viii) | #† |
| 1393153_at | Tardbp | **mRNA splicing** | 298 | 0.58 | 1.22 | (viii) | *#† |
| 1391630_at | Tbx18 | **Transcriptional regulation** | 460 | 0.57 | 0.88 | (viii) | #† |
| 1383282_at | Thap11 | **DNA structure and repair** | 584 | 0.64 | 0.98 | (viii) | #† |
| 1390832_at | Tmcc3 | **Unknown function** | 984 | 0.60 | 0.99 | (viii) | #† |
| 1372935_at | Tmem119 | **Unknown function** | 471 | 0.66 | 1.19 | (viii) | #† |
| 1378592_at | Trim59 | **Unknown function** | 337 | 0.57 | 0.93 | (viii) | #† |
| 1391027_at | Trim65 | **Unknown function** | 297 | 0.58 | 1.01 | (viii) | #† |
| 1371131_a_at | Txnip | **Transcriptional regulation** | 5221 | 0.56 | 0.86 | (viii) | #† |
| 1384135_at | Unknown | **No established gene** | 446 | 0.63 | 0.78 | (viii) | * |
| 1376617_at | Unknown | **No established gene** | 722 | 0.62 | 0.81 | (viii) | * |
| 1381228_at | Unknown | **No established gene** | 279 | 0.64 | 0.84 | (viii) | * |
| 1398366_at | Unknown | **No established gene** | 422 | 0.64 | 0.90 | (viii) | #† |
| 1383058_at | Unknown | **No established gene** | 2326 | 0.55 | 0.92 | (viii) | #† |
| 1375698_at | Unknown | **No established gene** | 432 | 0.58 | 0.95 | (viii) | #† |
| 1376376_at | Unknown | **No established gene** | 415 | 0.62 | 0.98 | (viii) | #† |
| 1392842_at | Unknown | **No established gene** | 542 | 0.64 | 1.02 | (viii) | #† |
| 1389419_at | Unknown | **No established gene** | 504 | 0.54 | 1.06 | (viii) | #† |
| 1374243_at | Unknown | **No established gene** | 269 | 0.66 | 1.08 | (viii) | #† |
| 1382228_at | Unknown | **No established gene** | 495 | 0.55 | 1.20 | (viii) | #† |
| 1391863_at | Unknown | **No established gene** | 255 | 0.32 | 1.36 | (viii) | #† |
| 1386633_at | Unknown | **No established gene** | 299 | 0.62 | 0.75 | (viii) | *#† |
| 1384401_at | Unknown | **No established gene** | 522 | 0.63 | 0.82 | (viii) | *#† |
| 1368641_at | Wnt4 | **Agonists** | 450 | 0.65 | 0.91 | (viii) | #† |
| 1380914_at, 1393652_at | Zbtb1 | **Transcriptional regulation** | 271, 582 | 0.44 | 0.99 | (viii) | #† |
| 1377154_at | Zfp157 | **Unknown function** | 250 | 0.50 | 1.01 | (viii) | #† |
| 1393120_at | Zfp251 | **Transcriptional regulation** | 579 | 0.65 | 0.87 | (viii) | * |
| 1372205_at | Zfp278 | **Transcriptional regulation** | 483 | 0.59 | 0.97 | (viii) | #† |
| 1381902_at, 1399119_at | Zfp292 | **Transcriptional regulation** | 487, 366 | 0.63 | 0.84 | (viii) | #† |
| 1368877_at | Zfp354a | **Transcriptional regulation** | 372 | 0.56 | 1.01 | (viii) | #† |
| 1381065_at | Zfp383 | **Transcriptional regulation** | 808 | 0.60 | 0.97 | (viii) | #† |
| 1370984_at | Zfp46 | **Transcriptional regulation** | 735 | 0.53 | 0.86 | (viii) | #† |
| 1391507_at | Zfp467 | **Transcriptional regulation** | 378 | 0.51 | 0.89 | (viii) | #† |
| 1389366_at | Zfp553 | **Transcriptional regulation** | 465 | 0.54 | 1.00 | (viii) | #† |
| 1382362_at | Zfp87 | **Unknown function** | 376 | 0.60 | 0.88 | (viii) | #† |
| 1380186_at | Zmym5 | **Unknown function** | 366 | 0.58 | 1.07 | (viii) | #† |
| 1376917_at | Znf292 | **Transcriptional regulation** | 364 | 0.58 | 0.79 | (viii) | * |
