# Supplementary material for: Regulation of the cardiomyocyte transcriptome vs translatome by endothelin-1 and insulin: translational regulation of 5' terminal oligopyrimidine tract (TOP) mRNAs by insulin
Source: BMC Genomics. 2010 May 29;11:343. doi: 10.1186/1471-2164-11-343 (PMC2900265; doi:10.1186/1471-2164-11-343)
Supplement: Additional file 2 — Functional classifications of the top 10th percentile RNAs identified in neonatal rat cardiomyocytes (Microsoft Word Table). Total and polysomal RNA from unstimulated neonatal rat cardiomyocytes (serum-starved, 20 h) were analysed using microarrays. Results are the mean raw fluorescence values (n = 4). For transcripts represented by more than one probeset, the probesets and mean corresponding raw values are listed. Differential expression in polysome vs total RNA was analysed by t-test with Benjamini-Hochberg FDR correction (*p < 0.05). RNAs are grouped according to general functional categories (summarised in Figure 3C of the associated manuscript) with detailed functions within the Table. [file 1471-2164-11-343-S2.DOC]

**Additional file 2. Functional classifications of the top 10th percentile RNAs identified in neonatal rat cardiomyocytes.** Total and polysomal RNA from unstimulated neonatal rat cardiomyocytes (serum-starved, 20 h) were analysed using microarrays. Results are the mean raw fluorescence values (n=4). For transcripts represented by more than one probeset, the probesets and mean corresponding raw values are listed. Differential expression in polysome *vs* total RNA was analysed by t-test with Benjamini-Hochberg FDR correction (*p<0.05). RNAs are grouped according to general functional categories (summarised in Figure 3C of the associated manuscript) with detailed functions within the Table.

| **Probeset** | **Gene Symbol** | **Function** | **Polysomal RNA** | **Total RNA** |  |
| --- | --- | --- | --- | --- | --- |
| **Agonists and receptors** | | | --- | --- |  |
| 1398345_at | Angptl2 | Agonists | 3179 | 3183 |  |
| 1389546_at | Amotl2 | Agonists | 2925 | 4116 | * |
| 1372352_at | Armet | Agonists | 4266 | 3038 | * |
| 1367973_at | Ccl2 | Agonists | 12079 | 13578 |  |
| 1379935_at | Ccl7 | Agonists | 4178 | 2730 | * |
| 1372042_at | Cmtm3 | Agonists | 3626 | 2406 | * |
| 1367631_at | Ctgf | Agonists | 10354 | 12903 |  |
| 1387316_at | Cxcl1 | Agonists | 4345 | 3361 |  |
| 1372064_at | Cxcl16 | Agonists | 4253 | 2900 | * |
| 1370328_at | Dkk3 | Agonists | 2492 | 3075 | * |
| 1386893_at | Grn | Agonists | 7952 | 10752 | * |
| 1370333_a_at, 1388469_at, 1392569_at | Igf1 | Agonists | 6771, 5137, 5301 | 3957, 4729, 3586 | * |
| 1386881_at | Igfbp3 | Agonists | 6030 | 4480 |  |
| 1371357_at | Igfbp7 | Agonists | 11814 | 11491 |  |
| 1367682_at | Mdk | Agonists | 3713 | 2472 | * |
| 1367609_at | Mif | Agonists | 9463 | 6491 | * |
| 1371841_at | Mtpn | Agonists | 6172 | 5009 | * |
| 1367564_at | Nppa | Agonists | 21411 | 25501 |  |
| 1367616_at | Nppb | Agonists | 16458 | 18425 |  |
| 1398810_at | Pdap1 | Agonists | 7561 | 4264 | * |
| 1367949_at | Penk1 | Agonists | 6361 | 6117 |  |
| 1371250_at | Pf4 | Agonists | 5516 | 3083 | * |
| 1369968_at | Ptn | Agonists | 4833 | 3911 |  |
| 1390119_at | Sfrp2 | Agonists | 3794 | 3106 |  |
| 1373807_at | Vegfa | Agonists | 3205 | 5969 | * |
| 1380854_at | Vegfb | Agonists | 3537 | 1838 | * |
| 1388373_at | Adipor1 | Receptors | 4784 | 4512 |  |
| 1371328_at | Amfr | Receptors | 5501 | 5110 |  |
| 1370891_at | Cd48 | Receptors | 3488 | 4523 | * |
| 1367929_at | Cd59 | Receptors | 4756 | 4853 |  |
| 1367850_at, 1398246_s_at | Fcgr2a | Receptors | 5595, 5768 | 3813, 4173 | * |
| 1370256_at | Fzd1 | Receptors | 5052 | 6645 | * |
| 1374816_at | Gcom1 | Receptors | 2392 | 3543 | * |
| 1372724_at | Grina | Receptors | 6262 | 4162 | * |
| 1398889_at | Grinl1a | Receptors | 6996 | 5745 | * |
| 1369956_at | Ifngr1 | Receptors | 2893 | 3174 |  |
| 1367636_at, 1386872_at | Igf2r | Receptors | 1536, 3063 | 4542, 6881 | * |
| 1392946_at | Il1r1 | Receptors | 2561 | 3374 | * |
| 1370957_at | Il6st | Receptors | 3944 | 4153 |  |
| 1376089_at | Ldlr | Receptors | 1453 | 3814 | * |
| 1386878_at | Lrp10 | Receptors | 3465 | 3394 |  |
| 1372914_at | Ltbr | Receptors | 4078 | 2551 | * |
| 1371495_at | M6pr | Receptors | 5157 | 4354 | * |
| 1372029_at | M6prbp1 | Receptors | 3190 | 2774 |  |
| 1388588_at | Mtvr2 | Receptors | 3254 | 2001 | * |
| 1388549_at | Ncoa4 | Receptors | 2052 | 3470 | * |
| 1369948_at | Ngfrap1 | Receptors | 3659 | 1978 | * |
| 1371558_at | Nisch | Receptors | 2821 | 4800 | * |
| 1370570_at, 1373577_at | Nrp1 | Receptors | 2638, 1036 | 4382, 3044 | * |
| 1391106_at | Osmr | Receptors | 2909 | 6562 | * |
| 1370941_at | Pdgfra | Receptors | 2980 | 4289 |  |
| 1367619_at | Pgrmc1 | Receptors | 3596 | 3170 |  |
| 1388531_at | Pgrmc2 | Receptors | 4839 | 3017 | * |
| 1371411_at | Plxnb2 | Receptors | 2534 | 3633 | * |
| 1371840_at | S1pr1 | Receptors | 3750 | 4796 | * |
| 1371506_at | Scarb2 | Receptors | 1845 | 3218 | * |
| 1388750_at | Tfrc | Receptors | 2061 | 6870 | * |
| 1372466_at | Tgfbr2 | Receptors | 5881 | 5596 |  |
| 1373092_at | Tgfbr3 | Receptors | 1739 | 3473 | * |
| 1371785_at | Tnfrsf12a | Receptors | 6412 | 3932 |  |
| 1367715_at | Tnfrsf1a | Receptors | 6051 | 4232 |  |
| --- | --- | --- | --- | --- |  |
| **Cell-cell/Cell-matrix adhesion//Extracellular matrix** | | | --- | --- |  |
| 1371703_at | Ahnak | Cell-cell adhesion/interaction | 2235 | 7066 | * |
| 1386886_at | Cd164 | Cell-cell adhesion/interaction | 7446 | 8008 |  |
| 1371499_at | Cd9 | Cell-cell adhesion/interaction | 3341 | 3486 |  |
| 1368642_at, 1387259_at | Cdh2 | Cell-cell adhesion/interaction | 2969, 2071 | 5823, 4638 | * |
| 1373067_at, 1373470_at | Ctnnb1 | Cell-cell adhesion/interaction | 9339, 7178 | 13831, 11173 | * |
| 1398600_at | Dcbld2 | Cell-cell adhesion/interaction | 5373 | 6639 |  |
| 1367654_at | Fat1 | Cell-cell adhesion/interaction | 1432 | 3585 | * |
| 1367746_a_at | Flot2 | Cell-cell adhesion/interaction | 4536 | 3684 | * |
| 1372002_at | Gja1 | Cell-cell adhesion/interaction | 12980 | 19135 | * |
| 1368819_at, 1387346_at | Itgb1 | Cell-cell adhesion/interaction | 6736 | 10517 | * |
| 1387061_at | Jup | Cell-cell adhesion/interaction | 2040 | 3076 | * |
| 1373072_at | Lims1 | Cell-cell adhesion/interaction | 4151 | 4199 |  |
| 1388422_at | Lims2 | Cell-cell adhesion/interaction | 3802 | 3444 |  |
| 1371734_at | Maea | Cell-cell adhesion/interaction | 4380 | 3549 | * |
| 1387016_a_at | Nptn | Cell-cell adhesion/interaction | 4818 | 5628 | * |
| 1388551_at | Pcdhga1/2/3/5/7/8/9/10/11/12 | Cell-cell adhesion/interaction | 3302 | 4301 | * |
| 1398975_at | Aamp | Cell-matrix adhesion | 4474 | 2190 | * |
| 1367681_at | Cd151 | Cell-matrix adhesion | 5589 | 5930 |  |
| 1367689_a_at, 1386901_at | Cd36 | Cell-matrix adhesion | 824, 3361 | 3460, 8336 | * |
| 1398827_at | Cd81 | Cell-matrix adhesion | 12483 | 13848 | * |
| 1371518_at | Nid1 | Cell-matrix adhesion | 3985 | 7377 | * |
| 1367721_at | Sdc4 | Cell-matrix adhesion | 5899 | 4134 | * |
| 1374529_at, 1394109_at | Thbs1 | Cell-matrix adhesion | 3224, 1042 | 10105, 5478 | * |
| 1371571_at, 1371572_at | App | Extracellular matrix | 7414, 2596 | 11497, 5358 | * |
| 1367594_at | Bgn | Extracellular matrix | 5385 | 6220 |  |
| 1370161_at | Ccdc80 | Extracellular matrix | 3835 | 5861 | * |
| 1376105_at | Col14a1 | Extracellular matrix | 1844 | 3676 | * |
| 1388939_at | Col15a1 | Extracellular matrix | 2107 | 4038 | * |
| 1370864_at, 1388116_at | Col1a1 | Extracellular matrix | 9601, 15552 | 12628, 20910 | * |
| 1370155_at, 1387854_at | Col1a2 | Extracellular matrix | 11235, 8288 | 17009, 13480 | * |
| 1370959_at | Col3a1 | Extracellular matrix | 13542 | 21085 | * |
| 1372439_at, 1373245_at | Col4a1 | Extracellular matrix | 4612, 4541 | 11913, 12386 | * |
| 1388494_at | Col4a2 | Extracellular matrix | 2870 | 8064 | * |
| 1376099_at | Col5a1 | Extracellular matrix | 1462 | 3166 | * |
| 1370895_at, 1373463_at | Col5a2 | Extracellular matrix | 4219, 1671 | 10545, 4638 | * |
| 1371349_at | Col6a1 | Extracellular matrix | 5091 | 8482 | * |
| 1371369_at | Col6a2 | Extracellular matrix | 3753 | 4988 | * |
| 1371847_at | Crtap | Extracellular matrix | 4480 | 2663 | * |
| 1370956_at | Dcn | Extracellular matrix | 16466 | 20220 |  |
| 1368829_at | Fbn1 | Extracellular matrix | 1431 | 4585 | * |
| 1370234_at | Fn1 | Extracellular matrix | 4364 | 14991 | * |
| 1373206_at, 1373416_at | Fndc3b | Extracellular matrix | 2613, 2940 | 4690, 3469 | * |
| 1371331_at | Fstl1 | Extracellular matrix | 5336 | 5299 |  |
| 1387039_at | Gpc1 | Extracellular matrix | 3917 | 4664 |  |
| 1368187_at | Gpnmb | Extracellular matrix | 11025 | 14389 | * |
| 1389214_at | Lama4 | Extracellular matrix | 1345 | 2895 | * |
| 1373210_at | Lamb1 | Extracellular matrix | 1723 | 4181 | * |
| 1370993_at, 1371322_at | Lamc1 | Extracellular matrix | 938, 2769 | 4323, 8078 | * |
| 1367628_at | Lgals1 | Extracellular matrix | 9667 | 7295 | * |
| 1386879_at | Lgals3 | Extracellular matrix | 10995 | 10010 |  |
| 1387946_at | Lgals3bp | Extracellular matrix | 2251 | 3707 | * |
| 1373815_at | Lman2 | Extracellular matrix | 3690 | 2933 | * |
| 1367749_at | Lum | Extracellular matrix | 10882 | 15969 | * |
| 1386860_at | Mfge8 | Extracellular matrix | 2818 | 4028 | * |
| 1367568_a_at | Mgp | Extracellular matrix | 17203 | 18165 |  |
| 1370296_at | Podn | Extracellular matrix | 5047 | 3201 | * |
| 1373911_at | Postn | Extracellular matrix | 10116 | 17361 | * |
| 1367562_at, 1367563_at | Sparc | Extracellular matrix | 12348, 6746 | 13212, 5555 |  |
| 1367581_a_at | Spp1 | Extracellular matrix | 3853 | 6038 | * |
| --- | --- | --- | --- | --- |  |
| **Channels, pumps, transporters, Ca^2+^ handling** | | | --- | --- |  |
| 1375146_at | Slc25a39 | Heme biosynthesis | 5312 | 3167 | * |
| 1388112_at | Slc25a4 | ADP transport | 14282 | 16561 | * |
| 1388163_at | Slc25a5 | ADP transport | 11517 | 14560 | * |
| 1370286_at | Slc38a2 | Amino acid transport | 2692 | 5840 | * |
| 1398771_at | Slc3a2 | Amino acid transport | 3830 | 4174 |  |
| 1367706_at, 1386909_a_at | Vdac1 | Anion channel | 7491, 2886 | 8344, 3267 |  |
| 1369933_at | Vdac2 | Anion channel | 9165 | 9529 |  |
| 1386902_at | Vdac3 | Anion channel | 10408 | 11016 |  |
| 1369936_at, 1369937_at, 1387772_at | Calm1 | Calcium binding/trafficking | 8779, 10522, 5380 | 6087, 6056, 3735 | * |
| 1370246_at | Calm2 | Calcium binding/trafficking | 10223 | 6829 | * |
| 1370873_at | Calm3 | Calcium binding/trafficking | 3577 | 1649 | * |
| 1398750_at | Calr | Calcium binding/trafficking | 7267 | 8977 |  |
| 1388217_a_at | Calu | Calcium binding/trafficking | 3734 | 4971 | * |
| 1371686_at, 1388442_at | Canx | Calcium binding/trafficking | 2834, 4091 | 5019, 7067 | * |
| 1368988_at, 1387401_at | Casq2 | Calcium binding/trafficking | 4607, 1733 | 8913, 5357 | * |
| 1393643_at | Rcn1 | Calcium binding/trafficking | 4801 | 4621 |  |
| 1387081_at | Rcn2 | Calcium binding/trafficking | 3426 | 3554 |  |
| 1386890_at | S100a10 | Calcium binding/trafficking | 7281 | 4535 | * |
| 1375170_at | S100a11 | Calcium binding/trafficking | 15346 | 12501 |  |
| 1388356_at | S100a16 | Calcium binding/trafficking | 6133 | 3302 | * |
| 1367846_at | S100a4 | Calcium binding/trafficking | 13499 | 13597 |  |
| 1367661_at | S100a6 | Calcium binding/trafficking | 12981 | 8690 | * |
| 1371746_at | Srl | Calcium binding/trafficking | 2253 | 4216 | * |
| 1369065_a_at, 1370426_a_at | Atp2a2 | Calcium binding/trafficking | 11346, 3289 | 22761, 6314 | * |
| 1370157_at, 1388876_at | Pln | Calcium binding/trafficking | 16007, 13615 | 18345, 14069 | * |
| 1382088_at | Ryr2 | Calcium binding/trafficking | 1132 | 4234 | * |
| 1383094_at | Sri | Calcium binding/trafficking | 5453 | 3328 | * |
| 1375633_at | Clic1 | Chloride channel | 8237 | 6043 | * |
| 1388759_at | Clic4 | Chloride channel | 7815 | 7435 |  |
| 1369960_at | Fxyd1 | Chloride channel | 8093 | 6911 |  |
| 1367873_at | Atp6ap1 | H+ transport (lysosomes) | 3074 | 4179 | * |
| 1398930_at | Atp6v0b | H+ transport (lysosomes) | 4147 | 3001 | * |
| 1398755_at | Atp6v0c | H+ transport (lysosomes) | 10141 | 8064 | * |
| 1388365_at | Atp6v0d1 | H+ transport (lysosomes) | 5304 | 3808 | * |
| 1367724_a_at | Atp6v0e1 | H+ transport (lysosomes) | 5677 | 3302 | * |
| 1371402_at | Atp6v1b2 | H+ transport (lysosomes) | 3164 | 3069 |  |
| 1388325_at | Atp6v1d | H+ transport (lysosomes) | 8039 | 7035 | * |
| 1398781_at | Atp6v1f | H+ transport (lysosomes) | 9184 | 5803 | * |
| 1398905_at | Atp6v1g1 | H+ transport (lysosomes) | 3348 | 1820 | * |
| 1370275_at | Atp5b | H+ transport (mitochondrial) | 16238 | 19161 | * |
| 1370918_a_at | Atp5c1 | H+ transport (mitochondrial) | 12840 | 13474 |  |
| 1370278_at | Atp5d | H+ transport (mitochondrial) | 9201 | 7283 | * |
| 1370284_at | Atp5e | H+ transport (mitochondrial) | 11374 | 9476 |  |
| 1398855_at | Atp5f1 | H+ transport (mitochondrial) | 11688 | 13520 | * |
| 1367599_at | Atp5g1 | H+ transport (mitochondrial) | 11714 | 11167 |  |
| 1370207_at | Atp5g2 | H+ transport (mitochondrial) | 7370 | 6039 |  |
| 1367620_at | Atp5g3 | H+ transport (mitochondrial) | 16123 | 16082 |  |
| 1367622_at | Atp5h | H+ transport (mitochondrial) | 12963 | 12697 |  |
| 1387019_at | Atp5i | H+ transport (mitochondrial) | 5413 | 4287 |  |
| 1370230_at | Atp5j | H+ transport (mitochondrial) | 13143 | 11062 | * |
| 1371398_at | Atp5j2 | H+ transport (mitochondrial) | 11318 | 10774 |  |
| 1371335_at | Atp5l | H+ transport (mitochondrial) | 12775 | 11265 |  |
| 1370276_at | Atp5o | H+ transport (mitochondrial) | 13488 | 13096 |  |
| 1386981_at | Slc16a1 | Monocarboxylic acid transport | 2561 | 5156 | * |
| 1368965_at | Slc16a3 | Monocarboxylic acid transport | 4961 | 4310 |  |
| 1374171_at | Abcc9 | Potassium channel | 2039 | 3351 | * |
| 1370060_at | Slc25a11 | Oxoglutarate transport | 5632 | 4420 | * |
| 1370277_at | Slc25a3 | Phosphate transport | 16464 | 17836 |  |
| 1371911_at | Tnfaip1 | Potassium channel | 3579 | 2603 | * |
| 1372449_at | Slc8a1 | Sodium/calcium channel | 2555 | 6888 | * |
| 1367585_a_at, 1371108_a_at | Atp1a1 | Sodium/potassium channel | 3547, 1028 | 7852, 5621 | * |
| 1367814_at, 1386937_at | Atp1b1 | Sodium/potassium channel | 5192, 5069 | 6418, 6982 |  |
| 1398300_at | Atp1b3 | Sodium/potassium channel | 4516 | 5172 | * |
| 1372778_at | Slc39a1 | Zinc transporter | 6894 | 4807 | * |
| --- | --- | --- | --- | --- |  |
| **Cytoskeleton and myofibrillar apparatus** | | | --- | --- |  |
| 1398835_at, 1398836_s_at, AFFX_Rat_beta-actin_3/5/M_at | Actb | Actin structures | 20182, 15793, 17848, 10805, 15490 | 21594, 14562, 18982, 11775, 14148 |  |
| 1373048_at | Actr10 | Actin structures | 3783 | 3504 |  |
| 1375424_at, 1398626_s_at | Actr2 | Actin structures | 5763, 9428 | 3225, 7611 | * |
| 1370890_at | Actr3 | Actin structures | 5635 | 6054 |  |
| 1388487_at | Add1 | Actin structures | 2834 | 3087 | * |
| 1392597_at | Aif1l | Actin structures | 4048 | 2975 | * |
| 1387018_at | Argbp2 | Actin structures | 4316 | 7953 | * |
| 1386925_at | Arpc1b | Actin structures | 9190 | 7427 |  |
| 1371511_at, 1375137_at | Arpc2 | Actin structures | 8917, 3833 | 7452, 3926 | * |
| 1371977_at | Arpc3 | Actin structures | 7503 | 5695 | * |
| 1372577_at | Arpc4 | Actin structures | 3959 | 1984 | * |
| 1390022_at | Arpc5 | Actin structures | 7388 | 5209 | * |
| 1370184_at | Cfl1 | Actin structures | 11089 | 8535 | * |
| 1388483_at | Cfl2 | Actin structures | 8406 | 7460 |  |
| 1387856_at | Cnn3 | Actin structures | 4993 | 4307 |  |
| 1371632_at | Coro1c | Actin structures | 3126 | 2498 | * |
| 1371921_at | Ctnna1 | Actin structures | 5245 | 9966 | * |
| 1373291_at | Dlc1 | Actin structures | 2304 | 3183 | * |
| 1371375_at, 1375881_at | Dstn | Actin structures | 12105, 7992 | 11475, 8206 | * |
| 1370875_at | Ezr | Actin structures | 1822 | 4000 | * |
| 1398327_at | Fermt2 | Actin structures | 4234 | 4904 | * |
| 1371382_at | Flna | Actin structures | 2282 | 5607 | * |
| 1371414_at | Gsn | Actin structures | 2182 | 3464 | * |
| 1388566_at | Lasp1 | Actin structures | 5725 | 3342 | * |
| 1370948_a_at, 1373432_at, 1375523_at | Marcks | Actin structures | 2667, 3510, 3318 | 3927, 4824, 3975 | * |
| 1371575_at | Msn | Actin structures | 3211 | 5963 | * |
| 1370697_a_at, 1370854_at | Nexn | Actin structures | 1519, 1347 | 3423, 3264 | * |
| 1389172_at | Pdlim5 | Actin structures | 3342 | 5584 | * |
| 1372745_at | Pdlim5 | Actin structures | 3295 | 3942 |  |
| 1372037_at | Pdlim7 | Actin structures | 5185 | 2517 | * |
| 1367605_at | Pfn1 | Actin structures | 10908 | 6983 | * |
| 1372947_at | Pls3 | Actin structures | 3134 | 4792 | * |
| 1398762_at | Sdcbp | Actin structures | 10308 | 11854 | * |
| 1370165_at | Smpx | Actin structures | 9898 | 7863 | * |
| 1371679_at | Synpo2 | Actin structures | 2692 | 4171 | * |
| 1372979_at | Synpo2l | Actin structures | 5825 | 4866 | * |
| 1367570_at | Tagln | Actin structures | 4355 | 2778 |  |
| 1388335_at | Tagln2 | Actin structures | 10588 | 8777 | * |
| 1367655_at | Tmsb10 | Actin structures | 7339 | 9018 |  |
| 1387883_a_at | Tmsb4x | Actin structures | 17759 | 20502 |  |
| 1371954_at | T1 | Actin structures | 3350 | 4330 | * |
| 1372459_at | Vasp | Actin structures | 3533 | 2563 | * |
| 1372905_at, 1375538_at | Vcl | Actin structures | 1645, 2576 | 4753, 6175 | * |
| 1371821_at | Wasf2 | Actin structures | 3333 | 3452 |  |
| 1388130_at | Zyx | Actin structures | 5677 | 4942 |  |
| 1367600_at | Des | Intermediate filaments | 7668 | 12315 | * |
| 1388506_at | Dsp | Intermediate filaments | 1423 | 5508 | * |
| 1367574_at | Vim | Intermediate filaments | 13568 | 17442 | * |
| 1372111_at, 1393281_at | Cav1 | Lipid rafts | 6591, 3179 | 5455, 3500 | * |
| 1387814_at | Cav3 | Lipid rafts | 3806 | 2961 |  |
| 1371741_at | Actr1a | Microtubules | 3789 | 3234 |  |
| 1398900_at | Dctn3 | Microtubules | 3669 | 2273 | * |
| 1371533_at | Dctn6 | Microtubules | 3628 | 2413 | * |
| 1370804_at | Gabarap | Microtubules | 11527 | 9639 | * |
| 1371680_at | Gabarapl1 | Microtubules | 3571 | 1554 | * |
| 1367783_at | Gabarapl2 | Microtubules | 7042 | 4177 | * |
| 1371682_at | Map1lc3a | Microtubules | 8401 | 5277 | * |
| 1367669_a_at | Map1lc3b | Microtubules | 4599 | 3704 | * |
| 1373397_at | Mapre1 | Microtubules | 4450 | 4268 |  |
| 1386857_at | Stmn1 | Microtubules | 3576 | 2049 | * |
| 1367579_a_at | Tuba1a/b/c | Microtubules | 17378 | 21101 | * |
| 1371542_at | Tuba4a | Microtubules | 2367 | 4507 | * |
| 1373718_at | Tubb2a | Microtubules | 3222 | 3280 |  |
| 1371390_at | Tubb2c | Microtubules | 6205 | 7180 | * |
| 1370290_at, 1387892_at | Tubb5 | Microtubules | 4528, 9667 | 4890, 12055 |  |
| 1376100_at | Tubb6 | Microtubules | 4355 | 2958 | * |
| 1369928_at | Acta1 | Myofilaments | 14421 | 17121 | * |
| 1370857_at | Acta2 | Myofilaments | 6780 | 7006 |  |
| 1370856_at, 1374352_at, 1374353_x_at, 1385797_at | Actc1 | Myofilaments | 19152 | 23415 |  |
| 1371327_a_at | Actg1 | Myofilaments | 13994 | 16684 | * |
| 1389189_at | Actn1 | Myofilaments | 1886 | 3393 | * |
| 1372595_at | Actn2 | Myofilaments | 3671 | 11095 | * |
| 1371969_at | Cald1 | Myofilaments | 2763 | 3539 | * |
| 1392900_at | Capza1 | Myofilaments | 3462 | 2715 | * |
| 1399043_at | Capza2 | Myofilaments | 3967 | 3358 |  |
| 1399091_at | Capzb | Myofilaments | 7248 | 5244 | * |
| 1370057_at | Csrp1 | Myofilaments | 10097 | 6318 | * |
| 1398243_at | Csrp3 | Myofilaments | 14324 | 13171 |  |
| 1371984_at, 1375982_at | Ldb3 | Myofilaments | 4269, 2943 | 6846, 4529 | * |
| 1388597_at | Mybpc3 | Myofilaments | 3654 | 8175 | * |
| 1368093_at, 1387049_at | Myh6 | Myofilaments | 6917, 266 | 19459, 6492 | * |
| 1398248_s_at | Myh6/ Myh7 | Myofilaments | 3720 | 15488 | * |
| 1367928_at, 1386993_at | Myh7 | Myofilaments | 561, 7200 | 7169, 21096 | * |
| 1388200_at | Myl2 | Myofilaments | 15704 | 17783 |  |
| 1367572_at | Myl3 | Myofilaments | 14718 | 17557 | * |
| 1371293_at | Myl4 | Myofilaments | 9007 | 9159 |  |
| 1371315_at | Myl7 | Myofilaments | 8608 | 7747 |  |
| 1388298_at | Myl9 | Myofilaments | 7464 | 5795 |  |
| 1373130_at | Myom2 | Myofilaments | 1527 | 3671 | * |
| 1371801_at | Myoz2 | Myofilaments | 9992 | 10828 |  |
| 1389532_at | Nebl | Myofilaments | 1577 | 4358 | * |
| 1388718_at | Tmod1 | Myofilaments | 2426 | 3487 | * |
| 1371354_at | Tnnc1 | Myofilaments | 18120 | 19581 |  |
| 1371339_at, 1386873_at | Tnni1 | Myofilaments | 9822, 10457 | 7835, 9602 | * |
| 1386931_at | Tnni3 | Myofilaments | 12325 | 11812 |  |
| 1367592_at | Tnnt2 | Myofilaments | 18762 | 21959 | * |
| 1370287_a_at | Tpm1 | Myofilaments | 4050 | 2268 | * |
| 1368724_a_at | Tpm1 | Myofilaments | 16941 | 20445 | * |
| 1370288_a_at | Tpm1 | Myofilaments | 12426 | 15487 | * |
| 1371241_x_at | Tpm1 | Myofilaments | 14353 | 17640 |  |
| 1372219_at | Tpm2 | Myofilaments | 5104 | 3586 | * |
| 1370339_at | Tpm3 | Myofilaments | 6966 | 5491 |  |
| 1383986_at | Tpm3 | Myofilaments | 6866 | 4719 | * |
| 1368838_at, 1371653_at | Tpm4 | Myofilaments | 5374, 10838 | 4932, 8669 |  |
| 1375518_at | Ttn | Myofilaments | 1323 | 4429 | * |
| 1386996_at | Mrlc2 | Myosin structures | 4704 | 2795 | * |
| 1371725_at | Myh9 | Myosin structures | 1115 | 3504 | * |
| 1371304_a_at | Myl6 | Myosin structures | 13514 | 10801 | * |
| 1369631_at | Myo1c | Myosin structures | 3807 | 4554 |  |
| 1388445_at | Anxa11 | Nuclear envelope | 3052 | 3710 |  |
| 1368151_at | Matr3 | Nuclear matrix | 1643 | 3306 | * |
| 1367614_at | Anxa1 | Sarcolemma | 4772 | 4962 |  |
| 1367584_at | Anxa2 | Sarcolemma | 10360 | 10831 |  |
| 1386862_at | Anxa5 | Sarcolemma | 7746 | 8350 |  |
| 1387673_a_at | Anxa6 | Sarcolemma | 2760 | 3841 | * |
| 1368143_at | Anxa7 | Sarcolemma | 2928 | 2817 |  |
| 1371429_at | Dag1 | Sarcolemma | 1483 | 3396 | * |
| 1372273_at | Gypc | Sarcolemma | 3668 | 2537 | * |
| 1372159_at | Jph2 | Sarcolemma | 5161 | 3567 |  |
| 1372949_at | Sept11 | Sarcolemma | 3128 | 2767 |  |
| 1388389_at | Sept2 | Sarcolemma | 7029 | 7661 |  |
| 1374513_at | Sept7 | Sarcolemma | 4047 | 5925 | * |
| 1370838_s_at | Spna2 | Sarcolemma | 1516 | 5860 | * |
| 1395359_at | Spnb2 | Sarcolemma | 2622 | 5117 | * |
| 1371419_at | Sptbn1 | Sarcolemma | 1761 | 6675 | * |
| 1388866_at | Sspn | Sarcolemma | 4258 | 4521 |  |
| --- | --- | --- | --- | --- |  |
| **DNA/RNA regulation** | | | --- | --- |  |
| 1371772_at | Alkbh3 | DNA structure/repair | 3803 | 2788 | * |
| 1367820_at | Banf1 | DNA structure/repair | 5907 | 3039 | * |
| 1371395_at | Cbx3 | DNA structure/repair | 5704 | 4551 | * |
| 1371648_at | Ddb1 | DNA structure/repair | 2962 | 4516 | * |
| 1367759_at | H1f0 | DNA structure/repair | 4084 | 2462 | * |
| 1388827_at | H2afv | DNA structure/repair | 5654 | 2956 | * |
| 1386861_at | H2afz | DNA structure/repair | 13432 | 9739 | * |
| 1370190_at, 1390019_at, 1398849_at, 1398888_at | H3f3b | DNA structure/repair | 13832, 13358, 9632, 10584 | 10846, 10930, 6969, 9055 | * |
| 1371332_at | Hist1h1c | DNA structure/repair | 7343 | 5749 | * |
| 1388314_at | Hmgn1 | DNA structure/repair | 13247 | 9690 | * |
| 1371352_at | Hmgn2 | DNA structure/repair | 10429 | 8101 | * |
| 1386004_s_at, 1399057_at | Morf4l1 | DNA structure/repair | 10020, 7953 | 10046, 7998 |  |
| 1371422_at | Morf4l2 | DNA structure/repair | 9287 | 9680 |  |
| 1370826_at | Nap1l1 | DNA structure/repair | 4102 | 4762 | * |
| 1367671_at | Pcna | DNA structure/repair | 4065 | 3966 |  |
| 1388367_at | Pole4 | DNA structure/repair | 3674 | 2554 | * |
| 1372460_at, 1372461_at | Set | DNA structure/repair | 3951, 6428 | 3400, 4478 | * |
| 1367500_at | Shfm1 | DNA structure/repair | 4500 | 3571 |  |
| 1387653_at | Tsnax | DNA structure/repair | 3092 | 2168 | * |
| 1370803_at | Zwint | DNA structure/repair | 4090 | 3397 | * |
| 1372242_at | Ddx3x | RNA helicase | 4131 | 6551 | * |
| 1371837_at | Ddx5 | RNA helicase | 5082 | 9579 | * |
| 1398937_at | Dhx15 | RNA helicase | 1420 | 2938 | * |
| 1388158_at | Bat1 | mRNA splicing | 3030 | 3548 | * |
| 1372720_at, 1376333_at | Btbd1 | mRNA splicing | 5516, 4210 | 4303, 3418 |  |
| 1388704_at | Cugbp1 | mRNA splicing | 3130 | 3392 |  |
| 1373195_at | Fus | mRNA splicing | 3684 | 4527 |  |
| 1388446_at | Hnrnpa0 | mRNA splicing | 5943 | 5679 |  |
| 1398883_at | Hnrnpa2b1 | mRNA splicing | 6132 | 5907 |  |
| 1370871_at, 1388119_at | Hnrnpa3 | mRNA splicing | 7685, 5256 | 7254, 5454 |  |
| 1370309_a_at | Hnrnpab | mRNA splicing | 7382 | 6554 |  |
| 1371505_at | Hnrnpc | mRNA splicing | 3711 | 3501 |  |
| 1367895_at, 1398797_at | Hnrnpk | mRNA splicing | 4674, 5862 | 6242, 8047 | * |
| 1398873_at | Hnrnpl | mRNA splicing | 6128 | 6618 |  |
| 1370170_at | Hnrnpu | mRNA splicing | 3097 | 4862 | * |
| 1372810_at | Hnrpdl | mRNA splicing | 2529 | 3690 | * |
| 1371683_at | Lsm4 | mRNA splicing | 4059 | 1967 | * |
| 1372815_at | Magoh | mRNA splicing | 4822 | 3123 | * |
| 1375630_at | Nhp2l1 | mRNA splicing | 4655 | 2905 | * |
| 1398904_at | Nono | mRNA splicing | 5149 | 6703 | * |
| 1367466_at | Prpf8 | mRNA splicing | 1102 | 3779 | * |
| 1367931_a_at | Ptbp1 | mRNA splicing | 3851 | 4025 |  |
| 1374463_at | Qk | mRNA splicing | 4792 | 4737 |  |
| 1399101_at | Rbm39 | mRNA splicing | 1682 | 3636 | * |
| 1372829_at | Rbm8 | mRNA splicing | 3302 | 2035 | * |
| 1390234_at | Sf3b1 | mRNA splicing | 1288 | 3548 | * |
| 1371396_at | Sf3b14 | mRNA splicing | 5265 | 3723 | * |
| 1388614_at | Sf3b2 | mRNA splicing | 2710 | 3075 |  |
| 1371324_at | Sf3b5 | mRNA splicing | 4474 | 2418 | * |
| 1370188_at, 1370189_at | Sfrs10 | mRNA splicing | 3157, 3398 | 3350, 3955 |  |
| 1371838_at | Sfrs2 | mRNA splicing | 4448 | 4577 |  |
| 1368992_a_at | Sfrs5 | mRNA splicing | 2390 | 3568 | * |
| 1372075_at | Sfrs6 | mRNA splicing | 2454 | 3007 | * |
| 1373563_at | Snrnp27 | mRNA splicing | 4436 | 2337 | * |
| 1388117_at | Snrpb | mRNA splicing | 5837 | 5228 |  |
| 1371486_at | Snrpc | mRNA splicing | 4548 | 2796 | * |
| 1371341_at | Snrpd2 | mRNA splicing | 5281 | 4089 |  |
| 1371848_at | Snrpd3 | mRNA splicing | 4198 | 2090 | * |
| 1372333_at | Snrpe | mRNA splicing | 8082 | 6914 | * |
| 1379542_at | Snrpf | mRNA splicing | 4950 | 2777 | * |
| 1372167_at | Snrpg | mRNA splicing | 3835 | 2845 |  |
| 1372077_at | Strap | mRNA splicing | 5950 | 4958 | * |
| 1367493_at, 1371971_at | Txnl4a | mRNA splicing | 3480, 3225 | 2292, 1775 | * |
| 1393431_at | U2af1 | mRNA splicing | 3273 | 2103 | * |
| 1388797_at | U2af2 | mRNA splicing | 3923 | 3170 | * |
| 1388355_at | Rbm17 | RNA binding | 3721 | 2118 | * |
| 1374056_at | Rbm24 | RNA binding | 5609 | 6166 |  |
| 1372380_at, 1375898_at | Rbpms | RNA binding | 3741, 3962 | 2334, 3348 | * |
| 1388076_at | Serbp1 | RNA binding | 6924 | 6652 |  |
| 1371475_at | Rnase4 | RNA degradation | 4972 | 3302 | * |
| 1398923_at | Rnasek | RNA degradation | 5773 | 4758 |  |
| 1375211_at | Rnaset2 | RNA degradation | 3607 | 3441 |  |
| 1370880_at | Rnh1 | RNA degradation | 3888 | 2790 | * |
| 1371596_at | Rnps1 | RNA degradation | 4437 | 3251 | * |
| 1398801_at | Cdk105 | rRNA synthesis/processing | 3733 | 5601 | * |
| 1388528_at | Fbl | rRNA synthesis/processing | 3281 | 2046 | * |
| 1367494_at | Fcf1 | rRNA synthesis/processing | 4151 | 3065 | * |
| 1398832_at | Ncl | rRNA synthesis/processing | 4065 | 6506 | * |
| 1371539_at | Nola2 | rRNA synthesis/processing | 3249 | 1619 | * |
| 1367479_at | Nola3 | rRNA synthesis/processing | 7338 | 5510 | * |
| 1398757_at, 1399158_a_at | Npm1 | rRNA synthesis/processing | 9938, 10083 | 12164, 9932 |  |
| 1371612_at | Tsen34 | tRNA processing | 5052 | 3079 | * |
| --- | --- | --- | --- | --- |  |
| **Intracellular signalling** | | | --- | --- |  |
| 1383358_at | Akap1 | Adaptors/scaffold proteins | 1222 | 3437 | * |
| 1398369_at | Akap13 | Adaptors/scaffold proteins | 1755 | 2792 | * |
| 1373843_at | Akap2 | Adaptors/scaffold proteins | 4050 | 5706 | * |
| 1371517_at | Grb10 | Adaptors/scaffold proteins | 2860 | 5263 | * |
| 1372088_at | Ppfibp1 | Adaptors/scaffold proteins | 2741 | 3615 | * |
| 1372296_at | Sh3bgr | Adaptors/scaffold proteins | 9503 | 8792 |  |
| 1387294_at | Sh3bp5 | Adaptors/scaffold proteins | 3594 | 3197 |  |
| 1387957_a_at | Sh3kbp1 | Adaptors/scaffold proteins | 5669 | 4576 |  |
| 1377594_at | Shc1 | Adaptors/scaffold proteins | 3385 | 2181 | * |
| 1386999_at, 1398800_at | Ywhab | Adaptors/scaffold proteins | 3448, 5861 | 3164, 5779 |  |
| 1398851_at | Ywhae | Adaptors/scaffold proteins | 8536 | 7149 |  |
| 1386866_at | Ywhag | Adaptors/scaffold proteins | 8060 | 7454 |  |
| 1367693_at | Ywhah | Adaptors/scaffold proteins | 8414 | 7308 | * |
| 1370168_at, 1387862_at | Ywhaq | Adaptors/scaffold proteins | 6970, 10152 | 6816, 9543 |  |
| 1372249_at, 1387774_at | Ywhaz | Adaptors/scaffold proteins | 4788, 6295 | 3745, 5740 | * |
| 1387805_at | Bnip3 | Apoptosis | 9893 | 8553 |  |
| 1367898_at, 1386978_at | Bnip3l | Apoptosis | 5062, 3305 | 3037, 2262 | * |
| 1373733_at | Bok | Apoptosis | 3400 | 1974 | * |
| 1372498_at | Ciapin1 | Apoptosis | 3559 | 3305 |  |
| 1389179_at | Cidea | Apoptosis | 4621 | 4218 |  |
| 1388861_at | Clptm1l | Apoptosis | 4249 | 3051 | * |
| 1367784_a_at | Clu | Apoptosis | 10062 | 18228 | * |
| 1369941_at | Dap | Apoptosis | 7017 | 4880 | * |
| 1372076_at | Hbxip | Apoptosis | 5086 | 2962 | * |
| 1388587_at | Ier3 | Apoptosis | 4989 | 4246 |  |
| 1371319_at, 1371320_at | Itm2b | Apoptosis | 11949, 10591 | 13056, 11088 |  |
| 1372520_at, 1373225_at | Mcl1 | Apoptosis | 5284, 3438 | 5372, 3427 |  |
| 1389454_at | Pdcd5 | Apoptosis | 6631 | 4195 | * |
| 1388767_at | Pdcd6 | Apoptosis | 6932 | 4670 | * |
| 1375224_at | Phlda3 | Apoptosis | 4379 | 1732 | * |
| 1370156_at | Prnp | Apoptosis | 4441 | 5655 | * |
| 1388307_at | Serinc1 | Apoptosis | 5094 | 8020 | * |
| 1398986_at | Serinc3 | Apoptosis | 3800 | 4989 |  |
| 1388776_at | Shisa5 | Apoptosis | 5088 | 3665 | * |
| 1398948_at | Tax1bp1 | Apoptosis | 3607 | 4645 | * |
| 1369879_a_at | Tmbim6 | Apoptosis | 4612 | 4786 |  |
| 1371309_at | Tmbim6 | Apoptosis | 5487 | 4276 | * |
| 1367583_at | Tpt1 | Apoptosis | 12306 | 17374 | * |
| 1371786_at | Trim35 | Apoptosis | 3817 | 4131 |  |
| 1398817_at | Arf1 | G proteins | 5188 | 3374 | * |
| 1367459_at | Arf1 | G proteins | 13457 | 9034 | * |
| 1388997_at | Arf3 | G proteins | 4841 | 2948 | * |
| 1398758_at | Arf4 | G proteins | 7887 | 7247 |  |
| 1367748_at | Arf5 | G proteins | 3468 | 1560 | * |
| 1371994_at | Arhgap1 | G proteins | 3749 | 4210 |  |
| 1371366_at | Arhgdia | G proteins | 9733 | 6575 | * |
| 1387358_at | Arl1 | G proteins | 3514 | 3177 |  |
| 1389941_at | Arl2bp | G proteins | 4770 | 3254 | * |
| 1374387_at | Arl6ip5 | G proteins | 4574 | 3746 | * |
| 1373133_at | Arl8b | G proteins | 3616 | 3359 |  |
| 1367475_at, 1370825_a_at | Cdc42 | G proteins | 7081, 8408 | 6154, 6880 | * |
| 1375910_at | Cdc42ep3 | G proteins | 4839 | 3587 | * |
| 1367460_at, 1398822_at | Gdi2 | G proteins | 5840, 2254 | 7421, 4085 | * |
| 1371798_at | Gna12 | G proteins | 4439 | 3325 | * |
| 1367844_at | Gnai2 | G proteins | 5399 | 3008 | * |
| 1368029_at | Gnai3 | G proteins | 3329 | 3317 |  |
| 1369897_s_at | Gnas | G proteins | 16008 | 16335 |  |
| 1367732_at | Gnb1 | G proteins | 3753 | 2268 | * |
| 1370867_at, 1398863_at | Gnb2 | G proteins | 3703, 7098 | 2463, 4986 | * |
| 1367618_a_at | Gnb2l1 | G proteins | 12421 | 15646 | * |
| 1388455_at | Gng10 | G proteins | 5501 | 4155 | * |
| 1367902_at | Gng11 | G proteins | 3884 | 2610 | * |
| 1392471_at | Gng12 | G proteins | 3338 | 2075 | * |
| 1387342_at | Gng5 | G proteins | 10660 | 10178 |  |
| 1371255_at | Hras | G proteins | 3356 | 1732 | * |
| 1398841_at | Rab1 | G proteins | 12104 | 11307 |  |
| 1398814_at | Rab11a | G proteins | 7053 | 4116 | * |
| 1375163_at | Rab11b | G proteins | 4719 | 3366 | * |
| 1371871_at | Rab12 | G proteins | 6754 | 4845 | * |
| 1367884_at | Rab14 | G proteins | 4574 | 2825 | * |
| 1389292_at | Rab18 | G proteins | 3487 | 2948 |  |
| 1384148_at | Rab20 | G proteins | 3472 | 2242 | * |
| 1383089_at | Rab21 | G proteins | 3269 | 2252 | * |
| 1368216_at | Rab28 | G proteins | 3056 | 2890 |  |
| 1370087_at, 1392681_at | Rab2a | G proteins | 5457, 4484 | 3823, 3640 | * |
| 1373894_at | Rab31 | G proteins | 4653 | 2827 | * |
| 1383786_at, 1390866_at | Rab32 | G proteins | 4409, 5905 | 2753, 4198 |  |
| 1376573_at | Rab34 | G proteins | 4006 | 2254 | * |
| 1388823_at | Rab5b | G proteins | 3831 | 2286 | * |
| 1372707_at | Rab6a | G proteins | 3697 | 3691 |  |
| 1387797_at | Rab7a | G proteins | 5238 | 4278 | * |
| 1398780_at | Rabac1 | G proteins | 5401 | 4021 | * |
| 1372513_at, 1388332_at | Rac1 | G proteins | 6385, 8475 | 5422, 6667 |  |
| 1388504_at | Rad21 | G proteins | 3133 | 4072 | * |
| 1388550_at | Rad23b | G proteins | 5770 | 5092 | * |
| 1367590_at | Ran | G proteins | 11178 | 9709 | * |
| 1388615_at | Rap1a | G proteins | 4643 | 4171 |  |
| 1387806_at | Rap1b | G proteins | 4653 | 3690 | * |
| 1398787_at | Rheb | G proteins | 8147 | 5983 | * |
| 1399027_at | Rhoa | G proteins | 5738 | 4588 | * |
| 1369958_at | Rhob | G proteins | 3705 | 2376 | * |
| 1371659_at | Rhoc | G proteins | 5635 | 3315 | * |
| 1386967_at | Rhoq | G proteins | 4322 | 3526 |  |
| 1371723_at | Rragc | G proteins | 4173 | 2727 | * |
| 1388729_at | Rras | G proteins | 4221 | 2716 | * |
| 1383126_at | Akt1 | Kinases/kinase regulators | 8249 | 5097 | * |
| 1372879_at | Akt1s1 | Kinases/kinase regulators | 3639 | 2203 | * |
| 1375266_at | Ccnd2 | Kinases/kinase regulators | 6900 | 6213 |  |
| 1369935_at | Ccnd3 | Kinases/kinase regulators | 4467 | 2469 | * |
| 1367764_at | Ccng1 | Kinases/kinase regulators | 8301 | 9699 | * |
| 1388370_at | Ccni | Kinases/kinase regulators | 6195 | 6155 |  |
| 1388443_at | Cdk2ap1 | Kinases/kinase regulators | 7229 | 4777 | * |
| 1369950_at | Cdk4 | Kinases/kinase regulators | 6414 | 4035 | * |
| 1388674_at | Cdkn1a | Kinases/kinase regulators | 4359 | 2117 | * |
| 1374392_at, 1374646_at | Csnk1a1 | Kinases/kinase regulators | 3329, 5598 | 3923, 6210 | * |
| 1375636_at | Csnk2a1 | Kinases/kinase regulators | 2218 | 3257 |  |
| 1387108_at | Csnk2b | Kinases/kinase regulators | 6077 | 4142 | * |
| 1373915_at | Dmpk | Kinases/kinase regulators | 4755 | 3147 | * |
| 1387777_at | Ilk | Kinases/kinase regulators | 4230 | 3785 |  |
| 1383478_at | Jak1 | Kinases/kinase regulators | 3116 | 6376 | * |
| 1390207_at | Lats2 | Kinases/kinase regulators | 3024 | 3113 |  |
| 1367760_at | Map2k1 | Kinases/kinase regulators | 3626 | 3619 |  |
| 1371587_at | Map2k1ip1 | Kinases/kinase regulators | 4153 | 2944 | * |
| 1398834_at | Map2k2 | Kinases/kinase regulators | 4203 | 1762 | * |
| 1388858_at | Map2k3 | Kinases/kinase regulators | 4963 | 2245 | * |
| 1371446_at | Mapkapk2 | Kinases/kinase regulators | 3749 | 2726 | * |
| 1369944_at | Marcksl1 | Kinases/kinase regulators | 9053 | 5966 | * |
| 1371998_at | Mobkl1b | Kinases/kinase regulators | 3557 | 2484 | * |
| 1372475_at | Pink1 | Kinases/kinase regulators | 3496 | 2651 | * |
| 1373082_at | Pkia | Kinases/kinase regulators | 6861 | 6605 |  |
| 1371600_at | Pkig | Kinases/kinase regulators | 3869 | 3000 |  |
| 1373951_at, 1386905_at, 1388375_at | Prkar1a | Kinases/kinase regulators | 5137, 4619, 5014 | 5931, 5532, 6000 | * |
| 1367691_at | Prkcdbp | Kinases/kinase regulators | 13235 | 11592 |  |
| 1388371_at | Prkcsh | Kinases/kinase regulators | 2591 | 3364 | * |
| 1372727_at | Socs2 | Kinases/kinase regulators | 3899 | 3599 |  |
| 1379271_at | Socs5 | Kinases/kinase regulators | 4598 | 4491 |  |
| 1371353_at | Sqstm1 | Kinases/kinase regulators | 7415 | 6728 |  |
| 1372791_at | Wnk1 | Kinases/kinase regulators | 2620 | 2973 |  |
| 1372606_at | Zak | Kinases/kinase regulators | 2907 | 2918 |  |
| 1389050_at | Dusp3 | Phosphatases | 3528 | 2362 | * |
| 1387024_at | Dusp6 | Phosphatases | 3778 | 2253 | * |
| 1369980_s_at | Mprip | Phosphatases | 5899 | 4756 | * |
| 1371543_at | Mtmr2 | Phosphatases | 2784 | 3027 |  |
| 1398997_at | Phpt1 | Phosphatases | 4526 | 2949 | * |
| 1369961_at | Ppap2a | Phosphatases | 3430 | 3233 |  |
| 1386863_at | Ppp1ca | Phosphatases | 10649 | 7314 | * |
| 1386950_at | Ppp1cb | Phosphatases | 5369 | 6983 | * |
| 1367637_a_at | Ppp1cc | Phosphatases | 8691 | 7434 | * |
| 1389815_at | Ppp1r14b | Phosphatases | 6636 | 5089 | * |
| 1372446_at | Ppp1r2 | Phosphatases | 3874 | 3170 |  |
| 1398790_at | Ppp2ca | Phosphatases | 5382 | 5440 |  |
| 1367827_at | Ppp2cb | Phosphatases | 5342 | 3894 | * |
| 1375245_at | Ppp2r1a | Phosphatases | 4469 | 4960 |  |
| 1399005_at | Ppp2r5a | Phosphatases | 3595 | 2615 | * |
| 1375298_at | Ppp2r5c | Phosphatases | 4326 | 3410 | * |
| 1370193_at | Ptp4a1 | Phosphatases | 5151 | 3509 | * |
| 1367971_at | Ptp4a2 | Phosphatases | 7426 | 5334 | * |
| 1372084_at | Ptp4a3 | Phosphatases | 4401 | 2980 | * |
| 1383696_at, 1392953_at | Ptpla | Phosphatases | 5349, 6993 | 6900, 8216 | * |
| 1388379_at | Ptpn11 | Phosphatases | 3212 | 2718 |  |
| 1388686_at | Rcan1 | Phosphatases | 7512 | 5992 |  |
| 1384202_at | Tesc | Phosphatases | 3658 | 2223 | * |
| --- | --- | --- | --- | --- |  |
| **Protein processing/modification** | | | --- | --- |  |
| 1372065_at | Art3 | ADP-ribosylation | 4060 | 7671 | * |
| 1367687_a_at | Pam | Amidation | 6749 | 10224 | * |
| 1367595_s_at | B2m | Antigen processing | 14517 | 14511 |  |
| 1372070_at | Ifi30 | Antigen processing | 3522 | 3045 |  |
| 1388071_x_at | RT1-Aw2 | Antigen processing | 7024 | 6163 |  |
| 1371693_at | Ahsa1 | Chaperones/protein folding | 3392 | 1624 | * |
| 1375550_at | Bag1 | Chaperones/protein folding | 3540 | 2139 | * |
| 1390026_at | Bag3 | Chaperones/protein folding | 3045 | 3606 | * |
| 1371418_at | Cct2 | Chaperones/protein folding | 3184 | 4732 | * |
| 1371403_at | Cct3 | Chaperones/protein folding | 4010 | 5094 | * |
| 1371656_at | Cct4 | Chaperones/protein folding | 8500 | 9726 | * |
| 1376570_at, 1395508_at | Cct5 | Chaperones/protein folding | 5364, 3643 | 6890, 6269 | * |
| 1398960_at | Cct6a | Chaperones/protein folding | 2875 | 3708 | * |
| 1371641_at | Cct7 | Chaperones/protein folding | 3721 | 4177 | * |
| 1367453_at | Cdc37 | Chaperones/protein folding | 5449 | 3829 | * |
| 1370026_at | Cryab | Chaperones/protein folding | 16704 | 15913 |  |
| 1398819_at | Dnaja1 | Chaperones/protein folding | 2604 | 3009 | * |
| 1387780_at | Dnaja2 | Chaperones/protein folding | 3487 | 4009 | * |
| 1398343_at | Dnaja4 | Chaperones/protein folding | 3525 | 2448 | * |
| 1371666_at | Dnajc5 | Chaperones/protein folding | 3864 | 2485 | * |
| 1369621_s_at, 1398828_at, 1398829_at | Fkbp1a | Chaperones/protein folding | 7676, 10227, 13682 | 4748, 7523, 10982 | * |
| 1388882_at | Fkbp3 | Chaperones/protein folding | 4523 | 3790 | * |
| 1375640_at | Fkbp9 | Chaperones/protein folding | 2804 | 3691 |  |
| 1368552_at | Grpel1 | Chaperones/protein folding | 3420 | 2232 | * |
| 1372701_at, 1388850_at | Hsp90aa1 | Chaperones/protein folding | 7637, 3682 | 10914, 5227 | * |
| 1375335_at, 1375336_at | Hsp90ab1 | Chaperones/protein folding | 4625, 8026 | 8742, 11633 | * |
| 1388331_at | Hsp90b1 | Chaperones/protein folding | 3284 | 6209 | * |
| 1370283_at | Hspa5 | Chaperones/protein folding | 6251 | 9890 | * |
| 1398240_at | Hspa8 | Chaperones/protein folding | 12849 | 18361 | * |
| 1388851_at | Hspa9 | Chaperones/protein folding | 1520 | 3378 | * |
| 1367577_at | Hspb1 | Chaperones/protein folding | 10947 | 8396 | * |
| 1387898_at | Hspb6 | Chaperones/protein folding | 6565 | 3360 | * |
| 1372649_at | Hspb7 | Chaperones/protein folding | 11667 | 8856 | * |
| 1388721_at | Hspb8 | Chaperones/protein folding | 4875 | 4042 | * |
| 1386894_at | Hspd1 | Chaperones/protein folding | 6812 | 10257 | * |
| 1386898_at | Hspe1 | Chaperones/protein folding | 9393 | 7747 | * |
| 1398788_at | Pdia3 | Chaperones/protein folding | 3902 | 6158 | * |
| 1370859_at | Pdia6 | Chaperones/protein folding | 5695 | 9056 | * |
| 1398926_at | Pfdn1 | Chaperones/protein folding | 4304 | 3622 |  |
| 1372141_at | Pfdn2 | Chaperones/protein folding | 3476 | 1901 | * |
| 1398884_at | Pfdn5 | Chaperones/protein folding | 3287 | 2665 | * |
| 1371449_at | Pin1 | Chaperones/protein folding | 4935 | 2719 | * |
| 1398850_at | Ppia | Chaperones/protein folding | 17015 | 18752 |  |
| 1369934_at | Ppib | Chaperones/protein folding | 8290 | 8732 |  |
| 1372489_at | Slmap | Chaperones/protein folding | 4674 | 5440 |  |
| 1398848_at | St13 | Chaperones/protein folding | 3831 | 3951 |  |
| 1373408_at | Tbca | Chaperones/protein folding | 7906 | 6412 | * |
| 1371885_at | Tbcb | Chaperones/protein folding | 3558 | 2374 | * |
| 1368049_at | Tcp1 | Chaperones/protein folding | 4159 | 5258 | * |
| 1369943_at | Tgm2 | Crosslinking | 5112 | 8279 | * |
| 1368171_at | Lox | Cross-linking | 2592 | 4140 | * |
| 1372006_at | Loxl2 | Cross-linking | 2822 | 4266 | * |
| 1398938_at | Acp1 | Dephosphorylation | 3500 | 2261 | * |
| 1367465_at | Dad1 | Glycosylation | 6673 | 3854 | * |
| 1372247_at | Ddost | Glycosylation | 3671 | 5580 | * |
| 1375912_a_at | Galnt2 | Glycosylation | 3633 | 3204 |  |
| 1372337_at, 1388726_a_at | LOC100188932 | Glycosylation | 3967, 6362 | 2709, 4883 | * |
| 1398766_at | Rpn1 | Glycosylation | 6342 | 7279 |  |
| 1398776_at | Rpn2 | Glycosylation | 3325 | 5322 | * |
| 1389023_at | Stt3b | Glycosylation | 2926 | 4189 | * |
| 1370954_at | P4ha1 | Hydroxylation | 3147 | 4910 | * |
| 1372610_at | P4ha2 | Hydroxylation | 3607 | 4840 | * |
| 1367635_at | P4hb | Hydroxylation | 12566 | 16384 | * |
| 1367807_at | Plod1 | Hydroxylation | 2192 | 4168 | * |
| 1372897_at | Plod2 | Hydroxylation | 2421 | 4468 | * |
| 1375522_at | Nmt1 | Lipid modification | 3633 | 3475 |  |
| 1386897_at | Prmt1 | Methylation | 3682 | 2819 | * |
| 1389151_at | Adam19 | Proteolysis | 2572 | 3105 |  |
| 1388440_at | Aph1b | Proteolysis | 3708 | 2656 |  |
| 1398784_at | C1qbp | Proteolysis | 6770 | 7140 |  |
| 1383241_at | C1r | Proteolysis | 7254 | 7730 |  |
| 1387893_at | C1s | Proteolysis | 6873 | 11056 | * |
| 1388557_at, 1383291_at | C7 | Proteolysis | 4372, 3573 | 8902, 8308 | * |
| 1367462_at | Cap1 | Proteolysis | 8357 | 5534 | * |
| 1373029_at | Cops2 | Proteolysis | 3711 | 3840 |  |
| 1371476_at | Cops4 | Proteolysis | 4474 | 3662 | * |
| 1399011_at | Cops6 | Proteolysis | 3628 | 2496 | * |
| 1399010_at | Cops7a | Proteolysis | 3578 | 2693 | * |
| 1398955_at | Cops8 | Proteolysis | 3891 | 2128 | * |
| 1369215_a_at | Cpd | Proteolysis | 3060 | 5178 |  |
| 1386921_at | Cpe | Proteolysis | 5396 | 8957 | * |
| 1370855_at | Cst3 | Proteolysis | 8932 | 8147 |  |
| 1370226_at | Cstb | Proteolysis | 10775 | 9218 |  |
| 1367646_at | Ctsb | Proteolysis | 9453 | 11755 | * |
| 1367651_at | Ctsd | Proteolysis | 6228 | 8534 | * |
| 1386899_at | Ctsh | Proteolysis | 3972 | 4235 |  |
| 1370244_at, 1370245_at | Ctsl1 | Proteolysis | 13940, 3489 | 17627, 4118 | * |
| 1398954_at | Dnpep | Proteolysis | 4002 | 3759 |  |
| 1367722_at | Dpp7 | Proteolysis | 4417 | 4669 |  |
| 1386884_at | Htra1 | Proteolysis | 5084 | 5653 |  |
| 1368430_at | Lgmn | Proteolysis | 4350 | 6420 | * |
| 1398798_at | Metap2 | Proteolysis | 3653 | 3743 |  |
| 1367860_a_at | Mmp14 | Proteolysis | 9810 | 12359 | * |
| 1370301_at | Mmp2 | Proteolysis | 7688 | 10744 | * |
| 1386912_at | Pcolce | Proteolysis | 6448 | 6767 |  |
| 1386891_at | Pebp1 | Proteolysis | 12225 | 8620 | * |
| 1375215_x_at | Pgpep1 | Proteolysis | 4379 | 2518 | * |
| 1367800_at | Plat | Proteolysis | 1947 | 3445 | * |
| 1371325_at | Ppgb | Proteolysis | 3570 | 4986 | * |
| 1375280_at | Pppde1 | Proteolysis | 3864 | 1771 | * |
| 1373152_at | Prss23 | Proteolysis | 4808 | 6992 | * |
| 1371338_at | Psenen | Proteolysis | 5596 | 4184 | * |
| 1370220_at | Scpep1 | Proteolysis | 2901 | 3268 |  |
| 1372254_at | Serping1 | Proteolysis | 7284 | 9950 |  |
| 1371310_s_at | Serpinh1 | Proteolysis | 10504 | 11502 |  |
| 1371427_at | Spcs1 | Proteolysis | 9124 | 6745 | * |
| 1389140_at | Spcs2 | Proteolysis | 3419 | 2694 | * |
| 1371579_at | Spcs3 | Proteolysis | 3462 | 3449 |  |
| 1371677_at | Spink8 | Proteolysis | 4501 | 4088 |  |
| 1368323_at | Tfpi | Proteolysis | 4371 | 5362 | * |
| 1367712_at | Timp1 | Proteolysis | 7965 | 7503 |  |
| 1375144_at | Timp2 | Proteolysis | 4681 | 2618 | * |
| 1386940_at | Timp2 | Proteolysis | 11535 | 9965 |  |
| 1368843_at | Yme1l1 | Proteolysis | 1545 | 3021 | * |
| 1399095_at | Sumo1 | Sumoylation | 4487 | 3229 | * |
| 1367452_at | Sumo2 | Sumoylation | 7613 | 5159 | * |
| 1388640_at | Sumo3 | Sumoylation | 3452 | 2084 | * |
| 1367482_at | Anapc11 | Ubiquitin cycle | 4537 | 2214 | * |
| 1388417_at | Anapc5 | Ubiquitin cycle | 6830 | 4881 | * |
| 1388855_at | Cul1 | Ubiquitin cycle | 1989 | 2965 | * |
| 1399132_at | Cul3 | Ubiquitin cycle | 4259 | 4475 |  |
| 1371566_at | Fbxl22 | Ubiquitin cycle | 5714 | 4291 | * |
| 1370820_at | Fbxo6 | Ubiquitin cycle | 3328 | 2658 | * |
| 1372126_at | Fem1b | Ubiquitin cycle | 2839 | 2859 |  |
| 1372659_at | Kcmf1 | Ubiquitin cycle | 3602 | 3377 |  |
| 1371314_at, 1375119_at, 1383899_at, 1386263_at, 1395157_at | Nedd4 | Ubiquitin cycle | 7502, 13429, 2714, 1651, 2729 | 11562, 15614, 7000, 3437, 6336 | * |
| 1398860_at | Nedd8 | Ubiquitin cycle | 9855 | 6833 | * |
| 1398778_at | Psma1 | Ubiquitin cycle | 4682 | 4379 |  |
| 1398856_at | Psma2 | Ubiquitin cycle | 6039 | 5399 |  |
| 1368507_at | Psma3 | Ubiquitin cycle | 6667 | 5818 |  |
| 1387884_at | Psma5 | Ubiquitin cycle | 5162 | 4447 | * |
| 1369930_at | Psma6 | Ubiquitin cycle | 7919 | 6314 | * |
| 1371869_at | Psma7 | Ubiquitin cycle | 7227 | 4466 | * |
| 1398812_at | Psmb1 | Ubiquitin cycle | 8248 | 6786 | * |
| 1398786_at | Psmb2 | Ubiquitin cycle | 5209 | 3911 | * |
| 1398853_at | Psmb3 | Ubiquitin cycle | 6180 | 5147 | * |
| 1398831_at | Psmb4 | Ubiquitin cycle | 8927 | 7305 | * |
| 1371348_at | Psmb5 | Ubiquitin cycle | 8374 | 6362 | * |
| 1398777_at | Psmb6 | Ubiquitin cycle | 4324 | 3049 | * |
| 1367656_at | Psmb7 | Ubiquitin cycle | 6300 | 5473 | * |
| 1367711_at | Psmc2 | Ubiquitin cycle | 4528 | 5047 |  |
| 1374712_at | Psmd11 | Ubiquitin cycle | 5250 | 2815 | * |
| 1398858_at | Psmd2 | Ubiquitin cycle | 4343 | 4586 |  |
| 1371851_at | Psmd6 | Ubiquitin cycle | 3443 | 4077 | * |
| 1389519_at | Psmd8 | Ubiquitin cycle | 4319 | 3190 | * |
| 1367663_at | Psme1 | Ubiquitin cycle | 8429 | 7579 |  |
| 1367710_at | Psme2 | Ubiquitin cycle | 3455 | 2378 | * |
| 1368817_at | Psme4 | Ubiquitin cycle | 2112 | 3242 | * |
| 1387345_at | Psme4 | Ubiquitin cycle | 1541 | 3189 | * |
| 1388333_at | Rbx1 | Ubiquitin cycle | 9149 | 5373 | * |
| 1371638_at | Rnf7 | Ubiquitin cycle | 4848 | 3833 |  |
| 1372437_at | Skp1 | Ubiquitin cycle | 9121 | 7517 | * |
| 1398910_at | Stub1 | Ubiquitin cycle | 4530 | 2742 | * |
| 1367472_at | Uba1 | Ubiquitin cycle | 3587 | 6101 | * |
| 1371657_at | Uba2 | Ubiquitin cycle | 2748 | 3119 |  |
| 1398813_at | Uba3 | Ubiquitin cycle | 2625 | 3101 |  |
| 1370274_at, 1386852_x_at | Ubb | Ubiquitin cycle | 15692, 14812 | 17403, 17437 |  |
| 1398767_at | Ubc | Ubiquitin cycle | 9457 | 13352 | * |
| 1386923_at | Ube2b | Ubiquitin cycle | 5060 | 3806 | * |
| 1370523_a_at, 1370524_at | Ube2d2 | Ubiquitin cycle | 3932, 4318 | 2696, 3469 | * |
| 1367456_at, 1398802_at, 1399160_a_at | Ube2d3 | Ubiquitin cycle | 8140, 3881, 5820 | 7636, 3618, 5581 |  |
| 1367484_at | Ube2e2 | Ubiquitin cycle | 3115 | 1970 | * |
| 1389534_at | Ube2e3 | Ubiquitin cycle | 5522 | 3408 | * |
| 1398864_at | Ube2g1 | Ubiquitin cycle | 4480 | 2715 | * |
| 1370250_at | Ube2i | Ubiquitin cycle | 4878 | 2938 | * |
| 1371764_at | Ube2j2 | Ubiquitin cycle | 3449 | 2383 | * |
| 1371944_at | Ube2l3 | Ubiquitin cycle | 4970 | 4068 | * |
| 1388559_at | Ube2m | Ubiquitin cycle | 3435 | 1748 | * |
| 1371365_at | Ube2s | Ubiquitin cycle | 3661 | 2071 | * |
| 1398897_at | Ube2v1 | Ubiquitin cycle | 3759 | 2199 | * |
| 1371589_at, 1371590_s_at | Ubl5 | Ubiquitin cycle | 4728, 6942 | 2698, 4328 | * |
| 1367719_at | Ubr5 | Ubiquitin cycle | 1880 | 3148 | * |
| 1373299_at | Ubxn1 | Ubiquitin cycle | 5414 | 2558 | * |
| 1388444_at | Ubxn4 | Ubiquitin cycle | 2630 | 2997 |  |
| 1371758_at | Ufc1 | Ubiquitin cycle | 4593 | 3083 | * |
| 1383073_at | Usp14 | Ubiquitin cycle | 2692 | 3644 | * |
| 1398424_at | Wsb2 | Ubiquitin cycle | 4124 | 3678 |  |
| --- | --- | --- | --- | --- |  |
| **Protein synthesis** | | | --- | --- |  |
| 1371378_at, 1388310_at | Eif1 | Initiation of translation | 7507, 12746 | 6332, 11665 |  |
| 1372331_at | Eif1ay | Initiation of translation | 4084 | 3302 | * |
| 1367713_at | Eif2s1 | Initiation of translation | 3241 | 3565 |  |
| 1374397_at | Eif2s2 | Initiation of translation | 5946 | 6208 |  |
| 1388378_at | Eif3c | Initiation of translation | 3868 | 6036 | * |
| 1388568_at | Eif3d | Initiation of translation | 3820 | 3669 |  |
| 1371973_at | Eif3e | Initiation of translation | 5383 | 8256 | * |
| 1373040_at | Eif3f | Initiation of translation | 5967 | 7439 | * |
| 1388381_at | Eif3g | Initiation of translation | 6075 | 5222 | * |
| 1388390_at | Eif3h | Initiation of translation | 5844 | 6872 | * |
| 1388328_at | Eif3i | Initiation of translation | 5230 | 3987 | * |
| 1388424_at | Eif3j | Initiation of translation | 3170 | 3246 |  |
| 1373044_at | Eif3m | Initiation of translation | 6166 | 7853 | * |
| 1389968_at | Eif3s10 | Initiation of translation | 1382 | 3581 | * |
| 1398929_at | Eif3s6ip | Initiation of translation | 3519 | 4425 | * |
| 1371936_at | Eif4a1 | Initiation of translation | 8260 | 7716 |  |
| 1371642_at | Eif4a2 | Initiation of translation | 4784 | 6690 | * |
| 1367480_at | Eif4a3 | Initiation of translation | 3514 | 3545 |  |
| 1371404_at | Eif4b | Initiation of translation | 3051 | 3808 | * |
| 1398799_at | Eif4e | Initiation of translation | 4096 | 3295 | * |
| 1386888_at | Eif4ebp1 | Initiation of translation | 7083 | 3549 | * |
| 1371448_at | Eif4ebp2 | Initiation of translation | 3398 | 3083 |  |
| 1367469_at, 1397520_at | Eif4g2 | Initiation of translation | 10387, 3630 | 11703, 5063 | * |
| 1367531_at | Eif4h | Initiation of translation | 8458 | 6472 | * |
| 1398845_at, 1398846_at | Eif5 | Initiation of translation | 3285, 5655 | 3978, 4443 |  |
| 1371329_at | Eif5a | Initiation of translation | 11144 | 7780 | * |
| 1388507_at | Eif6 | Initiation of translation | 4485 | 3226 | * |
| 1369952_at | Pabpc1 | Initiation of translation | 9309 | 18453 |  |
| 1371777_at | Pabpc4 | Initiation of translation | 5284 | 8808 | * |
| 1398891_at | Mrpl15 | Mitochondrial ribosome | 4674 | 4061 |  |
| 1369013_a_at | Mrpl17 | Mitochondrial ribosome | 7125 | 4207 | * |
| 1399058_at | Mrpl18 | Mitochondrial ribosome | 4184 | 2758 | * |
| 1371720_at | Mrpl20 | Mitochondrial ribosome | 3729 | 2447 | * |
| 1368004_at | Mrpl23 | Mitochondrial ribosome | 4312 | 2734 | * |
| 1399047_at | Mrpl27 | Mitochondrial ribosome | 3900 | 2204 | * |
| 1373705_at | Mrpl28 | Mitochondrial ribosome | 3489 | 1979 | * |
| 1371709_at | Mrpl3 | Mitochondrial ribosome | 3077 | 2954 |  |
| 1371512_at | Mrpl33 | Mitochondrial ribosome | 4246 | 3441 | * |
| 1371553_at | Mrpl36 | Mitochondrial ribosome | 3198 | 1772 | * |
| 1371423_at | Mrpl41 | Mitochondrial ribosome | 4491 | 3060 | * |
| 1367546_at | Mrpl43 | Mitochondrial ribosome | 3753 | 2602 | * |
| 1367477_at | Mrpl53 | Mitochondrial ribosome | 3563 | 2637 | * |
| 1388853_at | Mrpl54 | Mitochondrial ribosome | 3761 | 2248 | * |
| 1383090_at | Mrps14 | Mitochondrial ribosome | 3667 | 2136 | * |
| 1399002_at | Mrps17 | Mitochondrial ribosome | 5520 | 3619 | * |
| 1371661_at | Mrps23 | Mitochondrial ribosome | 3427 | 2226 | * |
| 1371649_at | Mrps24 | Mitochondrial ribosome | 3220 | 1690 | * |
| 1371576_at | Mrps36 | Mitochondrial ribosome | 4147 | 3639 |  |
| 1389110_at | Mrps6 | Mitochondrial ribosome | 4661 | 2668 | * |
| 1372431_at | Mrpl12 | Mitochondrial ribosome | 6014 | 3137 | * |
| 1370109_s_at, 1388110_at | Eef1a1 | Peptide elongation | 16582, 16076 | 21116, 21373 | * |
| 1367799_at | Eef1a2 | Peptide elongation | 2733 | 4013 | * |
| 1388449_at | Eef1b2 | Peptide elongation | 10253 | 13607 | * |
| 1388134_at | Eef1d | Peptide elongation | 5099 | 4554 | * |
| 1388297_at | Eef1g | Peptide elongation | 12232 | 17681 | * |
| 1370003_at | Eef2 | Peptide elongation | 11443 | 14766 | * |
| 1371515_at | Elof1 | Peptide elongation | 3748 | 2349 | * |
| 1371435_at | Naca | Peptide elongation | 11006 | 11046 |  |
| 1367476_at | Srp14 | Peptide elongation | 7466 | 4654 | * |
| 1372445_at | Srp9 | Peptide elongation | 3412 | 2161 | * |
| 1367560_at | Arbp | Ribosome | 12115 | 20682 | * |
| 1371316_at | Fau | Ribosome | 10982 | 13277 |  |
| 1367625_at | Rpl10 | Ribosome | 14614 | 18176 | * |
| 1367580_at | Rpl10a | Ribosome | 10458 | 13265 |  |
| 1371330_at | Rpl11 | Ribosome | 13491 | 15484 | * |
| 1375181_at | Rpl12 | Ribosome | 12107 | 16780 | * |
| 1386858_at | Rpl13 | Ribosome | 11354 | 14268 | * |
| 1367588_a_at | Rpl13a | Ribosome | 12994 | 16082 | * |
| 1387887_at | Rpl14 | Ribosome | 10911 | 13678 | * |
| 1398315_at | Rpl15 | Ribosome | 12489 | 14922 | * |
| 1398871_at | Rpl17 | Ribosome | 11968 | 15219 | * |
| 1367623_at | Rpl18 | Ribosome | 14252 | 15067 |  |
| 1398324_at | Rpl18a | Ribosome | 14800 | 18894 |  |
| 1367610_at | Rpl19 | Ribosome | 15574 | 18163 |  |
| 1398764_at | Rpl21 | Ribosome | 9027 | 13042 | * |
| 1370253_at | Rpl22 | Ribosome | 13987 | 15132 |  |
| 1388989_at | Rpl22l1 | Ribosome | 6528 | 8488 | * |
| 1398885_at | Rpl23 | Ribosome | 14730 | 17305 | * |
| 1371313_at | Rpl23a | Ribosome | 10469 | 13049 | * |
| 1398854_at | Rpl24 | Ribosome | 9492 | 13170 | * |
| 1388303_at | Rpl26 | Ribosome | 15973 | 20713 | * |
| 1367561_at | Rpl27 | Ribosome | 14914 | 17966 |  |
| 1371344_at | Rpl27a | Ribosome | 17194 | 21055 | * |
| 1398830_at | Rpl28 | Ribosome | 14564 | 16016 |  |
| 1367582_at | Rpl29 | Ribosome | 10887 | 13332 |  |
| 1371300_at | Rpl3 | Ribosome | 17499 | 22331 | * |
| 1398774_at | Rpl30 | Ribosome | 13966 | 16882 | * |
| 1367634_at | Rpl31 | Ribosome | 15859 | 19904 | * |
| 1384548_at | Rpl32 | Ribosome | 15481 | 19917 | * |
| 1371761_at | Rpl34 | Ribosome | 13240 | 16068 | * |
| 1388372_at | Rpl35 | Ribosome | 11004 | 13216 | * |
| 1398760_at | Rpl35a | Ribosome | 10750 | 14919 | * |
| 1371573_at | Rpl36a | Ribosome | 7985 | 11013 | * |
| 1398770_at | Rpl36al | Ribosome | 8525 | 7595 |  |
| 1398789_at | Rpl37 | Ribosome | 13068 | 17387 | * |
| 1371504_at | Rpl37a | Ribosome | 10194 | 15019 |  |
| 1375632_at | Rpl38 | Ribosome | 14067 | 14602 |  |
| 1398749_at | Rpl4 | Ribosome | 10465 | 14655 | * |
| 1370866_at | Rpl41 | Ribosome | 11709 | 14986 | * |
| 1398761_at | Rpl5 | Ribosome | 12941 | 18018 | * |
| 1367567_at | Rpl6 | Ribosome | 15034 | 18753 | * |
| 1398917_at | Rpl7 | Ribosome | 13696 | 18289 | * |
| 1371297_at | Rpl7a | Ribosome | 17027 | 20695 |  |
| 1371305_at, 1384714_x_at | Rpl8 | Ribosome | 12381, 2311 | 14361, 2965 |  |
| 1371301_at | Rpl9 | Ribosome | 16687 | 22100 | * |
| 1371307_at | Rplp1 | Ribosome | 13493 | 19823 | * |
| 1371340_at | Rplp2 | Ribosome | 12373 | 14524 |  |
| 1386868_at | Rps10 | Ribosome | 10700 | 11394 |  |
| 1367630_at | Rps11 | Ribosome | 12567 | 14959 | * |
| 1367640_at | Rps12 | Ribosome | 14107 | 19633 | * |
| 1398872_at | Rps13 | Ribosome | 12407 | 14348 | * |
| 1368211_at | Rps14 | Ribosome | 15262 | 19724 | * |
| 1386874_at | Rps15 | Ribosome | 10445 | 13062 |  |
| 1398775_at | Rps15a | Ribosome | 15245 | 19085 | * |
| 1371318_at | Rps16 | Ribosome | 12846 | 16946 | * |
| 1367645_at | Rps17 | Ribosome | 17319 | 20586 | * |
| 1388296_at | Rps18 | Ribosome | 14887 | 18119 |  |
| 1371377_at | Rps19 | Ribosome | 13953 | 19232 | * |
| 1367639_a_at, 1375219_a_at | Rps2 | Ribosome | 14012, 17528 | 20145, 22481 | * |
| 1371295_at | Rps20 | Ribosome | 12201 | 16102 | * |
| 1398852_at | Rps21 | Ribosome | 15037 | 17747 |  |
| 1370242_at | Rps23 | Ribosome | 19085 | 22493 |  |
| 1369966_a_at | Rps24 | Ribosome | 13412 | 17064 | * |
| 1388313_at | Rps25 | Ribosome | 5090 | 6886 | * |
| 1367596_at | Rps26 | Ribosome | 12954 | 16664 | * |
| 1367717_at | Rps27 | Ribosome | 15952 | 22438 | * |
| 1367685_at | Rps27a | Ribosome | 13170 | 16492 | * |
| 1388481_at | Rps28 | Ribosome | 13827 | 16273 |  |
| 1387890_at | Rps29 | Ribosome | 16446 | 21694 | * |
| 1371299_at | Rps3 | Ribosome | 12239 | 14579 |  |
| 1367606_at | Rps3a | Ribosome | 15550 | 21015 | * |
| 1371308_at | Rps4x | Ribosome | 14850 | 20060 | * |
| 1398882_at | Rps5 | Ribosome | 13544 | 17809 | * |
| 1367573_at | Rps6 | Ribosome | 16339 | 20516 | * |
| 1398751_at | Rps7 | Ribosome | 10647 | 15663 | * |
| 1367597_at | Rps8 | Ribosome | 17027 | 20077 |  |
| 1387888_at | Rps9 | Ribosome | 13057 | 13949 |  |
| 1367569_at, 1371189_x_at, 1388244_s_at | Rpsa | Ribosome | 9913, 2677, 9497 | 14337, 4134, 12951 | * |
| 1371964_at | Grsf1 | RNA recruitment | 5146 | 4751 |  |
| 1388870_at | Msi2 | RNA recruitment | 3459 | 2691 |  |
| 1374385_at | Pcbp1 | RNA recruitment | 8117 | 7021 |  |
| 1398941_at | Pcbp2 | RNA recruitment | 7250 | 6848 |  |
| 1371583_at | Rbm3 | RNA recruitment | 11634 | 11865 |  |
| 1371585_at | Gspt1 | Termination of translation | 2555 | 3586 | * |
| 1389038_at | Etf1 | Termination of translation | 3604 | 2809 | * |
| 1373013_at, 1399026_at | Paip2 | Translational repressor | 6384, 4048 | 5329, 3161 | * |
| 1388394_at | Aars | tRNA | 2470 | 3826 | * |
| 1373418_at, 1383455_at | Eprs | tRNA | 1773, 1814 | 3475, 4049 | * |
| 1388715_at | Gars | tRNA | 4273 | 4493 |  |
| 1374437_at | Nars | tRNA | 3472 | 4324 | * |
| 1371930_at | Qars | tRNA | 2326 | 3021 | * |
| 1388376_at | Trmt112 | tRNA | 5237 | 3005 | * |
| 1367686_at | Serp1 | Unfolded protein response | 6689 | 4883 | * |
| --- | --- | --- | --- | --- |  |
| **Regulation of metabolism and redox** | | | --- | --- |  |
| 1367819_at | Got2 | Amino acid metabolism | 5467 | 7763 | * |
| 1377761_at | Gfpt2 | Carbohydrate metabolism | 3707 | 4599 |  |
| 1399098_at | Glo1 | Carbohydrate metabolism | 4984 | 5118 |  |
| 1398903_at | Esd | Carboxylesterase activity | 3625 | 4002 |  |
| 1367667_at | Fdps | Cholesterol metabolism | 9694 | 9972 |  |
| 1367735_at | Acadl | Fatty acid metabolism | 3984 | 5396 | * |
| 1367702_at | Acadm | Fatty acid metabolism | 2397 | 3278 |  |
| 1387101_at | Acsl4 | Fatty acid metabolism | 1688 | 3052 | * |
| 1367742_at | Cpt1b | Fatty acid metabolism | 2144 | 3543 | * |
| 1370808_at | Cyb5r3 | Fatty acid metabolism | 3586 | 3397 |  |
| 1370235_at | Dbi | Fatty acid metabolism | 7163 | 5437 | * |
| 1367659_s_at | Dci | Fatty acid metabolism | 3586 | 2768 | * |
| 1367770_at | Degs1 | Fatty acid metabolism | 2883 | 3501 |  |
| 1388348_at | Elovl5 | Fatty acid metabolism | 3034 | 4049 | * |
| 1367660_at | Fabp3 | Fatty acid metabolism | 13475 | 12886 |  |
| 1370281_at | Fabp5 | Fatty acid metabolism | 12450 | 8563 | * |
| 1371358_at | Gpsn2 | Fatty acid metabolism | 3022 | 3285 |  |
| 1370164_at | Hadha | Fatty acid metabolism | 1071 | 3244 | * |
| 1367694_at | Hadhb | Fatty acid metabolism | 2820 | 6442 | * |
| 1388118_at | Hibadh | Fatty acid metabolism | 5614 | 4137 | * |
| 1369954_at | Idh1 | Fatty acid metabolism | 3016 | 3407 |  |
| 1386965_at | Lpl | Fatty acid metabolism | 3387 | 6144 | * |
| 1367832_at | Lypla1 | Fatty acid metabolism | 4465 | 3693 | * |
| 1371421_at | Oxct1 | Fatty acid metabolism | 3072 | 5989 | * |
| 1368128_at | Pla2g2a | Fatty acid metabolism | 8018 | 6629 |  |
| 1371372_at | Ptges3 | Fatty acid metabolism | 7751 | 7366 |  |
| 1367668_a_at | Scd | Fatty acid metabolism | 6186 | 7987 |  |
| 1389265_at | Gbe1 | Glycogen metabolism | 2734 | 3258 |  |
| 1367900_at | Gyg1 | Glycogen metabolism | 10942 | 13203 | * |
| 1371625_at | Pygb | Glycogen metabolism | 2017 | 3267 | * |
| 1388410_at | Ugp2 | Glycogen metabolism | 4287 | 4205 |  |
| 1367617_at | Aldoa | Glycolysis | 17645 | 19904 |  |
| 1367575_at | Eno1 | Glycolysis | 14842 | 18157 | * |
| 1370341_at | Eno2 | Glycolysis | 4527 | 3172 |  |
| AFFX_Rat_GAPDH_3/5/M_at, 1367557_s_at | Gapdh | Glycolysis | 17475, 15298, 14528, 18239 | 20609, 19860, 17963, 22428 | * |
| 1371392_at | Gpi | Glycolysis | 8065 | 10218 |  |
| 1386929_at | Hk1 | Glycolysis | 2638 | 4475 | * |
| 1367586_at | Ldha | Glycolysis | 13796 | 16288 | * |
| 1370218_at | Ldhb | Glycolysis | 11989 | 15204 | * |
| 1372182_at | Pfkp | Glycolysis | 2552 | 4966 | * |
| 1386864_at | Pgam1 | Glycolysis | 15242 | 14520 |  |
| 1387361_s_at, 1388318_at | Pgk1 | Glycolysis | 10108, 15000 | 13939, 17148 | * |
| 1388634_at | Pgm1 | Glycolysis | 4341 | 4915 |  |
| 1369931_at | Pkm2 | Glycolysis | 10439 | 13505 | * |
| 1367603_at | Tpi1 | Glycolysis | 12984 | 12330 |  |
| 1374537_at | Chsy1 | Glycosaminoglycan/extracellular matrix metabolism | 2669 | 3298 |  |
| 1371894_at | Gns | Glycosaminoglycan/extracellular matrix metabolism | 2402 | 3057 | * |
| 1375843_at | Ids | Glycosaminoglycan/extracellular matrix metabolism | 3088 | 3649 |  |
| 1367938_at | Ugdh | Glycosaminoglycan/extracellular matrix metabolism | 4203 | 4723 |  |
| 1373367_at | Impad1 | Inositol metabolism | 3824 | 3499 |  |
| 1388697_at | Inpp5a | Inositol metabolism | 3090 | 2772 |  |
| 1367653_a_at | Mdh1 | Malate-aspartate shuttle | 10988 | 13718 | * |
| 1367793_at | Ddt | Melanin metabolism | 4144 | 2775 | * |
| 1371237_a_at | Mt1a | Metal ion binding | 14025 | 10917 |  |
| 1388271_at | Mt2A | Metal ion binding | 10232 | 7586 |  |
| 1372438_at | Nit2 | Nitrogen metabolism | 3660 | 3542 |  |
| 1389185_at | Ak1 | Nucleoside/nucleotide metabolism | 5143 | 3170 | * |
| 1398349_at | Ak2 | Nucleoside/nucleotide metabolism | 3767 | 2313 | * |
| 1368095_at, 1388558_at | Ak3 | Nucleoside/nucleotide metabolism | 3426, 7156 | 1992, 5583 | * |
| 1371244_at | Aprt | Nucleoside/nucleotide metabolism | 10589 | 7423 | * |
| 1369588_a_at | Atpif1 | Nucleoside/nucleotide metabolism | 8163 | 5326 | * |
| 1367626_at | Ckm | Nucleoside/nucleotide metabolism | 3686 | 5255 | * |
| 1373365_at | Cmpk1 | Nucleoside/nucleotide metabolism | 5330 | 4126 |  |
| 1371694_at | Dpysl2 | Nucleoside/nucleotide metabolism | 5389 | 6459 |  |
| 1388479_at | Dpysl3 | Nucleoside/nucleotide metabolism | 4851 | 4714 |  |
| 1367905_at | Enpp3 | Nucleoside/nucleotide metabolism | 2668 | 6089 | * |
| 1387659_at | Gda | Nucleoside/nucleotide metabolism | 4017 | 2452 | * |
| 1371743_at | Guk1 | Nucleoside/nucleotide metabolism | 5216 | 2912 | * |
| 1370280_at | Hprt1 | Nucleoside/nucleotide metabolism | 5279 | 4562 |  |
| 1388629_at | Impdh2 | Nucleoside/nucleotide metabolism | 4092 | 5456 | * |
| 1371350_at | Mat2a | Nucleoside/nucleotide metabolism | 1928 | 3333 | * |
| 1370295_at | Nme1 | Nucleoside/nucleotide metabolism | 3202 | 1691 | * |
| 1367766_at | Nme2 | Nucleoside/nucleotide metabolism | 14987 | 12689 | * |
| 1388337_at | Np | Nucleoside/nucleotide metabolism | 4479 | 3208 | * |
| 1370180_at, 1398847_at | Nudt4 | Nucleoside/nucleotide metabolism | 6979, 5749 | 5466, 4085 | * |
| 1388645_at | Ola1 | Nucleoside/nucleotide metabolism | 3560 | 3639 |  |
| 1387790_at | Paics | Nucleoside/nucleotide metabolism | 5009 | 4975 |  |
| 1374784_at | Prtfdc1 | Nucleoside/nucleotide metabolism | 3961 | 2869 | * |
| 1371724_at | Rexo2 | Nucleoside/nucleotide metabolism | 3762 | 1882 | * |
| 1388327_at | Cisd1 | OxPhos/electron transport | 10898 | 7633 | * |
| 1388570_at | Coq9 | OxPhos/electron transport | 3927 | 2868 | * |
| 1389956_a_at | COX1 | OxPhos/electron transport | 16523 | 21712 | * |
| 1375107_at | COX2 | OxPhos/electron transport | 12440 | 23518 | * |
| 1367607_at | Cox4i1 | OxPhos/electron transport | 15277 | 17011 |  |
| 1370888_at | Cox5a | OxPhos/electron transport | 15712 | 16394 |  |
| 1386887_at | Cox5b | OxPhos/electron transport | 14122 | 13269 |  |
| 1370861_at | Cox6a1 | OxPhos/electron transport | 11557 | 10780 |  |
| 1367782_at | Cox6a2 | OxPhos/electron transport | 13214 | 13151 |  |
| 1371321_at | Cox6b1 | OxPhos/electron transport | 10187 | 9780 |  |
| 1367757_at | Cox6c | OxPhos/electron transport | 17266 | 17738 |  |
| 1367629_at | Cox7a2 | OxPhos/electron transport | 17486 | 19037 |  |
| 1371337_at | Cox7a2l | OxPhos/electron transport | 6761 | 6249 |  |
| 1371387_at | Cox7b | OxPhos/electron transport | 12775 | 13347 |  |
| 1389820_at | Cox7c | OxPhos/electron transport | 11749 | 11498 |  |
| 1388113_at | Cox8a | OxPhos/electron transport | 15345 | 16759 | * |
| 1367739_at | Cox8b | OxPhos/electron transport | 3674 | 3634 |  |
| 1371342_at | Cyc1 | OxPhos/electron transport | 12735 | 9641 | * |
| 1369939_at, 1387773_at | Cycs | OxPhos/electron transport | 14033, 11054 | 14618, 11213 |  |
| 1388159_at | CYTB | OxPhos/electron transport | 14237 | 20459 | * |
| 1371253_at | Etfa | OxPhos/electron transport | 4089 | 3992 |  |
| 1388358_at | Etfb | OxPhos/electron transport | 5029 | 3864 |  |
| 1368336_at | Fdx1 | OxPhos/electron transport | 3720 | 2354 | * |
| 1388603_a_at | Isca1 | OxPhos/electron transport | 5433 | 3721 | * |
| 1367467_at | Iscu | OxPhos/electron transport | 4846 | 3039 | * |
| 1375651_at | Nd1 | OxPhos/electron transport | 11814 | 20840 | * |
| 1375340_at | Nd2 | OxPhos/electron transport | 12926 | 25920 | * |
| 1375108_at | Nd3 | OxPhos/electron transport | 10989 | 18625 | * |
| 1388391_at | Ndufa1 | OxPhos/electron transport | 13879 | 11543 | * |
| 1375220_at | Ndufa11 | OxPhos/electron transport | 8336 | 7309 |  |
| 1371605_at | Ndufa12 | OxPhos/electron transport | 9837 | 7326 | * |
| 1388362_at | Ndufa13 | OxPhos/electron transport | 8357 | 6927 | * |
| 1389288_at | Ndufa2 | OxPhos/electron transport | 5084 | 3217 | * |
| 1388489_at | Ndufa3 | OxPhos/electron transport | 5667 | 4103 | * |
| 1371323_at | Ndufa4 | OxPhos/electron transport | 12561 | 12551 |  |
| 1379243_at | Ndufa6 | OxPhos/electron transport | 7321 | 6141 | * |
| 1375411_at | Ndufa7 | OxPhos/electron transport | 7750 | 4966 | * |
| 1371355_at | Ndufa8 | OxPhos/electron transport | 6838 | 4929 | * |
| 1388323_at | Ndufa9 | OxPhos/electron transport | 5089 | 5567 |  |
| 1389964_at | Ndufab1 | OxPhos/electron transport | 8553 | 7681 |  |
| 1388361_at | Ndufb10 | OxPhos/electron transport | 5804 | 4062 | * |
| 1388315_at | Ndufb11 | OxPhos/electron transport | 9943 | 9184 |  |
| 1389012_at | Ndufb2 | OxPhos/electron transport | 10813 | 7956 | * |
| 1373041_at | Ndufb3 | OxPhos/electron transport | 9364 | 6448 | * |
| 1371371_at | Ndufb4 | OxPhos/electron transport | 10385 | 10552 |  |
| 1388304_at | Ndufb5 | OxPhos/electron transport | 11050 | 9309 | * |
| 1371346_at | Ndufb6 | OxPhos/electron transport | 6963 | 5436 | * |
| 1388343_at | Ndufb7 | OxPhos/electron transport | 7571 | 4996 | * |
| 1371701_at | Ndufb9 | OxPhos/electron transport | 15672 | 16813 |  |
| 1392530_at | Ndufc1 | OxPhos/electron transport | 6638 | 4706 | * |
| 1375516_at | Ndufc2 | OxPhos/electron transport | 7700 | 6586 |  |
| 1371577_at | Ndufs1 | OxPhos/electron transport | 1414 | 4298 | * |
| 1371482_at | Ndufs2 | OxPhos/electron transport | 3492 | 5351 | * |
| 1388364_at | Ndufs3 | OxPhos/electron transport | 4529 | 3687 | * |
| 1372147_at | Ndufs4 | OxPhos/electron transport | 6257 | 2121 | * |
| 1388414_at | Ndufs5 | OxPhos/electron transport | 11069 | 8759 | * |
| 1370006_at | Ndufs6 | OxPhos/electron transport | 9830 | 7507 | * |
| 1371912_at | Ndufs7 | OxPhos/electron transport | 5397 | 3354 | * |
| 1388326_at | Ndufs8 | OxPhos/electron transport | 3851 | 2590 | * |
| 1371416_at | Ndufv1 | OxPhos/electron transport | 4385 | 5437 | * |
| 1371041_at | Ndufv2 | OxPhos/electron transport | 7410 | 7293 |  |
| 1368566_a_at | Ndufv3 | OxPhos/electron transport | 3939 | 2847 | * |
| 1371483_at | Nnt | OxPhos/electron transport | 2348 | 9424 | * |
| 1372291_at | Oxa1l | OxPhos/electron transport | 2183 | 3514 | * |
| 1375197_at | Uqcr | OxPhos/electron transport | 11350 | 8562 | * |
| 1374426_at | Uqcrb | OxPhos/electron transport | 12635 | 12003 |  |
| 1388301_at | Uqcrc1 | OxPhos/electron transport | 7028 | 8292 |  |
| 1373362_at | Uqcrc2 | OxPhos/electron transport | 4847 | 10175 | * |
| 1371254_at | Uqcrfs1 | OxPhos/electron transport | 11779 | 12606 |  |
| 1371415_at | Uqcrh | OxPhos/electron transport | 13234 | 14010 |  |
| 1371417_at | Uqcrq | OxPhos/electron transport | 9190 | 8005 |  |
| 1370239_at, 1370240_x_at, 1388608_x_at | Hba-a2 | Oxygen transport | 4706, 4242, 3175 | 10725, 10236, 5159 | * |
| 1367553_x_at | Hbb | Oxygen transport | 2152 | 5010 | * |
| 1387768_at | Mb | Oxygen transport | 3879 | 3388 |  |
| 1367856_at | G6pd | Pentose-phosphate shunt | 3064 | 2904 |  |
| 1386859_at | Tkt | Pentose-phosphate shunt | 3952 | 3196 |  |
| 1370191_at, 1370575_a_at | Azin1 | Polyamine metabolism | 4827, 5441 | 4403, 5662 |  |
| 1372241_at | Oaz1 | Polyamine metabolism | 16496 | 15451 |  |
| 1388454_at | Oaz2 | Polyamine metabolism | 4312 | 2938 | * |
| 1370163_at | Odc1 | Polyamine metabolism | 3774 | 2216 | * |
| 1371774_at | Sat1 | Polyamine metabolism | 5125 | 3725 | * |
| 1398753_at | Akr1a1 | Redox/detoxification | 14169 | 10161 | * |
| 1367734_at | Akr1b1 | Redox/detoxification | 5310 | 4570 |  |
| 1371567_at | Aldh7a1 | Redox/detoxification | 2187 | 3106 | * |
| 1367870_at | Glrx3 | Redox/detoxification | 4113 | 3252 | * |
| 1389286_at | Glrx5 | Redox/detoxification | 6244 | 3575 | * |
| 1367576_at | Gpx1 | Redox/detoxification | 7841 | 7130 |  |
| 1369926_at | Gpx3 | Redox/detoxification | 16278 | 11993 | * |
| 1386871_at | Gpx4 | Redox/detoxification | 10871 | 8865 |  |
| 1367774_at | Gsta3 | Redox/detoxification | 4674 | 3451 | * |
| 1372297_at | Gsta4 | Redox/detoxification | 3703 | 3127 |  |
| 1389832_at | Gsto1 | Redox/detoxification | 6641 | 4238 | * |
| 1388122_at | Gstp1 | Redox/detoxification | 12622 | 11803 |  |
| 1370080_at | Hmox1 | Redox/detoxification | 6662 | 5434 |  |
| 1367612_at | Mgst1 | Redox/detoxification | 7922 | 6722 |  |
| 1388300_at | Mgst3 | Redox/detoxification | 3885 | 4303 |  |
| 1368653_a_at | Park7 | Redox/detoxification | 5281 | 4249 | * |
| 1367613_at | Prdx1 | Redox/detoxification | 12677 | 10782 | * |
| 1367578_at | Prdx2 | Redox/detoxification | 12458 | 11053 |  |
| 1367591_at | Prdx3 | Redox/detoxification | 4232 | 3355 | * |
| 1387891_at | Prdx4 | Redox/detoxification | 4044 | 4099 |  |
| 1367677_at | Prdx5 | Redox/detoxification | 7795 | 4496 | * |
| 1367969_at | Prdx6 | Redox/detoxification | 4361 | 3025 | * |
| 1372294_at | Pxdn | Redox/detoxification | 1703 | 3951 | * |
| 1399004_at | Romo1 | Redox/detoxification | 4270 | 2371 | * |
| 1371783_at | Selk | Redox/detoxification | 4675 | 3379 |  |
| 1388705_at | Selm | Redox/detoxification | 4302 | 2036 | * |
| 1387915_at | Sels | Redox/detoxification | 4828 | 3222 | * |
| 1398964_at | Selt | Redox/detoxification | 4481 | 3381 | * |
| 1398752_at | Sep15 | Redox/detoxification | 6995 | 5639 |  |
| 1389395_at | Sepn1 | Redox/detoxification | 3265 | 2766 |  |
| 1368806_at | Sepp1 | Redox/detoxification | 5734 | 4658 | * |
| 1371487_at | Sh3bgrl3 | Redox/detoxification | 5779 | 2634 | * |
| 1367641_at | Sod1 | Redox/detoxification | 10885 | 8383 | * |
| 1370172_at | Sod2 | Redox/detoxification | 10789 | 8392 |  |
| 1398839_at | Txn1 | Redox/detoxification | 15130 | 13036 |  |
| 1398844_at | Txn2 | Redox/detoxification | 6399 | 3183 | * |
| 1375221_at | Txndc13 | Redox/detoxification | 2390 | 4219 | * |
| 1368936_at, 1380191_s_at | Txnl1 | Redox/detoxification | 3526, 6101 | 3609, 5457 |  |
| 1371432_at | Vat1 | Redox/detoxification | 4341 | 3103 | * |
| 1388330_at | Vkorc1 | Redox/detoxification | 4973 | 3071 | * |
| 1369929_at | Psap | Sphingolipid metabolism | 3797 | 5704 | * |
| 1388552_at | Smpd1 | Sphingolipid metabolism | 3249 | 3015 |  |
| 1368990_at | Cyp1b1 | Steroid metabolism | 5597 | 6989 | * |
| 1367979_s_at | Cyp51 | Steroid metabolism | 4109 | 5176 |  |
| 1367932_at | Hmgcs1 | Steroid metabolism | 4367 | 7375 | * |
| 1386953_at | Hsd11b1 | Steroid metabolism | 6801 | 6863 |  |
| 1367662_at | Hsd17b10 | Steroid metabolism | 4209 | 3200 | * |
| 1367894_at | Iig1 | Steroid metabolism | 5919 | 4726 | * |
| 1368275_at | Sc4mol | Steroid metabolism | 4908 | 4908 |  |
| 1370249_at | Tspo | Steroid metabolism | 7733 | 5406 | * |
| 1367854_at | Acly | TCA cycle | 1854 | 3354 | * |
| 1367589_at | Aco2 | TCA cycle | 3963 | 10335 | * |
| 1375295_at | Cs | TCA cycle | 3637 | 6840 | * |
| 1373016_at | Dld | TCA cycle | 2397 | 4786 | * |
| 1370879_at | Dlst | TCA cycle | 2788 | 3269 |  |
| 1367670_at | Fh1 | TCA cycle | 2262 | 4335 | * |
| 1367737_at | Fuca1 | TCA cycle | 3501 | 4416 |  |
| 1388403_at | Idh2 | TCA cycle | 11430 | 12128 |  |
| 1388160_a_at | Idh3B | TCA cycle | 3789 | 3936 |  |
| 1370865_at | Idh3g | TCA cycle | 3503 | 4470 | * |
| 1369927_at | Mdh2 | TCA cycle | 6387 | 7443 |  |
| 1390020_at | Ogdh | TCA cycle | 2011 | 5204 | * |
| 1371380_at, 1383698_at | Pdha1 | TCA cycle | 5342, 1135 | 8011, 3594 | * |
| 1371388_at | Pdhb | TCA cycle | 4588 | 5655 | * |
| 1367678_at | Sdha | TCA cycle | 2663 | 7511 | * |
| 1371488_at | Sdhaf1 | TCA cycle | 3466 | 2190 | * |
| 1372123_at | Sdhb | TCA cycle | 8183 | 7807 |  |
| 1371311_at | Sdhc | TCA cycle | 9153 | 7051 | * |
| 1371296_at, 1388294_at | Sdhd | TCA cycle | 7165, 10593 | 5758, 8793 | * |
| 1388975_at | Sucla2 | TCA cycle | 2875 | 4323 | * |
| 1367642_at | Suclg1 | TCA cycle | 4349 | 3427 | * |
| 1373017_at | Suclg2 | TCA cycle | 3028 | 2848 |  |
| 1379330_s_at | Pcbd2 | Tetrahydrobiopterin metabolism | 4015 | 2626 | * |
| --- | --- | --- | --- | --- |  |
| **Trafficking** | | | --- | --- |  |
| 1371514_at | Kdelr1 | ER retention | 5651 | 4862 | * |
| 1371780_at | Kdelr2 | ER retention | 5557 | 5240 |  |
| 1371362_at | Kdelr3 | ER retention | 2107 | 3702 | * |
| 1373382_at | Rer1 | ER retention | 4688 | 3198 | * |
| 1367565_a_at | Fth1 | Iron transport | 14379 | 21718 | * |
| 1367559_at | Ftl | Iron transport | 12387 | 18943 | * |
| 1390383_at | Adfp | Lipid transport | 4343 | 4307 |  |
| 1370862_at | Apoe | Lipid transport | 8380 | 7349 |  |
| 1388918_at, 1398859_at | Hdlbp | Lipid transport | 2042, 3847 | 2902, 4624 | * |
| 1398892_at | Npc2 | Lipid transport | 8830 | 7202 | * |
| 1398806_at | Pitpna | Lipid transport | 3718 | 2764 | * |
| 1386952_a_at | Dync1i2 | Microtubule-based transport | 2199 | 3225 | * |
| 1369976_at | Dynll1 | Microtubule-based transport | 11527 | 7611 | * |
| 1372612_at | Dynll2 | Microtubule-based transport | 5025 | 2586 | * |
| 1370179_at | Dynlrb1 | Microtubule-based transport | 9121 | 5827 | * |
| 1386882_at | Dynlt1 | Microtubule-based transport | 4022 | 3542 |  |
| 1371474_at | Mtch1 | Mitochondrial transport | 10146 | 6776 | * |
| 1375131_at | Mtch2 | Mitochondrial transport | 8384 | 6588 | * |
| 1388766_at | Mtx2 | Mitochondrial transport | 3181 | 3018 |  |
| 1388380_at | Samm50 | Mitochondrial transport | 4093 | 3199 | * |
| 1370366_at | Timm10 | Mitochondrial transport | 5052 | 3305 | * |
| 1398868_at | Timm13 | Mitochondrial transport | 5282 | 3277 | * |
| 1370304_at | Timm17a | Mitochondrial transport | 4378 | 2817 | * |
| 1398763_at | Timm23 | Mitochondrial transport | 8194 | 5473 | * |
| 1367808_at | Timm8b | Mitochondrial transport | 5594 | 5037 |  |
| 1370785_s_at, 1398870_at | Tomm20 | Mitochondrial transport | 6169, 7052 | 4765, 6366 | * |
| 1367473_at | Tomm22 | Mitochondrial transport | 4374 | 3207 | * |
| 1389518_at | Tomm5 | Mitochondrial transport | 4286 | 3166 | * |
| 1371399_at | Tomm6 | Mitochondrial transport | 5382 | 3492 | * |
| 1389544_at | Tomm7 | Mitochondrial transport | 6402 | 5084 |  |
| 1375185_at | Ipo7 | Nuclear transport | 1323 | 2993 | * |
| 1367683_at | Kpna2 | Nuclear transport | 2767 | 5188 | * |
| 1371246_at | Nutf2 | Nuclear transport | 3646 | 1920 | * |
| 1389903_at | Pttg1ip | Nuclear transport | 5674 | 4486 | * |
| 1368888_a_at | Rtn4 | Nuclear transport | 4305 | 6877 | * |
| 1367704_at | Ap2b1 | Protein transport | 2040 | 3553 | * |
| 1398765_at | Ap2m1 | Protein transport | 9559 | 5940 | * |
| 1367549_a_at | Ap3d1 | Protein transport | 5118 | 4946 |  |
| 1398896_at | Arcn1 | Protein transport | 3156 | 3862 |  |
| 1373042_at | Chmp2a | Protein transport | 4834 | 3388 | * |
| 1367512_at | Chmp5 | Protein transport | 4593 | 3265 | * |
| 1386875_a_at | Clta | Protein transport | 9361 | 7614 | * |
| 1367907_a_at | Cltb | Protein transport | 6538 | 4196 | * |
| 1398842_at | Cltc | Protein transport | 1983 | 5428 | * |
| 1388682_at | Cnih | Protein transport | 4770 | 4561 |  |
| 1388377_at | Copa | Protein transport | 3294 | 6325 | * |
| 1372464_at | Copg | Protein transport | 1970 | 2973 | * |
| 1375520_at | Copz1 | Protein transport | 6811 | 4133 | * |
| 1368202_a_at, 1372031_at | Dab2 | Protein transport | 4833, 2582 | 5170, 3546 |  |
| 1368504_at | Lamp1 | Protein transport | 6897 | 6313 |  |
| 1370010_at | Lamp2 | Protein transport | 5805, 3414 | 7890, 5917 | * |
| 1388748_at | Laptm4a | Protein transport | 8458 | 11385 | * |
| 1388728_at | Laptm4b | Protein transport | 4969 | 4716 |  |
| 1398782_at | Napa | Protein transport | 3360 | 2301 | * |
| 1373877_at | Picalm | Protein transport | 3638 | 4406 |  |
| 1398887_at | Robld3 | Protein transport | 5434 | 3150 | * |
| 1367470_at, 1398353_at | Sar1a | Protein transport | 6371, 3863 | 4483, 3336 | * |
| 1388681_at | Sar1b | Protein transport | 3609 | 3654 |  |
| 1369991_at | Sec11a | Protein transport | 4334 | 3548 | * |
| 1371437_at | Sec13 | Protein transport | 3885 | 3207 | * |
| 1373610_at | Sec24d | Protein transport | 2118 | 3253 | * |
| 1371368_at | Sec61a1 | Protein transport | 2211 | 2977 | * |
| 1388519_at | Sec61b | Protein transport | 9042 | 5057 | * |
| 1371428_at | Sec61g | Protein transport | 11159 | 9704 |  |
| 1375296_at | Snx3 | Protein transport | 9002 | 5650 | * |
| 1372743_at | Snx5 | Protein transport | 4169 | 3630 |  |
| 1373090_at | Ssr1 | Protein transport | 2480 | 3191 |  |
| 1373185_at | Ssr2 | Protein transport | 5215 | 4449 |  |
| 1375652_at, 1388777_at | Ssr3 | Protein transport | 5734, 8687 | 4114, 8361 |  |
| 1367690_at | Ssr4 | Protein transport | 5794 | 5062 |  |
| 1370014_at | Stx4 | Protein transport | 4417 | 2503 | * |
| 1389973_a_at | Surf4 | Protein transport | 4168 | 2950 | * |
| 1372474_at | Sypl1 | Protein transport | 4731 | 5876 | * |
| 1398796_at | Tmed10 | Protein transport | 5070 | 4824 |  |
| 1389940_at, 1398824_at | Tmed2 | Protein transport | 9344, 3476 | 7102, 3798 |  |
| 1373862_at | Tmed7 | Protein transport | 2929 | 3532 | * |
| 1388369_at | Tmed9 | Protein transport | 5898 | 3916 | * |
| 1371688_at | Tram1 | Protein transport | 5212 | 5517 |  |
| 1372284_at | Trappc3 | Protein transport | 3274 | 1954 | * |
| 1399159_a_at | Vamp3 | Protein transport | 4913 | 3101 | * |
| 1398840_at | Vamp5 | Protein transport | 4148 | 2473 | * |
| 1398843_at | Vapa | Protein transport | 9326 | 8524 |  |
| 1367455_at | Vcp | Protein transport | 3525 | 6797 | * |
| 1371639_at | Vps29 | Protein transport | 5555 | 4097 | * |
| 1372806_at | Vps35 | Protein transport | 2528 | 4507 | * |
| 1371843_at | Yipf5 | Protein transport | 4603 | 4047 | * |
| 1367939_at | Rbp1 | Vitamin transport | 8662 | 4127 | * |
| --- | --- | --- | --- | --- |  |
| **Transcriptional regulation** | | | --- | --- |  |
| 1367664_at, 1367665_at | Ankrd1 | Transcriptional regulation | 16642, 12993 | 20076, 16797 |  |
| 1367624_at | Atf4 | Transcriptional regulation | 9396 | 5811 | * |
| 1372601_at | Atf5 | Transcriptional regulation | 4599 | 1896 | * |
| 1367657_at | Btg1 | Transcriptional regulation | 12176 | 12478 |  |
| 1387002_at | Bud31 | Transcriptional regulation | 4307 | 3072 | * |
| 1371949_at | Bzw1 | Transcriptional regulation | 3646 | 3380 |  |
| 1367972_at | Cand1 | Transcriptional regulation | 2046 | 3898 | * |
| 1371536_at, 1388659_at | Carhsp1 | Transcriptional regulation | 7100, 4043 | 4517, 2061 | * |
| 1399033_at | Cbfb | Transcriptional regulation | 3362 | 2845 | * |
| 1387087_at | Cebpb | Transcriptional regulation | 7014 | 3433 | * |
| 1368141_at | Cnbp | Transcriptional regulation | 8008 | 7055 | * |
| 1370169_at, 1387863_at | Csde1 | Transcriptional regulation | 3145, 3691 | 3670, 4885 |  |
| 1375189_at | Edf1 | Transcriptional regulation | 5349 | 3498 | * |
| 1368321_at | Egr1 | Transcriptional regulation | 6553 | 3193 | * |
| 1388503_at | Eid1 | Transcriptional regulation | 5706 | 3564 | * |
| 1389617_at | Elk3 | Transcriptional regulation | 3521 | 2522 | * |
| 1376606_a_at | Eny2 | Transcriptional regulation | 4095 | 2648 | * |
| 1372107_at, 1390049_at | Fhl1 | Transcriptional regulation | 10462, 4064 | 10700, 5158 |  |
| 1369313_at, 1371951_at | Fhl2 | Transcriptional regulation | 3359, 7329 | 4053, 8109 |  |
| 1387894_at | Gata4 | Transcriptional regulation | 3755 | 3680 |  |
| 1374335_at | Gata6 | Transcriptional regulation | 2136 | 2997 | * |
| 1387076_at | Hif1a | Transcriptional regulation | 3831 | 5938 | * |
| 1388309_at | Hmga1 | Transcriptional regulation | 3908 | 2357 | * |
| 1371989_at | Hmgn3 | Transcriptional regulation | 3317 | 2371 | * |
| 1393138_at | Jund | Transcriptional regulation | 7106 | 7532 |  |
| 1388447_at | Lbh | Transcriptional regulation | 9845 | 8079 |  |
| 1370928_at | Litaf | Transcriptional regulation | 9620 | 5508 | * |
| 1373374_at | Lmo4 | Transcriptional regulation | 6709 | 4667 | * |
| 1386895_at | Maged1 | Transcriptional regulation | 3181 | 4428 | * |
| 1383093_at | Mef2a | Transcriptional regulation | 2680 | 4098 | * |
| 1367826_at | Nfe2l2 | Transcriptional regulation | 4163 | 4421 |  |
| 1367946_at | Pdlim1 | Transcriptional regulation | 3026 | 2909 |  |
| 1370381_at | Pnrc1 | Transcriptional regulation | 4615 | 3415 |  |
| 1371856_at | Pnrc2 | Transcriptional regulation | 4569 | 4047 | * |
| 1392543_at | Rbbp4 | Transcriptional regulation | 3299 | 2785 |  |
| 1367468_at | Scand1 | Transcriptional regulation | 3843 | 2312 | * |
| 1371521_at | Sin3b | Transcriptional regulation | 3934 | 2472 | * |
| 1383137_at | Sox4 | Transcriptional regulation | 4355 | 5030 |  |
| 1388842_at | Srf | Transcriptional regulation | 4065 | 2603 | * |
| 1370224_at | Stat3 | Transcriptional regulation | 4649 | 5611 |  |
| 1398880_at | Sub1 | Transcriptional regulation | 10461 | 8997 |  |
| 1388392_at | Tax1bp3 | Transcriptional regulation | 5819 | 3759 | * |
| 1398759_at | Tsc22d1 | Transcriptional regulation | 15220 | 14936 |  |
| 1371550_at | Tsc22d4 | Transcriptional regulation | 3533 | 2173 | * |
| 1371131_a_at | Txnip | Transcriptional regulation | 4822 | 5221 |  |
| 1372758_at | Wwtr1 | Transcriptional regulation | 3590 | 4024 |  |
| 1371249_at | Xbp1 | Transcriptional regulation | 7029 | 4522 | * |
| 1371433_at | Yap1 | Transcriptional regulation | 3693 | 3308 |  |
| 1370213_at | Ybx1 | Transcriptional regulation | 8887 | 12707 | * |
| 1369959_at | Zfp36l1 | Transcriptional regulation | 4821 | 4844 |  |
| 1373369_at | Zmiz1 | Transcriptional regulation | 2376 | 2992 | * |
| 1383260_at | Znf553/771 | Transcriptional regulation | 3445 | 1955 | * |
| 1371384_at | Btf3 | Transcriptional regulation | 8370 | 7782 |  |
| 1373846_at | Btf3l4 | Transcriptional regulation | 3632 | 2486 | * |
| 1388533_at | Ctdsp1 | Transcriptional regulation | 4397 | 2764 | * |
| 1371792_at | Ctdsp2 | Transcriptional regulation | 6827 | 4027 | * |
| 1387775_at | Gtf2a2 | Transcriptional regulation | 4818 | 4056 |  |
| 1367509_at | Gtf2h5 | Transcriptional regulation | 3640 | 2604 | * |
| 1371351_at | Polr1d | Transcriptional regulation | 4756 | 4302 |  |
| 1367471_at | Polr2e | Transcriptional regulation | 6119 | 3216 | * |
| 1367769_at | Polr2g | Transcriptional regulation | 3398 | 2134 | * |
| 1372556_at | Polr2l | Transcriptional regulation | 4453 | 3479 | * |
| 1371406_at | Ptrf | Transcriptional regulation | 6250 | 5367 | * |
| 1375414_at | Taf9 | Transcriptional regulation | 5701 | 5096 | * |
| 1367485_at | Tcea1 | Transcriptional regulation | 3338 | 3134 |  |
| 1398794_at | Tceb1 | Transcriptional regulation | 5108 | 3552 | * |
| 1398837_at | Tceb2 | Transcriptional regulation | 8985 | 6123 | * |
| 1372301_at | Aebp1 | Transcriptional regulation | 1782 | 2979 | * |
| 1387861_at | Aes | Transcriptional regulation | 3525 | 2658 | * |
| 1370029_at | Ctbp1 | Transcriptional regulation | 6916 | 4096 | * |
| 1389207_at | Egln1 | Transcriptional regulation | 4099 | 2955 | * |
| 1368174_at | Egln3 | Transcriptional regulation | 6103 | 5357 |  |
| 1370908_at | Hdac2 | Transcriptional regulation | 3633 | 3454 |  |
| 1371490_at | Hsbp1 | Transcriptional regulation | 3346 | 2087 | * |
| 1387028_a_at | Id1 | Transcriptional regulation | 6337 | 3549 | * |
| 1387769_a_at | Id3 | Transcriptional regulation | 11555 | 7851 | * |
| 1373566_at | Irf2bp2 | Transcriptional regulation | 4533 | 3479 | * |
| 1398773_at | Khdrbs1 | Transcriptional regulation | 2630 | 3717 | * |
| 1388774_at | Mbd2 | Transcriptional regulation | 4099 | 2452 | * |
| 1371345_at | Mbd3 | Transcriptional regulation | 4109 | 1918 | * |
| 1367927_at | Phb | Transcriptional regulation | 3656 | 3288 |  |
| 1367463_at | Phb2 | Transcriptional regulation | 6869 | 5328 | * |
| 1398768_at | Rbbp7 | Transcriptional regulation | 5820 | 6756 |  |
| 1392465_at | Sap18 | Transcriptional regulation | 5530 | 3364 | * |
| 1372938_at | Smarce1 | Transcriptional regulation | 3961 | 3356 |  |
| 1388478_at | Tbl1x | Transcriptional regulation | 3178 | 2014 | * |
| 1371870_at | Tcf25 | Transcriptional regulation | 3496 | 2740 |  |
| 1372155_at | Trim28 | Transcriptional regulation | 3703 | 4964 | * |
| --- | --- | --- | --- | --- |  |
| **Others** | | | --- | --- |  |
| 1399019_at | Abhd4 | Unknown function | 4453 | 2765 | * |
| 1373059_at | Ankrd13a | Unknown function | 3293 | 2280 | * |
| 1386915_at | Anp32b | Unknown function | 6229 | 4494 | * |
| 1398874_at | Atxn10 | Unknown function | 4053 | 3978 |  |
| 1370252_at | Avpi1 | Unknown function | 5851 | 3931 | * |
| 1398350_at | Basp1 | Unknown function | 9543 | 6419 | * |
| 1372137_at | Bloc1s1 | Unknown function | 4087 | 2570 | * |
| 1371833_at | Bri3 | Unknown function | 8812 | 7205 | * |
| 1371379_at | Brp44 | Unknown function | 5878 | 3869 | * |
| 1386867_at | Brp44l | Unknown function | 10396 | 9458 |  |
| 1367643_at | Bsg | Unknown function | 9889 | 9625 |  |
| 1371938_at, 1371939_at, 1395412_at | Caprin1 | Unknown function | 2977 | 4209 | * |
| 1375634_at | Ccdc53 | Unknown function | 4292 | 2558 | * |
| 1375246_at | Ccdc72 | Unknown function | 5787 | 7548 |  |
| 1367709_at | Cd63 | Unknown function | 14892 | 15801 |  |
| 1371918_at | Cd99 | Unknown function | 4080 | 2931 |  |
| 1389126_at | Chchd1 | Unknown function | 4070 | 2464 | * |
| 1398326_at | Chchd10 | Unknown function | 13607 | 10401 | * |
| 1371312_at | Chchd2 | Unknown function | 12931 | 8141 | * |
| 1389291_at | Chchd3 | Unknown function | 3403 | 2733 | * |
| 1398894_at | Commd3 | Unknown function | 5700 | 4812 | * |
| 1367604_at | Crip2 | Unknown function | 15305 | 13524 | * |
| 1398928_at | Cuta | Unknown function | 4732 | 2379 | * |
| 1371629_at | Cxxc5 | Unknown function | 3613 | 2384 | * |
| 1367532_at, 1372511_at | Dazap2 | Unknown function | 6658, 3880 | 4954, 3138 | * |
| 1374388_at | Efhd2 | Unknown function | 3435 | 1808 | * |
| 1372106_at, 1375739_at | Ehd4 | Unknown function | 3120, 5396 | 4085, 6094 |  |
| 1371527_at | Emp1 | Unknown function | 7831 | 4530 | * |
| 1367914_at | Emp3 | Unknown function | 3887 | 2932 | * |
| 1371544_at | Erh | Unknown function | 7376 | 4176 | * |
| 1372526_at | Flcn | Unknown function | 2922 | 2903 |  |
| 1389015_at | Fundc2 | Unknown function | 4294 | 2470 | * |
| 1388395_at | G0s2 | Unknown function | 9021 | 6308 | * |
| 1373021_at | Gbas | Unknown function | 3640 | 3204 |  |
| 1371574_at | Ghitm | Unknown function | 5695 | 6678 | * |
| 1387367_at | Glg1 | Unknown function | 1059 | 2864 | * |
| 1390356_at | Gmfb | Unknown function | 3909 | 2920 | * |
| 1398895_at | Golga7 | Unknown function | 3443 | 2855 | * |
| 1371547_at | Grcc10 | Unknown function | 5866 | 3433 | * |
| 1371510_at | Hcfc1r1 | Unknown function | 3972 | 2902 | * |
| 1370062_at | Higd1a | Unknown function | 12424 | 8123 | * |
| 1398933_at | Higd2a | Unknown function | 2937 | 2927 |  |
| 1398932_at | Hint1 | Unknown function | 10218 | 9467 |  |
| 1368042_a_at | Hmg1l1 | Unknown function | 7936 | 7538 |  |
| 1371336_at | Hn1 | Unknown function | 8510 | 5851 | * |
| 1390382_at | Hypk | Unknown function | 3548 | 2028 | * |
| 1387770_at | Ifi27l | Unknown function | 16901 | 15346 |  |
| 1372013_at | Ifitm1 | Unknown function | 12331 | 7198 | * |
| 1367696_at | Ifitm2 | Unknown function | 8655 | 5391 | * |
| 1387995_a_at | Ifitm3 | Unknown function | 13575 | 12399 |  |
| 1372080_at | Immt | Unknown function | 1350 | 3235 | * |
| 1376845_at | isg12(b) | Unknown function | 12649 | 7967 | * |
| 1371334_at | Itm2c | Unknown function | 7737 | 5997 | * |
| 1374406_at | Klhdc2 | Unknown function | 3569 | 2680 | * |
| 1389482_at | Krcc1 | Unknown function | 3830 | 2659 | * |
| 1371540_at | Krtcap2 | Unknown function | 5048 | 3514 | * |
| 1387011_at | Lcn2 | Unknown function | 13607 | 13129 |  |
| 1373151_at | Lhfp | Unknown function | 6297 | 5343 | * |
| 1372234_at | Lix1l | Unknown function | 3616 | 2323 | * |
| 1389147_at | Lrrc58 | Unknown function | 4806 | 5541 | * |
| 1371445_at | Lrrc59 | Unknown function | 4972 | 4492 | * |
| 1388683_at | Lsmd1 | Unknown function | 4593 | 2699 | * |
| 1389301_at | Mbnl2 | Unknown function | 3103 | 3502 |  |
| 1388388_at | Mea1 | Unknown function | 6478 | 3661 | * |
| 1371820_at | Mesdc2 | Unknown function | 3861 | 3106 | * |
| 1371731_at | Mest | Unknown function | 6444 | 5433 |  |
| 1376589_at | Mettl9 | Unknown function | 7008 | 6318 |  |
| 1371941_at | Mff | Unknown function | 4920 | 3975 | * |
| 1371359_at | Mlf2 | Unknown function | 3798 | 2423 | * |
| 1388311_at | Mrfap1 | Unknown function | 13646 | 12843 |  |
| 1388427_at | Mxra8 | Unknown function | 2812 | 3058 |  |
| 1388453_at | Myadm | Unknown function | 4234 | 4303 |  |
| 1399106_at | Myeov2 | Unknown function | 6953 | 6350 |  |
| 1371407_at, 1388196_at | Nckap1 | Unknown function | 6053, 3841 | 7600, 6187 | * |
| 1398893_at | Ndfip1 | Unknown function | 6480 | 6051 |  |
| 1387121_a_at | Ndrg2 | Unknown function | 6825 | 5748 |  |
| 1370229_at | Ndrg4 | Unknown function | 3787 | 4376 |  |
| 1371782_at | Nipsnap3a | Unknown function | 3226 | 1523 | * |
| 1371412_a_at | Nrep | Unknown function | 8242 | 5122 | * |
| 1370199_at | Nucb1 | Unknown function | 2812 | 3679 | * |
| 1372336_at | Nucks1 | Unknown function | 3644 | 2728 | * |
| 1388612_at | Ociad1 | Unknown function | 3230 | 3560 |  |
| 1371924_at | Olfml3 | Unknown function | 4414 | 4204 |  |
| 1372704_at | Ostc | Unknown function | 6589 | 4173 | * |
| 1372246_at | Ostf1 | Unknown function | 3185 | 2133 | * |
| 1375173_at | P76 | Unknown function | 3588 | 2889 |  |
| 1371933_at | Pde4dip | Unknown function | 2451 | 3857 | * |
| 1388393_at | Plp2 | Unknown function | 5610 | 5589 |  |
| 1392534_at | Pmepa1 | Unknown function | 3258 | 1852 | * |
| 1388351_at | Pomp | Unknown function | 11272 | 7760 | * |
| 1376646_at | Popdc2 | Unknown function | 7451 | 6645 |  |
| 1371620_at | Prelid1 | Unknown function | 3525 | 2102 | * |
| 1388408_at | Prr13 | Unknown function | 5812 | 2892 | * |
| 1370243_a_at | Ptma | Unknown function | 9542 | 6088 | * |
| 1386892_at | Ptms | Unknown function | 4511 | 2432 | * |
| 1371481_at | Reep5 | Unknown function | 7817 | 6596 |  |
| 1368238_at | Reg3b | Unknown function | 16868 | 21007 |  |
| 1374545_at | Rkhd2 | Unknown function | 2813 | 3039 |  |
| 1371526_at | Rnf10 | Unknown function | 4071 | 2321 | * |
| 1371597_at | Rnf187 | Unknown function | 3511 | 1816 | * |
| 1387778_at | Sdf4 | Unknown function | 3977 | 3282 |  |
| 1375638_at | Sdpr | Unknown function | 3968 | 4978 |  |
| 1371302_at | Serf2 | Unknown function | 15023 | 15899 |  |
| 1373033_at | Sft2d1 | Unknown function | 3239 | 2745 | * |
| 1371904_at | Smyd2 | Unknown function | 4033 | 2900 | * |
| 1388565_at | Spg21 | Unknown function | 3809 | 2459 | * |
| 1388562_at | Stard7 | Unknown function | 3765 | 3860 |  |
| 1371509_at | Tbrg1 | Unknown function | 3834 | 3572 |  |
| 1370887_at | Tgfb1i1 | Unknown function | 6596 | 5427 | * |
| 1367496_at | Tm9sf2 | Unknown function | 2281 | 4036 | * |
| 1372230_at | Tmem147 | Unknown function | 4226 | 2856 | * |
| 1387636_a_at | Tmem14c | Unknown function | 3544 | 2021 | * |
| 1372721_at | Tmem167 | Unknown function | 4591 | 3771 |  |
| 1368840_at | Tmem176b | Unknown function | 5535 | 4795 |  |
| 1373058_at | Tmem30a | Unknown function | 2295 | 3837 | * |
| 1376706_at | Tmem47 | Unknown function | 3669 | 2597 | * |
| 1370807_at | Tmem49 | Unknown function | 5119 | 6024 | * |
| 1372100_at | Tmem50a | Unknown function | 5737 | 3910 | * |
| 1399012_at | Tmem59 | Unknown function | 4501 | 5010 |  |
| 1399013_at | Tmem85 | Unknown function | 3860 | 2630 | * |
| 1371454_at | Tmem93 | Unknown function | 3872 | 2581 | * |
| 1388492_at | Tnip1 | Unknown function | 4556 | 2413 | * |
| 1388308_at | Tprgl | Unknown function | 6100 | 2874 | * |
| 1370176_at | Trak2 | Unknown function | 6290 | 3840 | * |
| 1375517_at | Trp53inp2 | Unknown function | 11485 | 8662 | * |
| 1398325_at | Tspan3 | Unknown function | 6360 | 4697 | * |
| 1372752_at | Tspan4 | Unknown function | 5446 | 3999 | * |
| 1372286_at | Tspan6 | Unknown function | 3720 | 3617 |  |
| 1398754_at | Uba52 | Unknown function | 12078 | 12799 |  |
| 1370238_at | Usmg5 | Unknown function | 13102 | 14078 |  |
| 1389857_at | Wbp5 | Unknown function | 8360 | 5927 | * |
| 1389520_at | Wdr1 | Unknown function | 3787 | 4525 |  |
| 1389165_at | Wdr26 | Unknown function | 4142 | 3446 | * |
| 1383651_a_at | Wdr45l | Unknown function | 3502 | 2673 | * |
| 1373149_at | Ypel3 | Unknown function | 3534 | 1661 | * |
| 1371960_at | Ythdf2 | Unknown function | 3193 | 4202 |  |
| 1388868_at | Zfand5 | Unknown function | 4667 | 4425 |  |
| 1389531_at | Zfp330 | Unknown function | 3421 | 2884 | * |
| 1372865_at | Zfp364 | Unknown function | 4653 | 3941 | * |
| 1372878_at | Zfr | Unknown function | 1893 | 2988 | * |
| 1398962_at | Fam108a1 | Hypothetical protein | 3688 | 2029 | * |
| 1371386_at | Fam120a | Hypothetical protein | 7773 | 7135 |  |
| 1388482_at | Fam129b | Hypothetical protein | 3679 | 2666 | * |
| 1373011_at | Fam134b | Hypothetical protein | 3230 | 4638 | * |
| 1372767_at | Fam168b | Hypothetical protein | 4141 | 2675 | * |
| 1372621_at | Fam176b | Hypothetical protein | 3659 | 1474 | * |
| 1371461_at | Fam54b | Hypothetical protein | 6462 | 4204 | * |
| 1374487_at | Fam96a | Hypothetical protein | 4737 | 4332 |  |
| 1372258_at | LOC100192313 | Hypothetical protein | 3926 | 2607 | * |
| 1371599_at | LOC294154 | Hypothetical protein | 3877 | 2715 | * |
| 1398909_at | LOC301124 | Hypothetical protein | 7862 | 5253 | * |
| 1388382_at | LOC361985 | Hypothetical protein | 3949 | 3472 |  |
| 1373100_at | LOC499779 | Hypothetical protein | 4744 | 3033 |  |
| 1371531_at | LOC678880 | Hypothetical protein | 4937 | 2697 | * |
| 1371460_at | LOC681219 | Hypothetical protein | 5043 | 3359 | * |
| 1373648_at | LOC681849 | Hypothetical protein | 3093 | 4284 | * |
| 1373088_at | LOC682888 | Hypothetical protein | 7139 | 4368 | * |
| 1371381_at | LOC685322 | Hypothetical protein | 12886 | 13778 |  |
| 1376209_at | LOC687237 | Hypothetical protein | 6635 | 3976 | * |
| 1388441_at | LOC689574 | Hypothetical protein | 7618 | 5241 | * |
| 1371434_at | LOC691807 | Hypothetical protein | 5201 | 3572 | * |
| 1388980_at | MGC72560 | Hypothetical protein | 3653 | 2466 | * |
| 1371905_at | MGC94190 | Hypothetical protein | 4254 | 3673 |  |
| 1371946_at | RGD1303130 | Hypothetical protein | 5192 | 3873 | * |
| 1398919_at | RGD1304704 | Hypothetical protein | 6990 | 4988 | * |
| 1399001_at | RGD1305481 | Hypothetical protein | 4292 | 3703 | * |
| 1374333_at | RGD1306058 | Hypothetical protein | 3290 | 2526 | * |
| 1383175_a_at, 1385458_a_at, 1392938_s_at | RGD1306959 | Hypothetical protein | 5065, 4625, 6009 | 2998, 2582, 3786 | * |
| 1371478_at | RGD1307752 | Hypothetical protein | 8749 | 5352 | * |
| 1398971_at | RGD1307929 | Hypothetical protein | 3386 | 2727 | * |
| 1374554_at | RGD1308048 | Hypothetical protein | 3560 | 1809 | * |
| 1388114_at | RGD1309537 | Hypothetical protein | 12192 | 15077 | * |
| 1371619_at | RGD1310352 | Hypothetical protein | 3449 | 3425 |  |
| 1375637_at | RGD1311122 | Hypothetical protein | 4322 | 2282 | * |
| 1389575_at | RGD1311703 | Hypothetical protein | 3511 | 2588 | * |
| 1376878_at | RGD1559896 | Hypothetical protein | 5226 | 3241 | * |
| 1389343_at | RGD1560187 | Hypothetical protein | 10305 | 7303 | * |
| 1383643_at | RGD1560328 | Hypothetical protein | 7241 | 5447 | * |
| 1371409_at | RGD1562987 | Hypothetical protein | 3380 | 1908 | * |
| 1375526_at | RGD1563438 | Hypothetical protein | 6039 | 3609 | * |
| 1371410_at | RGD1564058 | Hypothetical protein | 4792 | 3582 | * |
| 1371405_at | RGD1565095 | Hypothetical protein | 3953 | 3505 |  |
| 1371736_at | RGD1565192 | Hypothetical protein | 3683 | 2884 |  |
| 1377114_at | Mitochondrial genome | Mitochondrial genome | 798 | 3017 | * |
| 1377719_a_at | Mitochondrial genome | Mitochondrial genome | 1303 | 4732 | * |
| 1377720_x_at | Mitochondrial genome | Mitochondrial genome | 1498 | 7369 | * |
| 1383161_a_at | Mitochondrial genome | Mitochondrial genome | 1646 | 8116 | * |
| 1383162_at | Mitochondrial genome | Mitochondrial genome | 1579 | 7757 | * |
| 1371507_at | Unknown | No established gene | 3600 | 6044 | * |
| 1371755_at | Unknown | No established gene | 4579 | 3924 |  |
| 1371926_at | Unknown | No established gene | 2655 | 4687 | * |
| 1372293_at | Unknown | No established gene | 8693 | 7209 |  |
| 1372444_at | Unknown | No established gene | 2869 | 5075 | * |
| 1373140_at | Unknown | No established gene | 2376 | 3809 | * |
| 1373193_at | Unknown | No established gene | 8353 | 6913 |  |
| 1375230_at | Unknown | No established gene | 2293 | 3580 | * |
| 1377705_at | Unknown | No established gene | 6301 | 4404 |  |
| 1383058_at | Unknown | No established gene | 4105 | 2326 |  |
| 1383599_at | Unknown | No established gene | 5686 | 5156 |  |
| 1388516_at | Unknown | No established gene | 4020 | 3730 |  |
| 1389287_at | Unknown | No established gene | 3448 | 3463 |  |
| 1389866_at | Unknown | No established gene | 5728 | 4539 | * |
| 1389904_at | Unknown | No established gene | 4839 | 5174 |  |
| 1392627_x_at | Unknown | No established gene | 3179 | 4300 |  |
| 1399079_at | Unknown | No established gene | 2016 | 6282 | * |
| 1399097_at | Unknown | No established gene | 3793 | 3271 |  |
| 1393252_at | AS: Fbln1 | Non-protein coding | 5622 | 6303 |  |
| 1375705_at | AS: Gnb1 | Non-protein coding | 5961 | 4845 |  |
| 1375532_at | AS: Id2 | Non-protein coding | 5422 | 3831 | * |
| 1390116_at | AS: Ptrf | Non-protein coding | 6500 | 7745 |  |
| 1375687_at | AS: Rab14 | Non-protein coding | 4931 | 4603 |  |
| 1385889_at | AS: RGD1565095 | Non-protein coding | 1906 | 3409 | * |
| 1375343_at | AS: Tcf4 | Non-protein coding | 2827 | 3507 |  |
| 1375422_at | Ets1 | Non-protein coding | 218 | 8995 | * |
| 1373499_at | Gas5 | Non-protein coding | 1309 | 2928 | * |
| 1371298_at | H19 | Non-protein coding | 1893 | 3537 | * |
| 1373920_at | Intron:Trak1 | Non-protein coding | 7110 | 4988 | * |
