# Supplementary material for: Regulation of the cardiomyocyte transcriptome vs translatome by endothelin-1 and insulin: translational regulation of 5' terminal oligopyrimidine tract (TOP) mRNAs by insulin
Source: BMC Genomics. 2010 May 29;11:343. doi: 10.1186/1471-2164-11-343 (PMC2900265; doi:10.1186/1471-2164-11-343)
Supplement: Additional file 3 — Differential expression of cardiomyocyte transcripts in polysomal and total RNA (Microsoft Word Table). Total and polysomal RNA from unstimulated neonatal rat cardiomyocytes were analysed using microarrays. The data were normalised to the gene median. Transcripts with differential expression in total and polysomal RNA pools were identified (t-test with Benjamini-Hochberg FDR correction p < 0.05; >3-fold difference). Mean raw fluorescence and normalised values are given (n = 4). For transcripts represented by more than one probeset, the probesets and mean corresponding raw values are listed. RNAs are listed according to relative expression in polysomal or total pools and whether they are protein-coding, non-protein-coding or associated with no known gene. AS = Antisense. [file 1471-2164-11-343-S3.DOC]

**Additional file 3. Differential expression of cardiomyocyte transcripts in polysomal and total RNA.** Total and polysomal RNA from unstimulated neonatal rat cardiomyocytes were analysed using microarrays. The data were normalised to the gene median. Transcripts with differential expression in total and polysomal RNA pools were identified (t-test with Benjamini-Hochberg FDR correction p<0.05; >3-fold difference). Mean raw fluorescence and normalised values are given (n=4). For transcripts represented by more than one probeset, the probesets and mean corresponding raw values are listed. RNAs are listed according to relative expression in polysomal or total pools and whether they are protein-coding, non-protein-coding or associated with no known gene. AS = Antisense.

| **Probeset** | **Gene symbol** | **Function** | **Raw fluorescence values** | | **Values normalised to gene median** | | **Ratio**  **(T/P)** |
| --- | --- | --- | --- | --- | --- | --- | --- |
|  |  |  | **Polysomal RNA** | **Total RNA** | **Polysomal RNA (P)** | **Total RNA (T)** |
| **Polysome > Total** | | --- | --- | --- | --- | --- | --- |
| 1373085_at | Cbr3 | Regulation of metabolism | 282 | 80 | 1.74 | 0.43 | 0.25 |
| 1376493_at | Commd7 | Intracellular signaling | 1308 | 351 | 1.78 | 0.41 | 0.23 |
| 1391935_at | Eif4e3 | Protein synthesis | 1563 | 456 | 1.66 | 0.49 | 0.29 |
| 1372942_at | Exosc5 | DNA/RNA regulation | 692 | 206 | 1.85 | 0.54 | 0.29 |
| 1368085_at | Gchfr | Regulation of metabolism | 163 | 55 | 1.28 | 0.37 | 0.29 |
| 1387264_at | Kcnk6 | Channels/transporters | 690 | 215 | 1.72 | 0.51 | 0.30 |
| 1373167_at | Lrrc20 | Unknown function | 293 | 86 | 1.32 | 0.32 | 0.24 |
| 1372147_at | Ndufs4 | Regulation of metabolism | 6257 | 2121 | 2.18 | 0.72 | 0.33 |
| 1367984_at | Scaf1 | DNA/RNA regulation | 288 | 71 | 1.36 | 0.31 | 0.23 |
| 1389420_at | Stap2 | Intracellular signaling | 378 | 95 | 1.52 | 0.29 | 0.19 |
| 1379469_at | Tbl1x | Transcriptional regulation | 485 | 181 | 1.17 | 0.34 | 0.29 |
| 1370695_s_at | Trib3 | Intracellular signaling | 927 | 301 | 1.19 | 0.36 | 0.30 |
| --- | --- | --- | --- | --- | --- | --- | --- |
| **Total > Polysome** | | --- | --- | --- | --- | --- | --- |
| ***Protein-coding*** | | | --- | --- | --- | --- | --- |
| 1394490_at, 1384381_at | Abca1 | Channels/transporters | 213, 74 | 631, 236 | 0.53 | 1.68 | 3.17 |
| 1376683_at | Abcb7 | Channels/transporters | 253 | 773 | 0.62 | 1.89 | 3.05 |
| 1391758_at | Ablim1 | Cytoskeleton/myofibrillar | 158 | 500 | 0.45 | 1.44 | 3.19 |
| 1369526_at | Acadsb | Regulation of metabolism | 63 | 186 | 0.51 | 1.61 | 3.13 |
| 1372595_at, 1377256_at | Actn2 | Cytoskeleton/myofibrillar | 3671, 126 | 11095, 431 | 0.58 | 1.87 | 3.25 |
| 1384333_at | Actr2 | Cytoskeleton/myofibrillar | 31 | 91 | 0.53 | 1.62 | 3.06 |
| 1370955_at | Adam10 | Proteolysis | 74 | 260 | 0.56 | 1.86 | 3.33 |
| 1385665_at | Adam19 | Proteolysis | 25 | 109 | 0.31 | 1.63 | 5.19 |
| 1383671_at | Adam9 | Proteolysis | 90 | 334 | 0.50 | 1.80 | 3.63 |
| 1395695_at | Aebp1 | Transcriptional regulation | 363 | 1263 | 0.49 | 1.74 | 3.54 |
| 1371703_at | Ahnak | Unknown function | 2235 | 7066 | 0.51 | 1.69 | 3.28 |
| 1370321_at | Aifm1 | Intracellular signaling | 641 | 2109 | 0.57 | 1.87 | 3.29 |
| 1369069_at | Akap1 | Intracellular signaling | 414 | 1418 | 0.56 | 1.87 | 3.37 |
| 1369326_at | Akap6 | Intracellular signaling | 72 | 309 | 0.52 | 2.20 | 4.19 |
| 1368814_at | Aldh6a1 | Regulation of metabolism | 223 | 706 | 0.57 | 1.83 | 3.18 |
| 1378238_at | Anapc1 | Proteolysis | 83 | 254 | 0.54 | 1.63 | 3.05 |
| 1370638_at | Ank3 | Cytoskeleton/myofibrillar | 234 | 724 | 0.59 | 1.82 | 3.08 |
| 1395108_at | Ap1g1 | Trafficking | 95 | 310 | 0.52 | 1.69 | 3.24 |
| 1383096_at | Aplp2 | Proteolysis | 120 | 684 | 0.40 | 2.25 | 5.66 |
| 1380533_at | App | Agonists/receptors | 712 | 3622 | 0.42 | 2.21 | 5.24 |
| 1393113_at | Arhgap1 | Intracellular signaling | 157 | 571 | 0.41 | 1.51 | 3.71 |
| 1369244_at | Arnt | Transcriptional regulation | 104 | 263 | 0.51 | 1.53 | 3.00 |
| 1376599_at | Atad2 | Channels/transporters | 100 | 343 | 0.54 | 1.87 | 3.43 |
| 1395687_at | Atf7ip | Transcriptional regulation | 51 | 197 | 0.44 | 1.71 | 3.88 |
| 1391211_at | Atp11c | Channels/transporters | 87 | 276 | 0.55 | 1.74 | 3.18 |
| 1371108_a_at | Atp1a1 | Channels/transporters | 1028 | 5621 | 0.37 | 2.03 | 5.48 |
| 1386683_at, 1393596_at | Atrx | DNA/RNA regulation | 118, 32 | 383, 127 | 0.53 | 1.87 | 3.52 |
| 1375323_a_at | Bat3 | Unknown function | 202 | 628 | 0.48 | 1.47 | 3.10 |
| 1392421_at | Baz2b | Transcriptional regulation | 72 | 224 | 0.56 | 1.75 | 3.13 |
| 1385592_at | Bcor | Transcriptional regulation | 254 | 795 | 0.60 | 1.91 | 3.16 |
| 1385437_at | Cad | Regulation of metabolism | 52 | 193 | 0.46 | 1.70 | 3.71 |
| 1395412_at, 1395173_at | Caprin1 | Unknown function | 1179, 332 | 3790, 1214 | 0.51 | 1.73 | 3.38 |
| 1387401_at | Casq2 | Calcium regulation | 1733 | 5357 | 0.59 | 1.81 | 3.08 |
| 1371173_a_at | Cast | Calcium regulation | 135 | 516 | 0.54 | 2.02 | 3.73 |
| 1380549_at | Ccar1 | Intracellular signaling | 37 | 180 | 0.35 | 1.83 | 5.18 |
| 1389564_at | Ccnl2 | Intracellular signaling | 677 | 2048 | 0.45 | 1.40 | 3.07 |
| 1367689_a_at | Cd36 | Agonists/receptors | 824 | 3461 | 0.42 | 1.77 | 4.25 |
| 1368393_at | Cd93 | Cell-cell adhesion | 455 | 1895 | 0.45 | 2.49 | 5.47 |
| 1387029_at | Cfh | Proteolysis | 52 | 158 | 0.55 | 1.75 | 3.17 |
| 1386783_at | Chsy3 | Extracellular matrix | 26 | 123 | 0.38 | 1.79 | 4.74 |
| 1399022_at | Clk1 | Intracellular signaling | 447 | 1900 | 0.49 | 2.11 | 4.27 |
| 1376868_at | Cobll1 | Unknown function | 115 | 376 | 0.46 | 1.45 | 3.18 |
| 1370927_at, 1398321_a_at | Col12a1 | Extracellular matrix | 371, 52 | 1257, 233 | 0.40 | 1.66 | 4.13 |
| 1382050_at | Col4a4 | Extracellular matrix | 112 | 371 | 0.60 | 2.01 | 3.33 |
| 1374705_at | Col4a5 | Extracellular matrix | 382 | 1601 | 0.59 | 2.46 | 4.19 |
| 1369955_at | Col5a1 | Extracellular matrix | 1230 | 4230 | 0.57 | 2.32 | 4.08 |
| 1389966_at, 1396055_at | Col6a3 | Extracellular matrix | 848, 136 | 2719, 583 | 0.56 | 2.05 | 3.67 |
| 1382954_at | Corin | Proteolysis | 55 | 172 | 0.64 | 1.96 | 3.06 |
| 1368420_at | Cp | Trafficking | 150 | 499 | 0.57 | 1.94 | 3.41 |
| 1397954_at | Cpd | Proteolysis | 122 | 388 | 0.58 | 1.85 | 3.19 |
| 1392391_at | Dag1 | Cytoskeleton/myofibrillar | 178 | 646 | 0.38 | 1.40 | 3.73 |
| 1389105_at | Dag1 | Cytoskeleton/myofibrillar | 80 | 409 | 0.30 | 1.53 | 5.05 |
| 1383649_a_at, 1386114_at | Ddx26b | DNA/RNA regulation | 105, 41 | 351, 136 | 0.42 | 1.61 | 3.82 |
| 1385214_at | Ddx3x | DNA/RNA regulation | 491 | 1910 | 0.51 | 1.96 | 3.83 |
| 1374299_at | Dhx9 | DNA/RNA regulation | 241 | 830 | 0.57 | 1.99 | 3.46 |
| 1375936_at | Dsc2 | Cell-cell/matrix adhesion | 172 | 563 | 0.57 | 1.87 | 3.30 |
| 1388506_at | Dsp | Cytoskeleton/myofibrillar | 1423 | 5508 | 0.49 | 1.91 | 3.92 |
| 1398598_at, 1395274_at | Dst | Cell-cell/matrix adhesion | 639, 46 | 2360, 425 | 0.40 | 2.34 | 5.88 |
| 1398803_at | Dync1h1 | Cytoskeleton/myofibrillar | 483 | 2319 | 0.50 | 2.38 | 4.80 |
| 1395586_at | Eef1a1 | Protein synthesis | 299 | 1304 | 0.40 | 1.74 | 4.31 |
| 1397674_at | Eif3c | Protein synthesis | 711 | 1988 | 0.52 | 1.66 | 3.20 |
| 1397697_at | Eif4a2 | Protein synthesis | 74 | 440 | 0.35 | 2.11 | 5.99 |
| 1390340_a_at | Eif4g1 | Protein synthesis | 138 | 423 | 0.51 | 1.71 | 3.33 |
| 1382040_at | Eprs | Protein synthesis | 235 | 1331 | 0.41 | 2.33 | 5.68 |
| 1371519_at | Etfdh | Regulation of metabolism | 500 | 1673 | 0.58 | 1.93 | 3.35 |
| 1387351_at, 1368829_at | Fbn1 | Extracellular matrix | 1276, 1431 | 3930, 4585 | 0.55 | 1.77 | 3.20 |
| 1374667_at | Fhod3 | Cytoskeleton/myofibrillar | 631 | 2011 | 0.61 | 1.89 | 3.13 |
| 1388401_at | Flnb | Cytoskeleton/myofibrillar | 252 | 893 | 0.61 | 2.12 | 3.48 |
| 1388496_at, 1396085_at | Flnc | Cytoskeleton/myofibrillar | 787, 382 | 2800, 1455 | 0.47 | 1.68 | 3.58 |
| 1370234_at | Fn1 | Extracellular matrix | 4364 | 14991 | 0.53 | 1.83 | 3.47 |
| 1376532_at | Fndc3b | Extracellular matrix | 312 | 1028 | 0.53 | 1.70 | 3.18 |
| 1367756_at | Gfm1 | Transcriptional regulation | 454 | 1503 | 0.62 | 1.99 | 3.22 |
| 1369640_at | Gja1 | Channels/transporters | 529 | 2041 | 0.50 | 1.97 | 3.95 |
| 1382616_at | Gls | Regulation of metabolism | 67 | 300 | 0.40 | 1.78 | 4.50 |
| 1389019_at | Golgb1 | Trafficking | 74 | 248 | 0.49 | 1.73 | 3.52 |
| 1382986_at | Gpam | Regulation of metabolism | 79 | 276 | 0.47 | 1.64 | 3.49 |
| 1397211_at | Grb10 | Intracellular signaling | 139 | 569 | 0.53 | 2.18 | 4.15 |
| 1376892_at | Gria3 | Agonists/receptors | 169 | 575 | 0.70 | 2.24 | 3.19 |
| 1370164_at | Hadha | Regulation of metabolism | 1071 | 3244 | 0.64 | 1.95 | 3.02 |
| 1383780_at, 1372394_at, 1392259_at | Hectd1 | Proteolysis | 23, 648, 56 | 217, 2062, 287 | 0.38 | 1.93 | 5.09 |
| 1390648_at | Herc2 | Proteolysis | 146 | 647 | 0.47 | 2.12 | 4.54 |
| AFFX_Rat_Hexokinase_5/M_at | Hk1 | Regulation of metabolism | 91, 234 | 429, 1149 | 0.44 | 2.09 | 4.78 |
| 1373540_at | Hnrnpa2b1 | DNA/RNA regulation | 222 | 753 | 0.61 | 2.09 | 3.43 |
| 1370997_at | Homer1 | Transcriptional regulation | 11 | 34 | 0.37 | 1.16 | 3.12 |
| 1370690_at | Hspa9 | Chaperone | 517 | 2020 | 0.55 | 2.12 | 3.88 |
| 1380561_at | Iars2 | Protein synthesis | 153 | 522 | 0.53 | 1.81 | 3.44 |
| 1367636_at | Igf2r | Agonists/receptors | 1536 | 4543 | 0.54 | 1.61 | 3.00 |
| 1396152_s_at | Igfbp5 | Agonists/receptors | 45 | 166 | 0.38 | 1.39 | 3.65 |
| 1370750_a_at | Il1r1 | Agonists/receptors | 117 | 458 | 0.44 | 1.65 | 3.77 |
| 1395339_at | Impact | Proteolysis | 157 | 528 | 0.46 | 1.51 | 3.29 |
| 1394065_at | Ints2 | DNA/RNA regulation | 66 | 216 | 0.66 | 2.20 | 3.30 |
| 1388762_at | Iqgap1 | Intracellular signaling | 290 | 962 | 0.57 | 1.87 | 3.28 |
| 1388240_a_at | Itga7 | Cell-cell/matrix adhesion/interaction | 322 | 1242 | 0.45 | 1.76 | 3.87 |
| 1372811_at | Ktn1 | Cytoskeleton/myofibrillar | 468 | 1710 | 0.54 | 1.96 | 3.67 |
| 1390404_at | Lama2 | Extracellular matrix | 508 | 1810 | 0.57 | 2.08 | 3.63 |
| 1367880_at | Lamb2 | Extracellular matrix | 734 | 2276 | 0.67 | 2.05 | 3.05 |
| 1391022_at | Lamb3 | Extracellular matrix | 431 | 1458 | 0.55 | 1.85 | 3.39 |
| 1370993_at | Lamc1 | Extracellular matrix | 939 | 4323 | 0.44 | 2.07 | 4.66 |
| 1388218_at | Ldlr | Agonists/receptors | 115 | 533 | 0.38 | 2.04 | 5.36 |
| 1396013_at | Letm1 | Cytoskeleton/myofibrillar | 174 | 604 | 0.53 | 1.85 | 3.48 |
| 1383561_at | Lig4 | DNA/RNA regulation | 55 | 296 | 0.28 | 1.74 | 6.23 |
| 1372110_at | Limch1 | Cytoskeleton/myofibrillar | 136 | 477 | 0.51 | 1.84 | 3.59 |
| 1375726_at, 1381190_at | Lmo7 | Cell-cell/matrix adhesion | 550, 584 | 1653, 2983 | 0.41 | 1.73 | 4.18 |
| 1395338_at | Lrpprc | Trafficking | 57 | 485 | 0.28 | 2.51 | 8.84 |
| 1367912_at | Ltbp1 | Agonists/receptors | 154 | 546 | 0.58 | 2.08 | 3.59 |
| 1371940_at | Macf1 | Cytoskeleton/myofibrillar | 700 | 2267 | 0.54 | 1.77 | 3.28 |
| 1392648_at | Mrc1 | Agonists/receptors | 245 | 749 | 0.55 | 1.73 | 3.16 |
| 1385690_at, 1382748_at, 1393152_at | Mut | Regulation of metabolism | 79, 109, 134 | 353, 519, 644 | 0.52 | 2.43 | 4.70 |
| 1370158_at | Myh10 | Cytoskeleton/myofibrillar | 457 | 2075 | 0.40 | 1.87 | 4.63 |
| 1387049_at, 1396165_at | Myh6 | Cytoskeleton/myofibrillar | 266, 333 | 6492, 2720 | 0.25 | 3.42 | 13.94 |
| 1398248_s_at | Myh6/Myh7 | Cytoskeleton/myofibrillar | 3720 | 15488 | 0.44 | 1.87 | 4.24 |
| 1367928_at | Myh7 | Cytoskeleton/myofibrillar | 562 | 7169 | 0.27 | 3.49 | 12.98 |
| 1387402_at, 1371725_at | Myh9 | Cytoskeleton/myofibrillar | 51, 1115 | 516, 3504 | 0.35 | 1.86 | 5.23 |
| 1371577_at | Ndufs1 | Regulation of metabolism | 1414 | 4298 | 0.61 | 1.85 | 3.05 |
| 1371483_at | Nnt | Regulation of metabolism | 2348 | 9424 | 0.51 | 2.05 | 4.05 |
| 1368033_at | Nolc1 | DNA/RNA regulation | 60 | 227 | 0.44 | 1.64 | 3.74 |
| 1393277_at | Nup155 | Cytoskeleton/myofibrillar | 52 | 164 | 0.51 | 1.65 | 3.26 |
| 1388758_at, 1370543_at | Ogt | Regulation of metabolism | 310, 154 | 1235, 660 | 0.54 | 2.25 | 4.20 |
| 1384608_at | Opa1 | Trafficking | 178 | 557 | 0.51 | 1.57 | 3.10 |
| 1382443_at | Pabpc4 | DNA/RNA regulation | 856 | 2811 | 0.54 | 1.78 | 3.29 |
| 1392890_at | Pafah1b1 | Proteolysis | 200 | 605 | 0.57 | 1.75 | 3.09 |
| 1393762_at | Pcaf | Transcriptional regulation | 87 | 242 | 0.47 | 1.48 | 3.13 |
| 1371064_at | Pcm1 | Cytoskeleton/myofibrillar | 165 | 645 | 0.36 | 1.72 | 4.79 |
| 1383698_at | Pdha1 | Regulation of metabolism | 1135 | 3594 | 0.52 | 1.66 | 3.16 |
| 1373854_at | Pdpr | Regulation of metabolism | 472 | 1433 | 0.61 | 1.85 | 3.04 |
| 1372390_at | Peg3 | Transcriptional regulation | 520 | 1713 | 0.54 | 1.81 | 3.34 |
| 1383002_at | Phf20l1 | Unknown function | 62 | 196 | 0.60 | 1.91 | 3.19 |
| 1386941_at | Plec1 | Cytoskeleton/myofibrillar | 369 | 1600 | 0.49 | 2.08 | 4.27 |
| 1373913_at | Pnpt1 | DNA/RNA regulation | 194 | 624 | 0.56 | 1.82 | 3.23 |
| 1381850_at, 1383576_at | Ppp1r12a | Intracellular signaling | 204, 611 | 591, 2074 | 0.43 | 1.41 | 3.24 |
| 1382646_at | Prpf4b | DNA/RNA regulation | 140 | 437 | 0.53 | 1.67 | 3.14 |
| 1367466_at | Prpf8 | DNA/RNA regulation | 1102 | 3779 | 0.53 | 1.85 | 3.49 |
| 1375476_at | Pygm | Regulation of metabolism | 503 | 1986 | 0.45 | 1.78 | 3.96 |
| 1383172_at, 1375371_at | Ranbp2 | Intracellular signaling | 291, 145 | 982, 877 | 0.47 | 2.16 | 4.62 |
| 1372081_at | Rbm26 | DNA/RNA regulation | 199 | 614 | 0.55 | 1.73 | 3.15 |
| 1382749_at | Rbm5 | DNA/RNA regulation | 328 | 1415 | 0.47 | 2.09 | 4.46 |
| 1395353_at | RGD1306148 | Hypothetical proteins | 121 | 392 | 0.55 | 1.80 | 3.29 |
| 1381175_at | RGD1307526 | Hypothetical proteins | 71 | 277 | 0.57 | 2.26 | 3.95 |
| 1383933_at | RGD1308772 | Hypothetical proteins | 78 | 242 | 0.56 | 1.78 | 3.15 |
| 1376896_at | RGD1309308 | Hypothetical proteins | 117 | 364 | 0.65 | 2.04 | 3.12 |
| 1390943_at, 1398364_at | RGD1359529 | Hypothetical proteins | 83, 429 | 380, 2454 | 0.46 | 2.40 | 5.26 |
| 1396175_at | RGD1560191 | Hypothetical proteins | 54 | 169 | 0.44 | 1.36 | 3.09 |
| 1393199_at | RGD1560601 | Hypothetical proteins | 47 | 189 | 0.51 | 2.00 | 3.90 |
| 1373514_at | Rnf213 | Unknown function | 68 | 387 | 0.33 | 2.12 | 6.38 |
| 1382088_at | Ryr2 | Channels/transporters | 1132 | 4234 | 0.54 | 2.03 | 3.76 |
| 1388035_a_at | Scn5a | Channels/transporters | 54 | 231 | 0.33 | 1.51 | 4.53 |
| 1397964_at | Sec63 | Trafficking | 29 | 94 | 0.58 | 1.82 | 3.16 |
| 1373534_at, 1398440_at | Sfrs18 | DNA/RNA regulation | 166, 52 | 523, 206 | 0.53 | 1.86 | 3.48 |
| 1379951_at | Shprh | DNA/RNA regulation | 94 | 328 | 0.54 | 1.86 | 3.46 |
| 1370588_a_at | Slc8a1 | Channels/transporters | 71 | 507 | 0.27 | 2.27 | 8.30 |
| 1387134_at | Slfn3 | Unknown function | 74 | 613 | 0.31 | 2.72 | 8.66 |
| 1397709_at | Smg1 | Intracellular signaling | 35 | 109 | 0.55 | 1.69 | 3.09 |
| 1398989_at | Son | Transcriptional regulation | 817 | 2476 | 0.58 | 1.80 | 3.08 |
| 1370838_s_at | Spna2 | Cytoskeleton/myofibrillar | 1516 | 5860 | 0.50 | 1.98 | 3.93 |
| 1371419_at, 1371046_at, 1390706_at | Sptbn1 | Cytoskeleton/myofibrillar | 1761, 629, 570 | 6675, 2505, 2847 | 0.44 | 1.88 | 4.29 |
| 1382978_at, 1393156_at | Tardbp | DNA/RNA regulation | 42, 58 | 146, 226 | 0.53 | 1.96 | 3.72 |
| 1388750_at, 1371113_a_at | Tfrc | Agonists/receptors | 2061, 580 | 6870, 2783 | 0.49 | 1.95 | 3.99 |
| 1369504_at | Tgfbr1 | Agonists/receptors | 58 | 199 | 0.51 | 1.80 | 3.52 |
| 1374529_at, 1394109_at | Thbs1 | Extracellular matrix | 3225, 1042 | 10105, 5478 | 0.25 | 0.97 | 3.85 |
| 1385493_at | Thoc2 | DNA/RNA regulation | 34 | 177 | 0.33 | 2.30 | 7.07 |
| 1397692_at | Tia1 | DNA/RNA regulation | 33 | 96 | 0.56 | 1.72 | 3.05 |
| 1395343_at | Tm9sf3 | Unknown function | 445 | 1342 | 0.55 | 1.67 | 3.04 |
| 1394808_at | Tm9sf4 | Unknown function | 81 | 226 | 0.42 | 1.61 | 3.82 |
| 1373401_at | Tnc | Extracellular matrix | 245 | 1073 | 0.37 | 1.64 | 4.44 |
| 1395871_at | Top2b | DNA/RNA regulation | 149 | 481 | 0.47 | 1.54 | 3.26 |
| 1367976_at | Tpp2 | Proteolysis | 304 | 1013 | 0.54 | 1.86 | 3.43 |
| 1394814_at | Tpr | Trafficking | 16 | 124 | 0.21 | 2.17 | 10.45 |
| 1370198_at | Trdn | Cytoskeleton/myofibrillar | 155 | 457 | 0.60 | 1.82 | 3.02 |
| 1385470_at | Trip11 | Transcriptional regulation | 109 | 338 | 0.57 | 1.79 | 3.14 |
| 1375518_at | Ttn | Cytoskeleton/myofibrillar | 1323 | 4429 | 0.55 | 1.87 | 3.42 |
| 1395647_at | Tufm | Protein synthesis | 66 | 174 | 0.62 | 1.92 | 3.11 |
| 1368953_at | Ugcgl1 | Regulation of metabolism | 124 | 438 | 0.49 | 1.75 | 3.59 |
| 1382280_at | Uqcrc2 | Regulation of metabolism | 1047 | 3589 | 0.64 | 2.16 | 3.40 |
| 1379277_at, 1395132_at | Utrn | Cytoskeleton/myofibrillar | 303, 72 | 1049, 252 | 0.56 | 1.91 | 3.40 |
| 1371232_a_at, 1388142_at | Vcan | Cytoskeleton/myofibrillar | 517, 396 | 1692, 1323 | 0.57 | 1.88 | 3.32 |
| 1398476_at | Vcl | Cytoskeleton/myofibrillar | 230 | 885 | 0.42 | 1.60 | 3.79 |
| 1377778_at | Vof16 | Unknown function | 80 | 327 | 0.63 | 2.32 | 3.69 |
| 1394059_s_at | Yme1l1 | Proteolysis | 256 | 898 | 0.51 | 1.77 | 3.50 |
| 1383462_at | Znf294 | Unknown function | 162 | 527 | 0.57 | 1.86 | 3.27 |
| --- | --- | --- | --- | --- | --- | --- | --- |
| ***Known/probable non-protein-coding*** | | | --- | --- | --- | --- | --- |
| 1379830_at | AS:Chd2 | Potential AS sequences | 22 | 128 | 0.35 | 1.99 | 5.72 |
| 1379712_at | AS:Igf2bp1 | Potential AS sequences | 27 | 141 | 0.34 | 2.09 | 6.08 |
| 1377451_at | AS:Larp4 | Potential AS sequences | 38 | 218 | 0.37 | 2.12 | 5.78 |
| 1381036_at | AS:Slc25a36 | Potential AS sequences | 19 | 202 | 0.22 | 2.85 | 12.95 |
| 1376129_at | AS:Vps13c | Potential AS sequences | 178 | 651 | 0.56 | 2.05 | 3.66 |
| 1375422_at | Ets1 | Non-protein coding | 218 | 8995 | 0.13 | 5.87 | 46.83 |
| 1388985_at | Ets1 | Non-protein coding | 442 | 1762 | 0.60 | 2.20 | 3.65 |
| 1396742_at | Intron:Ranbp5 | Sequences in introns | 59 | 407 | 0.32 | 2.43 | 7.62 |
| 1378866_at | Intron:Ablim1 | Sequences in introns | 118 | 447 | 0.46 | 1.85 | 3.99 |
| 1396098_at | Intron:Acin1 | Sequences in introns | 59 | 250 | 0.43 | 2.00 | 4.66 |
| 1378945_at | Intron:Aco2 | Sequences in introns | 4 | 199 | 0.06 | 3.58 | 56.31 |
| 1396539_at | Intron:Actn1 | Sequences in introns | 38 | 230 | 0.28 | 1.85 | 6.51 |
| 1397389_at | Intron:Adnp | Sequences in introns | 62 | 295 | 0.46 | 2.22 | 4.84 |
| 1380858_at | Intron:Ahdc1 | Sequences in introns | 28 | 152 | 0.36 | 2.03 | 5.61 |
| 1375577_at | Intron:Ak3 | Sequences in introns | 70 | 237 | 0.66 | 2.29 | 3.47 |
| 1394451_at | Intron:Anxa1 | Sequences in introns | 43 | 325 | 0.22 | 1.93 | 8.82 |
| 1381498_at | Intron:Arf4 | Sequences in introns | 14 | 114 | 0.15 | 2.18 | 14.65 |
| 1381151_at | Intron:Arid4b | Sequences in introns | 25 | 88 | 0.48 | 1.97 | 4.09 |
| 1396771_at | Intron:Asxl1 | Sequences in introns | 23 | 147 | 0.32 | 2.04 | 6.36 |
| 1392368_at | Intron:Ate1 | Sequences in introns | 49 | 138 | 0.46 | 1.42 | 3.11 |
| 1381121_at | Intron:Bhlhb2 | Sequences in introns | 60 | 422 | 0.27 | 1.96 | 7.20 |
| 1392325_at | Intron:Btbd4 | Sequences in introns | 13 | 78 | 0.23 | 1.89 | 8.35 |
| 1380358_at | Intron:Caprin1 | Sequences in introns | 34 | 181 | 0.40 | 2.23 | 5.57 |
| 1398549_at | Intron:Ccnl2 | Sequences in introns | 119 | 544 | 0.50 | 2.26 | 4.49 |
| 1397324_at | Intron:Cdk7 | Sequences in introns | 14 | 77 | 0.16 | 1.66 | 10.58 |
| 1397779_at | Intron:Chd2 | Sequences in introns | 32 | 174 | 0.41 | 2.37 | 5.83 |
| 1380430_at | Intron:Copg | Sequences in introns | 14 | 87 | 0.31 | 2.82 | 8.96 |
| 1394689_at | Intron:Csnk1a1 | Sequences in introns | 83 | 271 | 0.57 | 1.87 | 3.26 |
| 1392650_at | Intron:Csnk2a1 | Sequences in introns | 55 | 255 | 0.35 | 2.03 | 5.85 |
| 1390723_at | Intron:Ctnna1 | Sequences in introns | 39 | 201 | 0.25 | 1.52 | 6.06 |
| 1379896_at | Intron:Ddx42 | Sequences in introns | 40 | 130 | 0.47 | 1.64 | 3.48 |
| 1391644_at | Intron:Denr | Sequences in introns | 35 | 122 | 0.52 | 1.76 | 3.36 |
| 1392520_at | Intron:Dlc1 | Sequences in introns | 103 | 326 | 0.62 | 1.95 | 3.15 |
| 1378843_at | Intron:Dnajb6 | Sequences in introns | 25 | 104 | 0.56 | 2.42 | 4.30 |
| 1396562_at | Intron:Dnm2 | Sequences in introns | 44 | 130 | 0.63 | 1.95 | 3.08 |
| 1379116_at | Intron:Drg1 | Sequences in introns | 48 | 142 | 0.53 | 1.65 | 3.09 |
| 1375475_at | Intron:Dusp5 | Sequences in introns | 20 | 64 | 0.32 | 1.07 | 3.40 |
| 1392117_at | Intron:Ebf1 | Sequences in introns | 25 | 94 | 0.42 | 1.53 | 3.62 |
| 1382952_at | Intron:Elavl1 | Sequences in introns | 86 | 356 | 0.41 | 1.78 | 4.29 |
| 1391006_at | Intron:Elavl1 | Sequences in introns | 21 | 101 | 0.35 | 2.05 | 5.86 |
| 1397283_at | Intron:Elf1 | Sequences in introns | 40 | 122 | 0.65 | 1.97 | 3.01 |
| 1391373_at | Intron:Enah | Sequences in introns | 42 | 169 | 0.25 | 1.36 | 5.53 |
| 1397449_at | Intron:Enah | Sequences in introns | 53 | 256 | 0.29 | 1.56 | 5.34 |
| 1392249_at | Intron:Epb4.1l2 | Sequences in introns | 37 | 229 | 0.52 | 3.28 | 6.28 |
| 1382942_at | Intron:Ext1 | Sequences in introns | 30 | 223 | 0.27 | 1.94 | 7.31 |
| 1392567_at | Intron:Fkbp3 | Sequences in introns | 46 | 145 | 0.51 | 1.65 | 3.21 |
| 1377531_at | Intron:Fli1 | Sequences in introns | 36 | 122 | 0.34 | 1.45 | 4.28 |
| 1375054_at | Intron:Fubp1 | Sequences in introns | 32 | 192 | 0.32 | 2.31 | 7.28 |
| 1392818_at | Intron:Gas5 | Sequences in introns | 66 | 600 | 0.18 | 2.26 | 12.84 |
| 1396252_at | Intron:Gnas | Sequences in introns | 56 | 201 | 0.43 | 1.87 | 4.38 |
| 1382982_at | Intron:Gnb1 | Sequences in introns | 14 | 163 | 0.14 | 2.20 | 16.23 |
| 1398736_at | Intron:Golga4 | Sequences in introns | 32 | 147 | 0.44 | 2.08 | 4.68 |
| 1389841_at | Intron:Grb10 | Sequences in introns | 62 | 247 | 0.55 | 2.21 | 4.05 |
| 1391320_at | Intron:Hspbap1 | Sequences in introns | 31 | 102 | 0.69 | 2.42 | 3.50 |
| 1390671_at | Intron:Igf1r | Sequences in introns | 67 | 245 | 0.54 | 1.98 | 3.66 |
| 1395060_at | Intron:Inmt | Sequences in introns | 75 | 261 | 0.48 | 1.97 | 4.06 |
| 1396559_at | Intron:Khdrbs1 | Sequences in introns | 26 | 127 | 0.43 | 2.09 | 4.85 |
| 1396877_at | Intron:Lamc1 | Sequences in introns | 81 | 506 | 0.32 | 2.11 | 6.60 |
| 1380097_at | Intron:Laptm4a | Sequences in introns | 12 | 93 | 0.21 | 2.02 | 9.47 |
| 1394716_at | Intron:Lman2l | Sequences in introns | 36 | 127 | 0.60 | 2.49 | 4.16 |
| 1384692_at | Intron:LOC689323 | Sequences in introns | 28 | 227 | 0.26 | 2.13 | 8.15 |
| 1394283_at | Intron:Luc7l2 | Sequences in introns | 33 | 119 | 0.45 | 2.30 | 5.17 |
| 1383997_at | Intron:Mapk1 | Sequences in introns | 39 | 173 | 0.43 | 2.01 | 4.72 |
| 1382565_at | Intron:Mapk1ip1l | Sequences in introns | 112 | 537 | 0.50 | 2.37 | 4.69 |
| 1381651_at | Intron:March7 | Sequences in introns | 18 | 314 | 0.10 | 2.97 | 29.02 |
| 1377208_at | Intron:Mbnl2 | Sequences in introns | 22 | 104 | 0.31 | 1.83 | 5.83 |
| 1392578_at | Intron:Mgp | Sequences in introns | 45 | 300 | 0.54 | 4.15 | 7.71 |
| 1375573_at | Intron:Mll5 | Sequences in introns | 50 | 178 | 0.60 | 2.20 | 3.67 |
| 1378313_at | Intron:Mthfd2l | Sequences in introns | 78 | 297 | 0.46 | 1.74 | 3.81 |
| 1379089_at | Intron:Myh9 | Sequences in introns | 57 | 331 | 0.28 | 1.77 | 6.26 |
| 1393512_at | Intron:Myh9 | Sequences in introns | 46 | 133 | 0.63 | 2.04 | 3.22 |
| 1382229_at | Intron:Nexn | Sequences in introns | 217 | 554 | 0.48 | 1.58 | 3.29 |
| 1397749_at | Intron:Nf2 | Sequences in introns | 31 | 109 | 0.52 | 1.83 | 3.54 |
| 1378347_at | Intron:Nktr | Sequences in introns | 277 | 812 | 0.45 | 1.47 | 3.30 |
| 1391710_at | Intron:Nme2 | Sequences in introns | 104 | 345 | 0.48 | 1.60 | 3.31 |
| 1377910_at | Intron:Nol5 | Sequences in introns | 28 | 108 | 0.44 | 1.67 | 3.77 |
| 1397004_at | Intron:Nr3c1 | Sequences in introns | 45 | 204 | 0.51 | 2.35 | 4.58 |
| 1381408_at | Intron:Ociad1 | Sequences in introns | 63 | 284 | 0.43 | 1.97 | 4.54 |
| 1378814_at | Intron:Osbpl2 | Sequences in introns | 52 | 229 | 0.50 | 2.28 | 4.57 |
| 1390657_at | Intron:Patl1 | Sequences in introns | 40 | 120 | 0.44 | 1.38 | 3.14 |
| 1379520_at | Intron:Pcnp | Sequences in introns | 179 | 728 | 0.40 | 1.75 | 4.38 |
| 1395842_at | Intron:Postn | Sequences in introns | 36 | 461 | 0.23 | 3.12 | 13.33 |
| 1380020_at | Intron:Ppig | Sequences in introns | 27 | 104 | 0.29 | 1.16 | 4.00 |
| 1392730_at | Intron:Ppp1bc | Sequences in introns | 25 | 210 | 0.23 | 2.79 | 12.32 |
| 1394729_at | Intron:Prkwnk1 | Sequences in introns | 53 | 138 | 0.51 | 1.58 | 3.10 |
| 1381035_at | Intron:Psmc1 | Sequences in introns | 36 | 225 | 0.39 | 2.50 | 6.38 |
| 1397380_at | Intron:Pxk | Sequences in introns | 7 | 109 | 0.31 | 5.10 | 16.56 |
| 1394530_at | Intron:Qrich1 | Sequences in introns | 30 | 98 | 0.72 | 2.35 | 3.28 |
| 1381967_at | Intron:Rbm39 | Sequences in introns | 305 | 1537 | 0.43 | 2.24 | 5.22 |
| 1398595_at | Intron:Rbm5 | Sequences in introns | 64 | 322 | 0.43 | 2.22 | 5.10 |
| 1398560_at | Intron:Rcan2 | Sequences in introns | 33 | 169 | 0.64 | 3.66 | 5.70 |
| 1381714_at | Intron:Rfwd2 | Sequences in introns | 8 | 136 | 0.13 | 2.53 | 19.21 |
| 1385944_at | Intron:RGD1307084 | Sequences in introns | 24 | 200 | 0.25 | 2.56 | 10.21 |
| 1394756_at | Intron:RGD1308297 | Sequences in introns | 32 | 107 | 0.37 | 1.46 | 3.90 |
| 1396115_at | Intron:RGD1309102 | Sequences in introns | 16 | 109 | 0.23 | 1.90 | 8.32 |
| 1397676_at | Intron:RGD1563148 | Sequences in introns | 44 | 242 | 0.35 | 2.29 | 6.54 |
| 1381424_at | Intron:RGD1565775 | Sequences in introns | 29 | 94 | 0.47 | 1.52 | 3.23 |
| 1395092_at | Intron:Rnpc2 | Sequences in introns | 79 | 579 | 0.40 | 2.90 | 7.32 |
| 1376744_at | Intron:Rnpc3 | Sequences in introns | 8 | 109 | 0.12 | 2.17 | 18.43 |
| 1390494_at | Intron:Rps24 | Sequences in introns | 50 | 451 | 0.26 | 2.37 | 9.20 |
| 1380693_at | Intron:Rps6ka3 | Sequences in introns | 27 | 97 | 0.53 | 1.87 | 3.50 |
| 1391788_at | Intron:Sap18 | Sequences in introns | 46 | 226 | 0.45 | 2.16 | 4.85 |
| 1392262_at | Intron:Sars | Sequences in introns | 42 | 118 | 0.47 | 1.52 | 3.23 |
| 1390619_at | Intron:Selo | Sequences in introns | 69 | 222 | 0.44 | 1.78 | 4.02 |
| 1397483_at | Intron:Setd3 | Sequences in introns | 28 | 101 | 0.46 | 1.63 | 3.54 |
| 1382691_at | Intron:Sf3b1 | Sequences in introns | 45 | 420 | 0.41 | 3.94 | 9.58 |
| 1380752_at | Intron:Slain2 | Sequences in introns | 42 | 187 | 0.42 | 1.94 | 4.60 |
| 1380084_at | Intron:Slc25a36 | Sequences in introns | 69 | 229 | 0.68 | 2.19 | 3.24 |
| 1392317_at | Intron:Slc33a1 | Sequences in introns | 23 | 80 | 0.49 | 1.89 | 3.85 |
| 1378223_at | Intron:Smad4 | Sequences in introns | 22 | 109 | 0.51 | 2.54 | 4.95 |
| 1383944_at | Intron:Snx6 | Sequences in introns | 17 | 62 | 0.29 | 1.46 | 4.96 |
| 1382020_at | Intron:Spag9 | Sequences in introns | 109 | 346 | 0.44 | 1.43 | 3.28 |
| 1380701_at | Intron:Ssfa2 | Sequences in introns | 70 | 255 | 0.47 | 1.71 | 3.64 |
| 1392017_at | Intron:Stam2 | Sequences in introns | 20 | 84 | 0.33 | 1.45 | 4.36 |
| 1380644_at | Intron:Taok1 | Sequences in introns | 28 | 499 | 0.18 | 3.86 | 21.63 |
| 1397286_at | Intron:Tcf4 | Sequences in introns | 43 | 188 | 0.53 | 2.30 | 4.36 |
| 1374786_at | Intron:Tia1 | Sequences in introns | 27 | 121 | 0.36 | 2.67 | 7.33 |
| 1395443_at | Intron:Tmem49 | Sequences in introns | 52 | 318 | 0.51 | 3.13 | 6.09 |
| 1395047_at | Intron:Tnks2 | Sequences in introns | 41 | 153 | 0.58 | 2.18 | 3.77 |
| 1397608_at | Intron:Tnrc6a | Sequences in introns | 47 | 219 | 0.57 | 2.80 | 4.86 |
| 1379936_at | Intron:Tpm1 | Sequences in introns | 507 | 1874 | 0.36 | 1.55 | 4.28 |
| 1390471_at | Intron:Tpm1 | Sequences in introns | 274 | 689 | 0.41 | 1.26 | 3.10 |
| 1377762_at | Intron:Tra2a | Sequences in introns | 45 | 271 | 0.29 | 2.11 | 7.30 |
| 1394585_at | Intron:Tra2a | Sequences in introns | 103 | 499 | 0.28 | 1.84 | 6.55 |
| 1398691_at | Intron:Tsc22d2 | Sequences in introns | 12 | 58 | 0.17 | 1.26 | 7.49 |
| 1375031_at | Intron:Ttn | Sequences in introns | 155 | 446 | 0.44 | 1.35 | 3.07 |
| 1380087_at | Intron:Ube2b | Sequences in introns | 79 | 432 | 0.32 | 2.18 | 6.89 |
| 1376438_at | Intron:Ube2e2 | Sequences in introns | 24 | 143 | 0.36 | 2.46 | 6.85 |
| 1383522_at | Intron:Utx | Sequences in introns | 25 | 113 | 0.41 | 1.93 | 4.73 |
| 1381099_at | Intron:Uxt | Sequences in introns | 41 | 129 | 0.50 | 1.57 | 3.13 |
| 1381454_at | Intron:Wdr26 | Sequences in introns | 40 | 150 | 0.48 | 1.77 | 3.65 |
| 1394400_at | Intron:Wdr26 | Sequences in introns | 74 | 413 | 0.37 | 2.10 | 5.65 |
| 1380745_at | Intron:Wtap | Sequences in introns | 46 | 166 | 0.41 | 1.75 | 4.26 |
| 1395130_at | Intron:Zeb2 | Sequences in introns | 42 | 212 | 0.36 | 2.19 | 6.04 |
| 1392846_at | Intron:Zmynd11 | Sequences in introns | 37 | 160 | 0.31 | 1.76 | 5.60 |
| 1372760_at | Malat1 | Non-protein coding | 169 | 1204 | 0.28 | 2.05 | 7.24 |
| 1389986_at | Malat1 | Non-protein coding | 329 | 2183 | 0.33 | 2.40 | 7.19 |
| 1374290_at | Mirn145 precursor | MicroRNA precursor | 87 | 286 | 0.51 | 1.70 | 3.33 |
| 1398059_at | Mirn16-1 precursor | MicroRNA precursor | 25 | 97 | 0.45 | 1.67 | 3.71 |
| 1378867_at | Mirn221 precursor | MicroRNA precursor | 24 | 80 | 0.56 | 1.82 | 3.28 |
| 1391674_at | Mirn24-2 precursor | MicroRNA precursor | 30 | 240 | 0.16 | 1.74 | 10.79 |
| 1385205_at | Mirn99a precursor | MicroRNA precursor | 22 | 287 | 0.27 | 3.57 | 13.06 |
| 1378540_at | Mirnlet7i precursor | MicroRNA precursor | 92 | 499 | 0.49 | 2.71 | 5.47 |
| 1377114_at | Mitochondrial genome | Non-protein coding | 798 | 3017 | 0.51 | 1.93 | 3.78 |
| 1377719_a_at | Mitochondrial genome | Non-protein coding | 1303 | 4732 | 0.53 | 1.94 | 3.65 |
| 1377720_x_at | Mitochondrial genome | Non-protein coding | 1498 | 7369 | 0.46 | 2.26 | 4.94 |
| 1383161_a_at | Mitochondrial genome | Non-protein coding | 1646 | 8116 | 0.48 | 2.36 | 4.92 |
| 1383162_at | Mitochondrial genome | Non-protein coding | 1579 | 7757 | 0.45 | 2.20 | 4.90 |
| 1371595_at | Neat1 | Non-protein coding | 365 | 1756 | 0.32 | 1.56 | 4.89 |
| 1397164_at | Neat1 | Non-protein coding | 123 | 662 | 0.32 | 1.69 | 5.30 |
| 1382882_x_at | Non-coding RNA | Non-protein coding | 9 | 317 | 0.11 | 5.04 | 47.03 |
| 1392166_at | Non-coding RNA | Non-protein coding | 6 | 93 | 0.08 | 2.95 | 34.94 |
| 1374684_at | Xist | Non-protein coding | 52 | 2932 | 0.13 | 7.79 | 60.95 |
| 1375535_at | Xist | Non-protein coding | 92 | 3043 | 0.16 | 5.04 | 32.18 |
| 1377971_at | Xist | Non-protein coding | 51 | 986 | 0.17 | 3.59 | 21.16 |
| 1386785_a_at | Xist | Non-protein coding | 4 | 711 | 0.03 | 8.76 | 252.72 |
| 1386786_at | Xist | Non-protein coding | 7 | 381 | 0.09 | 4.87 | 56.02 |
| --- | --- | --- | --- | --- | --- | --- | --- |
| ***Sequences associated with no known gene*** | | | --- | --- | --- | --- | --- |
| 1372823_at | Unknown | No established gene | 95 | 454 | 0.43 | 2.10 | 4.87 |
| 1372928_at | Unknown | No established gene | 120 | 460 | 0.51 | 2.00 | 3.93 |
| 1373608_at | Unknown | No established gene | 8 | 331 | 0.09 | 3.91 | 43.90 |
| 1373651_at | Unknown | No established gene | 98 | 388 | 0.45 | 1.86 | 4.09 |
| 1374325_at | Unknown | No established gene | 304 | 984 | 0.55 | 1.78 | 3.24 |
| 1374803_at | Unknown | No established gene | 27 | 146 | 0.30 | 2.10 | 7.05 |
| 1375680_at | Unknown | No established gene | 67 | 590 | 0.46 | 3.45 | 7.43 |
| 1375751_at | Unknown | No established gene | 6 | 729 | 0.13 | 10.99 | 86.54 |
| 1375794_at | Unknown | No established gene | 28 | 93 | 0.50 | 1.87 | 3.72 |
| 1376157_at | Unknown | No established gene | 131 | 426 | 0.49 | 1.62 | 3.27 |
| 1376840_at | Unknown | No established gene | 26 | 209 | 0.30 | 2.32 | 7.84 |
| 1377381_at | Unknown | No established gene | 177 | 593 | 0.50 | 1.73 | 3.42 |
| 1377551_at | Unknown | No established gene | 54 | 803 | 0.30 | 4.38 | 14.41 |
| 1378595_at | Unknown | No established gene | 294 | 868 | 0.53 | 1.64 | 3.09 |
| 1378600_at | Unknown | No established gene | 35 | 226 | 0.34 | 2.39 | 6.97 |
| 1379223_x_at | Unknown | No established gene | 162 | 63 | 1.54 | 0.50 | 0.33 |
| 1381112_at | Unknown | No established gene | 58 | 219 | 0.53 | 1.99 | 3.75 |
| 1381210_at | Unknown | No established gene | 28 | 136 | 0.68 | 3.20 | 4.71 |
| 1381267_at | Unknown | No established gene | 56 | 176 | 0.66 | 2.17 | 3.29 |
| 1381612_at | Unknown | No established gene | 57 | 178 | 0.61 | 1.95 | 3.19 |
| 1381920_at | Unknown | No established gene | 43 | 129 | 0.55 | 1.67 | 3.01 |
| 1382755_at | Unknown | No established gene | 72 | 546 | 0.29 | 2.47 | 8.63 |
| 1384006_at | Unknown | No established gene | 17 | 90 | 0.34 | 2.28 | 6.62 |
| 1385506_at | Unknown | No established gene | 65 | 271 | 0.66 | 2.57 | 3.91 |
| 1385533_at | Unknown | No established gene | 26 | 161 | 0.37 | 2.63 | 7.04 |
| 1385701_at | Unknown | No established gene | 121 | 551 | 0.52 | 2.45 | 4.70 |
| 1386038_x_at | Unknown | No established gene | 23 | 141 | 0.32 | 2.90 | 9.16 |
| 1386102_at | Unknown | No established gene | 23 | 347 | 0.26 | 4.02 | 15.75 |
| 1386744_x_at | Unknown | No established gene | 21 | 120 | 0.21 | 2.20 | 10.24 |
| 1390743_at | Unknown | No established gene | 74 | 198 | 0.41 | 1.33 | 3.23 |
| 1391060_at | Unknown | No established gene | 22 | 119 | 0.31 | 1.61 | 5.14 |
| 1391399_at | Unknown | No established gene | 107 | 29 | 1.57 | 0.29 | 0.19 |
| 1391636_at | Unknown | No established gene | 30 | 89 | 0.46 | 1.40 | 3.01 |
| 1391654_at | Unknown | No established gene | 13 | 86 | 0.12 | 1.54 | 13.26 |
| 1391732_at | Unknown | No established gene | 49 | 138 | 0.42 | 1.44 | 3.45 |
| 1391863_at | Unknown | No established gene | 6 | 255 | 0.11 | 5.78 | 53.34 |
| 1392155_at | Unknown | No established gene | 29 | 103 | 0.61 | 2.00 | 3.27 |
| 1392788_at | Unknown | No established gene | 28 | 273 | 0.25 | 3.10 | 12.45 |
| 1392949_at | Unknown | No established gene | 92 | 329 | 0.54 | 1.91 | 3.58 |
| 1393135_at | Unknown | No established gene | 23 | 104 | 0.25 | 1.56 | 6.14 |
| 1393739_at | Unknown | No established gene | 30 | 109 | 0.59 | 2.04 | 3.44 |
| 1394330_at | Unknown | No established gene | 56 | 374 | 0.58 | 3.54 | 6.06 |
| 1394427_at | Unknown | No established gene | 392 | 1472 | 0.49 | 1.85 | 3.79 |
| 1395228_at | Unknown | No established gene | 29 | 232 | 0.25 | 2.47 | 9.77 |
| 1395523_at | Unknown | No established gene | 156 | 636 | 0.53 | 2.15 | 4.08 |
| 1395645_at | Unknown | No established gene | 187 | 592 | 0.41 | 1.50 | 3.65 |
| 1395728_at | Unknown | No established gene | 20 | 139 | 0.18 | 1.58 | 9.03 |
| 1395899_at | Unknown | No established gene | 253 | 1000 | 0.43 | 1.69 | 3.94 |
| 1397316_at | Unknown | No established gene | 22 | 83 | 0.39 | 1.62 | 4.11 |
| 1398213_at | Unknown | No established gene | 80 | 387 | 0.56 | 2.68 | 4.76 |
| 1398695_at | Unknown | No established gene | 21 | 77 | 0.48 | 1.82 | 3.77 |
| 1399079_at | Unknown | No established gene | 2016 | 6282 | 0.62 | 1.95 | 3.13 |
