# Supplementary material for: Regulation of the cardiomyocyte transcriptome vs translatome by endothelin-1 and insulin: translational regulation of 5' terminal oligopyrimidine tract (TOP) mRNAs by insulin
Source: BMC Genomics. 2010 May 29;11:343. doi: 10.1186/1471-2164-11-343 (PMC2900265; doi:10.1186/1471-2164-11-343)
Supplement: Additional file 4 — Regulation of polysomal and total RNA expression in cardiomyocytes in response to insulin (Microsoft Word Table). Neonatal rat cardiomyocytes were exposed to insulin (50 mU/ml, 1 h) or left unstimulated (controls). Total and polysomal RNA were analysed using microarrays. The data were normalised to controls. Transcripts with differential expression in insulin-treated cells relative to controls were identified (>1.25-fold difference; * FDR < 0.05 insulin vs controls for polysomal RNA, # FDR < 0.05 insulin vs controls for total RNA, t-test with Benjamini and Hochberg false discovery rate correction). Mean raw fluorescence values are provided for controls, with mean expression relative to controls for insulin-treated cells (n = 4). For transcripts represented by more than one probeset, the probesets and corresponding raw values are listed. RNAs are listed according to translational regulation and in order of functional category then alphabetical order of the gene symbol. AS = Antisense. [file 1471-2164-11-343-S4.DOC]

**Additional file 4. Regulation of polysomal and total RNA expression in cardiomyocytes in response to insulin.** Neonatal rat cardiomyocytes were exposed to insulin (50 mU/ml, 1 h) or left unstimulated (controls). Total and polysomal RNA were analysed using microarrays. The data were normalised to controls. Transcripts with differential expression in insulin-treated cells relative to controls were identified (>1.25-fold difference; * FDR<0.05 insulin *vs* controls for polysomal RNA, # FDR<0.05 insulin *vs* controls for total RNA, t-test with Benjamini and Hochberg false discovery rate correction). Mean raw fluorescence values are provided for controls, with mean expression relative to controls for insulin-treated cells (n=4). For transcripts represented by more than one probeset, the probesets and corresponding raw values are listed. RNAs are listed according to translational regulation and in order of functional category then alphabetical order of the gene symbol. AS = Antisense.

| **Probeset** | **Gene symbol** | **Gene title** | | | **Function** | **Control**  **(raw values)** | | **Insulin**  **(relative to control)** | |  |
| --- | --- | --- | --- | --- | --- | --- | --- | --- | --- | --- |
|  |  |  |  |  |  | **Polysomal RNA** | **Total RNA** | **Polysomal RNA** | **Total RNA** |  |
| **Translationally regulated (FDR<0.05 in polysomal RNA or total RNA AND ratio of P:T or T:P >1.2-fold** | | | | | | --- | --- | --- | --- |  |
| ***Regulated in polysomal RNA (not total RNA)*** | | | | | | --- | --- | --- | --- |  |
| 1391428_at | Angpt1 | Angiopoietin 1 | | **Agonists/receptors** | | 215 | 387 | 1.34 | 1.01 | * |
| 1380533_at | App | Amyloid beta (A4) precursor protein | | **Agonists/receptors** | | 712 | 3622 | 0.75 | 1.06 | * |
| 1376150_at | Edg3 | Endothelial differentiation, sphingolipid G-protein-coupled receptor, 3 | | **Agonists/receptors** | | 1210 | 1278 | 1.31 | 0.88 | * |
| 1369519_at | Edn1 | Endothelin 1 | | **Agonists/receptors** | | 295 | 276 | 0.70 | 0.93 | * |
| 1381145_at | Egfr | Epidermal growth factor receptor | | **Agonists/receptors** | | 320 | 696 | 0.76 | 0.94 | * |
| 1392664_at | Gpr182 | G protein-coupled receptor 182 | | **Agonists/receptors** | | 440 | 277 | 0.79 | 0.99 | * |
| 1387625_at | Igfbp6 | Insulin-like growth factor binding protein 6 | | **Agonists/receptors** | | 414 | 226 | 0.69 | 0.94 | * |
| 1374678_at | Sema4b | Sema domain, immunoglobulin domain (Ig), transmembrane domain (TM) and short cytoplasmic domain, (semaphorin) 4B | | **Agonists/receptors** | | 325 | 385 | 0.76 | 1.16 | * |
| 1396101_at | Stc1 | Stanniocalcin 1 | | **Agonists/receptors** | | 527 | 656 | 0.80 | 1.02 | * |
| 1387138_at | Tac2 | Tachykinin 2 | | **Agonists/receptors** | | 288 | 329 | 1.27 | 1.00 | * |
| 1374467_at | Trap1 | TNF receptor-associated protein 1 | | **Agonists/receptors** | | 740 | 1376 | 0.71 | 0.94 | * |
| 1395346_at | Aamp | Angio-associated, migratory cell protein | | **Cell-cell/matrix adhesion** | | 1855 | 1217 | 0.71 | 1.00 | * |
| 1384581_at | Actg1 | Actin, gamma, cytoplasmic 1 | | **Cell-cell/matrix adhesion** | | 499 | 536 | 0.79 | 0.99 | * |
| 1383240_at | Itga6 | Integrin, alpha 6 | | **Cell-cell/matrix adhesion** | | 324 | 849 | 0.74 | 0.94 | * |
| 1383880_at | Itgav | Integrin alpha V | | **Cell-cell/matrix adhesion** | | 237 | 494 | 0.68 | 0.97 | * |
| 1372647_at | Prelp | Proline arginine-rich end leucine-rich repeat | | **Cell-cell/matrix adhesion** | | 583 | 571 | 0.80 | 1.03 | * |
| 1373546_at | Atp11a | ATPase, class VI, type 11A | | **Channels/transporters** | | 536 | 506 | 0.74 | 0.93 | * |
| 1370207_at | Atp5g2 | ATP synthase, H+ transporting, mitochondrial F0 complex, subunit C2 (subunit 9) | | **Channels/transporters** | | 7370 | 6039 | 1.26 | 1.00 | * |
| 1399000_at | Atp9b | ATPase, class II, type 9B | | **Channels/transporters** | | 319 | 352 | 0.75 | 1.11 | * |
| 1379307_at | Sclt1 | Sodium channel and clathrin linker 1 | | **Channels/transporters** | | 255 | 267 | 1.28 | 1.05 | * |
| 1373282_at | Slc25a33 | Solute carrier family 25, member 33 | | **Channels/transporters** | | 308 | 253 | 1.28 | 0.94 | * |
| 1384302_at | Slc6a17 | Solute carrier family 6 (neurotransmitter transporter), member 17 | | **Channels/transporters** | | 333 | 650 | 1.33 | 0.97 | * |
| 1379206_at | Tmem38a | Transmembrane protein 38a | | **Channels/transporters** | | 1240 | 1559 | 0.79 | 0.99 | * |
| 1389294_at | Cyfip1 | Cytoplasmic FMR1 interacting protein 1 | | **Cytoskeleton/myofibrillar** | | 1607 | 2625 | 0.74 | 0.89 | * |
| 1368404_at | Dbn1 | Drebrin 1 | | **Cytoskeleton/myofibrillar** | | 669 | 1150 | 0.79 | 1.09 | * |
| 1372514_s_at | Dnalc4 | Dynein, axonemal, light chain 4 | | **Cytoskeleton/myofibrillar** | | 848 | 666 | 1.38 | 1.07 | * |
| 1371382_at | Flna | Filamin, alpha | | **Cytoskeleton/myofibrillar** | | 2282 | 5607 | 0.73 | 0.98 | * |
| 1391063_at | Kif23 | Kinesin family member 23 | | **Cytoskeleton/myofibrillar** | | 344 | 399 | 0.72 | 0.99 | * |
| 1376185_at | Kifc1 | Kinesin family member C1 | | **Cytoskeleton/myofibrillar** | | 459 | 395 | 0.79 | 0.98 | * |
| 1381992_at | Kptn | Kaptin (actin binding protein) | | **Cytoskeleton/myofibrillar** | | 618 | 450 | 0.76 | 0.97 | * |
| 1372737_at | Ldb3 | LIM domain binding 3 | | **Cytoskeleton/myofibrillar** | | 1094 | 1232 | 1.41 | 0.97 | * |
| 1392361_at | Lpp | LIM domain containing preferred translocation partner in lipoma | | **Cytoskeleton/myofibrillar** | | 409 | 298 | 0.73 | 0.99 | * |
| 1371940_at | Macf1 | Microtubule-actin crosslinking factor 1 | | **Cytoskeleton/myofibrillar** | | 700 | 2267 | 1.27 | 0.86 | * |
| 1370949_at | Marcks | Myristoylated alanine-rich C-kinase substrate | | **Cytoskeleton/myofibrillar** | | 2166 | 2358 | 0.79 | 1.07 | * |
| 1368948_at | Msn | Moesin | | **Cytoskeleton/myofibrillar** | | 343 | 912 | 0.75 | 1.18 | * |
| 1376722_at | Nup205 | Nucleoporin 205kDa | | **Cytoskeleton/myofibrillar** | | 397 | 709 | 0.74 | 1.05 | * |
| 1370266_at | Parva | Parvin, alpha | | **Cytoskeleton/myofibrillar** | | 616 | 764 | 0.74 | 1.00 | * |
| 1370838_s_at | Spna2 | Alpha-spectrin 2 | | **Cytoskeleton/myofibrillar** | | 1516 | 5860 | 0.76 | 0.93 | * |
| 1371695_at | Tpr | Translocated promoter region | | **Cytoskeleton/myofibrillar** | | 350 | 824 | 0.77 | 1.02 | * |
| 1380448_at | Alkbh | AlkB, alkylation repair homolog | | **DNA structure/repair** | | 1096 | 1079 | 1.33 | 1.10 | * |
| 1383050_at | Cenpv | Centromere protein V | | **DNA structure/repair** | | 329 | 209 | 0.72 | 1.11 | * |
| 1371648_at, 1399162_a_at | Ddb1 | Damage specific DNA binding protein 1 | | **DNA structure/repair** | | 2962, 1556 | 4516, 2842 | 0.74 | 0.92 | * |
| 1376676_a_at | Mphosph8 | M-phase phosphoprotein 8 | | **DNA structure/repair** | | 1191 | 1957 | 0.78 | 0.98 | * |
| 1390098_at | N6amt2 | N-6 adenine-specific DNA methyltransferase 2 | | **DNA structure/repair** | | 477 | 395 | 1.30 | 0.98 | * |
| 1368012_at | Tep1 | Telomerase associated protein 1 | | **DNA structure/repair** | | 483 | 916 | 0.75 | 0.92 | * |
| 1376105_at | Col14a1 | Procollagen, type XIV, alpha 1 | | **Extracellular matrix** | | 1844 | 3676 | 0.69 | 1.02 | * |
| 1371893_at | Col4a3bp | Procollagen, type IV, alpha 3 (Goodpasture antigen) binding protein | | **Extracellular matrix** | | 1063 | 1633 | 1.26 | 0.96 | * |
| 1370234_at | Fn1 | Fibronectin 1 | | **Extracellular matrix** | | 4364 | 14991 | 0.75 | 0.95 | * |
| 1372263_at, 1389713_at, 1399155_at | LOC296637 | Similar to HLA-B associated transcript-2 isoform a | | **Hypothetical protein** | | 451, 1441, 268 | 839, 2221, 500 | 0.75 | 1.12 | * |
| 1376661_at | LOC313672 | Kazrin | | **Hypothetical protein** | | 376 | 294 | 1.27 | 1.02 | * |
| 1371735_at | LOC682880 | Hypothetical protein LOC682880 | | **Hypothetical protein** | | 1239 | 1704 | 1.53 | 0.93 | * |
| 1397768_at | LOC686796 | Hypothetical protein LOC686796 | | **Hypothetical protein** | | 212 | 192 | 1.26 | 1.01 | * |
| 1380072_at | MGC93975 | similar to 2310044H10Rik protein | | **Hypothetical protein** | | 745 | 558 | 0.73 | 1.00 | * |
| 1378642_at | RGD1307071 | Similar to chromosome 20 open reading frame 19 | | **Hypothetical protein** | | 224 | 213 | 1.53 | 1.06 | * |
| 1379425_at | RGD1309802 | Similar to RIKEN cDNA 3110037I16 | | **Hypothetical protein** | | 329 | 191 | 0.71 | 0.88 | * |
| 1393015_at | RGD1310587 | Similar to hypothetical protein FLJ14146 | | **Hypothetical protein** | | 914 | 459 | 0.79 | 1.00 | * |
| 1397838_at | RGD1564792 | RGD1564792 | | **Hypothetical protein** | | 370 | 396 | 0.73 | 0.95 | * |
| 1392497_at, 1395064_at | RGD1564943 | Similar to 4930429A08Rik protein | | **Hypothetical protein** | | 583, 623 | 381, 492 | 0.75 | 0.98 | * |
| 1374325_at | Unknown | Unknown | | **No established gene** | | 304 | 984 | 0.72 | 1.10 | * |
| 1374339_at | Unknown | Unknown | | **No established gene** | | 1071 | 943 | 1.29 | 0.97 | * |
| 1379268_at | Unknown | Unknown | | **No established gene** | | 1148 | 616 | 0.75 | 0.93 | * |
| 1380470_x_at | Unknown | Unknown | | **No established gene** | | 1632 | 1156 | 1.35 | 1.05 | * |
| 1381428_a_at | Unknown | Unknown | | **No established gene** | | 1295 | 845 | 1.38 | 1.04 | * |
| 1383955_at | Unknown | Unknown | | **No established gene** | | 332 | 276 | 0.66 | 0.85 | * |
| 1384582_at | Unknown | Unknown | | **No established gene** | | 531 | 502 | 1.34 | 1.11 | * |
| 1388203_x_at | Unknown | Unknown | | **No established gene** | | 323 | 223 | 0.76 | 1.29 | * |
| 1389716_at | Unknown | Unknown | | **No established gene** | | 329 | 263 | 1.30 | 0.78 | * |
| 1390824_at | Unknown | Unknown | | **No established gene** | | 353 | 286 | 0.74 | 1.02 | * |
| 1392531_at | Unknown | Unknown | | **No established gene** | | 539 | 395 | 1.26 | 0.97 | * |
| 1392842_at | Unknown | Unknown | | **No established gene** | | 428 | 542 | 0.76 | 1.02 | * |
| 1392947_at | Unknown | Unknown | | **No established gene** | | 763 | 770 | 0.67 | 0.84 | * |
| 1394480_at | Unknown | Unknown | | **No established gene** | | 231 | 207 | 0.80 | 1.01 | * |
| 1395119_at | Unknown | Unknown | | **No established gene** | | 224 | 221 | 1.57 | 1.08 | * |
| 1377015_at | AS:Me | AS:malic enzyme 3, NADP(+)-dependent, mitochondrial | | **Non-protein coding** | | 796 | 839 | 0.77 | 0.96 | * |
| 1382015_at | Intron:Cdc6 | Intron:Cdc6 | | **Non-protein coding** | | 398 | 345 | 1.31 | 1.03 | * |
| 1397023_at | Intron:Ppp5c | Intron:protein phosphatase 5, catalytic subunit | | **Non-protein coding** | | 440 | 428 | 0.78 | 1.09 | * |
| 1377719_a_at | Mitochondrial genome | D-loop, partial sequence; mitochondrial | | **Non-protein coding** | | 1303 | 4732 | 0.69 | 0.88 | * |
| 1375022_at | Afg3l2 | AFG3(ATPase family gene 3)-like 2 (yeast) | | **Protein processing** | | 318 | 723 | 1.29 | 0.92 | * |
| 1379422_at | Alg13 | Asparagine-linked glycosylation 13 homolog | | **Protein processing** | | 410 | 332 | 1.26 | 1.03 | * |
| 1398815_at | Apeh | N-acylaminoacyl-peptide hydrolase | | **Protein processing** | | 1093 | 1307 | 0.80 | 0.99 | * |
| 1397670_at | Atg12 | Autophagy-related 12 (yeast) | | **Protein processing** | | 477 | 471 | 1.28 | 1.03 | * |
| 1370215_at | C1qb | Complement component 1, q subcomponent, beta polypeptide | | **Protein processing** | | 260 | 241 | 0.73 | 1.03 | * |
| 1373111_at | Capn7 | Calpain7 | | **Protein processing** | | 1127 | 1381 | 1.26 | 0.91 | * |
| 1384192_at | Chst1 | Carbohydrate (keratan sulfate Gal-6) sulfotransferase 1 | | **Protein processing** | | 1568 | 623 | 0.67 | 0.87 | * |
| 1378507_at | Dtl | Denticleless homolog | | **Protein processing** | | 185 | 254 | 1.30 | 0.97 | * |
| 1372776_at | Fbxl5 | F-box and leucine-rich repeat protein 5 | | **Protein processing** | | 1786 | 1599 | 1.29 | 1.04 | * |
| 1369972_at | Fbxo21 | F-box protein 21 | | **Protein processing** | | 412 | 383 | 0.77 | 0.98 | * |
| 1368314_at | Ggcx | Gamma-glutamyl carboxylase | | **Protein processing** | | 674 | 1305 | 0.75 | 0.95 | * |
| 1380474_at, 1391450_at | Loxl2 | Lysyl oxidase-like 2 | | **Protein processing** | | 271, 995 | 489, 1274 | 0.65 | 0.99 | * |
| 1398884_at | Pfdn5 | Prefoldin 5 | | **Protein processing** | | 3287 | 2665 | 1.28 | 0.90 | * |
| 1387781_at | Pmpcb | Peptidase (mitochondrial processing) beta | | **Protein processing** | | 983 | 2016 | 0.69 | 0.94 | * |
| 1382379_at | Rnf138 | Ring finger protein 138 | | **Protein processing** | | 555 | 604 | 1.30 | 1.03 | * |
| 1387408_at | Siah2 | Seven in absentia 2 | | **Protein processing** | | 262 | 228 | 1.39 | 1.00 | * |
| 1378640_at | Uhrf1 | Ubiquitin-like with PHD and ring finger domains 1 | | **Protein processing** | | 295 | 416 | 0.79 | 1.00 | * |
| 1389022_at | Usp52 | Ubiquitin specific peptidase 52 | | **Protein processing** | | 329 | 411 | 0.79 | 1.04 | * |
| 1397487_at | Xpnpep3 | X-prolyl aminopeptidase (aminopeptidase P) 3, putative | | **Protein processing** | | 433 | 332 | 0.62 | 1.21 | * |
| 1378088_at | Zdhhc1 | Zinc finger, DHHC domain containing 1 | | **Protein processing** | | 310 | 275 | 0.78 | 0.94 | * |
| 1367560_at | Arbp | Acidic ribosomal phosphoprotein P0 | | **Protein synthesis** | | 12115 | 20682 | 1.25 | 0.91 | * |
| 1388449_at | Eef1b2 | Eukaryotic translation elongation factor 1 beta 2 | | **Protein synthesis** | | 10253 | 13607 | 1.27 | 0.98 | * |
| 1387658_at | Eef2k | Eukaryotic elongation factor-2 kinase | | **Protein synthesis** | | 396 | 286 | 1.43 | 1.12 | * |
| 1376145_at | Eif2b5 | Eukaryotic translation initiation factor 2B, subunit 5 epsilon | | **Protein synthesis** | | 230 | 229 | 0.73 | 0.92 | * |
| 1373040_at | Eif3f | Eukaryotic translation initiation factor 3, subunit F | | **Protein synthesis** | | 5967 | 7439 | 1.28 | 0.93 | * |
| 1389968_at | Eif3s10 | eukaryotic translation initiation factor 3, subunit 10 (theta) | | **Protein synthesis** | | 1382 | 3581 | 0.80 | 1.02 | * |
| 1383867_at, 1393063_at | Eif5a2 | Eukaryotic translation initiation factor 5A2 | | **Protein synthesis** | | 612, 223 | 487, 224 | 1.35 | 1.09 | * |
| 1371316_at | Fau | Finkel-Biskis-Reilly murine sarcoma virus (FBR-MuSV) ubiquitously expressed (fox derived) protein (Rps30) | | **Protein synthesis** | | 10982 | 13277 | 1.32 | 0.92 | * |
| 1375135_at | Gcn1l1 | GCN1 general control of amino-acid synthesis 1-like 1 | | **Protein synthesis** | | 364 | 530 | 0.74 | 1.08 | * |
| 1383157_at | Mrpl44 | Mitochondrial ribosomal protein L44 | | **Protein synthesis** | | 628 | 618 | 1.28 | 0.89 | * |
| 1372215_at | Mrps11 | Mitochondrial ribosomal protein S11 | | **Protein synthesis** | | 1201 | 498 | 0.78 | 1.04 | * |
| 1390813_at | Msi2 | Musashi homolog 2 | | **Protein synthesis** | | 432 | 232 | 1.42 | 1.17 | * |
| 1383666_at | Ptrh1 | Peptidyl-tRNA hydrolase 1 homolog | | **Protein synthesis** | | 276 | 203 | 1.36 | 0.80 | * |
| 1383831_at | Qrsl1 | Glutaminyl-tRNA synthase (glutamine-hydrolyzing)-like 1 | | **Protein synthesis** | | 381 | 496 | 0.78 | 0.95 | * |
| 1367580_at | Rpl10a | Ribosomal protein L10A | | **Protein synthesis** | | 10458 | 13265 | 1.29 | 0.88 | * |
| 1375181_at | Rpl12 | Ribosomal protein L12 | | **Protein synthesis** | | 12107 | 16780 | 1.36 | 0.91 | * |
| 1386858_at | Rpl13 | Ribosomal protein L13 | | **Protein synthesis** | | 11354 | 14268 | 1.34 | 0.90 | * |
| 1387887_at | Rpl14 | Ribosomal protein L14 | | **Protein synthesis** | | 10911 | 13678 | 1.31 | 0.95 | * |
| 1398315_at | Rpl15 | Ribosomal protein L15 | | **Protein synthesis** | | 12489 | 14922 | 1.26 | 0.95 | * |
| 1398871_at | Rpl17 | Ribosomal protein L17 | | **Protein synthesis** | | 11968 | 15219 | 1.33 | 0.92 | * |
| 1367623_at | Rpl18 | Ribosomal protein L18 | | **Protein synthesis** | | 14252 | 15067 | 1.25 | 0.92 | * |
| 1398324_at | Rpl18a | Ribosomal protein L18a | | **Protein synthesis** | | 14800 | 18894 | 1.27 | 0.95 | * |
| 1370253_at | Rpl22 | Ribosomal protein L22 | | **Protein synthesis** | | 13987 | 15132 | 1.34 | 0.95 | * |
| 1388989_at | Rpl22l1 | Ribosomal protein L22 like 1 | | **Protein synthesis** | | 6528 | 8488 | 1.29 | 0.99 | * |
| 1398885_at | Rpl23 | Ribosomal protein L23 | | **Protein synthesis** | | 14730 | 17305 | 1.27 | 0.96 | * |
| 1388303_at | Rpl26 | Ribosomal protein L26 | | **Protein synthesis** | | 15973 | 20713 | 1.29 | 0.95 | * |
| 1367561_at | Rpl27 | Ribosomal protein L27 | | **Protein synthesis** | | 14914 | 17966 | 1.25 | 0.90 | * |
| 1398830_at | Rpl28 | Ribosomal protein L28 | | **Protein synthesis** | | 14564 | 16016 | 1.30 | 0.95 | * |
| 1367582_at | Rpl29 | Ribosomal protein L29 | | **Protein synthesis** | | 10887 | 13332 | 1.30 | 0.93 | * |
| 1367634_at | Rpl31 | Ribosomal protein L31 | | **Protein synthesis** | | 15859 | 19904 | 1.27 | 0.93 | * |
| 1384548_at | Rpl32 | Ribosomal protein L32 | | **Protein synthesis** | | 15481 | 19917 | 1.29 | 0.91 | * |
| 1371761_at | Rpl34 | Ribosomal protein L34 | | **Protein synthesis** | | 13240 | 16068 | 1.32 | 0.95 | * |
| 1388372_at | Rpl35 | Ribosomal protein L35 | | **Protein synthesis** | | 11004 | 13216 | 1.41 | 0.97 | * |
| 1398760_at | Rpl35a | Ribosomal protein L35a | | **Protein synthesis** | | 10750 | 14919 | 1.35 | 0.94 | * |
| 1371573_at | Rpl36a | Ribosomal protein L36a | | **Protein synthesis** | | 7985 | 11013 | 1.48 | 0.91 | * |
| 1367934_at | Rpl39 | Ribosomal protein L39 | | **Protein synthesis** | | 546 | 785 | 0.79 | 1.13 | * |
| 1398749_at | Rpl4 | Ribosomal protein L4 | | **Protein synthesis** | | 10465 | 14655 | 1.33 | 0.94 | * |
| 1398917_at | Rpl7 | Ribosomal protein L7 | | **Protein synthesis** | | 13696 | 18289 | 1.30 | 0.93 | * |
| 1371305_at | Rpl8 | Ribosomal protein L8 | | **Protein synthesis** | | 12381 | 14361 | 1.27 | 0.95 | * |
| 1371301_at | Rpl9 | Ribosomal protein L9 | | **Protein synthesis** | | 16687 | 22100 | 1.27 | 0.91 | * |
| 1371340_at | Rplp2 | Ribosomal protein, large P2 | | **Protein synthesis** | | 12373 | 14524 | 1.30 | 0.96 | * |
| 1386868_at | Rps10 | Ribosomal protein S10 | | **Protein synthesis** | | 10700 | 11394 | 1.28 | 1.03 | * |
| 1367630_at | Rps11 | Ribosomal protein S11 | | **Protein synthesis** | | 12567 | 14959 | 1.35 | 0.92 | * |
| 1367640_at | Rps12 | Ribosomal protein S12 | | **Protein synthesis** | | 14107 | 19633 | 1.31 | 0.92 | * |
| 1368211_at | Rps14 | Ribosomal protein S14 | | **Protein synthesis** | | 15262 | 19724 | 1.27 | 0.92 | * |
| 1386874_at | Rps15 | Ribosomal protein S15 | | **Protein synthesis** | | 10445 | 13062 | 1.30 | 0.93 | * |
| 1371318_at | Rps16 | Ribosomal protein S16 | | **Protein synthesis** | | 12846 | 16946 | 1.30 | 0.89 | * |
| 1388296_at | Rps18 | Ribosomal protein S18 | | **Protein synthesis** | | 14887 | 18119 | 1.26 | 0.93 | * |
| 1371377_at | Rps19 | Ribosomal protein S19 | | **Protein synthesis** | | 13953 | 19232 | 1.32 | 0.91 | * |
| 1371295_at | Rps20 | Ribosomal protein S20 | | **Protein synthesis** | | 12201 | 16102 | 1.36 | 0.93 | * |
| 1370242_at | Rps23 | Ribosomal protein S23 | | **Protein synthesis** | | 19085 | 22493 | 1.27 | 0.98 | * |
| 1369966_a_at | Rps24 | Ribosomal protein S24 | | **Protein synthesis** | | 13412 | 17064 | 1.32 | 0.95 | * |
| 1388313_at | Rps25 | Ribosomal protein s25 | | **Protein synthesis** | | 5090 | 6886 | 1.30 | 0.84 | * |
| 1367596_at | Rps26 | Ribosomal protein S26 | | **Protein synthesis** | | 12954 | 16664 | 1.33 | 0.93 | * |
| 1371299_at | Rps3 | Ribosomal protein S3 | | **Protein synthesis** | | 12239 | 14579 | 1.33 | 0.96 | * |
| 1398882_at | Rps5 | Ribosomal protein S5 | | **Protein synthesis** | | 13544 | 17809 | 1.27 | 0.90 | * |
| 1398751_at | Rps7 | Ribosomal protein S7 | | **Protein synthesis** | | 10647 | 15663 | 1.35 | 0.95 | * |
| 1367597_at | Rps8 | Ribosomal protein S8 | | **Protein synthesis** | | 17027 | 20077 | 1.25 | 1.00 | * |
| 1387888_at | Rps9 | Ribosomal protein S9 | | **Protein synthesis** | | 13057 | 13949 | 1.29 | 0.97 | * |
| 1367569_at | Rpsa | Ribosomal protein SA | | **Protein synthesis** | | 9913 | 14338 | 1.26 | 0.93 | * |
| 1388413_at | Rrbp1 | Ribosome binding protein 1 homolog 180kDa | | **Protein synthesis** | | 819 | 2104 | 0.62 | 1.03 | * |
| 1396167_at | Acp6 | Acid phosphatase 6, lysophosphatidic | | **Regulation of metabolism** | | 520 | 548 | 0.73 | 0.92 | * |
| 1367999_at | Aldh2 | Aldehyde dehydrogenase 2 | | **Regulation of metabolism** | | 1746 | 2876 | 0.65 | 0.97 | * |
| 1380525_at | Arse | Arylsulfatase E (chondrodysplasia punctata 1) | | **Regulation of metabolism** | | 499 | 500 | 0.75 | 1.02 | * |
| 1388290_at | Cox6a1 | Cytochrome c oxidase, subunit VIa, polypeptide 1 | | **Regulation of metabolism** | | 1295 | 1250 | 0.77 | 0.96 | * |
| 1397218_at | Dpysl3 | Dihydropyrimidinase-like 3 | | **Regulation of metabolism** | | 364 | 590 | 0.76 | 1.11 | * |
| 1385072_at | Galm | Galactose mutarotase (aldose 1-epimerase) | | **Regulation of metabolism** | | 634 | 445 | 0.80 | 0.97 | * |
| 1398990_at | Glo1 | Glyoxalase 1 | | **Regulation of metabolism** | | 1702 | 1179 | 1.29 | 1.03 | * |
| 1367806_at | Gls | Glutaminase | | **Regulation of metabolism** | | 309 | 508 | 0.68 | 0.92 | * |
| 1370164_at | Hadha | Hydroxyacyl-Coenzyme A dehydrogenase | | **Regulation of metabolism** | | 1071 | 3244 | 0.71 | 0.89 | * |
| 1388629_at | Impdh2 | Inosine 5-monophosphate dehydrogenase 2 | | **Regulation of metabolism** | | 4092 | 5456 | 1.26 | 0.89 | * |
| 1380071_at | Parp12 | Poly (ADP-ribose) polymerase family, member 12 | | **Regulation of metabolism** | | 627 | 645 | 0.73 | 0.95 | * |
| 1375213_at, 1376247_at | Pck2 | Phosphoenolpyruvate carboxykinase 2 (mitochondrial) | | **Regulation of metabolism** | | 937, 379 | 1179, 432 | 0.64 | 1.13 | * |
| 1382724_at | Pigo | Phosphatidylinositol glycan anchor biosynthesis, class O | | **Regulation of metabolism** | | 289 | 332 | 0.72 | 0.99 | * |
| 1389347_at | Pitpnm1 | Phosphatidylinositol transfer protein, membrane-associated 1 | | **Regulation of metabolism** | | 793 | 662 | 0.78 | 0.95 | * |
| 1384875_at | Rdh11 | Retinol dehydrogenase 11 (all-trans/9-cis/11-cis) | | **Regulation of metabolism** | | 184 | 264 | 1.31 | 1.02 | * |
| 1386535_at | Ddx3x | DEAD/H (Asp-Glu-Ala-Asp/His) box polypeptide 3, X-linked | | **RNA regulation** | | 621 | 1534 | 0.54 | 1.08 | * |
| 1370919_at | Hnrnpm | Heterogeneous nuclear ribonucleoprotein M | | **RNA regulation** | | 927 | 1097 | 0.78 | 1.04 | * |
| 1370171_at | Hnrnpu | Heterogeneous nuclear ribonucleoprotein U | | **RNA regulation** | | 1351 | 1468 | 0.74 | 1.38 | * |
| 1375019_at | Hnrph3 | Heterogeneous nuclear ribonucleoprotein H3 (2H9) | | **RNA regulation** | | 431 | 404 | 1.34 | 1.03 | * |
| 1379407_at | Imp4 | IMP4, U3 small nucleolar ribonucleoprotein, homolog | | **RNA regulation** | | 234 | 172 | 1.28 | 0.91 | * |
| 1399158_a_at | Npm1 | Nucleophosmin 1 | | **RNA regulation** | | 10083 | 9932 | 1.26 | 1.00 | * |
| 1379317_a_at | Pdcd11 | Programmed cell death 11 | | **RNA regulation** | | 455 | 927 | 0.72 | 0.92 | * |
| 1396185_at | Rbm17 | RNA binding motif protein 17 | | **RNA regulation** | | 368 | 236 | 0.78 | 1.11 | * |
| 1377787_at | Rbm6 | RNA binding motif protein 6 | | **RNA regulation** | | 380 | 545 | 0.79 | 1.02 | * |
| 1390032_at | Rbms2 | RNA binding motif, single stranded interacting protein 2 | | **RNA regulation** | | 1230 | 1663 | 1.27 | 0.96 | * |
| 1374063_at | Sfrs3 | Splicing factor, arginine/serine-rich 3 (SRp20) | | **RNA regulation** | | 2448 | 2670 | 1.30 | 1.08 | * |
| 1368992_a_at | Sfrs5 | Splicing factor, arginine/serine-rich 5 | | **RNA regulation** | | 2390 | 3568 | 1.48 | 0.98 | * |
| 1391387_s_at | Slbp | stem-loop binding protein | | **RNA regulation** | | 498 | 446 | 0.72 | 0.92 | * |
| 1395402_at | Smg5 | Smg-5 homolog, nonsense mediated mRNA decay factor | | **RNA regulation** | | 309 | 265 | 0.63 | 1.22 | * |
| 1371341_at | Snrpd2 | Small nuclear ribonucleoprotein D2 | | **RNA regulation** | | 5281 | 4089 | 1.38 | 0.94 | * |
| 1383660_at | Thoc4 | THO complex 4 | | **RNA regulation** | | 395 | 379 | 1.40 | 1.03 | * |
| 1388305_at | Araf | v-raf murine sarcoma 3611 viral oncogene homolog | | **Signalling/Trafficking** | | 1257 | 1069 | 0.68 | 0.91 | * |
| 1384797_at | Atl3 | Atlastin GTPase 3 | | **Signalling/Trafficking** | | 378 | 565 | 0.68 | 0.92 | * |
| 1390960_at | Azi2 | 5-azacytidine induced gene 2 | | **Signalling/Trafficking** | | 242 | 288 | 1.44 | 1.01 | * |
| 1390637_at | Bnip2 | BCL2/adenovirus E1B 19kDa interacting protein 2 | | **Signalling/Trafficking** | | 643 | 808 | 1.30 | 0.86 | * |
| 1380997_at | Ccnj | Cyclin J | | **Signalling/Trafficking** | | 182 | 202 | 1.52 | 1.20 | * |
| 1377213_at | Ern1 | Endoplasmic reticulum (ER) to nucleus signalling 1 | | **Signalling/Trafficking** | | 357 | 550 | 1.26 | 0.93 | * |
| 1378253_at | Exoc8 | Exocyst complex component 8 | | **Signalling/Trafficking** | | 461 | 528 | 0.79 | 1.11 | * |
| 1377661_at | Frs2 | Fibroblast growth factor receptor substrate 2 | | **Signalling/Trafficking** | | 640 | 648 | 1.34 | 1.08 | * |
| 1388792_at | Gadd45g | Growth arrest and DNA-damage-inducible 45 gamma | | **Signalling/Trafficking** | | 1899 | 1419 | 1.40 | 1.05 | * |
| 1371471_at | Gltscr2 | Glioma tumor suppressor candidate region gene 2 | | **Signalling/Trafficking** | | 2120 | 2339 | 1.28 | 0.91 | * |
| 1367618_a_at | Gnb2l1 | Guanine nucleotide binding protein, beta polypeptide 2 like 1 | | **Signalling/Trafficking** | | 12421 | 15646 | 1.29 | 0.97 | * |
| 1389556_at | Hus1 | Hus1 homolog | | **Signalling/Trafficking** | | 684 | 867 | 0.79 | 0.95 | * |
| 1387777_at | Ilk | Integrin linked kinase | | **Signalling/Trafficking** | | 4230 | 3785 | 1.35 | 0.98 | * |
| 1374462_at | Kifap3 | Kinesin-associated protein 3 | | **Signalling/Trafficking** | | 555 | 882 | 0.77 | 0.93 | * |
| 1373500_at | Lrpprc | Leucine-rich PPR-motif containing | | **Signalling/Trafficking** | | 662 | 1715 | 0.75 | 0.92 | * |
| 1371435_at | Naca | Nascent-polypeptide-associated complex alpha polypeptide | | **Signalling/Trafficking** | | 11006 | 11046 | 1.25 | 0.96 | * |
| 1383960_at | Pex16 | Peroxisome biogenesis factor 16 | | **Signalling/Trafficking** | | 625 | 523 | 0.74 | 0.95 | * |
| 1389076_at | Pkn3 | Protein kinase N3 | | **Signalling/Trafficking** | | 526 | 463 | 0.70 | 1.10 | * |
| 1390395_at | Plekha8 | Pleckstrin homology domain containing, family A (phosphoinositide binding specific) member 8 | | **Signalling/Trafficking** | | 347 | 434 | 1.29 | 1.01 | * |
| 1376060_at | Ppm1h | Protein phosphatase 1H (PP2C domain containing) | | **Signalling/Trafficking** | | 254 | 220 | 0.80 | 1.06 | * |
| 1368087_a_at | Ptpn21 | Protein tyrosine phosphatase, non-receptor type 21 | | **Signalling/Trafficking** | | 445 | 573 | 1.25 | 1.03 | * |
| 1370780_at | Rab31 | RAB31, member RAS oncogene family | | **Signalling/Trafficking** | | 409 | 529 | 0.79 | 1.18 | * |
| 1388477_at | Ranbp3 | RAN binding protein 3 | | **Signalling/Trafficking** | | 1399 | 944 | 0.80 | 0.99 | * |
| 1372875_at | Rcan | Regulator of calcineurin 3 | | **Signalling/Trafficking** | | 848 | 538 | 0.79 | 0.98 | * |
| 1385596_at | Tbc1d15 | TBC1 domain family, member 15 | | **Signalling/Trafficking** | | 1030 | 1537 | 0.69 | 0.86 | * |
| 1379429_at | Tmed5 | Transmembrane emp24 protein transport domain containing 5 | | **Signalling/Trafficking** | | 575 | 923 | 1.28 | 1.02 | * |
| 1399052_at | Tollip | Toll interacting protein | | **Signalling/Trafficking** | | 1021 | 766 | 1.33 | 1.01 | * |
| 1391643_at | Trib1 | Tribbles homolog 1 (Drosophila) | | **Signalling/Trafficking** | | 1357 | 1036 | 1.26 | 0.99 | * |
| 1383981_at | Trp53bp2 | Transformation related protein 53 binding protein 2 | | **Signalling/Trafficking** | | 370 | 599 | 0.79 | 1.01 | * |
| 1387557_s_at | Vps33a | Vacuolar protein sorting 33A (yeast) | | **Signalling/Trafficking** | | 525 | 509 | 0.62 | 0.81 | * |
| 1389644_at | Wdr67 | WD repeat domain 67 | | **Signalling/Trafficking** | | 325 | 267 | 0.70 | 0.96 | * |
| 1397312_at | Xpo7 | Exportin 7 | | **Signalling/Trafficking** | | 236 | 261 | 1.34 | 1.06 | * |
| 1373353_at | Brpf1 | Bromodomain and PHD finger containing, 1 | | **Transcriptional regulation** | | 458 | 474 | 1.27 | 1.01 | * |
| 1395821_at | Cbfb | Core-binding factor, beta subunit | | **Transcriptional regulation** | | 811 | 829 | 0.78 | 1.07 | * |
| 1389564_at | Ccnl2 | Cyclin L2 | | **Transcriptional regulation** | | 677 | 2048 | 1.43 | 1.02 | * |
| 1376631_at | Crtc3 | CREB regulated transcription coactivator 3 | | **Transcriptional regulation** | | 630 | 532 | 0.79 | 1.08 | * |
| 1383287_at | Fubp1 | Far upstream element (FUSE) binding protein 1 | | **Transcriptional regulation** | | 691 | 907 | 0.74 | 1.06 | * |
| 1386129_at | Ing2 | Inhibitor of growth family, member 2 | | **Transcriptional regulation** | | 247 | 185 | 1.29 | 1.00 | * |
| 1371560_at | Irf3 | Interferon regulatory factor 3 | | **Transcriptional regulation** | | 293 | 439 | 1.35 | 1.03 | * |
| 1370751_at | LOC257642 | rRNA promoter binding protein | | **Transcriptional regulation** | | 2886 | 3856 | 0.69 | 1.24 | * |
| 1375253_at | Nfe2l1 | Nuclear factor, erythroid derived 2,-like 1 | | **Transcriptional regulation** | | 388 | 405 | 0.80 | 1.06 | * |
| 1386935_at | Nr4a1 | Nuclear receptor subfamily 4, group A, member 1 | | **Transcriptional regulation** | | 574 | 635 | 1.39 | 1.08 | * |
| 1390647_at | Phtf2 | Putative homeodomain transcription factor 2 | | **Transcriptional regulation** | | 663 | 756 | 1.31 | 0.99 | * |
| 1395294_at | Runx1t1 | Runt-related transcription factor 1; translocated to, 1 | | **Transcriptional regulation** | | 444 | 328 | 0.76 | 1.01 | * |
| 1394093_at | Smarca5 | SWI/SNF related, matrix associated, actin dependent regulator of chromatin, subfamily a, member 5 | | **Transcriptional regulation** | | 266 | 507 | 0.80 | 1.09 | * |
| 1388426_at | Srebf1 | Sterol regulatory element binding transcription factor 1 | | **Transcriptional regulation** | | 910 | 1071 | 0.74 | 0.99 | * |
| 1392957_at | Ssbp2 | Single-stranded DNA-binding protein 2 | | **Transcriptional regulation** | | 637 | 418 | 1.35 | 1.02 | * |
| 1375418_at | Trrap | Transformation/transcription domain-associated protein | | **Transcriptional regulation** | | 562 | 615 | 0.73 | 1.15 | * |
| 1372593_at | Zfp110 | Zinc finger protein 110 | | **Transcriptional regulation** | | 235 | 360 | 1.27 | 0.95 | * |
| 1389674_at | Zfp282 | Zinc finger protein 282 | | **Transcriptional regulation** | | 330 | 352 | 0.80 | 1.00 | * |
| 1370459_at | Aard | Alanine and arginine rich domain containing protein | | **Unknown function** | | 261 | 216 | 1.28 | 0.92 | * |
| 1392911_at | Abp10 | Annexin V-binding protein ABP-10 | | **Unknown function** | | 316 | 605 | 0.72 | 1.06 | * |
| 1371703_at | Ahnak | AHNAK nucleoprotein (desmoyokin) | | **Unknown function** | | 2235 | 7066 | 0.71 | 0.93 | * |
| 1384253_at | Anxa10 | Annexin A10 | | **Unknown function** | | 468 | 508 | 1.27 | 1.01 | * |
| 1393051_at | Armcx1 | Armadillo repeat containing, X-linked 1 | | **Unknown function** | | 668 | 959 | 0.76 | 0.96 | * |
| 1388536_at | Bat2 | HLA-B associated transcript 2 | | **Unknown function** | | 852 | 1221 | 0.73 | 0.96 | * |
| 1369032_at | Blcap | Bladder cancer associated protein homolog (human) | | **Unknown function** | | 475 | 478 | 0.79 | 1.06 | * |
| 1375282_at | Ccm2 | Cerebral cavernous malformation 2 | | **Unknown function** | | 767 | 581 | 0.75 | 1.09 | * |
| 1388682_at | Cnih | Cornichon homolog (Drosophila) | | **Unknown function** | | 4770 | 4561 | 1.25 | 1.02 | * |
| 1379087_at | Fam109b | Family with sequence similarity 109, member B | | **Unknown function** | | 441 | 222 | 0.80 | 1.05 | * |
| 1393696_at | Fibin | Fin bud initiation factor | | **Unknown function** | | 513 | 407 | 0.79 | 1.03 | * |
| 1384378_at | Hcr | HCR (a-helix coiled-coil rod homolog) | | **Unknown function** | | 402 | 405 | 0.78 | 1.02 | * |
| 1390365_at | Leng1 | Leukocyte receptor cluster (LRC) member 1 | | **Unknown function** | | 207 | 161 | 1.26 | 0.93 | * |
| 1374287_at | Pald | Paladin | | **Unknown function** | | 509 | 516 | 0.77 | 1.03 | * |
| 1377384_at | Plekhh3 | Pleckstrin homology domain containing, family H member 3 | | **Unknown function** | | 238 | 237 | 1.41 | 1.15 | * |
| 1369895_s_at | Podxl | Podocalyxin-like | | **Unknown function** | | 561 | 613 | 0.77 | 0.98 | * |
| 1387046_at | Slb | Selective LIM binding factor, rat homolog | | **Unknown function** | | 259 | 371 | 0.68 | 0.90 | * |
| 1373998_at | Specc1 | Sperm antigen with calponin homology and coiled-coil domains 1 | | **Unknown function** | | 513 | 740 | 0.74 | 0.94 | * |
| 1390428_at | Thap6 | THAP domain containing 6 | | **Unknown function** | | 201 | 189 | 1.33 | 1.02 | * |
| 1382596_a_at | Tmem214 | Transmembrane protein 214 | | **Unknown function** | | 913 | 888 | 0.77 | 1.08 | * |
| 1392683_at | Tmem71 | Transmembrane protein 71 | | **Unknown function** | | 232 | 356 | 1.44 | 0.84 | * |
| 1378700_at | Tnfaip8l3 | Tumor necrosis factor, alpha-induced protein 8-like 3 | | **Unknown function** | | 449 | 294 | 0.72 | 0.93 | * |
| 1395974_at | Wdr81 | WD repeat domain 81 | | **Unknown function** | | 367 | 394 | 0.71 | 0.98 | * |
| 1391455_at | Zc3h18 | Zinc finger CCCH-type containing 18 | | **Unknown function** | | 300 | 326 | 1.32 | 1.09 | * |
| 1385719_at | Zfp597 | Zinc finger protein 597 | | **Unknown function** | | 179 | 209 | 1.42 | 1.16 | * |
| 1388919_at | Znf541 | Zinc finger protein 541 | | **Unknown function** | | 416 | 266 | 0.78 | 0.99 | * |
| --- | --- | --- | | --- | | --- | --- | --- | --- |  |
| ***Regulated in total RNA (not polysomal RNA)*** | | | | | | --- | --- | --- | --- |  |
| 1380583_s_at | Csf1 | Colony stimulating factor 1 (macrophage) | | **Agonists/receptors** | | 627 | 825 | 0.96 | 1.41 | # |
| 1383721_at | Fzd8 | Frizzled homolog 8 (Drosophila) | | **Agonists/receptors** | | 354 | 788 | 1.01 | 0.79 | # |
| 1390453_at | Ldlrad3 | Low density lipoprotein receptor class A domain containing 3 | | **Agonists/receptors** | | 515 | 383 | 1.00 | 1.33 | # |
| 1387269_s_at | Plaur | Plasminogen activator, urokinase receptor | | **Agonists/receptors** | | 482 | 372 | 0.98 | 1.36 | # |
| 1375207_at | Scarf2 | Scavenger receptor class F, member 2 | | **Agonists/receptors** | | 321 | 353 | 0.98 | 1.27 | # |
| 1396614_at | Sfrp2 | Secreted frizzled-related protein 2 | | **Agonists/receptors** | | 1114 | 983 | 1.01 | 1.28 | # |
| 1370526_at | Itgae | Integrin, alpha E, epithelial-associated | | **Cell-cell/matrix adhesion** | | 220 | 218 | 1.01 | 1.32 | # |
| 1382027_at | Itgb3 | Integrin beta 3 | | **Cell-cell/matrix adhesion** | | 658 | 458 | 1.05 | 1.27 | # |
| 1381798_at | Lmo7 | LIM domain 7 | | **Cell-cell/matrix adhesion** | | 206 | 492 | 0.88 | 0.55 | # |
| 1396106_at | Dtna | Dystrobrevin alpha | | **Cytoskeleton/myofibrillar** | | 279 | 320 | 1.02 | 1.36 | # |
| 1381678_at, 1385359_at, 1394315_at | Fscn1 | Fascin homolog 1, actin bundling protein (Strongylocentrotus purpuratus) | | **Cytoskeleton/myofibrillar** | | 427, 525, 503 | 393, 387, 546 | 0.90 | 1.45 | # |
| 1373751_at | Kif21b | Kinesin family member 21B | | **Cytoskeleton/myofibrillar** | | 336 | 361 | 1.08 | 1.33 | # |
| 1371125_at | Kif2a | Kinesin family member 2A | | **Cytoskeleton/myofibrillar** | | 481 | 748 | 1.07 | 1.32 | # |
| 1395794_at | Tpm1 | Tropomyosin 1, alpha | | **Cytoskeleton/myofibrillar** | | 436 | 884 | 0.97 | 1.29 | # |
| 1380760_at | LOC72486 | Hypothetical protein LOC72486 | | **Hypothetical protein** | | 261 | 330 | 0.91 | 1.27 | # |
| 1379987_at, 1391817_at | RGD1304595 | Similar to RIKEN cDNA 6330416G13 gene | | **Hypothetical protein** | | 392, 353 | 352, 381 | 0.99 | 1.27 | # |
| 1397681_at | RGD1305793 | Similar to hypothetical protein FLJ20154 | | **Hypothetical protein** | | 441 | 649 | 0.74 | 0.57 | # |
| 1377252_at | RGD1306862 | Similar to RIKEN cDNA 1200011M11 | | **Hypothetical protein** | | 400 | 425 | 0.92 | 1.34 | # |
| 1390232_at | RGD1311946 | Similar to RIKEN cDNA 1810055G02 | | **Hypothetical protein** | | 1197 | 819 | 1.04 | 1.29 | # |
| 1371189_x_at | RGD1563757 | Similar to 40S ribosomal protein SA (p40) | | **Hypothetical protein** | | 2677 | 4134 | 1.01 | 0.80 | # |
| 1384117_at | RGD1564887 | Similar to 9130011E15Rik protein | | **Hypothetical protein** | | 384 | 464 | 0.95 | 1.26 | # |
| 1373608_at | Unknown | Unknown | | **No established gene** | | 8 | 331 | 1.01 | 0.75 | # |
| 1374552_at | Unknown | Unknown | | **No established gene** | | 416 | 381 | 1.18 | 0.77 | # |
| 1375191_at | Unknown | Unknown | | **No established gene** | | 215 | 329 | 1.08 | 0.80 | # |
| 1376911_at | Unknown | Unknown | | **No established gene** | | 305 | 305 | 0.96 | 1.29 | # |
| 1377629_at | Unknown | Unknown | | **No established gene** | | 307 | 307 | 1.20 | 1.47 | # |
| 1378261_at | Unknown | Unknown | | **No established gene** | | 190 | 254 | 0.94 | 1.30 | # |
| 1379825_at | Unknown | Unknown | | **No established gene** | | 490 | 346 | 1.12 | 0.77 | # |
| 1380155_at | Unknown | Unknown | | **No established gene** | | 262 | 755 | 0.99 | 1.29 | # |
| 1383688_at | Unknown | Unknown | | **No established gene** | | 676 | 619 | 1.11 | 0.76 | # |
| 1385066_a_at | Unknown | Unknown | | **No established gene** | | 298 | 360 | 1.02 | 1.34 | # |
| 1385608_at | Unknown | Unknown | | **No established gene** | | 286 | 262 | 0.88 | 1.29 | # |
| 1389284_at | Unknown | Unknown | | **No established gene** | | 243 | 475 | 0.96 | 1.30 | # |
| 1390093_at | Unknown | Unknown | | **No established gene** | | 132 | 262 | 1.13 | 1.46 | # |
| 1390109_at | Unknown | Unknown | | **No established gene** | | 1038 | 1417 | 0.94 | 0.73 | # |
| 1396954_at | Unknown | Unknown | | **No established gene** | | 194 | 297 | 0.83 | 0.56 | # |
| 1391423_at | AS:Hsf2 | AS:heat shock factor 2 | | **Non-protein coding** | | 234 | 513 | 1.01 | 0.78 | # |
| 1397449_at | Intron:Enah | Intron:Enabled homolog | | **Non-protein coding** | | 53 | 256 | 1.15 | 1.41 | # |
| 1393540_at | Intron:Myh6 | Intron:myosin heavy chain, polypeptide 6 | | **Non-protein coding** | | 290 | 353 | 0.91 | 1.36 | # |
| 1391710_at | Intron:Nme2 | Intron:expressed in non-metastatic cells 2 | | **Non-protein coding** | | 104 | 345 | 1.21 | 1.59 | # |
| 1395443_at | Intron:Tmem49 | Intron:Transmembrane protein 49 | | **Non-protein coding** | | 52 | 318 | 0.92 | 0.72 | # |
| 1389616_at | Intron:Whsc1 | Intron:Wolf-Hirschhorn syndrome candidate 1 | | **Non-protein coding** | | 223 | 399 | 1.17 | 0.75 | # |
| 1396172_at | Cdc16 | CDC16 cell division cycle 16 homolog (S. cerevisiae) | | **Protein processing** | | 190 | 228 | 0.93 | 1.38 | # |
| 1369813_at | Dnajc5 | DnaJ (Hsp40) homolog, subfamily C, member 5 | | **Protein processing** | | 271 | 296 | 1.07 | 1.37 | # |
| 1389117_at | Osgep | O-sialoglycoprotein endopeptidase | | **Protein processing** | | 677 | 595 | 1.09 | 0.79 | # |
| 1392881_at | St3gal4 | ST3 beta-galactoside alpha-2,3-sialyltransferase 4 | | **Protein processing** | | 385 | 459 | 1.02 | 1.25 | # |
| 1369617_at | Ube2n | Ubiquitin-conjugating enzyme E2N | | **Protein processing** | | 418 | 401 | 1.03 | 1.39 | # |
| 1396444_at | Mrpl12 | Mitochondrial ribosomal protein L12 | | **Protein synthesis** | | 393 | 262 | 0.82 | 1.26 | # |
| 1393468_at | Mrps2 | Mitochondrial ribosomal protein S2 | | **Protein synthesis** | | 213 | 239 | 0.91 | 1.41 | # |
| 1376065_at | Rrs1 | RRS1 ribosome biogenesis regulator homolog | | **Protein synthesis** | | 574 | 353 | 1.16 | 1.48 | # |
| 1369520_a_at | Bcat1 | Branched chain aminotransferase 1, cytosolic | | **Regulation of metabolism** | | 394 | 483 | 1.00 | 1.29 | # |
| 1372613_at | Bdh2 | 3-hydroxybutyrate dehydrogenase, type 2 | | **Regulation of metabolism** | | 719 | 523 | 1.09 | 0.79 | # |
| 1373790_at | Car14 | Carbonic anhydrase 14 | | **Regulation of metabolism** | | 449 | 758 | 0.93 | 0.75 | # |
| 1389096_at | Mical2 | Microtubule associated monoxygenase, calponin and LIM domain containing 2 | | **Regulation of metabolism** | | 1037 | 869 | 1.22 | 1.48 | # |
| 1372808_at | Mthfd2 | Methylenetetrahydrofolate dehydrogenase (NADP+ dependent) 2, methenyltetrahydrofolate cyclohydrolase | | **Regulation of metabolism** | | 1210 | 664 | 1.14 | 1.43 | # |
| 1387926_at, 1389111_at | Sc5d | Sterol-C5-desaturase (fungal ERG3, delta-5-desaturase) | | **Regulation of metabolism** | | 450, 624 | 660, 899 | 1.21 | 1.34 | # |
| 1374524_at | Scly | Selenocysteine lyase | | **Regulation of metabolism** | | 665 | 389 | 0.96 | 1.26 | # |
| 1389114_at | Thnsl2 | Threonine synthase-like 2 (bacterial) | | **Regulation of metabolism** | | 400 | 405 | 0.97 | 0.75 | # |
| 1397405_at | Ddx17 | DEAD (Asp-Glu-Ala-Asp) box polypeptide 17 | | **RNA regulation** | | 125 | 403 | 0.74 | 1.43 | # |
| 1370979_at | Ddx20 | DEAD (Asp-Glu-Ala-Asp) box polypeptide 20 | | **RNA regulation** | | 238 | 315 | 1.00 | 1.35 | # |
| 1384377_at | Ddx28 | DEAD (Asp-Glu-Ala-Asp) box polypeptide 28 | | **RNA regulation** | | 247 | 250 | 0.85 | 1.38 | # |
| 1391754_at | Oas1i | 2 ' -5 ' oligoadenylate synthetase 1I | | **RNA regulation** | | 343 | 309 | 1.02 | 0.69 | # |
| 1397493_at | Papola | Poly (A) polymerase alpha | | **RNA regulation** | | 256 | 258 | 0.84 | 1.35 | # |
| 1371915_at | Pcbp4 | Poly(rC) binding protein 4 | | **RNA regulation** | | 1496 | 993 | 1.07 | 1.32 | # |
| 1383336_at | Pnn | Pinin | | **RNA regulation** | | 209 | 474 | 0.94 | 1.34 | # |
| 1395129_at | Rbm15 | RNA binding motif protein 15 | | **RNA regulation** | | 431 | 270 | 1.15 | 1.62 | # |
| 1371839_at | Sfrs2 | Splicing factor, arginine/serine-rich 2 (SC-35) | | **RNA regulation** | | 1124 | 1086 | 1.06 | 1.30 | # |
| 1396170_at | Wbp4 | WW domain binding protein 4 | | **RNA regulation** | | 303 | 217 | 1.10 | 1.33 | # |
| 1372173_at | Wdr57 | WD repeat domain 57 | | **RNA regulation** | | 667 | 443 | 1.00 | 1.37 | # |
| 1394975_at | Zfml | Zinc finger, matrin-like | | **RNA regulation** | | 179 | 294 | 1.01 | 1.44 | # |
| 1391581_at, 1392864_at | Arhgap5 | Rho GTPase activating protein 5 | | **Signalling/Trafficking** | | 509, 250 | 465, 230 | 0.92 | 1.30 | # |
| 1368066_at | Bak1 | BCL2-antagonist/killer 1 | | **Signalling/Trafficking** | | 885 | 407 | 1.07 | 1.39 | # |
| 1369122_at | Bax | Bcl2-associated X protein | | **Signalling/Trafficking** | | 2351 | 990 | 1.03 | 1.27 | # |
| 1397571_at | Casc3 | Cancer susceptibility candidate 3 | | **Signalling/Trafficking** | | 410 | 584 | 1.06 | 1.32 | # |
| 1387391_at | Cdkn1a | Cyclin-dependent kinase inhibitor 1A | | **Signalling/Trafficking** | | 868 | 542 | 0.92 | 1.35 | # |
| 1373812_at | Cdkn1b | Cyclin-dependent kinase inhibitor 1B | | **Signalling/Trafficking** | | 2407 | 2271 | 0.89 | 0.73 | # |
| 1393441_at | Cdkn2d | Cyclin-dependent kinase inhibitor 2D | | **Signalling/Trafficking** | | 950 | 459 | 1.03 | 1.28 | # |
| 1399022_at | Clk1 | CDC-like kinase 1 | | **Signalling/Trafficking** | | 447 | 1900 | 1.04 | 0.78 | # |
| 1369912_at | Crk | v-crk sarcoma virus CT10 oncogene homolog | | **Signalling/Trafficking** | | 317 | 411 | 0.98 | 1.31 | # |
| 1386460_x_at | Dnm1l | Dynamin 1-like | | **Signalling/Trafficking** | | 123 | 253 | 0.81 | 0.61 | # |
| 1368146_at | Dusp1 | Dual specificity phosphatase 1 | | **Signalling/Trafficking** | | 2345 | 2320 | 0.91 | 0.75 | # |
| 1377064_at | Dusp6 | Dual specificity phosphatase 6 | | **Signalling/Trafficking** | | 2693 | 1350 | 1.00 | 1.26 | # |
| 1369453_at | Epn1 | Epsin 1 | | **Signalling/Trafficking** | | 685 | 403 | 0.90 | 1.45 | # |
| 1382317_at | Eps8 | Epidermal growth factor receptor pathway substrate 8 | | **Signalling/Trafficking** | | 290 | 400 | 1.10 | 0.79 | # |
| 1387237_at | Exoc7 | Exocyst complex component 7 | | **Signalling/Trafficking** | | 203 | 275 | 0.94 | 0.75 | # |
| 1390050_at | Golm1 | Golgi membrane protein 1 | | **Signalling/Trafficking** | | 214 | 432 | 1.02 | 0.75 | # |
| 1388796_at | Gosr1 | Golgi SNAP receptor complex member 1 | | **Signalling/Trafficking** | | 462 | 365 | 1.01 | 1.35 | # |
| 1397211_at | Grb10 | Growth factor receptor bound protein 10 | | **Signalling/Trafficking** | | 139 | 569 | 0.89 | 1.40 | # |
| 1371091_at | Irs2 | Insulin receptor substrate 2 | | **Signalling/Trafficking** | | 601 | 959 | 0.95 | 0.67 | # |
| 1394077_at | Rnd3 | Rho family GTPase 3 | | **Signalling/Trafficking** | | 714 | 741 | 1.00 | 1.38 | # |
| 1390689_at | Rps6kc1 | Ribosomal protein S6 kinase, polypeptide 1 | | **Signalling/Trafficking** | | 240 | 228 | 1.07 | 1.36 | # |
| 1367862_at | Rrad | Ras-related associated with diabetes | | **Signalling/Trafficking** | | 1672 | 2283 | 0.93 | 0.63 | # |
| 1377174_at | Sbf1 | SET binding factor 1 | | **Signalling/Trafficking** | | 286 | 337 | 0.88 | 1.30 | # |
| 1381203_at | Sh3glb1 | SH3-domain GRB2-like endophilin B1 | | **Signalling/Trafficking** | | 577 | 531 | 1.06 | 0.78 | # |
| 1382643_at | Snx16 | Sorting nexin 16 | | **Signalling/Trafficking** | | 407 | 318 | 0.95 | 1.31 | # |
| 1373482_at | Traf3 | Tnf receptor-associated factor 3 | | **Signalling/Trafficking** | | 637 | 629 | 1.01 | 1.26 | # |
| 1384163_at | Trp53inp1 | Transformation related protein 53 inducible nuclear protein 1 | | **Signalling/Trafficking** | | 1581 | 1935 | 0.89 | 0.71 | # |
| 1369974_at | Vamp2 | Vesicle-associated membrane protein 2 | | **Signalling/Trafficking** | | 905 | 509 | 1.03 | 1.32 | # |
| 1385449_s_at | Vps37b | Vacuolar protein sorting 37 homolog B (S. cerevisiae) | | **Signalling/Trafficking** | | 253 | 220 | 1.04 | 1.33 | # |
| 1373885_at | Cbx5 | Chromobox homolog 5 (Drosophila HP1a) | | **Transcriptional regulation** | | 399 | 354 | 1.14 | 0.75 | # |
| 1384127_at | Cebpa | CCAAT/enhancer binding protein (C/EBP), alpha | | **Transcriptional regulation** | | 331 | 207 | 1.11 | 1.46 | # |
| 1389712_at | Ebf3 | Early B-cell factor 3 | | **Transcriptional regulation** | | 467 | 370 | 1.02 | 1.33 | # |
| 1387306_a_at | Egr2 | Early growth response 2 | | **Transcriptional regulation** | | 749 | 464 | 1.16 | 1.52 | # |
| 1389404_at | Fkhl18 | Forkhead-like 18 (Drosophila) | | **Transcriptional regulation** | | 619 | 385 | 1.16 | 1.52 | # |
| 1383199_at | Fli1 | Friend leukemia integration 1 | | **Transcriptional regulation** | | 374 | 385 | 0.97 | 0.76 | # |
| 1374404_at, 1389528_s_at | Jun | Jun oncogene | | **Transcriptional regulation** | | 406, 1313 | 449, 1432 | 1.04 | 1.33 | # |
| 1374231_at | Klf16 | Kruppel-like factor 16 | | **Transcriptional regulation** | | 1364 | 796 | 1.11 | 1.45 | # |
| 1376569_at | Klf2 | Kruppel-like factor 2 (lung) | | **Transcriptional regulation** | | 1009 | 673 | 1.05 | 1.26 | # |
| 1390728_at | Limd1 | LIM domains containing 1 | | **Transcriptional regulation** | | 538 | 535 | 1.14 | 0.70 | # |
| 1377802_at | Myst4 | MYST histone acetyltransferase monocytic leukemia 4 | | **Transcriptional regulation** | | 417 | 374 | 1.04 | 1.26 | # |
| 1372102_at | Ncor1 | Nuclear receptor co-repressor 1 | | **Transcriptional regulation** | | 441 | 390 | 0.93 | 1.31 | # |
| 1369678_a_at | Nfia | Nuclear factor I/A | | **Transcriptional regulation** | | 700 | 482 | 0.87 | 1.27 | # |
| 1379312_at | Pprc1 | Peroxisome proliferative activated receptor, gamma, coactivator-related 1 | | **Transcriptional regulation** | | 428 | 464 | 1.01 | 1.38 | # |
| 1394010_at | Rsf1 | Remodeling and spacing factor 1 | | **Transcriptional regulation** | | 288 | 382 | 0.94 | 1.30 | # |
| 1375121_at | Smad6 | MAD homolog 6 | | **Transcriptional regulation** | | 779 | 798 | 0.96 | 1.42 | # |
| 1368897_at | Smad7 | MAD homolog 7 | | **Transcriptional regulation** | | 263 | 291 | 1.00 | 1.34 | # |
| 1383210_at | Sox11 | SRY-box containing gene 11 | | **Transcriptional regulation** | | 340 | 264 | 1.07 | 1.35 | # |
| 1390940_at | Zfhx1b | Zinc finger homeobox 1b | | **Transcriptional regulation** | | 167 | 234 | 0.75 | 1.48 | # |
| 1393150_at | Zfp161 | Zinc finger protein 161 | | **Transcriptional regulation** | | 986 | 730 | 1.03 | 1.32 | # |
| 1384901_at | Zfp451 | Zinc finger protein 451 | | **Transcriptional regulation** | | 294 | 382 | 1.02 | 1.33 | # |
| 1369310_at | Basp1 | Brain abundant, membrane attached signal protein 1 | | **Unknown function** | | 2130 | 1049 | 0.99 | 1.38 | # |
| 1380232_at | Cbwd1 | COBW domain containing 1 | | **Unknown function** | | 316 | 310 | 0.87 | 1.28 | # |
| 1375890_at | Dact3 | Dapper, antagonist of beta-catenin, homolog 3 | | **Unknown function** | | 1381 | 777 | 1.06 | 1.31 | # |
| 1375416_at | Fam104a | Family with sequence similarity 104, member A | | **Unknown function** | | 1089 | 620 | 0.87 | 1.26 | # |
| 1372526_at | Flcn | Folliculin | | **Unknown function** | | 2922 | 2903 | 0.93 | 0.77 | # |
| 1372201_at | Ggnbp2 | Gametogenetin binding protein 2 | | **Unknown function** | | 946 | 1296 | 1.01 | 1.30 | # |
| 1378113_at | Gm22 | Gene model 22 | | **Unknown function** | | 299 | 282 | 0.94 | 1.29 | # |
| 1373773_at | Gpm6a | Glycoprotein m6a | | **Unknown function** | | 203 | 427 | 1.14 | 0.78 | # |
| 1391429_at | Hfe2 | Hemochromatosis type 2 (juvenile) homolog | | **Unknown function** | | 321 | 363 | 0.97 | 0.75 | # |
| 1378907_at | Kank2 | KN motif and ankyrin repeat domains 2 | | **Unknown function** | | 293 | 349 | 0.97 | 1.26 | # |
| 1383300_at | Klhl24 | Kelch-like 24 (Drosophila) | | **Unknown function** | | 1385 | 1404 | 1.06 | 0.79 | # |
| 1392755_at | Lrrc8c | Leucine rich repeat containing 8 family, member C | | **Unknown function** | | 422 | 470 | 0.98 | 1.29 | # |
| 1375224_at | Phlda3 | Pleckstrin homology-like domain, family A, member 3 | | **Unknown function** | | 4379 | 1732 | 1.06 | 1.29 | # |
| 1374701_at | Rnf185 | Ring finger protein 185 | | **Unknown function** | | 444 | 404 | 1.12 | 0.75 | # |
| 1395065_at | Wdr41 | WD repeat domain 41 | | **Unknown function** | | 526 | 495 | 1.03 | 0.78 | # |
| 1375695_at | Ythdf2 | YTH domain family 2 | | **Unknown function** | | 1222 | 1406 | 0.87 | 1.26 | # |
| 1380695_at | Zyg11b | Zyg-ll homolog B | | **Unknown function** | | 221 | 312 | 0.96 | 1.34 | # |
| --- | --- | --- | | --- | | --- | --- | --- | --- |  |
| **No evidence for translational regulation (FDR<0.05 in polysomal and total RNAs OR ratio of P:T or T:P >1.2-fold)** | | | | | | | --- | --- | --- |  |
| 1383848_at | Adrb1 | | Adrenergic receptor, beta 1 | **Agonists/receptors** | | 640 | 718 | 1.32 | 1.39 | * |
| 1369814_at | Ccl20 | | Chemokine (C-C motif) ligand 20 | **Agonists/receptors** | | 410 | 316 | 1.27 | 1.06 | * |
| 1370832_at | Ccl4 | | Chemokine (C-C motif) ligand 4 | **Agonists/receptors** | | 708 | 619 | 0.86 | 0.77 | # |
| 1389123_at | Ccl6 | | Chemokine (C-C motif) ligand 6 | **Agonists/receptors** | | 1473 | 972 | 0.91 | 0.79 | # |
| 1390024_at | Clec2d/g | | C-type lectin domain family 2, member d/g | **Agonists/receptors** | | 1845 | 1901 | 0.63 | 0.57 | *# |
| 1393038_at | Fcgr1 | | Fc receptor, IgG, high affinity I | **Agonists/receptors** | | 388 | 382 | 0.78 | 0.78 | * |
| 1367850_at | Fcgr3 | | Fc receptor, IgG, low affinity III | **Agonists/receptors** | | 5595 | 3814 | 0.82 | 0.75 | # |
| 1370106_at | Fgf18 | | Fibroblast growth factor 18 | **Agonists/receptors** | | 246 | 137 | 1.52 | 1.46 | * |
| 1372750_at | Fst | | Follistatin | **Agonists/receptors** | | 496 | 496 | 1.19 | 1.40 | # |
| 1382566_at | Il7r | | Interleukin 7 receptor | **Agonists/receptors** | | 295 | 492 | 0.79 | 0.82 | * |
| 1376089_at | Ldlr | | Low density lipoprotein receptor | **Agonists/receptors** | | 1453 | 3814 | 1.36 | 1.23 | * |
| 1393728_at | Lif | | Leukemia inhibitory factor | **Agonists/receptors** | | 416 | 291 | 1.80 | 2.11 | * |
| 1383135_at | Ntrk2 | | Neurotrophic tyrosine kinase, receptor, type 2 | **Agonists/receptors** | | 227 | 266 | 1.06 | 1.26 | # |
| 1377702_at | P2ry5 | | Purinergic receptor P2Y, G-protein coupled, 5 | **Agonists/receptors** | | 664 | 825 | 0.87 | 0.79 | # |
| 1373579_at | Rara | | Retinoic acid receptor, alpha | **Agonists/receptors** | | 1000 | 635 | 1.31 | 1.13 | * |
| 1371785_at | Tnfrsf12a | | Tumor necrosis factor receptor superfamily, member 12a | **Agonists/receptors** | | 6412 | 3932 | 1.25 | 1.32 | # |
| 1389660_at | Amigo3 | | Amphoterin induced gene and ORF 3 | **Cell-cell/matrix adhesion** | | 260 | 220 | 1.43 | 1.41 | *# |
| 1373182_at | Cldn12 | | Claudin 12 | **Cell-cell/matrix adhesion** | | 681 | 603 | 1.34 | 1.15 | * |
| 1369955_at, 1376099_at | Col5a1 | | Collagen, type V, alpha 1 | **Cell-cell/matrix adhesion** | | 1230, 1462 | 4230, 3166 | 0.76 | 0.85 | * |
| 1387451_at | Dcbld2 | | Discoidin, CUB and LCCL domain containing 2 | **Cell-cell/matrix adhesion** | | 383 | 808 | 1.34 | 1.13 | * |
| 1388230_at | Jub | | Ajuba homolog (Xenopus laevis) | **Cell-cell/matrix adhesion** | | 298 | 378 | 1.26 | 1.31 | # |
| 1371518_at | Nid1 | | Nidogen 1 | **Cell-cell/matrix adhesion** | | 3985 | 7377 | 0.77 | 0.87 | * |
| 1383224_at | Pard6b | | Par-6 (partitioning defective 6) homolog beta (C. elegans) | **Cell-cell/matrix adhesion** | | 265 | 276 | 1.27 | 1.34 | *# |
| 1397363_at | Pvrl3 | | Poliovirus receptor-related 3 | **Cell-cell/matrix adhesion** | | 233 | 358 | 1.28 | 1.08 | * |
| 1386865_at | Sparcl1 | | SPARC-like 1 (hevin) | **Cell-cell/matrix adhesion** | | 1001 | 1885 | 0.79 | 0.80 | * |
| 1389573_at | Chac1 | | ChaC, cation transport regulator-like 1 | **Channels/transporters** | | 1661 | 764 | 1.53 | 2.32 | *# |
| 1368343_at | Kcnh2 | | Potassium voltage-gated channel, subfamily H (eag-related), member 2 | **Channels/transporters** | | 239 | 319 | 1.28 | 1.07 | * |
| 1397764_at | Kctd5 | | Potassium channel tetramerisation domain containing 5 | **Channels/transporters** | | 1975 | 1016 | 1.29 | 1.26 | *# |
| 1382775_at | Ryr2 | | Ryanodine receptor 2, cardiac | **Channels/transporters** | | 405 | 961 | 0.69 | 0.74 | # |
| 1387010_s_at | Scn1b | | Sodium channel, voltage-gated, type I, beta | **Channels/transporters** | | 1574 | 1249 | 1.10 | 1.30 | # |
| 1376267_at | Slc16a6 | | Solute carrier family 16 (monocarboxylic acid transporters), member 6 | **Channels/transporters** | | 233 | 258 | 1.30 | 1.22 | * |
| 1371754_at | Slc25a25 | | Solute carrier family 25 (mitochondrial carrier, phosphate carrier), member 25 | **Channels/transporters** | | 493 | 295 | 1.50 | 1.90 | *# |
| 1374845_at | Slc25a37 | | Solute carrier family 25, member 37 | **Channels/transporters** | | 357 | 303 | 1.15 | 1.25 | # |
| 1383253_at, 1383632_at | Slc30a1 | | Solute carrier family 30 (zinc transporter), member 1 | **Channels/transporters** | | 636, 997 | 911, 1201 | 1.46 | 1.58 | *# |
| 1370286_at | Slc38a2 | | Solute carrier family 38, member 2 | **Channels/transporters** | | 2692 | 5840 | 0.91 | 0.78 | # |
| 1390800_a_at | Cav2 | | Caveolin 2 | **Cytoskeleton/myofibrillar** | | 515 | 369 | 0.66 | 0.64 | * |
| 1372569_at | Fhl3 | | Four and a half LIM domains 3 | **Cytoskeleton/myofibrillar** | | 2888 | 1808 | 1.14 | 1.29 | # |
| 1395663_at | Mall | | Mal, T-cell differentiation protein-like | **Cytoskeleton/myofibrillar** | | 424 | 295 | 0.75 | 0.87 | * |
| 1372091_at | Mid1ip1 | | MID1 interacting protein 1 | **Cytoskeleton/myofibrillar** | | 2453 | 1392 | 1.24 | 1.34 | # |
| 1386993_at | Myh7 | | Myosin, heavy chain 7, cardiac muscle, beta | **Cytoskeleton/myofibrillar** | | 7200 | 21096 | 0.78 | 0.93 | * |
| 1386957_at | Pom121 | | Nuclear pore membrane protein 121 | **Cytoskeleton/myofibrillar** | | 653 | 765 | 1.10 | 1.26 | # |
| 1388589_at | Dot1l | | DOT1-like, histone H3 methyltransferase | **DNA structure/repair** | | 534 | 602 | 1.28 | 1.19 | * |
| 1393210_at | Ecm2 | | Extracellular matrix protein 2 | **Extracellular matrix** | | 154 | 371 | 0.84 | 0.72 | # |
| 1372518_at | Fbln1 | | Fibulin 1 | **Extracellular matrix** | | 1335 | 1666 | 0.78 | 0.94 | * |
| 1398337_at | LOC686295 | | Similar to CG17293-PA | **Hypothetical protein** | | 1842 | 1693 | 1.26 | 1.11 | * |
| 1376623_at | MGC105601 | | Similar to hypothetical protein FLJ20898 | **Hypothetical protein** | | 1201 | 847 | 0.85 | 0.80 | # |
| 1376043_at | MGC94192 | | Similar to PHD zinc finger containing protein JUNE1 | **Hypothetical protein** | | 1708 | 1377 | 1.28 | 1.20 | * |
| 1373961_at | MGC95208 | | Similar to 4930453N24Rik protein | **Hypothetical protein** | | 728 | 652 | 1.33 | 1.11 | * |
| 1385208_at | RGD1305014 | | Similar to RIKEN cDNA 2310057M21 | **Hypothetical protein** | | 310 | 349 | 1.27 | 1.30 | * |
| 1384185_at | RGD1307704 | | Similar to RIKEN cDNA 2410016O06 | **Hypothetical protein** | | 610 | 534 | 1.19 | 1.26 | # |
| 1393009_at | RGD1309326 | | Similar to RIKEN cDNA 2410002F23 | **Hypothetical protein** | | 338 | 469 | 1.31 | 1.18 | * |
| 1390315_a_at | RGD1309414 | | Similar to KIAA0913 protein | **Hypothetical protein** | | 356 | 546 | 1.09 | 1.29 | # |
| 1383337_at | RGD1311055 | | Similar to HSPC037 protein | **Hypothetical protein** | | 150 | 210 | 1.31 | 1.56 | # |
| 1388525_at | RGD1311203 | | Similar to HGFL protein | **Hypothetical protein** | | 914 | 1160 | 0.76 | 0.67 | *# |
| 1372316_at | RGD1311939 | | Similar to AI115348 protein | **Hypothetical protein** | | 1066 | 756 | 1.39 | 1.68 | *# |
| 1374945_at | RGD1359191 | | GCD14/PCMT domain containing protein RGD1359191 | **Hypothetical protein** | | 908 | 437 | 1.42 | 1.39 | *# |
| 1398364_at | RGD1359529 | | Similar to chromosome 1 open reading frame 63 | **Hypothetical protein** | | 429 | 2454 | 0.68 | 0.70 | *# |
| 1383874_at | RGD1560812 | | Hypothetical protein RGD1560812 | **Hypothetical protein** | | 461 | 397 | 1.35 | 1.30 | * |
| 1392987_at | RGD1561605 | | Similar to hypothetical protein | **Hypothetical protein** | | 1149 | 834 | 1.26 | 1.33 | *# |
| 1390051_at | RGD1562533 | | Similar to mKIAA0774 protein | **Hypothetical protein** | | 1372 | 894 | 1.13 | 1.28 | # |
| 1372871_at | RGD735175 | | Hypothetical protein MGC:72616 | **Hypothetical protein** | | 1191 | 950 | 0.78 | 0.92 | * |
| 1371552_at | Unknown | | Unknown | **No established gene** | | 589 | 780 | 1.30 | 1.20 | * |
| 1371719_at | Unknown | | Unknown | **No established gene** | | 950 | 863 | 1.29 | 1.46 | # |
| 1373359_at | Unknown | | Unknown | **No established gene** | | 229 | 316 | 0.90 | 0.77 | # |
| 1373583_at | Unknown | | Unknown | **No established gene** | | 370 | 560 | 1.29 | 1.61 | *# |
| 1374060_at | Unknown | | Unknown | **No established gene** | | 1005 | 1314 | 0.74 | 0.79 | *# |
| 1374321_at | Unknown | | Unknown | **No established gene** | | 678 | 613 | 1.30 | 1.24 | * |
| 1375672_at | Unknown | | Unknown | **No established gene** | | 1041 | 1271 | 1.29 | 1.08 | * |
| 1376318_at | Unknown | | Unknown | **No established gene** | | 447 | 430 | 1.16 | 1.26 | # |
| 1376737_at | Unknown | | Unknown | **No established gene** | | 581 | 975 | 1.22 | 1.25 | # |
| 1377925_at | Unknown | | Unknown | **No established gene** | | 221 | 353 | 1.28 | 1.16 | * |
| 1378183_at | Unknown | | Unknown | **No established gene** | | 471 | 540 | 1.62 | 1.33 | *# |
| 1378808_at | Unknown | | Unknown | **No established gene** | | 239 | 472 | 0.75 | 0.68 | # |
| 1379089_at | Unknown | | Unknown | **No established gene** | | 57 | 331 | 1.40 | 1.38 | # |
| 1383486_at | Unknown | | Unknown | **No established gene** | | 310 | 290 | 1.28 | 1.29 | # |
| 1384188_at | Unknown | | Unknown | **No established gene** | | 362 | 526 | 0.82 | 0.76 | # |
| 1384401_at | Unknown | | Unknown | **No established gene** | | 410 | 522 | 0.78 | 0.82 | * |
| 1384427_at | Unknown | | Unknown | **No established gene** | | 668 | 743 | 1.26 | 1.19 | * |
| 1385877_at | Unknown | | Unknown | **No established gene** | | 225 | 222 | 1.07 | 1.28 | # |
| 1386065_at | Unknown | | Unknown | **No established gene** | | 312 | 269 | 1.12 | 1.33 | # |
| 1386633_at | Unknown | | Unknown | **No established gene** | | 167 | 299 | 0.73 | 0.75 | # |
| 1388473_at | Unknown | | Unknown | **No established gene** | | 331 | 336 | 1.47 | 1.26 | *# |
| 1389412_at | Unknown | | Unknown | **No established gene** | | 912 | 743 | 1.25 | 1.22 | * |
| 1389460_at | Unknown | | Unknown | **No established gene** | | 396 | 463 | 0.91 | 0.77 | # |
| 1389618_at | Unknown | | Unknown | **No established gene** | | 1142 | 1395 | 0.83 | 0.75 | # |
| 1390743_at | Unknown | | Unknown | **No established gene** | | 74 | 198 | 1.21 | 1.37 | # |
| 1398587_at | Unknown | | Unknown | **No established gene** | | 514 | 327 | 0.78 | 0.87 | * |
| 1375676_at | AS:Lin7c | | AS:Lin-7 homolog C (C. elegans) | **Non-protein coding** | | 373 | 370 | 1.51 | 1.40 | *# |
| 1374429_at | AS:Pim1 | | AS:Pim-1 kinase | **Non-protein coding** | | 1566 | 1425 | 0.73 | 0.59 | *# |
| 1382291_at | AS:Sox9 | | AS:SRY-box containing gene 9 | **Non-protein coding** | | 458 | 449 | 1.48 | 1.39 | * |
| 1378945_at | Intron:Aco2 | | Intron:Aconitase 2 | **Non-protein coding** | | 4 | 199 | 1.20 | 1.40 | # |
| 1395215_at | Intron:Baz2b | | Intron:Baz2b | **Non-protein coding** | | 745 | 917 | 0.76 | 0.90 | * |
| 1395350_at | Intron:Tpm1 | | Intron:Tropomyosin 1, alpha | **Non-protein coding** | | 120 | 234 | 1.10 | 1.27 | # |
| 1377234_at | Intron:Trim27 | | Intron:Trim27 | **Non-protein coding** | | 474 | 415 | 1.34 | 1.19 | * |
| 1380087_at | Intron:Ube2e | | Intron:Ubiquitin-conjugating enzyme E2B, RAD6 homolog | **Non-protein coding** | | 79 | 432 | 0.45 | 0.77 | # |
| 1372642_at | Snhg3 | | Small nucleolar RNA host gene (non-protein coding) 3 | **Non-protein coding** | | 555 | 934 | 1.29 | 1.13 | * |
| 1369027_at | A4galt | | Alpha 1,4-galactosyltransferase | **Protein processing** | | 415 | 294 | 1.19 | 1.34 | # |
| 1394483_at | Adamts5 | | A disintegrin-like and metallopeptidase (reprolysin type) with thrombospondin type 1 motif, 5 (aggrecanase-2) | **Protein processing** | | 677 | 730 | 0.91 | 0.78 | # |
| 1377789_at | Chst2 | | Carbohydrate sulfotransferase 2 | **Protein processing** | | 227 | 173 | 1.34 | 1.46 | * |
| 1383302_at | Dnajb1 | | DnaJ (Hsp40) homolog, subfamily B, member 1 | **Protein processing** | | 1327 | 1034 | 1.17 | 1.35 | # |
| 1387116_at | Dnajb9 | | DnaJ (Hsp40) homolog, subfamily B, member 9 | **Protein processing** | | 1232 | 1365 | 0.80 | 0.88 | * |
| 1383465_at | Fbxl14 | | F-box and leucine-rich repeat protein 14 | **Protein processing** | | 486 | 372 | 1.15 | 1.27 | # |
| 1389078_at | Fbxl6 | | F-box and leucine-rich repeat protein 6 | **Protein processing** | | 745 | 560 | 0.92 | 0.80 | # |
| 1379401_a_at | Fbxo33 | | F-box only protein 33 | **Protein processing** | | 392 | 424 | 0.75 | 0.79 | # |
| 1388439_at | Fkbp10 | | FK506 binding protein 10 | **Protein processing** | | 1527 | 1848 | 0.78 | 0.90 | * |
| 1388758_at | Ogt | | O-linked N-acetylglucosamine (GlcNAc) transferase | **Protein processing** | | 310 | 1235 | 0.78 | 0.84 | * |
| 1370699_a_at | Pepd | | Peptidase D | **Protein processing** | | 967 | 1056 | 0.88 | 0.78 | # |
| 1390855_at | Prep | | Prolyl endopeptidase | **Protein processing** | | 237 | 326 | 0.78 | 0.93 | * |
| 1380569_at | Rnf41 | | Ring finger protein 41 | **Protein processing** | | 330 | 259 | 1.16 | 1.27 | # |
| 1368519_at | Serpine1 | | Serine (or cysteine) peptidase inhibitor,clade E,member 1 | **Protein processing** | | 343 | 480 | 1.24 | 1.41 | # |
| 1384220_at | Tbcc | | Tubulin-specific chaperone c | **Protein processing** | | 783 | 531 | 1.36 | 1.23 | * |
| 1385407_at | Tiparp | | TCDD-inducible poly(ADP-ribose) polymerase | **Protein processing** | | 1312 | 1629 | 1.29 | 1.17 | * |
| 1398767_at | Ubc | | Ubiquitin C | **Protein processing** | | 9457 | 13352 | 0.75 | 0.68 | *# |
| 1395019_at | Usp38 | | Ubiquitin specific peptidase 38 | **Protein processing** | | 185 | 340 | 1.20 | 1.38 | # |
| 1383538_at | Zfp650 | | Zinc finger protein 650 | **Protein processing** | | 507 | 1340 | 0.79 | 0.90 | * |
| 1385212_at | Gspt1 | | G1 to S phase transition 1 | **Protein synthesis** | | 137 | 292 | 1.23 | 1.26 | # |
| 1375534_at | Pum2 | | Pumilio homolog 2 (Drosophila) | **Protein synthesis** | | 2213 | 1530 | 0.77 | 0.91 | * |
| 1367763_at | Acat1 | | Acetyl-coenzyme A acetyltransferase 1 | **Regulation of metabolism** | | 1732 | 2469 | 0.78 | 0.90 | * |
| 1373302_at | Acer2 | | Alkaline ceramidase 2 | **Regulation of metabolism** | | 615 | 455 | 0.78 | 0.75 | *# |
| 1367942_at | Acp5 | | Acid phosphatase 5, tartrate resistant | **Regulation of metabolism** | | 886 | 671 | 0.80 | 0.69 | * |
| 1387344_at | Aldh6a1 | | Aldehyde dehydrogenase 6 family, member A1 | **Regulation of metabolism** | | 441 | 1184 | 0.78 | 0.86 | * |
| 1370317_at | Alg10b | | Asparagine-linked glycosylation 10, alpha-1,2-glucosyltransferase homolog B (yeast) | **Regulation of metabolism** | | 524 | 660 | 1.31 | 1.09 | * |
| 1372536_at | Cabc1 | | Chaperone, ABC1 activity of bc1 complex like | **Regulation of metabolism** | | 388 | 585 | 0.78 | 0.75 | *# |
| 1373866_at | Coq10b | | Coenzyme Q10 homolog B | **Regulation of metabolism** | | 1602 | 1485 | 1.35 | 1.41 | *# |
| 1386885_at | Ech1 | | Enoyl coenzyme A hydratase 1, peroxisomal | **Regulation of metabolism** | | 2903 | 2487 | 0.94 | 0.79 | # |
| 1387548_at | Has2 | | Hyaluronan synthase 2 | **Regulation of metabolism** | | 340 | 413 | 1.47 | 1.56 | * |
| 1371245_a_at | Hbb-b1 | | Hemoglobin beta chain complex | **Regulation of metabolism** | | 1582 | 3358 | 0.85 | 0.76 | # |
| 1375852_at | Hmgcr | | 3-hydroxy-3-methylglutaryl-Coenzyme A reductase | **Regulation of metabolism** | | 1110 | 2051 | 1.30 | 1.54 | *# |
| 1389430_at | Hsd17b7 | | Hydroxysteroid (17-beta) dehydrogenase 7 | **Regulation of metabolism** | | 458 | 659 | 1.15 | 1.27 | # |
| 1388872_at | Idi1 | | Isopentenyl-diphosphate delta isomerase | **Regulation of metabolism** | | 1785 | 1817 | 1.22 | 1.27 | # |
| 1372524_at | Lpin1 | | Lipin 1 | **Regulation of metabolism** | | 259 | 458 | 0.79 | 0.80 | * |
| 1371350_at, 1387737_at | Mat2a | | Methionine adenosyltransferase II, alpha | **Regulation of metabolism** | | 1928, 355 | 3333, 822 | 1.56 | 1.40 | *# |
| 1390141_at | Mthfd1l | | Methylenetetrahydrofolate dehydrogenase 1-like | **Regulation of metabolism** | | 323 | 456 | 0.72 | 0.82 | * |
| 1375684_at | Neu1 | | Sialidase 1 | **Regulation of metabolism** | | 235 | 253 | 1.33 | 1.19 | * |
| 1394706_at | Pank3 | | Pantothenate kinase 3 | **Regulation of metabolism** | | 391 | 586 | 1.09 | 1.28 | # |
| 1374693_at | Parp16 | | Poly (ADP-ribose) polymerase family, member 16 | **Regulation of metabolism** | | 563 | 413 | 0.81 | 0.79 | # |
| 1378074_at | Pdk4 | | Pyruvate dehydrogenase kinase, isoenzyme 4 | **Regulation of metabolism** | | 346 | 504 | 0.51 | 0.45 | *# |
| 1374713_at | Prmt6 | | Protein arginine N-methyltransferase 6 | **Regulation of metabolism** | | 572 | 528 | 1.35 | 1.23 | * |
| 1378080_at | Sco1 | | SCO cytochrome oxidase deficient homolog 1 (yeast) | **Regulation of metabolism** | | 942 | 628 | 1.27 | 1.23 | * |
| 1388307_at | Serinc1 | | Serine incorporator 1 | **Regulation of metabolism** | | 5094 | 8020 | 0.78 | 0.90 | * |
| 1382500_at, 1386662_at, 1391607_at | Sesn2 | | Sestrin 2 | **Regulation of metabolism** | | 648, 366, 1002 | 550, 341, 722 | 1.50 | 1.41 | *# |
| 1387017_at | Sqle | | Squalene epoxidase | **Regulation of metabolism** | | 1848 | 2981 | 1.27 | 1.28 | *# |
| 1372510_at | Srxn1 | | Sulfiredoxin 1 homolog (3' end of 3' UTR) | **Regulation of metabolism** | | 1202 | 615 | 1.59 | 1.55 | *# |
| 1384331_at | Srxn1 | | Sulfiredoxin 1 homolog (5' end of 3' UTR) | **Regulation of metabolism** | | 309 | 328 | 1.33 | 1.17 | * |
| 1388354_at | Ascc3l1 | | Activating signal cointegrator 1 complex subunit 3-like 1 | **RNA regulation** | | 566 | 1132 | 0.75 | 0.88 | * |
| 1392514_at | Bxdc1 | | Brix domain containing 1 | **RNA regulation** | | 412 | 425 | 1.32 | 1.10 | * |
| 1388599_at | Mex3a | | Mex3 homolog A (C. elegans) | **RNA regulation** | | 335 | 240 | 1.07 | 1.27 | # |
| 1382584_at | Nufip2 | | Nuclear fragile X mental retardation protein | **RNA regulation** | | 470 | 466 | 1.23 | 1.37 | # |
| 1399065_at | Rbpms2 | | RNA binding protein with multiple splicing 2 | **RNA regulation** | | 2603 | 1326 | 1.26 | 1.07 | * |
| 1389184_at | Rpp30 | | Ribonuclease P/MRP 30 subunit | **RNA regulation** | | 372 | 351 | 1.30 | 1.09 | * |
| 1371999_at, 1372075_at | Sfrs6 | | Splicing factor, arginine/serine-rich 6 | **RNA regulation** | | 383, 2454 | 653, 3007 | 1.28 | 1.15 | * |
| 1387456_at | Stau2 | | Staufen, RNA binding protein, homolog 2 (Drosophila) | **RNA regulation** | | 387 | 445 | 1.22 | 1.31 | # |
| 1372787_at | Utp3 | | UTP3, small subunit (SSU) processome component | **RNA regulation** | | 1427 | 1450 | 1.30 | 1.23 | * |
| 1369198_at | Apaf1 | | Apoptotic peptidase activating factor 1 | **Signalling/Trafficking** | | 147 | 209 | 1.12 | 1.35 | # |
| 1395461_at | Apc2 | | Adenomatosis polyposis coli 2 | **Signalling/Trafficking** | | 299 | 343 | 0.77 | 0.87 | * |
| 1372604_at | Apol9a | | Apolipoprotein L 9a | **Signalling/Trafficking** | | 333 | 496 | 1.26 | 1.10 | * |
| 1392277_at | Arhgef12 | | Rho guanine nucleotide exchange factor (GEF) 12 | **Signalling/Trafficking** | | 269 | 304 | 1.25 | 1.24 | * |
| 1375008_at | Aurkc | | Aurora kinase C | **Signalling/Trafficking** | | 170 | 310 | 1.16 | 1.28 | # |
| 1389402_at | Axud1 | | AXIN1 up-regulated 1 | **Signalling/Trafficking** | | 375 | 314 | 1.71 | 1.58 | *# |
| 1367752_at | Bcar1 | | Breast cancer anti-estrogen resistance 1 | **Signalling/Trafficking** | | 1077 | 695 | 1.27 | 1.18 | * |
| 1388742_at | Bcl2l11 | | BCL2-like 11 (apoptosis facilitator) | **Signalling/Trafficking** | | 1168 | 1016 | 0.84 | 0.74 | # |
| 1374493_at | Bmf | | Bcl2 modifying factor | **Signalling/Trafficking** | | 518 | 466 | 0.70 | 0.81 | * |
| 1371953_at | Ccng2 | | Cyclin G2 | **Signalling/Trafficking** | | 958 | 904 | 0.34 | 0.35 | *# |
| 1388105_at | Cdc123 | | Cell division cycle 123 homolog (S. cerevisiae) | **Signalling/Trafficking** | | 1494 | 1209 | 0.94 | 0.79 | # |
| 1388468_at | Cdc42se1 | | CDC42 small effector 1 | **Signalling/Trafficking** | | 2155 | 1494 | 1.44 | 1.29 | *# |
| 1384162_at | Csnrp2 | | Cysteine-serine-rich nuclear protein 2 | **Signalling/Trafficking** | | 299 | 305 | 1.69 | 1.62 | *# |
| 1377992_at | Dusp7 | | Dual specificity phosphatase 7 | **Signalling/Trafficking** | | 966 | 614 | 1.18 | 1.30 | # |
| 1381410_a_at | Fgd5 | | FYVE, RhoGEF and PH domain containing 5 | **Signalling/Trafficking** | | 412 | 404 | 0.77 | 0.72 | *# |
| 1370204_at | Frag1 | | FGF receptor activating protein 1 | **Signalling/Trafficking** | | 652 | 323 | 1.26 | 1.09 | * |
| 1384260_at | Frat2 | | Frequently rearranged in advanced T-cell lymphomas 2 | **Signalling/Trafficking** | | 1411 | 914 | 0.54 | 0.39 | *# |
| 1391623_at | Gigyf2 | | GRB10 interacting GYF protein 2 | **Signalling/Trafficking** | | 161 | 341 | 0.88 | 0.77 | # |
| 1370843_at | Gng8 | | Guanine nucleotide binding protein (G protein), gamma 8 | **Signalling/Trafficking** | | 286 | 186 | 1.30 | 1.24 | * |
| 1383543_at | Golt1b | | Golgi transport 1 homolog B | **Signalling/Trafficking** | | 460 | 374 | 1.13 | 1.26 | # |
| 1374389_at | Gucy1b3 | | Guanylate cyclase 1, soluble, beta 3 | **Signalling/Trafficking** | | 370 | 455 | 0.79 | 0.91 | * |
| 1388764_at | Iqgap | | IQ motif containing GTPase activating protein 1 | **Signalling/Trafficking** | | 876 | 2174 | 0.78 | 0.89 | * |
| 1380855_at | Ksr1 | | Kinase suppressor of ras 1 | **Signalling/Trafficking** | | 212 | 235 | 1.26 | 1.17 | * |
| 1379282_at | Lrrfip2 | | Leucine rich repeat (in FLII) interacting protein 2 | **Signalling/Trafficking** | | 903 | 1082 | 1.30 | 1.10 | * |
| 1368871_at | Map3k1 | | Mitogen activated protein kinase kinase kinase 1 | **Signalling/Trafficking** | | 1006 | 1127 | 0.88 | 0.78 | # |
| 1391723_at | Map3k2 | | Mitogen-activated protein kinase kinase kinase 2 | **Signalling/Trafficking** | | 304 | 363 | 1.18 | 1.31 | # |
| 1368273_at | Mapk6 | | Mitogen-activated protein kinase 6 | **Signalling/Trafficking** | | 2032 | 1911 | 1.28 | 1.08 | * |
| 1393146_at | Mtmr9 | | Myotubularin related protein 9 | **Signalling/Trafficking** | | 401 | 628 | 0.93 | 0.79 | # |
| 1376282_at | Nuak1 | | NUAK family, SNF1-like kinase, 1 | **Signalling/Trafficking** | | 708 | 807 | 1.31 | 1.32 | *# |
| 1369655_at | Pik3c3 | | Phosphoinositide-3-kinase, class 3 | **Signalling/Trafficking** | | 273 | 438 | 0.72 | 0.79 | * |
| 1373532_at | Plekhf1 | | Pleckstrin homology domain containing, family F (with FYVE domain) member 1 | **Signalling/Trafficking** | | 1134 | 1082 | 0.79 | 0.78 | *# |
| 1387803_at | Ppp2r2b | | Protein phosphatase 2 (formerly 2A), regulatory subunit B (PR 52), beta isoform | **Signalling/Trafficking** | | 1273 | 1464 | 1.26 | 1.10 | * |
| 1389463_at | Prkar1b | | Protein kinase, cAMP dependent regulatory, type I, beta | **Signalling/Trafficking** | | 249 | 176 | 0.78 | 0.85 | * |
| 1373989_at | Rassf1 | | Ras association (RalGDS/AF-6) domain family 1 | **Signalling/Trafficking** | | 754 | 537 | 1.19 | 1.35 | # |
| 1368144_at, 1387074_at | Rgs2 | | Regulator of G-protein signaling 2 | **Signalling/Trafficking** | | 1725, 3188 | 1175, 1977 | 1.34 | 1.34 | *# |
| 1368505_at, 1368506_at | Rgs4 | | Regulator of G-protein signaling 4 | **Signalling/Trafficking** | | 1495, 695 | 1335, 704 | 0.81 | 0.72 | # |
| 1369958_at | Rhob | | Ras homolog gene family, member B | **Signalling/Trafficking** | | 3705 | 2376 | 1.29 | 1.50 | *# |
| 1372365_at | Rin2 | | Ras and Rab interactor 2 | **Signalling/Trafficking** | | 1313 | 1190 | 0.84 | 0.76 | # |
| 1392588_at | Ripk5 | | Receptor interacting protein kinase 5 | **Signalling/Trafficking** | | 380 | 614 | 0.83 | 0.75 | # |
| 1381533_at | Rnd1 | | Rho family GTPase 1 | **Signalling/Trafficking** | | 2128 | 1026 | 1.22 | 1.27 | # |
| 1390249_at | Sept14 | | Septin 14 | **Signalling/Trafficking** | | 598 | 560 | 1.29 | 1.27 | *# |
| 1383585_s_at | Snx10 | | Sorting nexin 10 | **Signalling/Trafficking** | | 462 | 317 | 0.78 | 0.89 | * |
| 1390555_at | Socs5 | | Suppressor of cytokine signaling 5 | **Signalling/Trafficking** | | 1707 | 2091 | 1.19 | 1.28 | # |
| 1389060_at | Socs7 | | Suppressor of cytokine signaling 7 | **Signalling/Trafficking** | | 436 | 309 | 1.14 | 1.34 | # |
| 1373164_at, 1387502_at | Stk17b | | Serine/threonine kinase 17b | **Signalling/Trafficking** | | 954, 592 | 1148, 929 | 0.79 | 0.86 | * |
| 1367765_at | Tcn2 | | Transcobalamin 2 | **Signalling/Trafficking** | | 1920 | 2661 | 0.87 | 0.79 | # |
| 1399088_at | Tlk2 | | Tousled-like kinase 2 | **Signalling/Trafficking** | | 745 | 834 | 1.27 | 1.10 | * |
| 1375760_at, 1391431_at | Tob2 | | Transducer of ERBB2, 2 | **Signalling/Trafficking** | | 333, 437 | 308, 542 | 1.21 | 1.43 | # |
| 1393809_at | Traf6 | | Tnf receptor-associated factor 6 | **Signalling/Trafficking** | | 557 | 258 | 1.09 | 1.30 | # |
| 1379488_at, 1381822_x_at | Trp53rk | | Transformation related protein 53 regulating kinase | **Signalling/Trafficking** | | 494, 545 | 271, 328 | 1.33 | 1.34 | *# |
| 1376680_at | Vps37c | | Vacuolar protein sorting 37C | **Signalling/Trafficking** | | 1156 | 824 | 1.29 | 1.11 | * |
| 1397386_at | Aff4 | | AF4/FMR2 family, member 4 | **Transcriptional regulation** | | 308 | 620 | 1.28 | 1.23 | * |
| 1367655_at | Ankrd1 | | Ankyrin repeat domain 1 (cardiac muscle) | **Transcriptional regulation** | | 7339 | 9018 | 0.63 | 0.68 | # |
| 1372964_at | Arid5b | | AT rich interactive domain 5B (Mrf1 like) | **Transcriptional regulation** | | 1352 | 1935 | 0.82 | 0.71 | # |
| 1379085_at | Asxl1 | | Additional sex combs like 1 (Drosophila) | **Transcriptional regulation** | | 482 | 736 | 0.82 | 0.78 | # |
| 1369268_at | Atf3 | | Activating transcription factor 3 | **Transcriptional regulation** | | 827 | 475 | 1.54 | 1.84 | *# |
| 1367624_at | Atf4 | | Activating transcription factor 4 | **Transcriptional regulation** | | 9396 | 5811 | 1.22 | 1.32 | # |
| 1385627_at, 1398482_at | Bcl3 | | B-cell leukemia/lymphoma 3 | **Transcriptional regulation** | | 715, 776 | 499, 455 | 1.12 | 1.27 | # |
| 1379368_at | Bcl6 | | B-cell leukemia/lymphoma 6 | **Transcriptional regulation** | | 518 | 682 | 0.71 | 0.72 | *# |
| 1368511_at | Bhlhb3 | | Basic helix-loop-helix domain containing, class B3 | **Transcriptional regulation** | | 1133 | 1098 | 1.34 | 1.34 | *# |
| 1377869_at | Ccrn4l | | Carbon catabolite repression 4 protein homolog | **Transcriptional regulation** | | 437 | 455 | 1.33 | 1.20 | * |
| 1368813_at, 1387343_at | Cebpd | | CCAAT/enhancer binding protein (C/EBP), delta | **Transcriptional regulation** | | 10813816 | 981, 2512 | 0.69 | 0.78 | *# |
| 1367601_at, 1367602_at | Cited2 | | Cbp/p300-interacting transactivator, with Glu/Asp-rich carboxy-terminal domain, 2 | **Transcriptional regulation** | | 1861, 1899 | 1314, 2091 | 0.79 | 0.75 | *# |
| 1392791_at | Egr3 | | Early growth response 3 | **Transcriptional regulation** | | 314 | 172 | 3.26 | 2.76 | *# |
| 1388714_at | Ell | | Elongation factor RNA polymerase II | **Transcriptional regulation** | | 1117 | 1056 | 0.87 | 0.75 | # |
| 1374055_at | Erf | | Ets2 repressor factor | **Transcriptional regulation** | | 1247 | 1014 | 1.12 | 1.34 | # |
| 1392220_at | Erg | | Avian erythroblastosis virus E-26 (v-ets) oncogene related | **Transcriptional regulation** | | 254 | 271 | 0.77 | 0.78 | * |
| 1393722_at | Fem1c | | Fem-1 homolog c | **Transcriptional regulation** | | 243 | 361 | 1.53 | 1.32 | * |
| 1393249_at | Fli1 | | Friend leukemia integration 1 (End of 3' UTR) | **Transcriptional regulation** | | 579 | 476 | 0.79 | 0.83 | * |
| 1368489_at | Fosl1 | | Fos-like antigen 1 | **Transcriptional regulation** | | 391 | 295 | 1.51 | 1.57 | *# |
| 1374125_at | Gata5 | | GATA binding factor-5 | **Transcriptional regulation** | | 426 | 538 | 1.31 | 1.27 | * |
| 1374223_at | Gmeb1 | | Glucocorticoid modulatory element binding protein 1 | **Transcriptional regulation** | | 497 | 486 | 1.26 | 1.06 | * |
| 1368549_at | Hbp1 | | High mobility group box transcription factor 1 | **Transcriptional regulation** | | 2132 | 2376 | 0.82 | 0.80 | # |
| 1387028_a_at | Id1 | | Inhibitor of DNA binding 1 | **Transcriptional regulation** | | 6337 | 3549 | 1.56 | 1.67 | *# |
| 1368870_at | Id2 | | Inhibitor of DNA binding 2 | **Transcriptional regulation** | | 1247 | 791 | 1.31 | 1.38 | # |
| 1394022_at | Id4 | | Inhibitor of DNA binding 4 | **Transcriptional regulation** | | 803 | 711 | 1.48 | 1.43 | *# |
| 1367795_at | Ifrd1 | | Interferon-related developmental regulator 1 | **Transcriptional regulation** | | 1465 | 1197 | 1.25 | 1.12 | * |
| 1390776_at | Irx3 | | Iroquois related homeobox 3 | **Transcriptional regulation** | | 1510 | 1685 | 1.36 | 1.24 | * |
| 1370975_at | Kdm3a | | Lysine (K)-specific demethylase 3A | **Transcriptional regulation** | | 536 | 1151 | 0.76 | 0.76 | *# |
| 1368650_at | Klf10 | | Kruppel-like factor 10 | **Transcriptional regulation** | | 1513 | 1399 | 2.09 | 1.95 | *# |
| 1379914_at | Klf11 | | Kruppel-like factor 11 | **Transcriptional regulation** | | 663 | 522 | 0.76 | 0.87 | * |
| 1368249_at, 1381396_s_at | Klf15 | | Kruppel-like factor 15 | **Transcriptional regulation** | | 1239, 305 | 941, 300 | 0.66 | 0.58 | *# |
| 1387260_at | Klf4 | | Kruppel-like factor 4 (gut) | **Transcriptional regulation** | | 1823 | 1242 | 1.23 | 1.40 | # |
| 1387060_at, 1388986_at, 1395557_at | Klf6 | | Kruppel-like factor 6 | **Transcriptional regulation** | | 2062, 1774, 697 | 959, 1895, 755 | 0.79 | 0.80 | *# |
| 1370209_at | Klf9 | | Kruppel-like factor 9 | **Transcriptional regulation** | | 2500 | 1630 | 0.90 | 0.79 | # |
| 1390907_at | LOC687219 | | Similar to zinc finger protein 84 (HPF2) | **Transcriptional regulation** | | 272 | 392 | 0.73 | 0.73 | # |
| 1383512_at | Mllt10 | | Myeloid/lymphoid or mixed-lineage leukemia (trithorax homolog, Drosophila); translocated to, 10 | **Transcriptional regulation** | | 357 | 420 | 0.77 | 0.87 | * |
| 1375929_at | Mnt | | Max binding protein | **Transcriptional regulation** | | 903 | 765 | 0.79 | 0.80 | *# |
| 1385125_at | Myocd | | Transcription factor myocardin | **Transcriptional regulation** | | 926 | 880 | 1.12 | 1.34 | # |
| 1375721_at | Nr2c2 | | Nuclear receptor subfamily 2, group C, member 2 | **Transcriptional regulation** | | 669 | 593 | 0.89 | 0.78 | # |
| 1398826_s_at | Nr2f6 | | Nuclear receptor subfamily 2, group F, member 6 | **Transcriptional regulation** | | 1281 | 688 | 1.12 | 1.29 | # |
| 1387152_at | Nrbf2 | | Nuclear receptor binding factor 2 | **Transcriptional regulation** | | 863 | 625 | 1.30 | 1.22 | * |
| 1385667_x_at | Pbx1 | | Pre B-cell leukemia transcription factor 1 | **Transcriptional regulation** | | 382 | 388 | 0.79 | 0.86 | * |
| 1370381_at | Pnrc1 | | Proline-rich nuclear receptor coactivator 1 | **Transcriptional regulation** | | 4615 | 3415 | 0.58 | 0.49 | *# |
| 1382400_at | Rlf | | Rearranged L-myc fusion sequence | **Transcriptional regulation** | | 364 | 502 | 0.72 | 0.78 | *# |
| 1390364_at | Runx1 | | Runt related transcription factor 1 | **Transcriptional regulation** | | 322 | 304 | 1.13 | 1.30 | # |
| 1372417_at | Sertad1 | | SERTA domain containing 1 | **Transcriptional regulation** | | 1804 | 870 | 1.44 | 1.27 | *# |
| 1372347_at | Skil | | SKI-like | **Transcriptional regulation** | | 1991 | 2699 | 1.39 | 1.28 | *# |
| 1373219_at | Snai1 | | Snail homolog 1 | **Transcriptional regulation** | | 1861 | 1250 | 1.58 | 1.49 | *# |
| 1385978_at, 1392813_at | Sox9 | | SRY-box containing gene 9 | **Transcriptional regulation** | | 349, 784 | 422, 853 | 1.35 | 1.45 | *# |
| 1388842_at | Srf | | Serum response factor | **Transcriptional regulation** | | 4065 | 2603 | 1.34 | 1.27 | *# |
| 1383889_at | Taf1a | | TATA box binding protein (Tbp)-associated factor, RNA polymerase I, A | **Transcriptional regulation** | | 244 | 264 | 1.20 | 1.33 | # |
| 1379469_at | Tbl1x | | Transducin (beta)-like 1 X-linked | **Transcriptional regulation** | | 485 | 181 | 1.29 | 1.35 | * |
| 1385641_at | Tnfaip3 | | Tumor necrosis factor, alpha-induced protein 3 | **Transcriptional regulation** | | 801 | 1101 | 0.77 | 0.73 | *# |
| 1368132_at | Tob1 | | Transducer of ERBB2, 1 | **Transcriptional regulation** | | 1044 | 1385 | 1.26 | 1.21 | * |
| 1375533_at, 1390436_at | Vgll4 | | Vestigial like 4 (Drosophila) | **Transcriptional regulation** | | 389, 2204 | 324, 1549 | 0.84 | 0.79 | # |
| 1373941_at | Yeats2 | | YEATS domain containing 2 | **Transcriptional regulation** | | 355 | 499 | 0.78 | 0.81 | * |
| 1373763_at | Zbtb43 | | Zinc finger and BTB domain containing 43 | **Transcriptional regulation** | | 448 | 509 | 0.76 | 0.91 | * |
| 1381902_at | Zfp292 | | Zinc finger protein 292 | **Transcriptional regulation** | | 260 | 487 | 0.80 | 0.85 | * |
| 1386721_at, 1393990_at | Zfp503 | | Zinc finger protein 503 | **Transcriptional regulation** | | 1054, 923 | 953, 952 | 1.12 | 1.27 | # |
| 1386837_x_at | Zfp574 | | Zinc finger protein 574 | **Transcriptional regulation** | | 199 | 242 | 1.42 | 1.35 | * |
| 1398377_at | Zfp672 | | Zinc finger protein 672 | **Transcriptional regulation** | | 484 | 440 | 1.19 | 1.38 | # |
| 1373680_at | Zfp697 | | Zinc finger protein 697 | **Transcriptional regulation** | | 389 | 426 | 1.36 | 1.26 | * |
| 1376917_at | Znf292 | | Zinc finger protein 292 | **Transcriptional regulation** | | 183 | 364 | 0.83 | 0.79 | # |
| 1372872_at | Zzz3 | | Zinc finger, ZZ domain containing 3 | **Transcriptional regulation** | | 250 | 352 | 1.41 | 1.27 | * |
| 1389230_at, 1394948_at | Arrdc3 | | Arrestin domain containing 3 | **Unknown function** | | 2272, 468 | 2632, 666 | 0.66 | 0.58 | *# |
| 1391808_at | Arrdc4 | | Arrestin domain containing 4 | **Unknown function** | | 425 | 419 | 1.37 | 1.26 | *# |
| 1374139_at | Cdr2 | | Cerebellar degeneration-related 2 | **Unknown function** | | 1145 | 609 | 1.34 | 1.49 | *# |
| 1388904_at | Dd25 | | Hypothetical protein Dd25 | **Unknown function** | | 308 | 538 | 1.28 | 1.09 | * |
| 1385369_at | Dzip1l | | DAZ interacting protein 1-like | **Unknown function** | | 525 | 614 | 1.26 | 1.07 | * |
| 1392818_at | Gas5 | | Growth arrest specific 5 | **Unknown function** | | 66 | 600 | 2.00 | 1.30 | # |
| 1382721_at | Ggnbp2 | | Gametogenetin binding protein 2 | **Unknown function** | | 1277 | 1526 | 1.28 | 1.22 | * |
| 1382111_at | Glod5 | | Glyoxalase domain containing 5 | **Unknown function** | | 813 | 780 | 0.93 | 0.78 | # |
| 1393128_at | Gpatch4 | | G patch domain containing 4 | **Unknown function** | | 212 | 135 | 1.39 | 1.50 | * |
| 1376076_at | Hig2 | | Hypoxia-inducible protein 2 | **Unknown function** | | 1556 | 934 | 0.80 | 0.82 | * |
| 1372389_at | Ier2 | | Immediate early response 2 | **Unknown function** | | 1177 | 637 | 1.25 | 1.36 | *# |
| 1389675_at, 1391026_at | Ier5l | | Immediate early response 5-like | **Unknown function** | | 944, 1820 | 637, 1234 | 1.42 | 1.43 | *# |
| 1376812_at | Ing5 | | Inhibitor of growth family, member 5 | **Unknown function** | | 370 | 325 | 1.29 | 1.21 | * |
| 1389727_at | Lrrc10 | | Leucine-rich repeat-containing 10 | **Unknown function** | | 1611 | 1318 | 0.86 | 0.74 | # |
| 1372060_at | Lysmd4 | | LysM, putative peptidoglycan-binding, domain containing 4 | **Unknown function** | | 704 | 467 | 0.78 | 0.74 | *# |
| 1377103_at | Midn | | Midnolin | **Unknown function** | | 2926 | 2392 | 1.36 | 1.24 | * |
| 1376342_at | Mier2 | | Mesoderm induction early response 1, family member 2 | **Unknown function** | | 515 | 403 | 1.19 | 1.33 | # |
| 1373496_at | Mkrn1 | | Makorin ring finger protein 1 | **Unknown function** | | 503 | 380 | 0.85 | 0.77 | # |
| 1367956_at | Ncdn | | Neurochondrin | **Unknown function** | | 341 | 358 | 1.25 | 1.23 | * |
| 1375925_at, 1395814_at | Palm2 | | Paralemmin 2 | **Unknown function** | | 216, 513 | 306, 520 | 1.16 | 1.28 | # |
| 1368860_at | Phlda1 | | Pleckstrin homology-like domain, family A, member 1 | **Unknown function** | | 221 | 181 | 1.44 | 1.51 | * |
| 1389809_at, 1392534_at | Pmepa1 | | Prostate transmembrane protein, androgen induced 1 | **Unknown function** | | 2133, 3258 | 1646, 1852 | 1.25 | 1.25 | *# |
| 1389254_at | Qser1 | | Glutamine and serine rich 1 | **Unknown function** | | 839 | 881 | 0.80 | 0.96 | * |
| 1370631_at | Reg3g | | Regenerating islet-derived 3 gamma | **Unknown function** | | 932 | 940 | 0.93 | 0.78 | # |
| 1373485_at | Rnf122 | | Ring finger protein 122 | **Unknown function** | | 799 | 559 | 0.76 | 0.85 | * |
| 1373936_at | Rnf44 | | Ring finger protein 44 | **Unknown function** | | 1703 | 1317 | 0.78 | 0.92 | * |
| 1386854_at | Samd4b | | Sterile alpha motif domain containing 4B | **Unknown function** | | 489 | 589 | 0.80 | 0.85 | * |
| 1372248_at | Sesn1 | | Sestrin 1 | **Unknown function** | | 577 | 564 | 0.75 | 0.90 | * |
| 1372633_at | Spg20 | | Spastic paraplegia 20, spartin (Troyer syndrome) homolog | **Unknown function** | | 615 | 718 | 0.80 | 0.93 | * |
| 1384821_at | Thap1 | | THAP domain containing, apoptosis associated protein 1 | **Unknown function** | | 436 | 287 | 1.14 | 1.32 | # |
| 1395343_at | Tm9sf3 | | Transmembrane 9 superfamily member 3 | **Unknown function** | | 445 | 1342 | 0.73 | 0.79 | * |
| 1374484_at | Tmem39a | | Transmembrane protein 39a | **Unknown function** | | 1038 | 928 | 1.29 | 1.18 | * |
| 1381376_at | Tnfaip2 | | Tumor necrosis factor, alpha-induced protein 2 | **Unknown function** | | 264 | 328 | 1.13 | 1.30 | # |
| 1390793_at | Ttc33 | | Tetratricopeptide repeat domain 33 | **Unknown function** | | 318 | 344 | 0.79 | 0.76 | # |
| 1376492_at | Unkl | | Unkempt homolog (Drosophila)-like | **Unknown function** | | 469 | 511 | 0.74 | 0.86 | * |
| 1398380_at | Vwa1 | | von Willebrand factor A domain containing 1 | **Unknown function** | | 537 | 357 | 0.76 | 0.82 | * |
| 1385699_at | Ythdf1 | | YTH domain family 1 | **Unknown function** | | 621 | 521 | 1.17 | 1.37 | # |
| 1371816_at | Zcch3 | | Zinc finger, CCHC domain containing 3 | **Unknown function** | | 751 | 793 | 1.32 | 1.13 | * |
| 1373767_at | Zfand2a | | Zinc finger, AN1-type domain 2A | **Unknown function** | | 766 | 636 | 1.28 | 1.08 | * |
| 1373606_at | Zfand2b | | Zinc finger, AN1 type domain 2B | **Unknown function** | | 372 | 208 | 1.27 | 1.10 | * |
| 1373786_at | Zfp703 | | Zinc finger protein 703 | **Unknown function** | | 301 | 227 | 1.34 | 1.43 | * |
