# Supplementary material for: Regulation of the cardiomyocyte transcriptome vs translatome by endothelin-1 and insulin: translational regulation of 5' terminal oligopyrimidine tract (TOP) mRNAs by insulin
Source: BMC Genomics. 2010 May 29;11:343. doi: 10.1186/1471-2164-11-343 (PMC2900265; doi:10.1186/1471-2164-11-343)
Supplement: Additional file 5 — Regulation of polysomal and total RNA expression in cardiomyocytes in response to ET-1 (Microsoft Word Table). Neonatal rat cardiomyocytes were exposed to ET-1 (100 nM, 1 h) or left unstimulated (controls). Total and polysomal RNA were analysed using Affymetrix rat genome 230 2.0 microarrays. The data were normalised to controls. Transcripts with differential expression in ET-1-treated cells relative to controls were identified (>1.25-fold difference; * FDR < 0.05 ET-1 vs Controls for polysomal RNA, # FDR < 0.05 ET-1 vs Controls for total RNA, t-test with Benjamini and Hochberg false discovery rate correction). Mean raw fluorescence values are provided for controls, and mean expression relative to controls is provided for ET-1-treated cells (n = 4). For transcripts represented by more than one probeset, the probesets and mean corresponding raw values are listed. RNAs are listed according to translational regulation and in order of functional category then alphabetical order of the gene symbol. AS = Antisense. [file 1471-2164-11-343-S5.DOC]

**Additional file 5. Regulation of polysomal and total RNA expression in cardiomyocytes in response to ET-1.** Neonatal rat cardiomyocytes were exposed to ET-1 (100 nM, 1 h) or left unstimulated (controls). Total and polysomal RNA were analysed using Affymetrix rat genome 230 2.0 microarrays. The data were normalised to controls. Transcripts with differential expression in ET-1-treated cells relative to controls were identified (>1.25-fold difference; * FDR<0.05 ET-1 *vs* Controls for polysomal RNA, # FDR<0.05 ET-1 *vs* Controls for total RNA, t-test with Benjamini and Hochberg false discovery rate correction). Mean raw fluorescence values are provided for controls, and mean expression relative to controls is provided for ET-1-treated cells (n=4). For transcripts represented by more than one probeset, the probesets and mean corresponding raw values are listed. RNAs are listed according to translational regulation and in order of functional category then alphabetical order of the gene symbol. AS = Antisense.

| **Probeset** | **Gene symbol** | | **Gene title** | | | | **Function** | **Control**  **(raw values)** | | **ET-1**  **(relative to control)** | |  |
| --- | --- | --- | --- | --- | --- | --- | --- | --- | --- | --- | --- | --- |
|  |  |  |  |  |  |  |  | **Polysomal RNA** | **Total RNA** | **Polysomal RNA** | **Total RNA** |  |
| **Translationally regulated (FDR<0.05 in polysomal RNA or total RNA AND ratio of P:T or T:P >1.2-fold** | | | | | | | | --- | --- | --- | --- |  |
| ***Regulated in polysomal RNA (not total RNA)*** | | | | | | | | --- | --- | --- | --- |  |
| 1383721_at | Fzd8 | | | Frizzled homolog 8 (Drosophila) | | **Agonists/receptors** | | 354 | 788 | 1.40 | 0.96 | * |
| 1387625_at | Igfbp6 | | | Insulin-like growth factor binding protein 6 | | **Agonists/receptors** | | 414 | 226 | 0.70 | 0.90 | * |
| 1390901_at | Igsf10 | | | Immunoglobulin superfamily, member 10 | | **Agonists/receptors** | | 468 | 1304 | 1.32 | 0.97 | * |
| 1370957_at | Il6st | | | Interleukin 6 signal transducer | | **Agonists/receptors** | | 3944 | 4153 | 0.75 | 1.01 | * |
| 1389020_at | Islr | | | Immunoglobulin superfamily containing leucine-rich repeat | | **Cell-cell/matrix adhesion** | | 457 | 453 | 0.78 | 0.98 | * |
| 1397535_at | Mpp5 | | | Membrane protein, palmitoylated 5 | | **Cell-cell/matrix adhesion** | | 322 | 487 | 1.27 | 1.01 | * |
| 1369110_x_at | RT1-Aw2 | | | RT1 class Ib, locus Aw2 | | **Cell-cell/matrix adhesion** | | 248 | 260 | 1.25 | 0.83 | * |
| 1373146_at | Ssx2ip | | | Synovial sarcoma, X breakpoint 2 interacting protein | | **Cell-cell/matrix adhesion** | | 501 | 359 | 0.75 | 0.94 | * |
| 1382431_at | Abca1 | | | ATP-binding cassette, sub-family A (ABC1), member 1 | | **Channels/transporters** | | 416 | 1032 | 1.25 | 0.87 | * |
| 1376683_at | Abcb7 | | | ATP-binding cassette, sub-family B (MDR/TAP), member 7 | | **Channels/transporters** | | 253 | 773 | 0.79 | 0.96 | * |
| 1387019_at | ATP5i | | | ATP synthase, H+ transporting, mitochondrial F0 complex, subunit e | | **Channels/transporters** | | 5413 | 4287 | 0.76 | 0.99 | * |
| 1390483_at | Slc25a29 | | | Solute carrier family 25 (mitochondrial carrier, palmitoylcarnitine transporter), member 29 | | **Channels/transporters** | | 390 | 285 | 0.62 | 0.90 | * |
| 1373282_at | Slc25a33 | | | Solute carrier family 25, member 33 | | **Channels/transporters** | | 308 | 253 | 1.45 | 0.97 | * |
| 1372595_at | Actn2 | | | Actinin alpha 2 | | **Cytoskeleton/myofibrillar** | | 3671 | 11095 | 0.77 | 1.03 | * |
| 1371607_at | Map4 | | | Microtubule-associated protein 4 | | **Cytoskeleton/myofibrillar** | | 1041 | 1576 | 0.77 | 0.94 | * |
| 1396057_at | Mtss1l | | | Metastasis suppressor 1-like | | **Cytoskeleton/myofibrillar** | | 326 | 262 | 1.30 | 0.94 | * |
| 1390469_at | Nrm | | | Nurim (nuclear envelope membrane protein) | | **Cytoskeleton/myofibrillar** | | 252 | 206 | 0.66 | 0.85 | * |
| 1398365_at | Tppp3 | | | Tubulin polymerization-promoting protein family member 3 | | **Cytoskeleton/myofibrillar** | | 284 | 245 | 1.58 | 0.87 | * |
| 1371618_s_at | Tubb3 | | | Tubulin, beta 3 | | **Cytoskeleton/myofibrillar** | | 466 | 300 | 0.80 | 1.01 | * |
| 1389993_at | Wdr33 | | | WD repeat domain 33 | | **DNA structure/repair** | | 758 | 958 | 0.80 | 0.99 | * |
| 1393891_at | Col8a1 | | | Procollagen, type VIII, alpha 1 | | **Extracellular matrix** | | 416 | 409 | 0.74 | 0.95 | * |
| 1373210_at | Lamb1 | | | Laminin, beta 1 | | **Extracellular matrix** | | 1723 | 4181 | 0.71 | 0.87 | * |
| 1375180_at | Marveld1 | | | MARVEL (membrane-associating) domain containing 1 | | **Hypothetical proteins** | | 393 | 811 | 1.38 | 1.05 | * |
| 1372213_at | LOC500300 | | | Similar to hypothetical protein MGC6835 | | **Hypothetical protein** | | 2255 | 2624 | 1.44 | 1.09 | * |
| 1371735_at | LOC682880 | | | Hypothetical protein LOC682880 | | **Hypothetical protein** | | 1239 | 1704 | 1.37 | 0.91 | * |
| 1373403_at | LOC684871 | | | Similar to Protein C8orf4 (Thyroid cancer protein 1) (TC-1) | | **Hypothetical protein** | | 100 | 86 | 5.68 | 2.61 | * |
| 1390824_at | RGD1304963 | | | Similar to hypothetical protein MGC38716 | | **Hypothetical protein** | | 353 | 286 | 0.61 | 0.82 | * |
| 1371747_at | RGD1310660 | | | Similar to RIKEN cDNA 2700038C09 | | **Hypothetical protein** | | 1943 | 1055 | 0.79 | 0.98 | * |
| 1380314_at, 1393074_at | RGD1563072 | | | Similar to hypothetical protein FLJ38984 | | **Hypothetical protein** | | 434, 481 | 202, 245 | 0.62 | 0.84 | * |
| 1376801_at | RGD1564450 | | | Hypothetical protein RGD1564450 | | **Hypothetical protein** | | 722 | 341 | 0.78 | 1.02 | * |
| 1371689_at | Unknown | | | Unknown | | **No established gene** | | 1326 | 2838 | 1.33 | 0.92 | * |
| 1373115_at | Unknown | | | Unknown | | **No established gene** | | 593 | 483 | 0.68 | 0.83 | * |
| 1374746_at | Unknown | | | Unknown | | **No established gene** | | 140 | 154 | 1.67 | 1.21 | * |
| 1376078_at | Unknown | | | Unknown | | **No established gene** | | 234 | 349 | 1.27 | 0.91 | * |
| 1382538_at | Unknown | | | Unknown | | **No established gene** | | 332 | 293 | 0.80 | 1.14 | * |
| 1383040_a_at | Unknown | | | Unknown | | **No established gene** | | 1163 | 1859 | 0.74 | 0.99 | * |
| 1383041_x_at | Unknown | | | Unknown | | **No established gene** | | 508 | 881 | 0.76 | 0.98 | * |
| 1386850_x_at | Unknown | | | Unknown | | **No established gene** | | 268 | 268 | 1.53 | 0.86 | * |
| 1390165_at | Unknown | | | Unknown | | **No established gene** | | 439 | 434 | 0.78 | 0.97 | * |
| 1391649_a_at | Unknown | | | Unknown | | **No established gene** | | 478 | 484 | 0.73 | 1.08 | * |
| 1391721_at | Unknown | | | Unknown | | **No established gene** | | 356 | 778 | 0.76 | 0.93 | * |
| 1392131_at | Unknown | | | Unknown | | **No established gene** | | 297 | 457 | 1.27 | 0.94 | * |
| 1392184_at | Unknown | | | Unknown | | **No established gene** | | 246 | 143 | 1.66 | 1.27 | * |
| 1383030_at | AS:Gna11 | | | AS:Guanine nucleotide binding protein, alpha 11 | | **Non-protein coding** | | 330 | 301 | 1.30 | 0.95 | * |
| 1394181_at | AS:Ndnl2 | | | AS:Necdin-like 2 | | **Non-protein coding** | | 254 | 228 | 0.67 | 1.24 | * |
| 1379866_at | Intron:Sept6 | | | Intron:Septin 6 | | **Non-protein coding** | | 550 | 638 | 0.78 | 0.98 | * |
| 1397734_at | Intron:Sp3 | | | Intron:Sp3 transcription factor | | **Non-protein coding** | | 174 | 214 | 1.38 | 1.03 | * |
| 1377719_a_at | Mitochondrial genome | | | Mitochondrial genome (D loop region) | | **Non-protein coding** | | 1303 | 4732 | 0.67 | 0.93 | * |
| 1375022_at | Afg3l2 | | | AFG3(ATPase family gene 3)-like 2 (yeast) | | **Protein processing** | | 318 | 723 | 1.42 | 0.93 | * |
| 1390520_at | Dnajb3 | | | DnaJ (Hsp40) homolog, subfamily B, member 3 | | **Protein processing** | | 233 | 140 | 1.43 | 1.11 | * |
| 1381903_at | Fbxo33 | | | F-box only protein 33 | | **Protein processing** | | 253 | 282 | 1.27 | 0.82 | * |
| 1377688_at | Grpel2 | | | GrpE-like 2, mitochondrial | | **Protein processing** | | 352 | 286 | 0.62 | 0.98 | * |
| 1391450_at | Loxl2 | | | Lysyl oxidase-like 2 | | **Protein processing** | | 995 | 1274 | 0.70 | 1.02 | * |
| 1373087_at | March7 | | | Membrane-associated ring finger (C3HC4) 7 or axotrophin | | **Protein processing** | | 1460 | 2585 | 1.33 | 1.10 | * |
| 1378225_at | Mmp14 | | | Matrix metallopeptidase 14 (membrane-inserted) | | **Protein processing** | | 1225 | 1296 | 0.62 | 0.99 | * |
| 1386912_at | Pcolce | | | Procollagen C-endopeptidase enhancer protein | | **Protein processing** | | 6448 | 6767 | 0.78 | 0.97 | * |
| 1388484_at | Ube2c | | | Ubiquitin-conjugating enzyme E2C | | **Protein processing** | | 1024 | 620 | 0.76 | 1.09 | * |
| 1372586_at | Ube2d3 | | | Ubiquitin-conjugating enzyme E2D 3 | | **Protein processing** | | 733 | 797 | 1.35 | 1.12 | * |
| 1387658_at | Eef2k | | | Eukaryotic elongation factor-2 kinase | | **Protein synthesis** | | 396 | 286 | 1.37 | 0.93 | * |
| 1377287_at | Mars2 | | | Methionine-tRNA synthetase 2 (mitochondrial) | | **Protein synthesis** | | 355 | 518 | 1.28 | 0.98 | * |
| 1388413_at | Rrbp1 | | | Ribosome binding protein 1 homolog 180kDa (dog) | | **Protein synthesis** | | 819 | 2104 | 0.60 | 0.95 | * |
| 1367828_at | Acads | | | Acyl-Coenzyme A dehydrogenase, C-2 to C-3 short chain | | **Regulation of metabolism** | | 1147 | 1188 | 0.78 | 1.38 | * |
| 1367999_at | Aldh2 | | | Aldehyde dehydrogenase 2 | | **Regulation of metabolism** | | 1746 | 2876 | 0.69 | 1.04 | * |
| 1382451_at | Hebp2 | | | Heme binding protein 2 | | **Regulation of metabolism** | | 633 | 536 | 0.78 | 0.95 | * |
| 1395632_at | Mthfsd | | | Methenyltetrahydrofolate synthetase domain containing | | **Regulation of metabolism** | | 234 | 158 | 0.62 | 1.01 | * |
| 1372147_at | Ndufs4 | | | NADH dehydrogenase (ubiquinone) Fe-S protein 4 | | **Regulation of metabolism** | | 6257 | 2121 | 0.68 | 0.91 | * |
| 1375213_at | Pck2 | | | Phosphoenolpyruvate carboxykinase 2 (mitochondrial) | | **Regulation of metabolism** | | 937 | 1179 | 0.78 | 1.01 | * |
| 1375084_at | Serinc2 | | | Serine incorporator 2 | | **Regulation of metabolism** | | 471 | 496 | 0.77 | 1.14 | * |
| 1397551_at | Ddx27 | | | DEAD (Asp-Glu-Ala-Asp) box polypeptide 27 | | **RNA regulation** | | 233 | 399 | 1.25 | 0.85 | * |
| 1376254_at | Ints1 | | | Integrator complex subunit 1 | | **RNA regulation** | | 325 | 423 | 1.32 | 1.09 | * |
| 1389684_at | Prpf39 | | | PRP39 pre-mRNA processing factor 39 homolog (yeast) | | **RNA regulation** | | 410 | 1116 | 1.32 | 1.02 | * |
| 1371975_at | Rbms2 | | | RNA binding motif, single stranded interacting protein 2 | | **RNA regulation** | | 718 | 602 | 0.78 | 1.04 | * |
| 1399101_at | Rnpc2 | | | RNA-binding region (RNP1, RRM) containing 2 | | **RNA regulation** | | 1682 | 3636 | 1.31 | 1.01 | * |
| 1368992_a_at | Sfrs5 | | | Splicing factor, arginine/serine-rich 5 | | **RNA regulation** | | 2390 | 3568 | 1.42 | 1.12 | * |
| 1390960_at | Azi2 | | | 5-azacytidine induced gene 2 | | **Signalling/Trafficking** | | 242 | 288 | 1.48 | 1.07 | * |
| 1374307_at | Camk2n1 | | | Calcium/calmodulin-dependent protein kinase II inhibitor 1 | | **Signalling/Trafficking** | | 1510 | 771 | 0.79 | 1.03 | * |
| 1368545_at | Cflar | | | CASP8 and FADD-like apoptosis regulator | | **Signalling/Trafficking** | | 206 | 184 | 1.30 | 1.02 | * |
| 1399022_at | Clk1 | | | CDC-like kinase 1 | | **Signalling/Trafficking** | | 447 | 1900 | 1.41 | 1.09 | * |
| 1375231_a_at | Cxxc5 | | | CXXC finger 5 | | **Signalling/Trafficking** | | 3133 | 1758 | 0.72 | 0.99 | * |
| 1381564_at | Glmn | | | Glomulin, FKBP associated protein | | **Signalling/Trafficking** | | 301 | 374 | 1.26 | 0.93 | * |
| 1391906_at | Kif1b | | | Kinesin family member 1B | | **Signalling/Trafficking** | | 314 | 535 | 0.79 | 1.16 | * |
| 1377994_at | Pmaip1 | | | Phorbol-12-myristate-13-acetate-induced protein 1 | | **Signalling/Trafficking** | | 292 | 160 | 1.68 | 1.28 | * |
| 1389463_at | Prkar1b | | | Protein kinase, cAMP dependent regulatory, type I, beta | | **Signalling/Trafficking** | | 249 | 176 | 0.75 | 0.91 | * |
| 1389692_at | Rab22a | | | RAB22A, member RAS oncogene family | | **Signalling/Trafficking** | | 483 | 332 | 0.70 | 0.88 | * |
| 1389700_at | Rasa2 | | | RAS p21 protein activator 2 | | **Signalling/Trafficking** | | 211 | 405 | 1.35 | 1.06 | * |
| 1397620_at | Sh3md2 | | | Putative scaffolding protein POSH | | **Signalling/Trafficking** | | 200 | 191 | 1.30 | 1.06 | * |
| 1383910_at | Sh3rf1 | | | SH3 domain containing ring finger 1 | | **Signalling/Trafficking** | | 864 | 541 | 1.32 | 1.04 | * |
| 1384222_at | Shoc2 | | | Soc-2 (suppressor of clear) homolog (C. elegans) | | **Signalling/Trafficking** | | 535 | 567 | 1.34 | 1.03 | * |
| 1369584_at, 1377092_at | Socs3 | | | Suppressor of cytokine signaling 3 | | **Signalling/Trafficking** | | 549, 2377 | 608, 2233 | 0.64 | 0.79 | * |
| 1375565_at | Timm22 | | | Translocase of inner mitochondrial membrane 22 homolog | | **Signalling/Trafficking** | | 617 | 370 | 0.78 | 1.03 | * |
| 1384313_at | Trappc5 | | | Trafficking protein particle complex 5 | | **Signalling/Trafficking** | | 2260 | 1560 | 0.69 | 0.84 | * |
| 1387557_s_at | Vps33a | | | Vacuolar protein sorting 33A (yeast) | | **Signalling/Trafficking** | | 525 | 509 | 0.68 | 0.81 | * |
| 1372301_at | Aebp1 | | | AE binding protein 1 | | **Transcriptional regulation** | | 1782 | 2979 | 0.72 | 0.89 | * |
| 1389564_at | Ccnl2 | | | Cyclin L2 | | **Transcriptional regulation** | | 677 | 2048 | 1.51 | 1.07 | * |
| 1368550_at | Foxq1 | | | Forkhead box Q1 | | **Transcriptional regulation** | | 172 | 148 | 1.77 | 1.33 | * |
| 1367535_at | Irf2bp1 | | | Interferon regulatory factor 2 binding protein 1 | | **Transcriptional regulation** | | 921 | 831 | 0.76 | 0.99 | * |
| 1379567_at | Thrap1 | | | Thyroid hormone receptor associated protein 1 | | **Transcriptional regulation** | | 179 | 248 | 1.48 | 1.23 | * |
| 1391879_at | Tox2 | | | TOX high mobility group box family member 2 | | **Transcriptional regulation** | | 184 | 211 | 1.41 | 0.72 | * |
| 1385472_at | Zbtb8 | | | Zinc finger and BTB domain containing 8 | | **Transcriptional regulation** | | 319 | 293 | 0.70 | 0.85 | * |
| 1393140_at | Zc3h12a | | | Zinc finger CCCH type containing 12A | | **Transcriptional regulation** | | 372 | 266 | 1.43 | 1.16 | * |
| 1390862_at | Zfp629 | | | Zinc finger protein 629 | | **Transcriptional regulation** | | 473 | 678 | 0.68 | 0.92 | * |
| 1385606_at | Brp16 | | | Brain protein 16 | | **Unknown function** | | 384 | 360 | 0.77 | 1.00 | * |
| 1383205_at | Dact2 | | | Dapper homolog 2, antagonist of beta-catenin (xenopus) | | **Unknown function** | | 283 | 190 | 0.61 | 0.85 | * |
| 1388904_at | Dd25 | | | Hypothetical protein Dd25 | | **Unknown function** | | 308 | 538 | 1.49 | 1.14 | * |
| 1377173_at | Drctnnb1a | | | Down-regulated by Ctnnb1, a | | **Unknown function** | | 1101 | 743 | 0.78 | 0.99 | * |
| 1372898_at | Heatr6 | | | HEAT repeat containing 6 | | **Unknown function** | | 310 | 348 | 0.80 | 1.02 | * |
| 1377153_a_at | Klhl6 | | | Kelch-like 6 (Drosophila) | | **Unknown function** | | 259 | 269 | 0.78 | 1.05 | * |
| 1373233_at, 1378179_a_at | Lhfpl2 | | | Lipoma HMGIC fusion partner-like 2 | | **Unknown function** | | 971, 716 | 949, 747 | 1.35 | 1.10 | * |
| 1392207_at | Luc7l | | | LUC7-like (S. cerevisiae) | | **Unknown function** | | 257 | 277 | 0.67 | 0.84 | * |
| 1390139_a_at | Obsl1 | | | Obscurin-like 1 | | **Unknown function** | | 682 | 942 | 0.69 | 0.85 | * |
| 1383306_at, 1385691_at | Spty2d1 | | | SPT2, Suppressor of Ty, domain containing 1 (S. cerevisiae) | | **Unknown function** | | 592, 200 | 1092, 371 | 1.61 | 1.26 | * |
| 1374948_at | Tmem106a | | | Transmembrane protein 106A | | **Unknown function** | | 286 | 233 | 0.73 | 0.98 | * |
| --- | --- | | | --- | | --- | | --- | --- | --- | --- |  |
| ***Regulated in total RNA (not polysomal RNA)*** | | | | | | | | --- | --- | --- | --- |  |
| 1369633_at | Cxcl12 | | | Chemokine (C-X-C motif) ligand 12 | | **Agonists/receptors** | | 3463 | 2704 | 0.95 | 0.71 | # |
| 1376150_at | Edg3 | | | Endothelial differentiation, sphingolipid G-protein-coupled receptor, 3 | | **Agonists/receptors** | | 1210 | 1278 | 0.96 | 0.70 | # |
| 1369519_at | Edn1 | | | Endothelin 1 | | **Agonists/receptors** | | 295 | 276 | 1.26 | 1.64 | # |
| 1387935_at | Il3ra | | | Interleukin 3 receptor, alpha chain | | **Agonists/receptors** | | 199 | 280 | 1.05 | 0.76 | # |
| 1382566_at | Il7r | | | Interleukin 7 receptor | | **Agonists/receptors** | | 295 | 492 | 0.93 | 0.75 | # |
| 1368725_at | Jag1 | | | Jagged 1 | | **Agonists/receptors** | | 169 | 256 | 0.86 | 1.25 | # |
| 1385299_at | Lpar4 | | | lysophosphatidic acid receptor 4 | | **Agonists/receptors** | | 217 | 283 | 1.02 | 0.63 | # |
| 1376101_at | Lrp6 | | | Low density lipoprotein receptor-related protein 6 | | **Agonists/receptors** | | 521 | 815 | 1.01 | 0.77 | # |
| 1389797_at | Lrp8 | | | Low density lipoprotein receptor-related protein 8, apolipoprotein e receptor | | **Agonists/receptors** | | 215 | 369 | 1.19 | 0.66 | # |
| 1383119_at | Ogfrl1 | | | Opioid growth factor receptor-like 1 | | **Agonists/receptors** | | 198 | 301 | 1.02 | 0.69 | # |
| 1390450_a_at | Ogn | | | Osteoglycin | | **Agonists/receptors** | | 469 | 631 | 0.94 | 0.69 | # |
| 1370941_at | Pdgfra | | | Platelet derived growth factor receptor, alpha polypeptide | | **Agonists/receptors** | | 2980 | 4289 | 0.89 | 0.72 | # |
| 1387172_a_a, 1388011_a_at | Tgfb2 | | | Transforming growth factor, beta 2 | | **Agonists/receptors** | | 932, 610 | 1434, 889 | 0.90 | 1.31 | # |
| 1392280_at | Tlr2 | | | Toll-like receptor 2 | | **Agonists/receptors** | | 464 | 731 | 0.93 | 0.71 | # |
| 1368641_at | Wnt4 | | | Wingless-related MMTV integration site 4 | | **Agonists/receptors** | | 1192 | 450 | 0.80 | 0.65 | # |
| 1372481_at | Cd34 | | | CD34 antigen | | **Cell-cell/matrix adhesion** | | 1000 | 940 | 0.93 | 0.76 | # |
| 1368518_at | Cd53 | | | Cd53 molecule | | **Cell-cell/matrix adhesion** | | 855 | 825 | 0.98 | 0.78 | # |
| 1392863_at | Flrt3 | | | Fibronectin leucine rich transmembrane protein 3 | | **Cell-cell/matrix adhesion** | | 115 | 172 | 1.15 | 1.72 | # |
| 1382183_at | Mpp7 | | | Membrane protein, palmitoylated 7 (MAGUK p55 subfamily member 7) | | **Cell-cell/matrix adhesion** | | 412 | 891 | 0.97 | 0.78 | # |
| 1373232_at | Nid2 | | | Nidogen 2 | | **Cell-cell/matrix adhesion** | | 646 | 474 | 1.06 | 1.55 | # |
| 1367721_at | Sdc4 | | | Syndecan 4 | | **Cell-cell/matrix adhesion** | | 5899 | 4134 | 1.17 | 1.43 | # |
| 1367893_a_at | Clcc1 | | | Chloride channel CLIC-like 1 | | **Channels/transporters** | | 205 | 412 | 0.97 | 0.76 | # |
| 1387420_at | Clic4 | | | Chloride intracellular channel 4 | | **Channels/transporters** | | 633 | 1171 | 1.02 | 1.45 | # |
| 1369640_at | Gja1 | | | Gap junction membrane channel protein alpha 1 | | **Channels/transporters** | | 529 | 2041 | 1.00 | 1.58 | # |
| 1369782_a_at 1387698_at, 1391007_s_at | Kcnj11 | | | Potassium inwardly rectifying channel, subfamily J, member 11 | | **Channels/transporters** | | 274, 346, 608 | 367, 377, 545 | 0.81 | 0.63 | # |
| 1389988_at | Kctd2 | | | Potassium channel tetramerisation domain containing protein 2 | | **Channels/transporters** | | 1615 | 1013 | 0.82 | 0.61 | # |
| 1376605_at | Slc17a5 | | | Solute carrier family 17 (anion/sugar transporter), member 5 | | **Channels/transporters** | | 468 | 591 | 0.99 | 0.79 | # |
| 1375080_at | Slc25a45 | | | Solute carrier family 25, member 45 | | **Channels/transporters** | | 489 | 369 | 1.02 | 0.76 | # |
| 1370848_at | Slc2a1 | | | Solute carrier family 2 (facilitated glucose transporter), member 1 | | **Channels/transporters** | | 2155 | 2803 | 1.18 | 1.42 | # |
| 1372326_at | Slc2a3 | | | Solute carrier family 2 (facilitated glucose transporter), member 3 | | **Channels/transporters** | | 640 | 640 | 1.11 | 1.38 | # |
| 1382919_at | Slc39a14 | | | Solute carrier family 39 (zinc transporter), member 14 | | **Channels/transporters** | | 290 | 614 | 1.14 | 1.44 | # |
| 1369704_at | Slc6a20 | | | Solute carrier family 6 (neurotransmitter transporter), member 20 | | **Channels/transporters** | | 289 | 524 | 1.12 | 0.72 | # |
| 1392705_at | Slc9a6 | | | Solute carrier family 9 (sodium/hydrogen exchanger), member 6 | | **Channels/transporters** | | 129 | 255 | 1.06 | 1.35 | # |
| 1370857_at | Acta2 | | | smooth muscle alpha-actin | | **Cytoskeleton/myofibrillar** | | 6780 | 7006 | 1.14 | 1.47 | # |
| AFFX_Rat_beta-actin_M_at | Actb | | | Actin, beta | | **Cytoskeleton/myofibrillar** | | 15490 | 14148 | 1.06 | 1.28 | # |
| 1373535_at, 1396450_at | Enah | | | Enabled homolog (Drosophila) | | **Cytoskeleton/myofibrillar** | | 2054, 376 | 2108, 371 | 1.16 | 1.69 | # |
| 1390049_at | Fhl1 | | | Four and a half LIM domains 1 | | **Cytoskeleton/myofibrillar** | | 4064 | 5158 | 1.01 | 1.35 | # |
| 1381678_at, 1385359_at | Fscn1 | | | Fascin homolog 1, actin bundling protein (Strongylocentrotus purpuratus) | | **Cytoskeleton/myofibrillar** | | 427, 525 | 393, 387 | 0.93 | 1.37 | # |
| 1370291_at | Pdlim3 | | | PDZ and LIM domain 3 | | **Cytoskeleton/myofibrillar** | | 3134 | 2581 | 0.99 | 1.27 | # |
| 1376802_at | Tchp | | | Trichoplein, keratin filament binding | | **Cytoskeleton/myofibrillar** | | 278 | 260 | 0.97 | 0.79 | # |
| 1372500_at | Tmod3 | | | Tropomodulin 3 | | **Cytoskeleton/myofibrillar** | | 816 | 914 | 1.04 | 0.79 | # |
| 1395132_at | Utrn | | | Utrophin | | **Cytoskeleton/myofibrillar** | | 72 | 252 | 0.90 | 1.31 | # |
| 1387227_at | Wipf1 | | | WAS/WASL interacting protein family, member 1 | | **Cytoskeleton/myofibrillar** | | 1272 | 1033 | 0.96 | 0.78 | # |
| 1372792_at | Alkbh6 | | | AlkB, alkylation repair homolog 6 (E. coli) | | **DNA structure/repair** | | 517 | 438 | 0.95 | 0.69 | # |
| 1392830_at | Chd1 | | | Chromodomain helicase DNA binding protein 1 | | **DNA structure/repair** | | 399 | 562 | 0.98 | 1.28 | # |
| 1371332_at | Hist1h1c | | | Histone cluster 1, H1c | | **DNA structure/repair** | | 7343 | 5749 | 0.94 | 0.73 | # |
| 1385595_at | Kin | | | Antigenic determinant of rec-A protein | | **DNA structure/repair** | | 257 | 334 | 0.96 | 0.79 | # |
| 1377846_a_at | Msh6 | | | mutS homolog 6 (E. coli) | | **DNA structure/repair** | | 410 | 611 | 0.87 | 0.71 | # |
| 1378010_at | Orc4l | | | Origin recognition complex, subunit 4-like (S. cerevisiae) | | **DNA structure/repair** | | 283 | 603 | 1.03 | 0.67 | # |
| 1377617_at | Pura | | | Purine rich element binding protein A | | **DNA structure/repair** | | 1631 | 1398 | 0.91 | 0.75 | # |
| 1390534_at | Smc5 | | | Structural maintenance of chromosomes 5 | | **DNA structure/repair** | | 258 | 631 | 0.94 | 0.78 | # |
| 1394884_s_at | Ssbp1 | | | Single-stranded DNA binding protein 1 | | **DNA structure/repair** | | 2189 | 1489 | 0.97 | 0.80 | # |
| 1369421_at | Top1 | | | Topoisomerase (DNA) I | | **DNA structure/repair** | | 160 | 343 | 0.92 | 1.27 | # |
| 1393210_at | Ecm2 | | | Extracellular matrix protein 2 | | **Extracellular matrix** | | 154 | 371 | 0.90 | 0.73 | # |
| 1389424_at | LOC365157 | | | Similar to ribosomal protein L22 proprotein | | **Hypothetical protein** | | 105 | 301 | 0.87 | 0.67 | # |
| 1381434_s_at | LOC681027 | | | Hypothetical protein LOC681027 | | **Hypothetical protein** | | 909 | 611 | 1.12 | 1.39 | # |
| 1390907_at | LOC691170 | | | Similar to zinc finger protein 84 (HPF2) | | **Hypothetical protein** | | 272 | 392 | 0.81 | 0.65 | # |
| 1398607_at | RGD1305680 | | | Similar to KIAA0240 | | **Hypothetical protein** | | 416 | 372 | 1.02 | 0.74 | # |
| 1397681_at | RGD1305793 | | | Similar to hypothetical protein FLJ20154 | | **Hypothetical protein** | | 441 | 649 | 0.84 | 0.52 | # |
| 1384084_at | RGD1308106 | | | Hypothetical protein LOC361719 | | **Hypothetical protein** | | 254 | 254 | 1.07 | 1.32 | # |
| 1373547_at | RGD1308147 | | | Similar to expressed sequence AW209491 | | **Hypothetical protein** | | 857 | 586 | 0.82 | 0.68 | # |
| 1379367_at | RGD1308251 | | | Similar to RIKEN cDNA 2810405K02 | | **Hypothetical protein** | | 658 | 395 | 1.06 | 0.76 | # |
| 1397179_at | RGD1309388 | | | Similar to DIP13 alpha | | **Hypothetical protein** | | 273 | 413 | 1.00 | 1.28 | # |
| 1392960_at | RGD1309906 | | | Similar to RIKEN cDNA 2310004I24 gene | | **Hypothetical protein** | | 1045 | 758 | 0.94 | 0.74 | # |
| 1372202_at | RGD1310553 | | | Similar to expressed sequence AI597479 | | **Hypothetical protein** | | 873 | 654 | 0.86 | 0.71 | # |
| 1374212_at | RGD1311358 | | | Similar to RIKEN cDNA 2410017P07 | | **Hypothetical protein** | | 313 | 413 | 1.02 | 0.76 | # |
| 1388407_at | RGD1311925 | | | Similar to BC003940 protein | | **Hypothetical protein** | | 2957 | 1514 | 0.96 | 0.78 | # |
| 1383641_at, 1393415_at | RGD1559432 | | | Hypothetical protein RGD1559432 | | **Hypothetical protein** | | 1142, 584 | 1887, 1276 | 0.97 | 0.69 | # |
| 1380766_a_at | RGD1563510 | | | Similar to RIKEN cDNA 8430427H17 gene | | **Hypothetical protein** | | 519 | 486 | 1.05 | 0.79 | # |
| 1371189_x_at | RGD1563757 | | | Similar to 40S ribosomal protein SA (p40) | | **Hypothetical protein** | | 2677 | 4134 | 1.02 | 0.80 | # |
| 1390197_at | RGD1565146 | | | Similar to hypothetical gene supported by AF226663 | | **Hypothetical protein** | | 340 | 410 | 1.00 | 0.75 | # |
| 1381174_at | RGD735029 | | | SEL1 domain containing protein RGD735029 | | **Hypothetical protein** | | 474 | 366 | 0.94 | 0.68 | # |
| 1372509_at | Unknown | | | Unknown | | **No established gene** | | 668 | 631 | 0.94 | 0.77 | # |
| 1373651_at | Unknown | | | Unknown | | **No established gene** | | 98 | 388 | 1.09 | 1.80 | # |
| 1373696_at | Unknown | | | Unknown | | **No established gene** | | 856 | 687 | 1.00 | 0.80 | # |
| 1374243_at | Unknown | | | Unknown | | **No established gene** | | 293 | 269 | 0.85 | 0.66 | # |
| 1375191_at | Unknown | | | Unknown | | **No established gene** | | 215 | 329 | 1.04 | 0.75 | # |
| 1375698_at | Unknown | | | Unknown | | **No established gene** | | 285 | 432 | 0.85 | 0.58 | # |
| 1376376_at | Unknown | | | Unknown | | **No established gene** | | 474 | 415 | 0.81 | 0.62 | # |
| 1376403_at | Unknown | | | Unknown | | **No established gene** | | 94 | 193 | 1.06 | 2.17 | # |
| 1376617_at | Unknown | | | Unknown | | **No established gene** | | 465 | 722 | 0.81 | 0.62 | # |
| 1376882_at | Unknown | | | Unknown | | **No established gene** | | 616 | 910 | 0.92 | 0.74 | # |
| 1377551_at | Unknown | | | Unknown | | **No established gene** | | 54 | 803 | 0.77 | 1.27 | # |
| 1379602_at | Unknown | | | Unknown | | **No established gene** | | 222 | 232 | 1.08 | 1.48 | # |
| 1380152_at | Unknown | | | Unknown | | **No established gene** | | 404 | 576 | 0.98 | 0.80 | # |
| 1382354_at | Unknown | | | Unknown | | **No established gene** | | 130 | 258 | 1.14 | 0.73 | # |
| 1383901_at | Unknown | | | Unknown | | **No established gene** | | 565 | 414 | 1.09 | 0.77 | # |
| 1384135_at | Unknown | | | Unknown | | **No established gene** | | 291 | 446 | 0.78 | 0.63 | # |
| 1384310_at | Unknown | | | Unknown | | **No established gene** | | 250 | 345 | 1.05 | 0.73 | # |
| 1384475_at | Unknown | | | Unknown | | **No established gene** | | 417 | 356 | 1.02 | 0.78 | # |
| 1385305_at | Unknown | | | Unknown | | **No established gene** | | 222 | 343 | 0.95 | 0.77 | # |
| 1385322_at | Unknown | | | Unknown | | **No established gene** | | 260 | 322 | 0.88 | 0.72 | # |
| 1389419_at | Unknown | | | Unknown | | **No established gene** | | 374 | 504 | 0.65 | 0.54 | # |
| 1389990_at | Unknown | | | Unknown | | **No established gene** | | 274 | 312 | 0.90 | 1.33 | # |
| 1392598_at | Unknown | | | Unknown | | **No established gene** | | 2126 | 1170 | 1.06 | 1.31 | # |
| 1392842_at | Unknown | | | Unknown | | **No established gene** | | 428 | 542 | 0.95 | 0.64 | # |
| 1393020_at | Unknown | | | Unknown | | **No established gene** | | 607 | 445 | 1.14 | 0.78 | # |
| 1393853_at | Unknown | | | Unknown | | **No established gene** | | 434 | 399 | 1.04 | 0.71 | # |
| 1394747_at | Unknown | | | Unknown | | **No established gene** | | 332 | 233 | 1.04 | 1.33 | # |
| 1395147_at | Unknown | | | Unknown | | **No established gene** | | 178 | 251 | 1.13 | 1.40 | # |
| 1398213_at | Unknown | | | Unknown | | **No established gene** | | 80 | 387 | 0.77 | 1.48 | # |
| 1374290_at, 1396435_at | Mirn145 precursor | | | MicroRNA 145 precursor | | **Non-protein coding** | | 87, 87 | 286, 178 | 1.14 | 1.85 | # |
| 1378867_at | Mirn221 precursor | | | MicroRNA 221 precursor | | **Non-protein coding** | | 24 | 80 | 1.43 | 8.09 | # |
| 1382017_at | Mirn322 precursor | | | MicroRNA 322 precursor | | **Non-protein coding** | | 769 | 341 | 1.06 | 1.38 | # |
| 1385205_at | Mirn99a precursor | | | MicroRNA 99a precursor | | **Non-protein coding** | | 22 | 287 | 0.66 | 1.41 | # |
| 1375532_at | AS:Id2 | | | AS:inhibitor of DNA binding 2 | | **Non-protein coding** | | 5422 | 3831 | 0.86 | 0.70 | # |
| 1398037_at | AS:Plk2 | | | AS:Polo-like kinase 2 (Drosophila) | | **Non-protein coding** | | 60 | 128 | 2.03 | 2.83 | # |
| 1379747_at | AS:Prss35 | | | AS:Protease, serine, 35 | | **Non-protein coding** | | 235 | 425 | 1.19 | 1.53 | # |
| 1384000_at | AS:Sox4 | | | AS:SRY-box containing gene 4 | | **Non-protein coding** | | 624 | 584 | 0.99 | 0.73 | # |
| 1378866_at | Intron:Ablim1 | | | Intron:Actin-binding LIM protein 1 | | **Non-protein coding** | | 118 | 447 | 1.11 | 0.77 | # |
| 1396539_at | Intron:Actn1 | | | Intron:Actinin, alpha 1 | | **Non-protein coding** | | 38 | 230 | 2.15 | 6.19 | # |
| 1391753_at | Intron:Ankrd1 | | | Intron:Ankyrin repeat domain 1 (cardiac muscle) | | **Non-protein coding** | | 371 | 556 | 1.63 | 2.14 | # |
| 1394451_at | Intron:Anxa1 | | | Intron:Annexin A1 | | **Non-protein coding** | | 43 | 325 | 2.16 | 2.61 | # |
| 1380195_at | Intron:Atp2b1 | | | Intron:ATPase, Ca++ transporting, plasma membrane 1 | | **Non-protein coding** | | 113 | 254 | 1.04 | 1.50 | # |
| 1390723_at | Intron:Ctnna1 | | | Intron:Catenin (cadherin-associated protein), alpha 1 | | **Non-protein coding** | | 39 | 201 | 2.01 | 3.30 | # |
| 1375475_at | Intron:Dusp5 | | | Intron:Dual specificity phosphatase 5 | | **Non-protein coding** | | 20 | 64 | 2.36 | 6.35 | # |
| 1394750_at | Intron:Fhl1 | | | Intron:Four and a half LIM domains 1 | | **Non-protein coding** | | 86 | 119 | 1.22 | 3.72 | # |
| 1383828_at | Intron:Klf14 | | | Intron:Kruppel-like factor 14 | | **Non-protein coding** | | 47 | 76 | 2.76 | 5.11 | # |
| 1396877_at | Intron:Lamc1 | | | Intron:Laminin, gamma 1 | | **Non-protein coding** | | 81 | 506 | 1.43 | 1.93 | # |
| 1379089_at, 1380155_at | Intron:Myh9 | | | Intron:Myosin, heavy polypeptide 9, non-muscle | | **Non-protein coding** | | 57, 262 | 331, 755 | 1.18 | 2.25 | # |
| 1398595_at | Intron:Rbm5 | | | Intron:RNA binding motif protein 5 | | **Non-protein coding** | | 64 | 322 | 1.30 | 0.76 | # |
| 1391215_at | Intron:Samd4 | | | Intron:Sterile alpha motif domain containing 4 | | **Non-protein coding** | | 125 | 190 | 0.95 | 1.60 | # |
| 1382020_at | Intron:Spag9 | | | Intron:sperm associated antigen 9 | | **Non-protein coding** | | 109 | 346 | 1.08 | 1.64 | # |
| 1378831_at | Intron:Srgap2 | | | Intron:SLIT-ROBO Rho GTPase activating protein 2 | | **Non-protein coding** | | 98 | 226 | 1.14 | 1.38 | # |
| 1380701_at | Intron:Ssfa2 | | | Intron:sperm specific antigen 2 | | **Non-protein coding** | | 70 | 255 | 1.34 | 1.83 | # |
| 1395443_at | Intron:Tmem49 | | | Intron:Transmembrane protein 49 | | **Non-protein coding** | | 52 | 318 | 0.93 | 0.71 | # |
| 1391841_at, 1395350_at | Intron:Tpm1 | | | Intron:Tropomyosin 1, alpha | | **Non-protein coding** | | 681, 120 | 1461, 234 | 1.15 | 1.46 | # |
| 1398691_at | Intron:TSC22d2 | | | Intron:TSC22 domain family 2 | | **Non-protein coding** | | 12 | 58 | 3.00 | 5.17 | # |
| 1389616_at | Intron:Whsc1 | | | Intron:Wolf-Hirschhorn syndrome candidate 1 | | **Non-protein coding** | | 223 | 399 | 1.12 | 0.78 | # |
| 1395130_at | Intron:Zfhx1b | | | Intron:Zinc finger E-box binding homeobox 2 | | **Non-protein coding** | | 42 | 212 | 0.91 | 1.26 | # |
| 1394483_at | Adamts5 | | | A disintegrin-like and metallopeptidase (reprolysin type) with thrombospondin type 1 motif, 5 (aggrecanase-2) | | **Protein processing** | | 677 | 730 | 1.22 | 0.80 | # |
| 1392422_at | Alg9 | | | Asparagine-linked glycosylation 9 homolog (yeast, alpha 1,2 mannosyltransferase) | | **Protein processing** | | 767 | 727 | 0.86 | 1.37 | # |
| 1375595_at | Arih1 | | | Ariadne homolog, ubiquitin-conjugating enzyme E2 binding protein, 1 (Drosophila) | | **Protein processing** | | 427 | 427 | 1.08 | 1.37 | # |
| 1389047_at | Bag2 | | | Bcl2-associated athanogene 2 | | **Protein processing** | | 1587 | 1111 | 1.12 | 1.37 | # |
| 1387446_at | C1galt1 | | | Core 1 UDP-galactose:N-acetylgalactosamine-alpha-R beta 1,3-galactosyltransferase | | **Protein processing** | | 414 | 365 | 0.98 | 1.44 | # |
| 1372400_at | Cul4a | | | Cullin 4A | | **Protein processing** | | 1263 | 1260 | 0.93 | 0.71 | # |
| 1384511_at | Dnajb5 | | | DnaJ (Hsp40) homolog, subfamily B, member 5 | | **Protein processing** | | 306 | 354 | 1.18 | 1.57 | # |
| 1380476_at | Dnajc1 | | | DnaJ (Hsp40) homolog, subfamily C, member 1 | | **Protein processing** | | 182 | 282 | 1.03 | 1.31 | # |
| 1390168_a_at | Dnajc24 | | | DnaJ (Hsp40) homolog, subfamily C, member 24 | | **Protein processing** | | 440 | 347 | 1.06 | 0.78 | # |
| 1372600_at | Fbxo31 | | | F-box only protein 31 | | **Protein processing** | | 873 | 659 | 0.95 | 0.72 | # |
| 1380500_s_at | Fkbp2 | | | FK506 binding protein 2 | | **Protein processing** | | 3185 | 2037 | 0.95 | 0.71 | # |
| 1375872_at | Fktn | | | Fukutin | | **Protein processing** | | 309 | 424 | 1.04 | 0.75 | # |
| 1388887_at | Ggta1 | | | Glycoprotein, alpha-galactosyltransferase 1 | | **Protein processing** | | 663 | 769 | 1.05 | 0.80 | # |
| 1372649_at, 1392435_at | Hspb7 | | | Heat shock 27kD protein family, member 7 (cardiovascular) | | **Protein processing** | | 11667, 2226 | 8856, 1655 | 1.02 | 1.35 | # |
| 1371424_at | Jmjd5 | | | Jumonji domain containing 5 | | **Protein processing** | | 641 | 663 | 0.91 | 0.70 | # |
| 1372659_at | Kcmf1 | | | Potassium channel modulatory factor 1 | | **Protein processing** | | 3602 | 3377 | 1.11 | 0.76 | # |
| 1372623_at | Mlec | | | Malectin | | **Protein processing** | | 793 | 631 | 0.96 | 1.27 | # |
| 1390142_at | Morc3 | | | Microrchidia 3 | | **Protein processing** | | 273 | 666 | 1.08 | 0.80 | # |
| 1388585_at | Ostm1 | | | Osteopetrosis associated transmembrane protein 1 | | **Protein processing** | | 1060 | 948 | 1.00 | 0.79 | # |
| 1392890_at | Pafah1b1 | | | Platelet-activating factor acetylhydrolase, isoform Ib, alpha subunit 45kDa | | **Protein processing** | | 200 | 605 | 0.96 | 0.79 | # |
| 1374454_at | Pcmtd2 | | | Protein-L-isoaspartate (D-aspartate) O-methyltransferase domain containing 2 | | **Protein processing** | | 801 | 1073 | 1.10 | 0.79 | # |
| 1370196_at | Pias3 | | | Protein inhibitor of activated STAT 3 | | **Protein processing** | | 760 | 615 | 0.92 | 0.76 | # |
| 1382385_at | Psmc6 | | | Proteasome (prosome, macropain) 26S subunit, ATPase, 6 | | **Protein processing** | | 379 | 423 | 0.95 | 0.62 | # |
| 1378524_at | Rnf19 | | | Ring finger protein (C3HC4 type) 19 | | **Protein processing** | | 583 | 716 | 1.08 | 1.31 | # |
| 1373592_at | Serpinb9 | | | Serine (or cysteine) peptidase inhibitor, clade B, member 9 | | **Protein processing** | | 1666 | 1175 | 1.01 | 1.29 | # |
| 1369617_at | Ube2n | | | Ubiquitin-conjugating enzyme E2N | | **Protein processing** | | 418 | 401 | 1.01 | 1.48 | # |
| 1373438_at | Ube2o | | | ubiquitin-conjugating enzyme E2O | | **Protein processing** | | 421 | 704 | 1.05 | 0.79 | # |
| 1371934_at | Usp21 | | | Ubiquitin specific peptidase 21 | | **Protein processing** | | 789 | 500 | 0.99 | 0.78 | # |
| 1385873_at | Usp42 | | | Ubiquitin specific protease 42 | | **Protein processing** | | 573 | 773 | 0.92 | 0.74 | # |
| 1383204_at | Znrf1 | | | Zinc and ring finger 1 | | **Protein processing** | | 340 | 542 | 0.93 | 0.67 | # |
| 1380447_a_at | Mrps18c | | | Mitochondrial ribosomal protein S18C | | **Protein synthesis** | | 781 | 680 | 0.94 | 0.77 | # |
| 1376415_at | Mrrf | | | Mitochondrial ribosome recycling factor | | **Protein synthesis** | | 540 | 459 | 1.00 | 0.77 | # |
| 1384314_at | Mtif2 | | | Mitochondrial translational initiation factor 2 | | **Protein synthesis** | | 146 | 431 | 0.95 | 0.79 | # |
| 1374990_at | Rpl22 | | | Ribosomal protein L22 | | **Protein synthesis** | | 887 | 675 | 0.89 | 0.74 | # |
| 1390383_at | Adfp | | | Adipose differentiation related protein | | **Regulation of metabolism** | | 4343 | 4307 | 1.09 | 1.41 | # |
| 1371748_at | Agpat1 | | | 1-acylglycerol-3-phosphate O-acyltransferase 1 | | **Regulation of metabolism** | | 1013 | 541 | 0.93 | 1.26 | # |
| 1368095_at | Ak3 | | | Adenylate kinase 3 | | **Regulation of metabolism** | | 3427 | 1993 | 0.91 | 0.75 | # |
| 1367775_at | Amacr | | | Alpha-methylacyl-CoA racemase | | **Regulation of metabolism** | | 335 | 384 | 1.01 | 0.78 | # |
| 1369520_a_at | Bcat1 | | | Branched chain aminotransferase 1, cytosolic | | **Regulation of metabolism** | | 394 | 483 | 1.02 | 1.31 | # |
| 1374006_at | Ccbl2 | | | Cysteine conjugate-beta lyase 2 | | **Regulation of metabolism** | | 311 | 370 | 1.07 | 0.79 | # |
| 1384192_at | Chst1 | | | Carbohydrate (keratan sulfate Gal-6) sulfotransferase 1 | | **Regulation of metabolism** | | 1568 | 623 | 0.77 | 0.49 | # |
| 1373304_at | Coasy | | | Coenzyme A synthase | | **Regulation of metabolism** | | 356 | 366 | 0.89 | 0.73 | # |
| 1397902_at | Cox15 | | | COX15 homolog, cytochrome c oxidase assembly protein | | **Regulation of metabolism** | | 273 | 558 | 1.09 | 0.79 | # |
| 1382616_at | Gls | | | Glutaminase | | **Regulation of metabolism** | | 67 | 300 | 0.78 | 1.47 | # |
| 1373797_at | Hyal3 | | | Hyaluronoglucosaminidase 3 | | **Regulation of metabolism** | | 492 | 471 | 0.90 | 0.74 | # |
| 1373627_at | Inpp5k | | | Inositol polyphosphate 5-phosphatase K | | **Regulation of metabolism** | | 350 | 422 | 1.00 | 0.78 | # |
| 1382516_at | Kdsr | | | 3-ketodihydrosphingosine reductase | | **Regulation of metabolism** | | 175 | 272 | 1.04 | 0.74 | # |
| 1389358_at | Lpgat1 | | | Lysophosphatidylglycerol acyltransferase 1 | | **Regulation of metabolism** | | 1002 | 1252 | 1.04 | 0.75 | # |
| 1371479_at | Mettl7a | | | Methyltransferase like 7A | | **Regulation of metabolism** | | 636 | 499 | 0.86 | 0.71 | # |
| 1383025_at | Mgea5 | | | Meningioma expressed antigen 5 (hyaluronidase) | | **Regulation of metabolism** | | 270 | 400 | 1.06 | 1.32 | # |
| 1376214_at | Mmachc | | | Methylmalonic aciduria (cobalamin deficiency) cblC type, with homocystinuria | | **Regulation of metabolism** | | 356 | 319 | 0.93 | 0.77 | # |
| 1374025_at | Nmnat3 | | | Nicotinamide nucleotide adenylyltransferase 3 | | **Regulation of metabolism** | | 615 | 398 | 1.06 | 0.75 | # |
| 1382874_at | Np1 | | | N-acetylneuraminate pyruvate lyase | | **Regulation of metabolism** | | 283 | 337 | 1.25 | 0.74 | # |
| 1367892_at | Pdk2 | | | Pyruvate dehydrogenase kinase, isozyme 2 | | **Regulation of metabolism** | | 1628 | 1481 | 1.05 | 0.80 | # |
| 1372765_a_at | Peci | | | Peroxisomal D3,D2-enoyl-CoA isomerase | | **Regulation of metabolism** | | 521 | 551 | 0.96 | 0.75 | # |
| 1390391_at | Pfkfb3 | | | 6-phosphofructo-2-kinase/fructose-2,6-biphosphatase 3 | | **Regulation of metabolism** | | 385 | 559 | 0.83 | 0.67 | # |
| 1386145_at | Plscr4 | | | Phospholipid scramblase 4 | | **Regulation of metabolism** | | 310 | 483 | 1.08 | 0.69 | # |
| 1382331_at | Rfk | | | Riboflavin kinase | | **Regulation of metabolism** | | 492 | 467 | 1.05 | 1.36 | # |
| 1368275_at | Sc4mol | | | Sterol-C4-methyl oxidase-like | | **Regulation of metabolism** | | 4908 | 4908 | 1.21 | 1.47 | # |
| 1375889_at | Sms | | | Spermine synthase | | **Regulation of metabolism** | | 743 | 460 | 0.96 | 0.77 | # |
| 1374588_at | Sumf1 | | | Sulfatase modifying factor 1 | | **Regulation of metabolism** | | 398 | 368 | 0.99 | 0.79 | # |
| 1386632_at | Tmem55a | | | Transmembrane protein 55A | | **Regulation of metabolism** | | 1408 | 1222 | 1.01 | 0.79 | # |
| 1370881_at | Tst | | | Thiosulfate sulfurtransferase, mitochondrial | | **Regulation of metabolism** | | 1505 | 1417 | 0.97 | 0.78 | # |
| 1386958_at | Txnrd1 | | | Thioredoxin reductase 1 | | **Regulation of metabolism** | | 772 | 1169 | 1.10 | 1.45 | # |
| 1382957_at | Crop | | | Cisplatin resistance-associated overexpressed protein | | **RNA regulation** | | 261 | 355 | 0.86 | 0.72 | # |
| 1392548_at | Cwc22 | | | CWC22 spliceosome-associated protein homolog | | **RNA regulation** | | 134 | 346 | 0.97 | 0.68 | # |
| 1371698_at | Eftud2 | | | Elongation factor Tu GTP binding domain containing 2 | | **RNA regulation** | | 1172 | 1478 | 0.96 | 1.44 | # |
| 1395455_at | Hnrnph3 | | | Heterogeneous nuclear ribonucleoprotein H3 (2H9) | | **RNA regulation** | | 225 | 259 | 1.00 | 0.80 | # |
| 1389726_at | Lsm11 | | | U7 snRNP-specific Sm-like protein LSM11 | | **RNA regulation** | | 299 | 319 | 0.87 | 0.68 | # |
| 1377187_at | Rbm12b | | | RNA binding motif protein 12B | | **RNA regulation** | | 126 | 294 | 0.74 | 0.61 | # |
| 1377787_at | Rbm6 | | | RNA binding motif protein 6 | | **RNA regulation** | | 380 | 545 | 1.03 | 0.73 | # |
| 1394155_at | Rbm7 | | | RNA binding motif protein 7 | | **RNA regulation** | | 401 | 287 | 1.15 | 2.05 | # |
| 1377815_at | Rpp14 | | | Ribonuclease P 14 subunit (human) | | **RNA regulation** | | 2852 | 1701 | 1.03 | 0.74 | # |
| 1368229_at | Sip1 | | | Survival of motor neuron protein interacting protein 1 | | **RNA regulation** | | 600 | 553 | 1.01 | 0.77 | # |
| 1383107_at | Snrpd1 | | | Small nuclear ribonucleoprotein D1 | | **RNA regulation** | | 968 | 442 | 1.01 | 1.25 | # |
| 1374688_at | Tial1 | | | Tia1 cytotoxic granule-associated RNA binding protein-like 1 | | **RNA regulation** | | 284 | 549 | 0.96 | 0.69 | # |
| 1382674_a_at | Tnrc6c | | | Trinucleotide repeat containing 6C | | **RNA regulation** | | 226 | 287 | 0.94 | 1.29 | # |
| 1385521_at | Trub2 | | | TruB pseudouridine (psi) synthase homolog 2 (E. coli) | | **RNA regulation** | | 542 | 341 | 1.02 | 0.80 | # |
| 1373205_at | U2af1l4 | | | U2 small nuclear RNA auxiliary factor 1-like 4 | | **RNA regulation** | | 681 | 515 | 0.96 | 0.68 | # |
| 1394975_at | Zfml | | | Zinc finger, matrin-like | | **RNA regulation** | | 179 | 294 | 0.89 | 1.29 | # |
| 1371451_at | Rnaseh2a | | | Ribonuclease H2, subunit A | | **RNA regulation** | | 1310 | 681 | 1.04 | 0.78 | # |
| 1385757_at | Intron:Homer1 | | | Intron:homer homolog 1 (Drosophila) | | **Sequences in introns** | | 153 | 228 | 1.03 | 1.59 | # |
| 1377725_at | Anp32a | | | Acidic (leucine-rich) nuclear phosphoprotein 32 family, member A | | **Signalling/Trafficking** | | 595 | 523 | 0.95 | 0.77 | # |
| 1377239_at | Apbb1ip | | | Amyloid beta (A4) precursor protein-binding, family B, member 1 interacting protein | | **Signalling/Trafficking** | | 540 | 519 | 1.02 | 1.47 | # |
| 1387447_at | Arf3 | | | ADP-ribosylation factor 3 | | **Signalling/Trafficking** | | 351 | 347 | 0.93 | 1.27 | # |
| 1391101_at | Arhgap12 | | | Rho GTPase activating protein 12 | | **Signalling/Trafficking** | | 179 | 359 | 1.00 | 0.71 | # |
| 1367747_at | Arl3 | | | ADP-ribosylation factor-like 3 | | **Signalling/Trafficking** | | 1687 | 921 | 0.96 | 0.78 | # |
| 1371733_at | Arl8a | | | ADP-ribosylation factor-like 8A | | **Signalling/Trafficking** | | 2123 | 1126 | 1.02 | 1.30 | # |
| 1371719_at | Brd2 | | | Bromodomain-containing 2 | | **Signalling/Trafficking** | | 950 | 863 | 1.24 | 1.55 | # |
| 1372536_at | Cabc1 | | | Chaperone, ABC1 activity of bc1 complex like (S. pombe) | | **Signalling/Trafficking** | | 388 | 585 | 0.86 | 0.68 | # |
| 1371953_at | Ccng2 | | | Cyclin G2 | | **Signalling/Trafficking** | | 958 | 904 | 0.84 | 0.58 | # |
| 1369040_at | Cdc42bpa | | | CDC42 binding protein kinase alpha | | **Signalling/Trafficking** | | 157 | 209 | 1.06 | 1.31 | # |
| 1388530_at | Cdc42se1 | | | CDC42 small effector 1 | | **Signalling/Trafficking** | | 1786 | 1275 | 1.02 | 1.30 | # |
| 1391503_at | Centd3 | | | Centaurin, delta 3 | | **Signalling/Trafficking** | | 414 | 527 | 0.96 | 0.77 | # |
| 1369912_at | Crk | | | v-crk sarcoma virus CT10 oncogene homolog (avian) | | **Signalling/Trafficking** | | 317 | 411 | 1.09 | 1.40 | # |
| 1376788_at | Dapk1 | | | Death associated protein kinase 1 | | **Signalling/Trafficking** | | 1778 | 2003 | 1.01 | 0.80 | # |
| 1387237_at | Exoc7 | | | Exocyst complex component 7 | | **Signalling/Trafficking** | | 203 | 275 | 0.95 | 0.76 | # |
| 1390050_at | Golm1 | | | Golgi membrane protein 1 | | **Signalling/Trafficking** | | 214 | 432 | 1.03 | 0.68 | # |
| 1369352_at | Hipk3 | | | Homeodomain interacting protein kinase 3 | | **Signalling/Trafficking** | | 335 | 577 | 1.02 | 1.42 | # |
| 1367876_at | Ipo13 | | | Importin 13 | | **Signalling/Trafficking** | | 680 | 559 | 0.95 | 0.79 | # |
| 1388858_at | Map2k3 | | | Mitogen activated protein kinase kinase 3 | | **Signalling/Trafficking** | | 4963 | 2245 | 1.08 | 1.62 | # |
| 1393146_at | Mtmr9 | | | Myotubularin related protein 9 | | **Signalling/Trafficking** | | 401 | 628 | 0.96 | 0.67 | # |
| 1394448_at | Osbpl6 | | | Oxysterol binding protein-like 6 | | **Signalling/Trafficking** | | 266 | 401 | 1.05 | 0.78 | # |
| 1393184_at | Pak2 | | | p21 (CDKN1A)-activated kinase 2 | | **Signalling/Trafficking** | | 262 | 353 | 1.05 | 1.31 | # |
| 1383327_at | Pdcd4 | | | Programmed cell death 4 | | **Signalling/Trafficking** | | 454 | 461 | 1.04 | 0.76 | # |
| 1369655_at | Pik3c3 | | | Phosphoinositide-3-kinase, class 3 | | **Signalling/Trafficking** | | 273 | 438 | 0.92 | 0.62 | # |
| 1376989_at | Pik3r3 | | | Phosphatidylinositol 3 kinase, regulatory subunit, polypeptide 3 | | **Signalling/Trafficking** | | 267 | 438 | 0.96 | 1.33 | # |
| 1392560_at | Pip5k1a | | | Phosphatidylinositol-4-phosphate 5-kinase, type 1, alpha | | **Signalling/Trafficking** | | 898 | 977 | 0.74 | 1.27 | # |
| 1375767_at | Plekhg3 | | | Pleckstrin homology domain containing, family G (with RhoGef domain) member 3 | | **Signalling/Trafficking** | | 189 | 298 | 0.85 | 0.67 | # |
| 1381850_at | Ppp1r12a | | | Protein phosphatase 1, regulatory (inhibitor) subunit 12A | | **Signalling/Trafficking** | | 204 | 591 | 1.13 | 1.48 | # |
| 1395236_at | Ppp1r3c | | | protein phosphatase 1, regulatory (inhibitor) subunit 3C | | **Signalling/Trafficking** | | 215 | 389 | 0.86 | 0.65 | # |
| 1372176_at | Prkca | | | Protein kinase C, alpha | | **Signalling/Trafficking** | | 204 | 313 | 0.98 | 1.26 | # |
| 1398143_at | Prkx | | | Protein kinase, X-linked | | **Signalling/Trafficking** | | 155 | 200 | 1.05 | 1.35 | # |
| 1376705_a_at | Rab24 | | | Rab24, member Ras oncogene family | | **Signalling/Trafficking** | | 1274 | 1002 | 1.03 | 0.79 | # |
| 1372636_at | Rab40c | | | Rab40c, member RAS oncogene family | | **Signalling/Trafficking** | | 576 | 349 | 0.88 | 0.72 | # |
| 1374284_at | Rassf4 | | | Ras association (RalGDS/AF-6) domain family 4 | | **Signalling/Trafficking** | | 520 | 402 | 0.92 | 0.72 | # |
| 1368506_at | Rgs4 | | | Regulator of G-protein signaling 4 | | **Signalling/Trafficking** | | 695 | 704 | 1.03 | 0.74 | # |
| 1392588_at | Ripk5 | | | Receptor interacting protein kinase 5 | | **Signalling/Trafficking** | | 380 | 614 | 0.86 | 0.66 | # |
| 1382058_at | Rras2 | | | Related RAS viral (r-ras) oncogene homolog 2 | | **Signalling/Trafficking** | | 2796 | 2208 | 1.06 | 1.28 | # |
| 1377174_at | Sbf1 | | | SET binding factor 1 | | **Signalling/Trafficking** | | 286 | 337 | 0.96 | 1.33 | # |
| 1390695_at | Scyl3 | | | SCY1-like 3 (S. cerevisiae) | | **Signalling/Trafficking** | | 171 | 277 | 0.82 | 0.64 | # |
| 1371882_a_at | Sh2bpsm1 | | | SH2-B PH domain containing signaling mediator 1 | | **Signalling/Trafficking** | | 1652 | 1102 | 1.06 | 0.78 | # |
| 1373239_at | Sh3px3 | | | SH3 and PX domain containing 3 | | **Signalling/Trafficking** | | 984 | 846 | 0.92 | 0.70 | # |
| 1379662_a_at 1386282_x_at | Snrk | | | SNF related kinase | | **Signalling/Trafficking** | | 1577, 255 | 1601, 285 | 0.88 | 0.70 | # |
| 1377458_at | Sorl1 | | | Sortilin-related receptor, LDLR class A repeats-containing | | **Signalling/Trafficking** | | 407 | 752 | 1.23 | 0.58 | # |
| 1390864_at | Sos1 | | | Son of sevenless homolog 1 (Drosophila) | | **Signalling/Trafficking** | | 221 | 284 | 0.95 | 0.75 | # |
| 1383247_a_at | Spns2 | | | Spinster homolog 2 (Drosophila) | | **Signalling/Trafficking** | | 568 | 431 | 0.95 | 0.73 | # |
| 1369718_at | Ssr3 | | | Signal sequence receptor, gamma | | **Signalling/Trafficking** | | 443 | 605 | 0.97 | 1.27 | # |
| 1381461_at, 1393183_at | Stard5 | | | StAR-related lipid transfer (START) domain containing | | **Signalling/Trafficking** | | 1056, 500 | 640, 361 | 1.11 | 1.45 | # |
| 1388679_at | Tbc1d14 | | | TBC1 domain family, member 14 | | **Signalling/Trafficking** | | 1089 | 836 | 0.89 | 0.71 | # |
| 1390434_at | Tradd | | | TNFRSF1A-associated via death domain | | **Signalling/Trafficking** | | 553 | 502 | 0.99 | 0.75 | # |
| 1376446_at | Visa | | | Virus-induced signaling adapter | | **Signalling/Trafficking** | | 386 | 530 | 1.04 | 0.77 | # |
| 1395289_at | Vps26b | | | Vacuolar protein sorting 26 homolog B (S. pombe) | | **Signalling/Trafficking** | | 502 | 488 | 0.97 | 0.73 | # |
| 1369402_at | Adnp | | | Activity-dependent neuroprotective protein (Protein-coding) | | **Transcriptional regulation** | | 236 | 390 | 0.88 | 1.36 | # |
| 1367624_at | Atf4 | | | Activating transcription factor 4 | | **Transcriptional regulation** | | 9396 | 5811 | 1.19 | 1.50 | # |
| 1372207_at | Brd8 | | | Bromodomain containing 8 | | **Transcriptional regulation** | | 278 | 494 | 0.92 | 0.68 | # |
| 1373885_at | Cbx5 | | | Chromobox homolog 5 (Drosophila HP1a) | | **Transcriptional regulation** | | 399 | 354 | 0.97 | 0.62 | # |
| 1370225_at | Cited4 | | | Cbp/p300-interacting transactivator, with Glu/Asp-rich carboxy-terminal domain, 4 | | **Transcriptional regulation** | | 979 | 994 | 0.98 | 0.71 | # |
| 1375666_at | Dmtf1 | | | Cyclin D binding myb-like transcription factor 1 | | **Transcriptional regulation** | | 947 | 626 | 1.04 | 1.28 | # |
| 1368851_at | Ets1 | | | v-ets erythroblastosis virus E26 oncogene homolog 1 (avian) | | **Transcriptional regulation** | | 1854 | 2042 | 1.13 | 1.41 | # |
| 1379244_at | Etv6 | | | ets variant gene 6 (TEL oncogene) | | **Transcriptional regulation** | | 483 | 351 | 0.90 | 1.27 | # |
| 1374125_at | Gata5 | | | GATA binding factor-5 | | **Transcriptional regulation** | | 426 | 538 | 0.93 | 0.75 | # |
| 1374335_at | Gata6 | | | GATA binding protein 6 | | **Transcriptional regulation** | | 2136 | 2997 | 0.94 | 0.73 | # |
| 1374451_at | Hand2 | | | Heart and neural crest derivatives expressed transcript 2 | | **Transcriptional regulation** | | 1171 | 1514 | 1.01 | 0.64 | # |
| 1387270_at | Hhex | | | Hematopoietically expressed homeobox | | **Transcriptional regulation** | | 446 | 315 | 0.85 | 0.65 | # |
| 1392981_at | Irx4 | | | Iroquois related homeobox 4 (Drosophila) | | **Transcriptional regulation** | | 2282 | 2277 | 0.83 | 0.68 | # |
| 1393138_at | Jund | | | Jun D proto-oncogene | | **Transcriptional regulation** | | 7106 | 7532 | 0.98 | 1.30 | # |
| 1385243_at | Maf | | | Avian musculoaponeurotic fibrosarcoma (v-maf) AS42 oncogene | | **Transcriptional regulation** | | 1208 | 1196 | 0.94 | 0.76 | # |
| 1381404_at | Mbd1 | | | Methyl-CpG binding domain protein 1 | | **Transcriptional regulation** | | 201 | 292 | 0.99 | 0.76 | # |
| 1393276_at | Med31 | | | Mediator complex subunit 31 | | **Transcriptional regulation** | | 1055 | 626 | 0.99 | 0.80 | # |
| 1384308_at | Meis1 | | | Meis homeobox 1 | | **Transcriptional regulation** | | 911 | 921 | 1.00 | 0.79 | # |
| 1391849_at | Mizf | | | MBD2-interacting zinc finger | | **Transcriptional regulation** | | 318 | 329 | 1.02 | 0.76 | # |
| 1373038_at | Mllt10 | | | Myeloid/lymphoid or mixed-lineage leukemia (trithorax homolog, Drosophila); translocated to, 10 | | **Transcriptional regulation** | | 352 | 572 | 1.03 | 0.79 | # |
| 1377124_at | Mtf1 | | | Metal response element binding transcription factor 1 | | **Transcriptional regulation** | | 587 | 326 | 1.09 | 1.39 | # |
| 1379982_at | Nrip1 | | | Nuclear receptor interacting protein 1 | | **Transcriptional regulation** | | 194 | 361 | 0.98 | 0.75 | # |
| 1393492_at | Pcgf5 | | | Polycomb group ring finger 5 | | **Transcriptional regulation** | | 360 | 374 | 1.00 | 0.78 | # |
| 1371856_at, 1397313_at | Pnrc2 | | | Proline-rich nuclear receptor coactivator 2 | | **Transcriptional regulation** | | 4569, 1207 | 4047, 1532 | 0.88 | 0.73 | # |
| 1388710_at | Rreb1 | | | Ras responsive element binding protein 1 | | **Transcriptional regulation** | | 228 | 583 | 0.99 | 0.72 | # |
| 1390364_at | Runx1 | | | Runt related transcription factor 1 | | **Transcriptional regulation** | | 322 | 304 | 0.94 | 1.61 | # |
| 1392269_at | Sin3a | | | Transcriptional regulator, SIN3A (yeast) | | **Transcriptional regulation** | | 281 | 365 | 0.91 | 0.74 | # |
| 1381175_at | Sltm | | | SAFB-like, transcription modulator | | **Transcriptional regulation** | | 71 | 277 | 1.21 | 0.74 | # |
| 1391630_at | Tbx18 | | | T-box 18 | | **Transcriptional regulation** | | 256 | 460 | 0.86 | 0.57 | # |
| 1377139_at | Tbx5 | | | T-box 5 | | **Transcriptional regulation** | | 479 | 514 | 0.86 | 0.69 | # |
| 1382288_at | Trip4 | | | Thyroid hormone receptor interactor 4 | | **Transcriptional regulation** | | 359 | 480 | 0.94 | 0.78 | # |
| 1380914_at, 1393652_at | Zbtb1 | | | Zinc finger and BTB domain containing 1 | | **Transcriptional regulation** | | 124, 250 | 271, 582 | 0.67 | 0.44 | # |
| 1385883_at | Zbtb11 | | | Zinc finger and BTB domain containing 11 | | **Transcriptional regulation** | | 295 | 769 | 0.84 | 0.69 | # |
| 1378009_at | Zbtb38 | | | Zinc finger and BTB domain containing 38 | | **Transcriptional regulation** | | 559 | 961 | 1.00 | 0.74 | # |
| 1390940_at | Zfhx1b | | | Zinc finger homeobox 1b | | **Transcriptional regulation** | | 167 | 234 | 1.09 | 1.34 | # |
| 1393120_at | Zfp251 | | | Zinc finger protein 251 | | **Transcriptional regulation** | | 664 | 579 | 0.84 | 0.65 | # |
| 1381902_at, 1399119_at | Zfp292 | | | Zinc finger protein 292 | | **Transcriptional regulation** | | 260, 187 | 487, 366 | 0.81 | 0.63 | # |
| 1398573_at | Zfp307 | | | Zinc finger protein 307 | | **Transcriptional regulation** | | 562 | 901 | 0.98 | 0.73 | # |
| 1368877_at | Zfp354a | | | Zinc finger protein 354A | | **Transcriptional regulation** | | 270 | 372 | 0.68 | 0.56 | # |
| 1379967_at | Zfp367 | | | Zinc finger protein 367 | | **Transcriptional regulation** | | 428 | 361 | 1.19 | 1.45 | # |
| 1368712_at | Zfp386 | | | Zinc finger protein 386 | | **Transcriptional regulation** | | 487 | 1263 | 0.96 | 0.80 | # |
| 1390148_a_at | Zfp395 | | | Zinc finger protein 395 | | **Transcriptional regulation** | | 1247 | 1056 | 0.84 | 0.70 | # |
| 1382242_at | Zfp451 | | | Zinc finger protein 451 | | **Transcriptional regulation** | | 218 | 340 | 0.96 | 0.76 | # |
| 1372802_at | Zfp512 | | | Zinc finger protein 512 | | **Transcriptional regulation** | | 590 | 574 | 0.94 | 0.73 | # |
| 1393572_at | Zfp592 | | | Zinc finger protein 592 | | **Transcriptional regulation** | | 230 | 304 | 1.00 | 0.69 | # |
| 1398377_at | Zfp672 | | | Zinc finger protein 672 | | **Transcriptional regulation** | | 484 | 440 | 1.14 | 1.43 | # |
| 1372751_at | Zfp740 | | | Zinc finger protein 740 | | **Transcriptional regulation** | | 356 | 344 | 1.00 | 0.74 | # |
| 1380794_at | Zfp770 | | | Zinc finger protein 770 | | **Transcriptional regulation** | | 465 | 522 | 0.88 | 0.72 | # |
| 1372872_at | Zzz3 | | | Zinc finger, ZZ domain containing 3 | | **Transcriptional regulation** | | 250 | 352 | 1.07 | 0.77 | # |
| 1392837_at | Aagab | | | Alpha- and gamma-adaptin binding protein | | **Unknown function** | | 719 | 554 | 1.01 | 0.73 | # |
| 1383964_at | Ankrd50 | | | Ankyrin repeat domain 50 | | **Unknown function** | | 443 | 779 | 1.01 | 0.79 | # |
| 1393051_at | Armcx1 | | | Armadillo repeat containing, X-linked 1 | | **Unknown function** | | 668 | 959 | 0.87 | 0.72 | # |
| 1372588_at | Bles03 | | | Basophilic leukemia expressed protein BLES03 | | **Unknown function** | | 1524 | 796 | 0.94 | 1.29 | # |
| 1380232_at | Cbwd1 | | | COBW domain containing 1 | | **Unknown function** | | 316 | 310 | 1.02 | 0.68 | # |
| 1382399_at | Ccdc55 | | | Coiled-coil domain containing 55 | | **Unknown function** | | 225 | 349 | 1.07 | 0.73 | # |
| 1374339_at | Ccdc90a | | | Coiled-coil domain containing 90A | | **Unknown function** | | 1071 | 943 | 1.08 | 0.76 | # |
| 1396317_at | Ccpg1 | | | Cell cycle progression 1 | | **Unknown function** | | 200 | 477 | 1.03 | 0.74 | # |
| 1379450_at, 1382873_at | Cttnbp2nl | | | CTTNBP2 N-terminal like | | **Unknown function** | | 939, 326 | 1100, 548 | 1.14 | 1.47 | # |
| 1374789_at | Dcun1d2 | | | DCN1, defective in cullin neddylation 1, domain containing 2 | | **Unknown function** | | 281 | 258 | 1.00 | 0.71 | # |
| 1398425_at | Fam110b | | | Family with sequence Similarity 110, member B | | **Unknown function** | | 1062 | 826 | 0.86 | 0.68 | # |
| 1388508_at | Fam32a | | | Family with sequence Similarity 32, member A | | **Unknown function** | | 1684 | 815 | 0.96 | 0.77 | # |
| 1388817_at | Fam63a | | | Family with sequence Similarity 63, member A | | **Unknown function** | | 827 | 804 | 0.96 | 0.76 | # |
| 1391218_at | Fam76b | | | Family with sequence Similarity 76, member B | | **Unknown function** | | 423 | 779 | 1.09 | 0.70 | # |
| 1384457_at | Fbxw17 | | | F-box and WD-40 domain protein 17 | | **Unknown function** | | 436 | 314 | 0.89 | 0.72 | # |
| 1383222_at | Frmd6 | | | FERM domain containing 6 | | **Unknown function** | | 992 | 984 | 1.10 | 1.48 | # |
| 1388395_at | G0s2 | | | G0/G1 switch gene 2 | | **Unknown function** | | 9021 | 6308 | 0.90 | 0.71 | # |
| 1378113_at | Gm22 | | | Gene model 22 | | **Unknown function** | | 299 | 282 | 1.05 | 1.53 | # |
| 1373773_at | Gpm6a | | | Glycoprotein m6a | | **Unknown function** | | 203 | 427 | 0.96 | 0.74 | # |
| 1381014_at | Ifi44 | | | Interferon-induced protein 44 | | **Unknown function** | | 372 | 433 | 1.05 | 0.75 | # |
| 1374614_at | Kbtbd4 | | | Kelch repeat and BTB (POZ) domain containing 4 | | **Unknown function** | | 387 | 486 | 0.96 | 0.74 | # |
| 1383359_at | Lnx2 | | | Ligand of numb-protein X 2 | | **Unknown function** | | 323 | 387 | 0.97 | 0.77 | # |
| 1371906_at | Lrig2 | | | leucine-rich repeats and immunoglobulin-like domains 2 | | **Unknown function** | | 388 | 597 | 0.85 | 0.68 | # |
| 1383342_at | Lrrc14 | | | Leucine-rich repeat-containing 14 | | **Unknown function** | | 378 | 337 | 1.03 | 0.77 | # |
| 1382941_at | Lrrc32 | | | Leucine rich repeat containing 32 | | **Unknown function** | | 818 | 497 | 0.93 | 0.74 | # |
| 1374296_at | Lrrc8a | | | Leucine-rich repeat-containing 8 family, member a | | **Unknown function** | | 3017 | 2396 | 1.02 | 1.28 | # |
| 1380357_a_at | Lrrc8c | | | Leucine rich repeat containing 8 family, member C | | **Unknown function** | | 690 | 808 | 1.03 | 0.76 | # |
| 1373431_at | Lrrc8d | | | Leucine rich repeat containing 8 family, member D | | **Unknown function** | | 699 | 604 | 0.93 | 0.74 | # |
| 1398804_at | Mak10 | | | MAK10 homolog, amino-acid N-acetyltransferase subunit | | **Unknown function** | | 509 | 680 | 0.99 | 0.61 | # |
| 1376718_at | Mblac1 | | | Metallo-beta-lactamase domain containing 1 | | **Unknown function** | | 733 | 458 | 0.85 | 0.62 | # |
| 1372420_at | Mpv17l2 | | | MPV17 mitochondrial membrane protein-like 2 | | **Unknown function** | | 1352 | 656 | 0.81 | 0.67 | # |
| 1391466_at | Nucks1 | | | Nuclear casein kinase and cyclin-dependent kinase substrate 1 | | **Unknown function** | | 473 | 343 | 1.08 | 0.73 | # |
| 1381088_at | Podn | | | Podocan | | **Unknown function** | | 599 | 553 | 0.96 | 0.73 | # |
| 1386653_at | Prickle2 | | | Prickle homolog 2 (Drosophila) | | **Unknown function** | | 663 | 592 | 0.98 | 0.76 | # |
| 1373132_at | Prr14 | | | Proline rich 14 | | **Unknown function** | | 725 | 646 | 1.02 | 0.72 | # |
| 1372108_at | Ptcd3 | | | Pentatricopeptide repeat domain 3 | | **Unknown function** | | 752 | 1583 | 0.97 | 0.79 | # |
| 1375849_at | Rgma | | | RGM domain family, member A | | **Unknown function** | | 1280 | 1201 | 0.90 | 0.72 | # |
| 1375393_at | Rnf214 | | | Ring finger protein 214 | | **Unknown function** | | 348 | 306 | 0.96 | 0.79 | # |
| 1373743_at | Rwdd2b | | | RWD domain containing 2B | | **Unknown function** | | 533 | 347 | 1.07 | 0.78 | # |
| 1392856_at | Serf1 | | | Small EDRK-rich factor 1 | | **Unknown function** | | 460 | 367 | 0.88 | 0.70 | # |
| 1389203_at | Sh3bp5l | | | SH3 binding domain protein 5 like | | **Unknown function** | | 1121 | 546 | 1.08 | 1.47 | # |
| 1375537_at | Strn3 | | | Striatin, calmodulin binding protein 3 | | **Unknown function** | | 2559 | 2080 | 1.13 | 1.37 | # |
| 1394842_at | Tmem19 | | | Transmembrane protein 19 | | **Unknown function** | | 605 | 621 | 1.05 | 0.74 | # |
| 1394160_at | Tmem2 | | | Transmembrane protein 2 | | **Unknown function** | | 350 | 656 | 1.13 | 1.40 | # |
| 1376610_a_at | Ttc28 | | | Tetratricopeptide repeat domain 28 | | **Unknown function** | | 495 | 676 | 0.95 | 0.73 | # |
| 1375858_at | Tusc4 | | | Tumor suppressor candidate 4 | | **Unknown function** | | 564 | 793 | 1.17 | 0.76 | # |
| 1377778_at | Vof16 | | | Ischemia related factor vof-16 | | **Unknown function** | | 80 | 327 | 1.04 | 2.92 | # |
| 1393955_at | Wdr44 | | | WD repeat domain 44 | | **Unknown function** | | 95 | 269 | 1.19 | 0.78 | # |
| 1379825_at | Wdr51b | | | WD repeat domain 51B | | **Unknown function** | | 490 | 346 | 1.09 | 0.77 | # |
| 1383239_at | Zcchc7 | | | Zinc finger, CCHC domain containing 7 | | **Unknown function** | | 524 | 921 | 1.03 | 0.78 | # |
| 1391558_at | Zfp608 | | | Zinc finger protein 608 | | **Unknown function** | | 124 | 189 | 0.92 | 1.36 | # |
| 1382362_at | Zfp87 | | | Zinc finger protein 87 | | **Unknown function** | | 195 | 376 | 0.75 | 0.60 | # |
| 1380186_at | Zmym5 | | | Zinc finger, MYM-type 5 | | **Unknown function** | | 222 | 366 | 0.89 | 0.58 | # |
| --- | --- | | | --- | | --- | | --- | --- | --- | --- |  |
| **No evidence for translational regulation (FDR<0.05 in polysomal and total RNAs OR ratio of P:T or T:P >1.2-fold)** | | | | | | | | --- | --- | --- | --- |  |
| 1387395_at | Adora2b | Adenosine A2B receptor | | | **Agonists/receptors** | | | 1239 | 808 | 0.73 | 0.74 | *# |
| 1383848_at | Adrb1 | Adrenergic receptor, beta 1 | | | **Agonists/receptors** | | | 640 | 718 | 0.57 | 0.40 | *# |
| 1390845_at | Agtrap | Angiotensin II, type I receptor-associated protein | | | **Agonists/receptors** | | | 817 | 544 | 0.94 | 0.80 | # |
| 1388924_at | Angptl4 | Angiopoietin-like 4 | | | **Agonists/receptors** | | | 666 | 574 | 0.73 | 0.76 | * |
| 1369871_at | Areg | Amphiregulin | | | **Agonists/receptors** | | | 94 | 73 | 10.13 | 11.55 | *# |
| 1370823_at | Bambi | BMP and activin membrane-bound inhibitor | | | **Agonists/receptors** | | | 1171 | 1061 | 0.69 | 0.52 | *# |
| 1368945_at, 1398270_at | Bmp2 | Bone morphogenetic protein 2 | | | **Agonists/receptors** | | | 113, 530 | 90, 491 | 2.31 | 2.33 | *# |
| 1388201_at | Bmp6 | Bone morphogenetic protein 6 | | | **Agonists/receptors** | | | 432 | 464 | 1.28 | 1.09 | * |
| 1367973_at | Ccl2 | Chemokine (C-C motif) ligand 2 | | | **Agonists/receptors** | | | 12079 | 13578 | 1.30 | 1.29 | *# |
| 1379935_at | Ccl7 | Chemokine (C-C motif) ligand 7 | | | **Agonists/receptors** | | | 4178 | 2730 | 1.69 | 1.61 | *# |
| 1369245_at | Chrm2 | Cholinergic receptor, muscarinic 2 | | | **Agonists/receptors** | | | 185 | 433 | 0.81 | 0.71 | # |
| 1390024_at | Clec2d/g | C-type lectin domain family 2, member d/g | | | **Agonists/receptors** | | | 1845 | 1901 | 0.64 | 0.62 | *# |
| 1367940_at | Cmkor1 | Chemokine orphan receptor 1 | | | **Agonists/receptors** | | | 1021 | 1041 | 1.45 | 1.30 | *# |
| 1367631_at | Ctgf | Connective tissue growth factor | | | **Agonists/receptors** | | | 10354 | 12903 | 1.78 | 1.83 | *# |
| 1387316_at | Cxcl1 | Chemokine (C-X-C motif) ligand 1 | | | **Agonists/receptors** | | | 4345 | 3361 | 1.44 | 1.53 | # |
| 1398390_at | Cxcl13 | Chemokine (C-X-C motif) ligand 13 | | | **Agonists/receptors** | | | 295 | 214 | 0.76 | 0.79 | * |
| 1368760_at | Cxcl2 | Chemokine (C-X-C motif) ligand 2 | | | **Agonists/receptors** | | | 584 | 471 | 1.32 | 1.11 | * |
| 1373661_a_at | Cxcr4 | Chemokine (C-X-C motif) receptor 4 | | | **Agonists/receptors** | | | 1400 | 1539 | 0.89 | 0.75 | # |
| 1371840_at | Edg1 | Endothelial differentiation sphingolipid G-protein-coupled receptor 1 | | | **Agonists/receptors** | | | 3750 | 4796 | 1.48 | 1.58 | *# |
| 1386989_at | Edg5 | Endothelial differentiation, sphingolipid G-protein-coupled receptor, 5 | | | **Agonists/receptors** | | | 2157 | 1428 | 0.79 | 0.76 | *# |
| 1389586_at | Ednrb | Endothelin receptor type B | | | **Agonists/receptors** | | | 888 | 1040 | 1.27 | 1.30 | *# |
| 1369587_at, 1385150_at | Ereg | Epiregulin | | | **Agonists/receptors** | | | 137, 157 | 160, 158 | 4.71 | 3.62 | *# |
| 1369182_at | F3 | Coagulation factor III | | | **Agonists/receptors** | | | 146 | 190 | 4.15 | 4.57 | *# |
| 1372750_at, 1387843_at | Fst | Follistatin | | | **Agonists/receptors** | | | 496, 467 | 496, 301 | 3.67 | 4.54 | *# |
| 1370256_at | Fzd1 | Frizzled homolog 1 (Drosophila) | | | **Agonists/receptors** | | | 5052 | 6645 | 0.89 | 0.79 | # |
| 1374530_at | Fzd7 | Frizzled homolog 7 (Drosophila) | | | **Agonists/receptors** | | | 255 | 377 | 0.68 | 0.67 | *# |
| 1377679_at | Gpr125 | G protein-coupled receptor 125 | | | **Agonists/receptors** | | | 340 | 367 | 0.95 | 0.79 | # |
| 1373158_at | Gpr146 | G protein-coupled receptor 146 | | | **Agonists/receptors** | | | 2291 | 2365 | 0.92 | 0.77 | # |
| 1382858_at | Gpr180 | G protein-coupled receptor 180 | | | **Agonists/receptors** | | | 714 | 446 | 0.88 | 0.74 | # |
| 1376828_at | Gprc5a | G protein-coupled receptor, family C, group 5, member A | | | **Agonists/receptors** | | | 341 | 443 | 1.44 | 1.59 | *# |
| 1368983_at | Hbegf | Heparin-binding EGF-like growth factor | | | **Agonists/receptors** | | | 393 | 381 | 2.65 | 3.04 | *# |
| 1370454_at, 1370997_at | Homer1 | Homer homolog 1 (Drosophila) | | | **Agonists/receptors** | | | 162, 11 | 210, 34 | 2.14 | 5.32 | *# |
| 1369124_at, 1369125_at | Htr2a | 5-hydroxytryptamine (serotonin) receptor 2A | | | **Agonists/receptors** | | | 163, 112 | 291, 305 | 1.56 | 1.42 | *# |
| 1387273_at | Il1rl1 | Interleukin 1 receptor-like 1 | | | **Agonists/receptors** | | | 997 | 1198 | 2.93 | 3.86 | *# |
| 1369191_at | Il6 | Interleukin 6 | | | **Agonists/receptors** | | | 132 | 131 | 8.93 | 10.48 | *# |
| 1369012_at, 1383486_at | Inhba | Inhibin beta-A | | | **Agonists/receptors** | | | 168, 310 | 223, 290 | 4.96 | 5.34 | *# |
| 1390177_at | Itpripl2 | Inositol 1,4,5-triphosphate receptor interacting protein-like 2 | | | **Agonists/receptors** | | | 1086 | 941 | 1.32 | 1.33 | * |
| 1376089_at | Ldlr | Low density lipoprotein receptor | | | **Agonists/receptors** | | | 1453 | 3814 | 1.43 | 1.35 | *# |
| 1388218_at | Ldlr | Low density lipoprotein receptor | | | **Agonists/receptors** | | | 115 | 533 | 1.53 | 1.50 | # |
| 1393728_at | Lif | Leukemia inhibitory factor | | | **Agonists/receptors** | | | 416 | 291 | 7.80 | 10.57 | *# |
| 1373178_at, 1383169_at | Lifr | leukemia inhibitory factor receptor | | | **Agonists/receptors** | | | 1031, 1135 | 1525, 1870 | 0.74 | 0.74 | *# |
| 1371259_at | Ngfb | Nerve growth factor, beta | | | **Agonists/receptors** | | | 230 | 217 | 1.20 | 1.39 | # |
| 1368883_at | Nov | Nephroblastoma overexpressed gene | | | **Agonists/receptors** | | | 583 | 710 | 1.10 | 1.32 | # |
| 1368683_at | Oldlr1 | Oxidized low density lipoprotein (lectin-like) receptor 1 | | | **Agonists/receptors** | | | 678 | 1316 | 1.49 | 1.85 | *# |
| 1368940_at | P2ry2 | Purinergic receptor P2Y, G-protein coupled 2 | | | **Agonists/receptors** | | | 1021 | 563 | 0.71 | 0.75 | *# |
| 1387269_s_at | Plaur | Plasminogen activator, urokinase receptor | | | **Agonists/receptors** | | | 482 | 372 | 2.53 | 3.63 | *# |
| 1393638_at | Ptger4 | Prostaglandin E receptor 4 (subtype EP4) | | | **Agonists/receptors** | | | 623 | 539 | 1.36 | 1.28 | * |
| 1370259_a_at | Pthr1 | Parathyroid hormone receptor 1 | | | **Agonists/receptors** | | | 1270 | 585 | 0.83 | 0.74 | # |
| 1370177_at | PVR | Poliovirus receptor | | | **Agonists/receptors** | | | 837 | 964 | 2.31 | 2.60 | *# |
| 1377404_at, 1386530_at, 1396101_at, 1393559_at | Stc1 | Stanniocalcin 1 | | | **Agonists/receptors** | | | 1274, 538, 1321, 527 | 1161, 627, 1849, 656 | 1.60 | 1.74 | *# |
| 1367859_at | Tgfb3 | Transforming growth factor, beta 3 | | | **Agonists/receptors** | | | 1338 | 927 | 1.39 | 1.83 | *# |
| 1375951_at | Thbd | Thrombomodulin | | | **Agonists/receptors** | | | 139 | 242 | 2.27 | 1.91 | *# |
| 1369407_at | Tnfrsf11b | Tumor necrosis factor receptor superfamily, member 11b (osteoprotegerin) | | | **Agonists/receptors** | | | 1003 | 1400 | 1.68 | 2.02 | *# |
| 1371785_at | Tnfrsf12a | Tumor necrosis factor receptor superfamily, member 12a | | | **Agonists/receptors** | | | 6412 | 3932 | 1.66 | 2.43 | *# |
| 1384842_s_at | Tnfrsf6 | Tumor necrosis factor receptor superfamily, member 6 | | | **Agonists/receptors** | | | 333 | 403 | 1.38 | 1.26 | *# |
| 1376472_at | Traf6 | Tnf receptor-associated factor 6 | | | **Agonists/receptors** | | | 315 | 428 | 0.90 | 0.79 | # |
| 1370081_a_at 1373807_at | Vegfa | Vascular endothelial growth factor A | | | **Agonists/receptors** | | | 798, 3205 | 1535, 5969 | 1.35 | 1.15 | * |
| 1386890_at | S100a10 | S100 calcium binding protein A10 (calpactin) | | | **Calcium regulation** | | | 7281 | 4535 | 1.22 | 1.28 | # |
| 1389546_at | Amotl2 | Angiomotin like 2 | | | **Cell-cell/matrix adhesion** | | | 2925 | 4116 | 1.11 | 1.26 | # |
| 1368921_a_at | Cd44 | CD44 antigen | | | **Cell-cell/matrix adhesion** | | | 795, 916, 1275 | 994, 1380, 2006 | 1.21 | 1.41 | # |
| 1368290_at | Cyr61 | Cysteine rich protein 61 | | | **Cell-cell/matrix adhesion** | | | 388 | 506 | 6.68 | 7.46 | *# |
| 1383353_at | Efnb2 | Ephrin B2 | | | **Cell-cell/matrix adhesion** | | | 615 | 537 | 1.55 | 1.63 | *# |
| 1374143_at | Epha2 | Eph receptor A2 | | | **Cell-cell/matrix adhesion** | | | 189 | 288 | 1.98 | 2.15 | *# |
| 1387202_at | Icam1 | Intercellular adhesion molecule 1 | | | **Cell-cell/matrix adhesion** | | | 1416 | 1620 | 1.96 | 2.87 | *# |
| 1385649_at | Itga5 | Integrin alpha 5 (mapped) | | | **Cell-cell/matrix adhesion** | | | 657 | 1302 | 1.51 | 1.63 | *# |
| 1386581_at | Itgav | Integrin alpha V (3' UTR close to protein coding region) | | | **Cell-cell/matrix adhesion** | | | 190 | 512 | 1.07 | 1.28 | # |
| 1383224_at | Pard6b | Par-6 (partitioning defective 6) homolog beta (C. elegans) | | | **Cell-cell/matrix adhesion** | | | 265 | 276 | 3.34 | 4.07 | *# |
| 1389919_at | Parvb | Parvin, beta | | | **Cell-cell/matrix adhesion** | | | 730 | 944 | 1.30 | 1.22 | * |
| 1377475_at | Pkp2 | Plakophilin 2 | | | **Cell-cell/matrix adhesion** | | | 238 | 424 | 1.25 | 1.26 | * |
| 1367849_at | Sdc1 | Syndecan 1 | | | **Cell-cell/matrix adhesion** | | | 1284 | 1316 | 1.17 | 1.27 | # |
| 1374529_at, 1394109_at | Thbs1 | Thrombospondin 1 | | | **Cell-cell/matrix adhesion** | | | 3225, 1042 | 10105, 5478 | 4.68 | 2.52 | *# |
| 1373401_at | Tnc | Tenascin C | | | **Cell-cell/matrix adhesion** | | | 245 | 1073 | 1.78 | 1.83 | *# |
| 1371194_at | Tnfaip6 | Tumor necrosis factor alpha induced protein 6 | | | **Cell-cell/matrix adhesion** | | | 392 | 458 | 4.47 | 4.96 | *# |
| 1398380_at | Vwa1 | von Willebrand factor A domain containing 1 | | | **Cell-cell/matrix adhesion** | | | 537 | 357 | 0.81 | 0.71 | # |
| 1389442_at | Zfp652 | Zinc finger protein 652 | | | **Cell-cell/matrix adhesion** | | | 637 | 628 | 0.89 | 0.77 | # |
| 1374539_at | Atp10d | ATPase, Class V, type 10D | | | **Channels/transporters** | | | 422 | 576 | 1.25 | 1.11 | * |
| 1373546_at | Atp11a | ATPase, class VI, type 11A | | | **Channels/transporters** | | | 536 | 506 | 0.83 | 0.78 | # |
| 1378134_at | Atp8b1 | ATPase, Class I, type 8B, member 1 | | | **Channels/transporters** | | | 229 | 418 | 1.46 | 1.29 | *# |
| 1389573_at | Chac1 | ChaC, cation transport regulator-like 1 (E. coli) | | | **Channels/transporters** | | | 1661 | 764 | 2.55 | 2.94 | *# |
| 1391270_at | Cnnm3 | Cyclin M3 | | | **Channels/transporters** | | | 675 | 735 | 0.82 | 0.73 | # |
| 1390969_at | Kcne4 | Potassium voltage-gated channel, Isk-related subfamily, gene 4 | | | **Channels/transporters** | | | 848 | 1056 | 0.71 | 0.51 | *# |
| 1368911_at | Kcnj8 | Potassium inwardly-rectifying channel,subfamily J, member 8 | | | **Channels/transporters** | | | 1213 | 855 | 0.84 | 0.75 | # |
| 1367853_at | Slc12a2 | Solute carrier family 12, member 2 | | | **Channels/transporters** | | | 523 | 968 | 1.38 | 1.35 | *# |
| 1370314_at | Slc20a1 | Solute carrier family 20 (phosphate transporter), member 1 | | | **Channels/transporters** | | | 1933 | 2847 | 1.65 | 1.58 | *# |
| 1371754_at | Slc25a25 | Solute carrier family 25 (mitochondrial carrier, phosphate carrier), member 25 | | | **Channels/transporters** | | | 493 | 295 | 2.99 | 4.06 | *# |
| 1382688_at | Slc25a37 | Solute carrier family 25, member 37 | | | **Channels/transporters** | | | 604 | 447 | 0.79 | 0.90 | * |
| 1379739_at | Slc4a7 | Solute carrier family 4, sodium bicarbonate cotransporter, member 7 | | | **Channels/transporters** | | | 335 | 817 | 1.20 | 1.39 | # |
| 1368391_at, 1368392_at | Slc7a1 | Solute carrier family 7 (cationic amino acid transporter, y+ system), member 1 | | | **Channels/transporters** | | | 778, 974 | 1298, 1348 | 1.47 | 1.48 | *# |
| 1378927_at | Slc7a2 | Solute carrier family 7 (cationic amino acid transporter, y+ system), member 2 | | | **Channels/transporters** | | | 730 | 1143 | 0.78 | 0.86 | * |
| 1385397_at | Steap4 | STEAP family member 4 | | | **Channels/transporters** | | | 869 | 1006 | 0.84 | 0.77 | # |
| 1377918_at | Stoml1 | Stomatin-like 1 | | | **Channels/transporters** | | | 340 | 302 | 0.89 | 0.76 | # |
| 1392531_at | Ttpal | Tocopherol (alpha) transfer protein-like | | | **Channels/transporters** | | | 539 | 395 | 1.42 | 1.26 | *# |
| 1386869_at | Actg2 | Actin, gamma 2 | | | **Cytoskeleton/myofibrillar** | | | 807 | 735 | 1.40 | 1.44 | *# |
| 1395886_at | Actr3 | ARP3 actin-related protein 3 homolog (yeast) | | | **Cytoskeleton/myofibrillar** | | | 583 | 1345 | 1.34 | 1.28 | *# |
| 1381504_at | Aspn | Asporin | | | **Cytoskeleton/myofibrillar** | | | 404 | 712 | 0.82 | 0.80 | # |
| 1393281_at | Cav1 | Caveolin 1 | | | **Cytoskeleton/myofibrillar** | | | 3179 | 3500 | 1.33 | 1.25 | *# |
| 1389119_at | Cmya1 | Xin actin-binding repeat containing 1 | | | **Cytoskeleton/myofibrillar** | | | 1081 | 2419 | 1.95 | 2.34 | *# |
| 1367785_at | Cnn1 | Calponin 1 | | | **Cytoskeleton/myofibrillar** | | | 790 | 875 | 1.25 | 1.32 | *# |
| 1370672_a_at | Dnm3 | Dynamin 3 | | | **Cytoskeleton/myofibrillar** | | | 233 | 226 | 0.77 | 0.75 | * |
| 1372569_at | Fhl3 | Four and a half LIM domains 3 | | | **Cytoskeleton/myofibrillar** | | | 2888 | 1808 | 1.10 | 1.29 | # |
| 1388496_at, 1396085_at | Flnc | Filamin C, gamma (actin binding protein 280) | | | **Cytoskeleton/myofibrillar** | | | 787, 382 | 2800, 1455 | 1.51 | 1.53 | *# |
| 1377072_at | Kank2 | KN motif and ankyrin repeat domains 2 | | | **Cytoskeleton/myofibrillar** | | | 1037 | 1083 | 0.79 | 0.87 | * |
| 1368252_at | Kbtbd10 | Kelch repeat and BTB (POZ) domain containing 10 | | | **Cytoskeleton/myofibrillar** | | | 484 | 1100 | 1.29 | 1.10 | * |
| 1376700_at | Lima1 | LIM domain and actin binding 1 | | | **Cytoskeleton/myofibrillar** | | | 1001 | 1490 | 1.41 | 1.36 | *# |
| 1368054_at, 1368055_a_at | Lmna | Lamin A | | | **Cytoskeleton/myofibrillar** | | | 727, 2136 | 881, 2521 | 1.33 | 1.50 | *# |
| 1377610_at | Lmod2 | Leiomodin 2 (cardiac) | | | **Cytoskeleton/myofibrillar** | | | 1380 | 2429 | 2.66 | 2.78 | *# |
| 1389727_at | Lrrc10 | Leucine-rich repeat-containing 10 | | | **Cytoskeleton/myofibrillar** | | | 1611 | 1318 | 0.76 | 0.67 | *# |
| 1388874_at | Mtss1 | Metastasis suppressor 1 | | | **Cytoskeleton/myofibrillar** | | | 1698 | 3072 | 0.79 | 0.82 | * |
| 1384232_at | Myo1b | Myosin IB | | | **Cytoskeleton/myofibrillar** | | | 400 | 972 | 0.91 | 0.77 | # |
| 1375857_at | Myof | Myoferlin | | | **Cytoskeleton/myofibrillar** | | | 693 | 1525 | 0.77 | 0.90 | * |
| 1367761_at | Ndel1 | NudE nuclear distribution gene E homolog like 1 (A. nidulans) | | | **Cytoskeleton/myofibrillar** | | | 1664 | 1239 | 1.27 | 1.31 | *# |
| 1374650_at, 1396053_at | Nedd9 | Neural precursor cell expressed, developmentally down-regulated gene 9 | | | **Cytoskeleton/myofibrillar** | | | 568, 226 | 883, 331 | 1.36 | 1.28 | *# |
| 1389918_at | Palld | Palladin | | | **Cytoskeleton/myofibrillar** | | | 770 | 1084 | 2.22 | 1.51 | *# |
| 1370347_at | Pdlim7 | PDZ and LIM domain 7 | | | **Cytoskeleton/myofibrillar** | | | 1575 | 903 | 1.12 | 1.26 | # |
| 1392105_at | Phactr2 | Phosphatase and actin regulator 2 | | | **Cytoskeleton/myofibrillar** | | | 516 | 552 | 0.88 | 0.77 | # |
| 1384182_at | Plekhc1 | Pleckstrin homology domain containing, family C (with FERM domain) member 1 | | | **Cytoskeleton/myofibrillar** | | | 396 | 639 | 1.27 | 1.24 | * |
| 1377595_at | Ssfa2 | Sperm specific antigen 2 | | | **Cytoskeleton/myofibrillar** | | | 1041 | 1520 | 1.29 | 1.24 | * |
| 1378508_at | Ssh1 | Slingshot homolog 1 (Drosophila) | | | **Cytoskeleton/myofibrillar** | | | 592 | 465 | 0.79 | 0.77 | * |
| 1388786_at, 1394907_at | Synpo | Synaptopodin | | | **Cytoskeleton/myofibrillar** | | | 2278, 397 | 2541, 613 | 0.65 | 0.69 | *# |
| 1371679_at, 1384160_at | Synpo2 | Synaptopodin-2 (Myopodin) | | | **Cytoskeleton/myofibrillar** | | | 2692, 801 | 4171, 1957 | 1.22 | 1.39 | # |
| 1372979_at | Synpo2l | Synaptopodin 2-like | | | **Cytoskeleton/myofibrillar** | | | 5825 | 4866 | 1.13 | 1.30 | # |
| 1367570_at | Tagln | Transgelin | | | **Cytoskeleton/myofibrillar** | | | 4355 | 2778 | 1.41 | 1.69 | *# |
| 1371554_at | Tcap | Titin-cap | | | **Cytoskeleton/myofibrillar** | | | 3190 | 1229 | 1.40 | 1.59 | *# |
| 1378311_at | Tln1 | Talin 1 | | | **Cytoskeleton/myofibrillar** | | | 2715 | 1893 | 1.07 | 1.26 | # |
| 1370287_a_at, 1395794_at | Tpm1 | Tropomyosin 1, alpha | | | **Cytoskeleton/myofibrillar** | | | 4050, 436 | 2268, 884 | 1.26 | 1.48 | *# |
| 1388795_at | Tppp | Tubulin polymerization promoting protein | | | **Cytoskeleton/myofibrillar** | | | 301 | 269 | 1.33 | 1.12 | * |
| 1375518_at | Ttn | Titin | | | **Cytoskeleton/myofibrillar** | | | 1323 | 4429 | 0.78 | 0.92 | * |
| 1376100_at | Tubb5/6 | Tubulin beta 5/6 | | | **Cytoskeleton/myofibrillar** | | | 4355 | 2958 | 1.25 | 1.39 | *# |
| 1372459_at | Vasp | Vasodilator-stimulated phosphoprotein | | | **Cytoskeleton/myofibrillar** | | | 3533 | 2563 | 1.20 | 1.29 | # |
| 1372905_at, 1398476_at | Vcl | Vinculin | | | **Cytoskeleton/myofibrillar** | | | 1645, 230 | 4753, 885 | 1.39 | 1.70 | *# |
| 1384101_at | Wasl | Wiskott-Aldrich syndrome-like | | | **Cytoskeleton/myofibrillar** | | | 572 | 670 | 1.29 | 1.30 | *# |
| 1389520_at | Wdr1 | WD repeat domain 1 | | | **Cytoskeleton/myofibrillar** | | | 3787 | 4525 | 1.13 | 1.28 | # |
| 1388130_at | Zyx | Zyxin | | | **Cytoskeleton/myofibrillar** | | | 5677 | 4942 | 1.43 | 1.73 | *# |
| 1392386_at | Aptx | Aprataxin | | | **DNA structure/repair** | | | 514 | 454 | 1.33 | 1.29 | *# |
| 1375307_at | Cbx6 | Chromobox homolog 6 | | | **DNA structure/repair** | | | 2355 | 2300 | 0.86 | 0.78 | # |
| 1399162_a_at | Ddb1 | Damage-specific DNA binding protein 1 | | | **DNA structure/repair** | | | 1556 | 2842 | 0.80 | 0.96 | * |
| 1388589_at | Dot1l | DOT1-like, histone H3 methyltransferase | | | **DNA structure/repair** | | | 534 | 602 | 2.05 | 2.78 | *# |
| 1388309_at | Hmga1 | High mobility group AT-hook 1 | | | **DNA structure/repair** | | | 3908 | 2357 | 1.26 | 1.43 | *# |
| 1399118_at | Msl1 | Male-specific lethal 1 homolog (Drosophila) | | | **DNA structure/repair** | | | 675 | 422 | 0.79 | 0.88 | * |
| 1371684_at | Pelo | Pelota homolog | | | **DNA structure/repair** | | | 2478 | 1830 | 1.30 | 1.49 | *# |
| 1384523_at | Pms1 | Postmeiotic segregation increased 1 | | | **DNA structure/repair** | | | 222 | 323 | 0.79 | 0.71 | # |
| 1374424_at | Prmt5 | Protein arginine N-methyltransferase 5 | | | **DNA structure/repair** | | | 675 | 711 | 0.78 | 0.89 | * |
| 1376401_at | Repin1 | Replication initiator 1 | | | **DNA structure/repair** | | | 797 | 686 | 0.79 | 0.89 | * |
| 1374919_at | Rfc1 | Replication factor C (activator 1) 1 | | | **DNA structure/repair** | | | 361 | 479 | 0.90 | 0.78 | # |
| 1374603_at | Setmar | SET domain and mariner transposase fusion gene | | | **DNA structure/repair** | | | 825 | 679 | 0.76 | 0.77 | *# |
| 1388780_at | Terf2ip | Telomeric repeat binding factor 2, interacting protein | | | **DNA structure/repair** | | | 798 | 853 | 0.83 | 0.73 | # |
| 1383282_at | Thap11 | THAP domain containing 11 | | | **DNA structure/repair** | | | 895 | 584 | 0.74 | 0.64 | *# |
| 1390928_at | Tigd2 | Tigger transposable element derived 2 | | | **DNA structure/repair** | | | 314 | 263 | 0.73 | 0.54 | * |
| 1391217_at | Zc3h12c | Zinc finger CCCH-type containing 12C | | | **DNA structure/repair** | | | 227 | 295 | 1.32 | 1.44 | # |
| 1394095_at | Zh2c2 | Zinc finger, H2C2 domain containing | | | **DNA structure/repair** | | | 192 | 334 | 0.79 | 0.72 | # |
| 1369955_at | Col5a1 | Collagen, type V, alpha 1 | | | **Extracellular matrix** | | | 1230 | 4230 | 0.77 | 0.71 | * |
| 1389533_at | Fbln2 | fibulin 2 | | | **Extracellular matrix** | | | 817 | 1501 | 0.80 | 0.94 | * |
| 1370623_at, 1383516_at, 1386637_at, 1392894_at | Fgl2 | Fibrinogen-like 2 | | | **Extracellular matrix** | | | 288, 277, 243, 542 | 451, 315, 268, 624 | 2.04 | 2.19 | *# |
| 1379340_at | Lamc2 | Laminin, gamma 2 | | | **Extracellular matrix** | | | 550 | 1171 | 1.33 | 1.13 | * |
| 1379526_at | Mbp | Myelin basic protein | | | **Extracellular matrix** | | | 1028 | 536 | 0.89 | 0.75 | # |
| 1371420_at | LOC494529 | 92Aa-Protein | | | **Hypothetical proteins** | | | 651 | 672 | 0.83 | 0.78 | # |
| 1389709_at | LOC499900 | Similar to Zinc finger protein 133 | | | **Hypothetical proteins** | | | 417 | 361 | 0.76 | 0.73 | *# |
| 1392057_at | LOC500893 | Similar to GLI-Kruppel family member GLI4 | | | **Hypothetical proteins** | | | 473 | 431 | 0.66 | 0.64 | *# |
| 1373830_at, 1373831_at | LOC619574 | Hypothetical protein LOC619574 | | | **Hypothetical proteins** | | | 528, 474 | 534, 439 | 0.81 | 0.75 | # |
| 1375988_at | LOC680262 | Hypothetical protein LOC680262 | | | **Hypothetical proteins** | | | 310 | 261 | 0.67 | 0.63 | *# |
| 1389196_at | LOC681367 | Hypothetical protein LOC681367 | | | **Hypothetical proteins** | | | 1787 | 1069 | 0.85 | 0.78 | # |
| 1379057_at | LOC683460 | Hypothetical protein LOC683460 | | | **Hypothetical proteins** | | | 278 | 192 | 0.75 | 0.87 | * |
| 1383405_at | LOC685398 | Similar to 1A6/DRIM (down-regulated in metastasis) interacting protein | | | **Hypothetical proteins** | | | 408 | 458 | 0.86 | 0.73 | # |
| 1377523_at | LOC686314 | Similar to dachshund b | | | **Hypothetical proteins** | | | 258 | 268 | 0.74 | 0.71 | * |
| 1373172_at | LOC687356 | hypothetical protein LOC687356 | | | **Hypothetical proteins** | | | 1121 | 591 | 0.78 | 0.70 | *# |
| 1372997_at | LOC687713 | Similar to Protein C22orf13 homolog | | | **Hypothetical proteins** | | | 1561 | 939 | 0.81 | 0.74 | # |
| 1373659_at | LOC688257/  LOC689926 | Hypothetical protein LOC688257/LOC689926 | | | **Hypothetical proteins** | | | 822 | 579 | 0.73 | 0.64 | * |
| 1383227_at | LOC688311 | Similar to ADP-ribosylation factor-like 1 | | | **Hypothetical proteins** | | | 1582 | 898 | 0.80 | 0.77 | # |
| 1389365_at | LOC690000 | Similar to CG3740-PA | | | **Hypothetical proteins** | | | 974 | 836 | 0.80 | 0.69 | *# |
| 1375884_at | MGC105560 | Similar to Hypothetical protein BC014729 | | | **Hypothetical proteins** | | | 1214 | 1378 | 0.88 | 0.74 | # |
| 1379343_at | MGC94335 | Similar to hypothetical protein FLJ22555 | | | **Hypothetical proteins** | | | 223 | 313 | 0.86 | 0.79 | # |
| 1385208_at | RGD1305014 | Similar to RIKEN cDNA 2310057M21 | | | **Hypothetical proteins** | | | 310 | 349 | 1.25 | 1.25 | # |
| 1399107_at | RGD1305158 | Similar to RIKEN cDNA 1810030N24 | | | **Hypothetical proteins** | | | 498 | 491 | 0.94 | 0.80 | # |
| 1393027_at | RGD1305235 | Similar to RIKEN cDNA 1700052N19 | | | **Hypothetical proteins** | | | 607 | 677 | 0.91 | 0.76 | # |
| 1380619_at | RGD1305537 | Similar to RIKEN cDNA 3110001I22 | | | **Hypothetical proteins** | | | 202 | 239 | 1.47 | 1.38 | *# |
| 1391461_at | RGD1306576 | Similar to hypothetical protein | | | **Hypothetical proteins** | | | 847 | 471 | 0.82 | 0.79 | # |
| 1373982_at | RGD1306595 | Similar to hypothetical protein | | | **Hypothetical proteins** | | | 474 | 451 | 0.85 | 0.77 | # |
| 1373321_at | RGD1306622 | Similar to KIAA0954 protein | | | **Hypothetical proteins** | | | 416 | 248 | 0.76 | 0.79 | * |
| 1376645_at | RGD1307396 | Similar to RIKEN cDNA 6330406I15 | | | **Hypothetical proteins** | | | 605 | 529 | 0.74 | 0.84 | * |
| 1398971_at | RGD1307929 | Similar to CG14967-PA | | | **Hypothetical proteins** | | | 3386 | 2727 | 0.87 | 0.80 | # |
| 1373226_at | RGD1308019 | Similar to hypothetical protein FLJ20245 | | | **Hypothetical proteins** | | | 446 | 294 | 0.66 | 0.72 | *# |
| 1372396_at | RGD1308026 | Similar to 2310047B19Rik protein | | | **Hypothetical proteins** | | | 731 | 346 | 0.80 | 0.90 | * |
| 1374554_at | RGD1308048 | Similar to HT014 | | | **Hypothetical proteins** | | | 3560 | 1809 | 0.88 | 0.79 | # |
| 1374067_at, 1389298_at | RGD1308127 | Similar to 2700078E11Rik protein | | | **Hypothetical proteins** | | | 1767, 757 | 1806, 698 | 1.22 | 1.29 | # |
| 1372184_at | RGD1309188 | Similar to hypothetical protein BC011833 | | | **Hypothetical proteins** | | | 1618 | 1009 | 0.77 | 0.74 | *# |
| 1383508_at | RGD1309403 | Similar to hypothetical protein FLJ12661 | | | **Hypothetical proteins** | | | 242 | 450 | 0.82 | 0.77 | # |
| 1372095_at | RGD1309821 | Similar to KIAA1161 protein | | | **Hypothetical proteins** | | | 226 | 418 | 0.90 | 0.76 | # |
| 1373596_at | RGD1310423 | Similar to hypothetical protein FLJ31737 | | | **Hypothetical proteins** | | | 921 | 528 | 0.87 | 0.74 | # |
| 1374169_at | RGD1310686 | Similar to chromosome 16 open reading frame 5 | | | **Hypothetical proteins** | | | 1073 | 812 | 0.75 | 0.71 | *# |
| 1388945_at | RGD1311307 | Similar to 1300014I06Rik protein | | | **Hypothetical proteins** | | | 809 | 511 | 1.70 | 2.03 | *# |
| 1390699_at | RGD1311595 | Similar to KIAA2026 protein | | | **Hypothetical proteins** | | | 315 | 373 | 0.88 | 0.76 | # |
| 1397579_x_at | RGD1359127 | RGD1359127 Similar to RIKEN cDNA 2310011J03 | | | **Hypothetical proteins** | | | 346 | 571 | 0.66 | 0.67 | # |
| 1398364_at | RGD1359529 | Similar to chromosome 1 open reading frame 63 | | | **Hypothetical proteins** | | | 429 | 2454 | 0.72 | 0.75 | *# |
| 1398513_at | RGD1559971 | Similar to Na+ dependent glucose transporter 1 | | | **Hypothetical proteins** | | | 234 | 342 | 0.76 | 0.76 | # |
| 1374321_at | RGD1560108 | Similar to RIKEN cDNA 2700081O15 | | | **Hypothetical proteins** | | | 678 | 613 | 1.28 | 1.15 | * |
| 1383001_at, 1383874_at, 1394278_at | RGD1560812 | Hypothetical protein RGD1560812 | | | **Hypothetical proteins** | | | 307, 461, 273 | 252, 397, 251 | 4.82 | 3.78 | *# |
| 1381923_at | RGD1564664 | Similar to LOC387763 protein | | | **Hypothetical proteins** | | | 278 | 277 | 2.74 | 1.69 | *# |
| 1395064_at | RGD1564943 | Similar to 4930429A08Rik protein | | | **Hypothetical proteins** | | | 623 | 492 | 0.70 | 0.76 | *# |
| 1376927_at | RGD1564978 | Similar to LOC432779 protein | | | **Hypothetical proteins** | | | 239 | 356 | 0.84 | 0.72 | # |
| 1376937_at | RGD1565927 | Similar to 4631422O05Rik protein | | | **Hypothetical proteins** | | | 691 | 1035 | 1.81 | 1.78 | *# |
| 1383791_at | RGD1566155 | Similar to 2610030H06Rik protein | | | **Hypothetical proteins** | | | 241 | 233 | 1.25 | 1.26 | # |
| 1372871_at | RGD735175 | Hypothetical protein MGC:72616 | | | **Hypothetical proteins** | | | 1191 | 950 | 0.76 | 0.82 | * |
| 1373338_at | Unknown | Unknown | | | **No established gene** | | | 832 | 475 | 0.92 | 0.77 | # |
| 1373679_at | Unknown | Unknown | | | **No established gene** | | | 420 | 664 | 2.08 | 1.96 | *# |
| 1374060_at | Unknown | Unknown | | | **No established gene** | | | 1005 | 1314 | 0.73 | 0.75 | *# |
| 1374246_at | Unknown | Unknown | | | **No established gene** | | | 597 | 662 | 0.76 | 0.78 | *# |
| 1374710_at | Unknown | Unknown | | | **No established gene** | | | 1746 | 1841 | 1.26 | 1.35 | *# |
| 1374771_at | Unknown | Unknown | | | **No established gene** | | | 735 | 742 | 0.87 | 0.80 | # |
| 1374932_at | Unknown | Unknown | | | **No established gene** | | | 476 | 258 | 0.46 | 0.41 | * |
| 1376824_at | Unknown | Unknown | | | **No established gene** | | | 284 | 307 | 0.91 | 0.80 | # |
| 1376990_at | Unknown | Unknown | | | **No established gene** | | | 504 | 604 | 0.79 | 0.82 | * |
| 1378070_at | Unknown | Unknown | | | **No established gene** | | | 750 | 863 | 0.78 | 0.77 | *# |
| 1378098_at | Unknown | Unknown | | | **No established gene** | | | 1105 | 1219 | 1.29 | 1.08 | * |
| 1378117_at | Unknown | Unknown | | | **No established gene** | | | 246 | 310 | 0.65 | 0.62 | *# |
| 1378183_at | Unknown | Unknown | | | **No established gene** | | | 471 | 540 | 1.26 | 1.40 | # |
| 1378241_at | Unknown | Unknown | | | **No established gene** | | | 634 | 421 | 0.74 | 0.86 | * |
| 1378620_at | Unknown | Unknown | | | **No established gene** | | | 308 | 393 | 0.87 | 0.78 | # |
| 1378649_at | Unknown | Unknown | | | **No established gene** | | | 179 | 326 | 0.69 | 0.77 | # |
| 1378754_at | Unknown | Unknown | | | **No established gene** | | | 608 | 359 | 2.15 | 2.66 | *# |
| 1378808_at | Unknown | Unknown | | | **No established gene** | | | 239 | 472 | 0.80 | 0.68 | # |
| 1379268_at | Unknown | Unknown | | | **No established gene** | | | 1148 | 616 | 0.78 | 0.79 | # |
| 1379506_at | Unknown | Unknown | | | **No established gene** | | | 373 | 418 | 0.79 | 0.85 | * |
| 1379584_at | Unknown | Unknown | | | **No established gene** | | | 180 | 215 | 2.12 | 2.08 | *# |
| 1379605_at | Unknown | Unknown | | | **No established gene** | | | 187 | 442 | 0.71 | 0.77 | # |
| 1379617_at | Unknown | Unknown | | | **No established gene** | | | 404 | 465 | 0.91 | 0.79 | # |
| 1379714_at | Unknown | Unknown | | | **No established gene** | | | 126 | 134 | 2.18 | 2.44 | *# |
| 1379882_a_at | Unknown | Unknown | | | **No established gene** | | | 296 | 642 | 0.86 | 0.77 | # |
| 1381341_at | Unknown | Unknown | | | **No established gene** | | | 307 | 180 | 3.18 | 4.93 | *# |
| 1381826_at | Unknown | Unknown | | | **No established gene** | | | 874 | 442 | 0.87 | 0.76 | # |
| 1382228_at | Unknown | Unknown | | | **No established gene** | | | 325 | 495 | 0.60 | 0.55 | *# |
| 1382705_at | Unknown | Unknown | | | **No established gene** | | | 276 | 314 | 0.82 | 0.78 | # |
| 1383058_at | Unknown | Unknown | | | **No established gene** | | | 4105 | 2326 | 0.46 | 0.55 | *# |
| 1383188_at | Unknown | Unknown | | | **No established gene** | | | 242 | 262 | 0.76 | 0.59 | * |
| 1383393_at | Unknown | Unknown | | | **No established gene** | | | 814 | 978 | 0.84 | 0.79 | # |
| 1383688_at | Unknown | Unknown | | | **No established gene** | | | 676 | 619 | 0.86 | 0.74 | # |
| 1383744_at | Unknown | Unknown | | | **No established gene** | | | 1173 | 1516 | 1.20 | 1.32 | # |
| 1383919_at | Unknown | Unknown | | | **No established gene** | | | 251 | 171 | 0.67 | 0.79 | * |
| 1384401_at | Unknown | Unknown | | | **No established gene** | | | 410 | 522 | 0.63 | 0.63 | *# |
| 1384562_at | Unknown | Unknown | | | **No established gene** | | | 498 | 410 | 1.50 | 1.36 | *# |
| 1384582_at | Unknown | Unknown | | | **No established gene** | | | 531 | 502 | 1.26 | 1.09 | * |
| 1385594_at | Unknown | Unknown | | | **No established gene** | | | 799 | 766 | 0.87 | 0.78 | # |
| 1386633_at | Unknown | Unknown | | | **No established gene** | | | 167 | 299 | 0.71 | 0.62 | # |
| 1389243_at | Unknown | Unknown | | | **No established gene** | | | 527 | 461 | 0.77 | 0.73 | *# |
| 1389618_at | Unknown | Unknown | | | **No established gene** | | | 1142 | 1395 | 0.77 | 0.69 | *# |
| 1390109_at | Unknown | Unknown | | | **No established gene** | | | 1038 | 1417 | 0.81 | 0.70 | # |
| 1390503_at | Unknown | Unknown | | | **No established gene** | | | 214 | 202 | 1.37 | 1.42 | * |
| 1390615_at | Unknown | Unknown | | | **No established gene** | | | 169 | 229 | 1.25 | 1.28 | # |
| 1391154_at | Unknown | Unknown | | | **No established gene** | | | 390 | 395 | 0.79 | 0.74 | *# |
| 1391744_at | Unknown | Unknown | | | **No established gene** | | | 182 | 391 | 0.80 | 0.75 | # |
| 1391939_at | Unknown | Unknown | | | **No established gene** | | | 331 | 410 | 1.31 | 1.18 | * |
| 1392087_at | Unknown | Unknown | | | **No established gene** | | | 196 | 322 | 0.91 | 0.79 | # |
| 1392702_at | Unknown | Unknown | | | **No established gene** | | | 328 | 261 | 0.80 | 0.77 | * |
| 1392992_at | Unknown | Unknown | | | **No established gene** | | | 345 | 366 | 0.79 | 0.89 | * |
| 1393019_at | Unknown | Unknown | | | **No established gene** | | | 245 | 370 | 0.87 | 0.73 | # |
| 1393122_at | Unknown | Unknown | | | **No established gene** | | | 677 | 316 | 0.72 | 0.72 | * |
| 1393360_at | Unknown | Unknown | | | **No established gene** | | | 955 | 1475 | 0.78 | 0.68 | *# |
| 1395119_at | Unknown | Unknown | | | **No established gene** | | | 224 | 221 | 2.36 | 2.06 | *# |
| 1396363_at | Unknown | Unknown | | | **No established gene** | | | 325 | 176 | 0.74 | 0.70 | * |
| 1397897_x_at | Unknown | Unknown | | | **No established gene** | | | 471 | 542 | 0.79 | 0.90 | * |
| 1398366_at | Unknown | Unknown | | | **No established gene** | | | 331 | 422 | 0.77 | 0.64 | *# |
| 1382778_at | AS:Dusp6 | AS:Dual specificity phosphatase 6 | | | **Non-protein coding** | | | 801 | 685 | 2.03 | 2.46 | *# |
| 1383905_at | AS:Foxc1 | AS:Forkhead box C1 | | | **Non-protein coding** | | | 461 | 504 | 1.61 | 1.40 | *# |
| 1389905_at | AS:Gas1 | AS:growth arrest specific 1 | | | **Non-protein coding** | | | 3160 | 2765 | 0.83 | 0.75 | # |
| 1375183_at | AS:Id4 | AS:Inhibitor of DNA binding 4 | | | **Non-protein coding** | | | 334 | 220 | 1.84 | 1.57 | * |
| 1385961_at | AS:Klf5 | AS:Kruppel-like factor 5 | | | **Non-protein coding** | | | 120 | 206 | 4.07 | 3.18 | *# |
| 1372183_at | AS:Kpna1 | AS:Karyopherin (importin) alpha 1 | | | **Non-protein coding** | | | 1209 | 2134 | 1.20 | 1.29 | # |
| 1375676_at | AS:Lin7c | AS:Lin-7 homolog C (C. elegans) | | | **Non-protein coding** | | | 373 | 370 | 1.59 | 1.60 | *# |
| 1374429_at | AS:Pim1 | AS:Pim-1 kinase | | | **Non-protein coding** | | | 1566 | 1425 | 2.29 | 2.01 | *# |
| 1374324_at | AS:Ptger1 | AS:prostaglandin E receptor 1 (subtype EP1) | | | **Non-protein coding** | | | 433 | 548 | 0.87 | 0.74 | # |
| 1382171_at | AS:Tsc22d2 | AS:TSC22 domain family protein 2 | | | **Non-protein coding** | | | 828 | 1040 | 2.64 | 2.98 | *# |
| 1375538_at | AS:Vcl | AS:Vinculin | | | **Non-protein coding** | | | 2576 | 6175 | 1.37 | 1.49 | *# |
| 1375850_at | Intron:Bat1a | Intron:HLA-B-associated transcript 1A | | | **Non-protein coding** | | | 347 | 820 | 1.15 | 1.25 | # |
| 1397449_at | Intron:Enah | Intron:Enabled homolog | | | **Non-protein coding** | | | 53 | 256 | 2.05 | 2.16 | # |
| 1382942_at | Intron:Ext1 | Intron:Exostoses (multiple) 1 | | | **Non-protein coding** | | | 30 | 223 | 1.96 | 1.73 | # |
| 1381343_at | Intron:Frmd6 | Intron:FERM domain containing 6 | | | **Non-protein coding** | | | 113 | 211 | 1.29 | 1.52 | # |
| 1374166_at | Intron:Grtp1 | Intron:GH regulated TBC protein 1 | | | **Non-protein coding** | | | 693 | 1327 | 1.62 | 1.47 | *# |
| 1395172_at | Intron:Map1b | Intron:Microtubule-associated protein 1b | | | **Non-protein coding** | | | 123 | 220 | 1.31 | 1.40 | # |
| 1393540_at | Intron:Myh6 | Intron:Myosin heavy chain, polypeptide 6 | | | **Non-protein coding** | | | 290 | 353 | 0.65 | 0.80 | # |
| 1382631_at | Intron: | Intron:Similar to liver-specific bHLH-Zip transcription factor | | | **Non-protein coding** | | | 300 | 327 | 1.15 | 1.35 | # |
| 1383659_a_at | Intron: | Intron:Similar to Nuclear protein SkiP (Ski-interacting protein) (SNW1 protein) | | | **Non-protein coding** | | | 1205 | 558 | 0.84 | 0.71 | # |
| 1378081_at | Intron: | Intron:Similar to Transforming growth factor beta 1 induced transcript 4, isoform 1 | | | **Non-protein coding** | | | 115 | 232 | 1.30 | 1.30 | # |
| 1382738_at | Intron:Ssrb | Intron:Signal sequence receptor, beta | | | **Non-protein coding** | | | 189 | 240 | 1.15 | 1.27 | # |
| 1378447_at | Intron:Thrap1 | Intron:Thyroid hormone receptor associated protein 1 | | | **Non-protein coding** | | | 95 | 176 | 3.17 | 3.04 | *# |
| 1374767_at | Mirna23b/27b/24-1 cluster | MicroRNA 23b/27b/24-1 cluster | | | **Non-protein coding** | | | 946 | 967 | 1.30 | 1.46 | *# |
| 1371595_at, 1397164_at | Neat1 | Nuclear paraspeckle assembly transcript 1 (non-protein coding) | | | **Non-protein coding** | | | 365, 123 | 1756, 662 | 1.89 | 2.42 | *# |
| 1397220_at | Snhg7 | Small nucleolar RNA host gene (non-protein coding) 7 | | | **Non-protein coding** | | | 225 | 386 | 0.78 | 0.69 | # |
| 1377022_at | Abhd10 | Abhydrolase domain containing 10 | | | **Protein processing** | | | 467 | 452 | 1.33 | 1.21 | * |
| 1368223_at | Adamts1 | A disintegrin-like and metallopeptidse (reprolysin type) with thrombospondin type 1 motif, 1 | | | **Protein processing** | | | 645 | 1415 | 2.21 | 2.52 | *# |
| 1390931_at | Adamts15 | A disintegrin-like and metallopeptidase (reprolysin type) with thrombospondin type 1 motif, 15 | | | **Protein processing** | | | 547 | 898 | 0.60 | 0.55 | *# |
| 1389087_at | Anapc2 | Anaphase promoting complex subunit 2 | | | **Protein processing** | | | 1285 | 1654 | 0.80 | 0.87 | * |
| 1371073_at | B4galt1 | UDP-Gal:betaGlcNAc beta 1,4- galactosyltransferase, polypeptide 1 | | | **Protein processing** | | | 967 | 944 | 1.10 | 1.26 | # |
| 1368860_at | Dnaja1 | DnaJ (Hsp40) homolog, subfamily A, member 1 | | | **Protein processing** | | | 221 | 181 | 7.27 | 7.10 | *# |
| 1383302_at, 1388722_at | Dnajb1 | DnaJ (Hsp40) homolog, subfamily B, member 1 | | | **Protein processing** | | | 1327, 1849 | 1034, 1359 | 1.37 | 1.46 | *# |
| 1372722_at | Dnajb4 | DnaJ (Hsp40) homolog, subfamily B, member 4 | | | **Protein processing** | | | 1279 | 1516 | 2.18 | 2.35 | *# |
| 1372189_at | Dnajc13 | DnaJ (Hsp40) homolog, subfamily C, member 13 | | | **Protein processing** | | | 584 | 1158 | 0.78 | 0.88 | * |
| 1369813_at | Dnajc5 | DnaJ (Hsp40) homolog, subfamily C, member 5 | | | **Protein processing** | | | 271 | 296 | 1.19 | 1.41 | # |
| 1382059_at, 1392747_at | Fbxo30 | F-box only protein 30 | | | **Protein processing** | | | 289, 201 | 631, 395 | 1.94 | 1.70 | *# |
| 1372273_at | Gypc | Glycophorin C (Gerbich blood group) | | | **Protein processing** | | | 3668 | 2537 | 0.78 | 0.71 | *# |
| 1367741_at | Herpud1 | Homocysteine-inducible, endoplasmic reticulum stress-inducible, ubiquitin-like domain member 1 | | | **Protein processing** | | | 1857 | 1584 | 1.29 | 1.14 | * |
| 1368247_at | Hspa1a/1b | Heat shock 70kD protein 1A/1B | | | **Protein processing** | | | 266 | 317 | 3.38 | 3.76 | *# |
| 1370912_at | Hspa1b | Heat shock 70kD protein 1B | | | **Protein processing** | | | 321 | 443 | 2.91 | 2.63 | *# |
| 1367577_at | Hspb1 | Heat shock 27kDa protein 1 | | | **Protein processing** | | | 10947 | 8396 | 1.09 | 1.26 | # |
| 1367478_at | Htra2 | HtrA serine peptidase 2 | | | **Protein processing** | | | 1974 | 1441 | 0.91 | 0.80 | # |
| 1376579_at | Lap3 | Leucine aminopeptidase 3 | | | **Protein processing** | | | 949 | 986 | 0.91 | 0.78 | # |
| 1383288_at, 1383485_at, 1384427_at | Mdm2 | Transformed mouse 3T3 cell double minute 2 | | | **Protein processing** | | | 1495, 184, 668 | 1737, 361, 743 | 1.85 | 1.81 | *# |
| 1367858_at | Mmp11 | Matrix metallopeptidase 11 | | | **Protein processing** | | | 666 | 537 | 0.79 | 0.71 | *# |
| 1389474_at | Mylip | Myosin regulatory light chain interacting protein | | | **Protein processing** | | | 377 | 318 | 0.78 | 0.68 | * |
| 1388758_at | Ogt | O-linked N-acetylglucosamine (GlcNAc) transferase | | | **Protein processing** | | | 310 | 1235 | 0.58 | 0.75 | *# |
| 1376056_at | Parp10 | Poly (ADP-ribose) polymerase family, member 10 | | | **Protein processing** | | | 379 | 446 | 0.86 | 0.78 | # |
| 1374693_at, 1374694_at | Parp16 | Poly (ADP-ribose) polymerase family, member 16 | | | **Protein processing** | | | 563, 482 | 413, 710 | 0.69 | 0.62 | *# |
| 1375215_x_at | Pgpep1 | Pyroglutamyl-peptidase I | | | **Protein processing** | | | 4379 | 2518 | 0.92 | 0.79 | # |
| 1398352_at | Pias4 | Protein inhibitor of activated STAT, 4 | | | **Protein processing** | | | 392 | 422 | 0.87 | 0.77 | # |
| 1387781_at | Pmpcb | Peptidase (mitochondrial processing) beta | | | **Protein processing** | | | 983 | 2016 | 0.79 | 0.91 | * |
| 1372517_at | Ppil1 | Peptidylprolyl isomerase (cyclophilin)-like 1 | | | **Protein processing** | | | 836 | 421 | 0.89 | 0.78 | # |
| 1396171_at | Ppil4 | Peptidylprolyl isomerase (cyclophilin)-like 4 | | | **Protein processing** | | | 241 | 328 | 1.42 | 1.27 | * |
| 1384290_at | Rbbp6 | Retinoblastoma binding protein 6 | | | **Protein processing** | | | 780 | 887 | 1.28 | 1.26 | * |
| 1374514_at, 1375561_at | Rc3h1 | RING CCCH (C3H) domains 1 | | | **Protein processing** | | | 858, 596 | 1002, 523 | 1.22 | 1.32 | # |
| 1373456_at | Rnf111 | Ring finger protein 111 | | | **Protein processing** | | | 974 | 1249 | 1.21 | 1.30 | # |
| 1390524_at | Rnf12 | Ring finger protein 12 | | | **Protein processing** | | | 642 | 831 | 1.36 | 1.30 | * |
| 1382379_at, 1389258_at | Rnf138 | Ring finger protein 138 | | | **Protein processing** | | | 555, 293 | 604, 275 | 1.44 | 1.27 | *# |
| 1386685_at | Sacs | Sacsin | | | **Protein processing** | | | 262 | 539 | 1.25 | 1.37 | # |
| 1368487_at | Serpinb2 | Serine (or cysteine) proteinase inhibitor, clade B, member 2 | | | **Protein processing** | | | 314 | 354 | 15.78 | 17.02 | *# |
| 1368519_at, 1392264_s_at | Serpine1 | Serine (or cysteine) peptidase inhibitor, clade E, member 1 | | | **Protein processing** | | | 343, 140 | 480, 153 | 12.47 | 19.08 | *# |
| 1387408_at | Siah2 | Seven in absentia 2 | | | **Protein processing** | | | 262 | 228 | 1.91 | 1.61 | *# |
| 1387195_at | St14 | Suppression of tumorigenicity 14 (colon carcinoma) | | | **Protein processing** | | | 373 | 251 | 0.80 | 0.78 | * |
| 1377340_at | Tfpi2 | Tissue factor pathway inhibitor 2 | | | **Protein processing** | | | 256 | 270 | 1.99 | 2.10 | *# |
| 1367712_at | Timp1 | Tissue inhibitor of metallopeptidase 1 | | | **Protein processing** | | | 7965 | 7503 | 1.19 | 1.36 | # |
| 1372926_at, 1375138_at, 1389836_a_at | Timp3 | Tissue inhibitor of metalloproteinase 3 | | | **Protein processing** | | | 380, 1977, 2635 | 590, 2592, 2343 | 1.58 | 1.55 | *# |
| 1374446_at, 1385407_at | Tiparp | TCDD-inducible poly(ADP-ribose) polymerase | | | **Protein processing** | | | 1715, 1312 | 2600, 1629 | 1.98 | 1.55 | *# |
| 1389163_at | Trim32 | Tripartite motif protein 32 | | | **Protein processing** | | | 770 | 1014 | 0.70 | 0.71 | *# |
| 1389498_at | Tysnd1 | Trypsin domain containing 1 | | | **Protein processing** | | | 720 | 613 | 0.94 | 0.79 | # |
| 1392685_at | Ube2cbp | Ubiquitin-conjugating enzyme E2C binding protein | | | **Protein processing** | | | 282 | 180 | 0.78 | 0.68 | * |
| 1383023_at | Ube2h | Ubiquitin-conjugating enzyme E2H | | | **Protein processing** | | | 225 | 345 | 1.29 | 1.42 | # |
| 1373249_at | Ubl4 | Ubiquitin-like 4 | | | **Protein processing** | | | 769 | 561 | 0.90 | 0.80 | # |
| 1368076_at | Vhl | von Hippel-Lindau syndrome homolog | | | **Protein processing** | | | 1858 | 1133 | 0.77 | 0.76 | *# |
| 1374204_at | Wsb1 | WD repeat and SOCS box-containing 1 | | | **Protein processing** | | | 1762 | 2706 | 1.26 | 1.18 | * |
| 1383538_at | Zfp650 | Zinc finger protein 650 | | | **Protein processing** | | | 507 | 1340 | 0.88 | 0.76 | # |
| 1373917_at | Etf1 | Eukaryotic translation termination factor 1 | | | **Protein synthesis** | | | 1883 | 1524 | 1.21 | 1.28 | # |
| 1388517_at | Mrpl40 | Mitochondrial ribosomal protein L40 | | | **Protein synthesis** | | | 924 | 717 | 0.95 | 0.80 | # |
| 1372456_at | Mrps31 | Mitochondrial ribosomal protein S31 | | | **Protein synthesis** | | | 397 | 481 | 0.84 | 0.76 | # |
| 1368173_at | Nol5 | Nucleolar protein 5 | | | Protein synthesis | | | 1510 | 1826 | 1.17 | 1.27 | # |
| 1383831_at | Qrsl1 | Glutaminyl-tRNA synthase (glutamine-hydrolyzing)-like 1 | | | **Protein synthesis** | | | 381 | 496 | 0.81 | 0.79 | # |
| 1384926_at | Sdad1 | SDA1 domain containing 1 | | | **Protein synthesis** | | | 247 | 399 | 0.79 | 0.82 | * |
| 1393197_at | Abhd8 | Abhydrolase domain containing 8 | | | **Regulation of metabolism** | | | 722 | 572 | 0.88 | 0.77 | # |
| 1389229_at | Acpl2 | Acid phosphatase-like 2 | | | **Regulation of metabolism** | | | 1747 | 1709 | 0.94 | 0.79 | # |
| 1374610_at | Agpat9 | 1-acylglycerol-3-phosphate O-acyltransferase 9 | | | **Regulation of metabolism** | | | 370 | 432 | 1.60 | 2.10 | *# |
| 1368342_at | Ampd3 | Adenosine monophosphate deaminase 3 | | | **Regulation of metabolism** | | | 711 | 1257 | 0.94 | 0.80 | # |
| 1373302_at | Asah3l | N-acylsphingosine amidohydrolase 3-like | | | **Regulation of metabolism** | | | 615 | 455 | 0.77 | 0.66 | *# |
| 1373085_at | Cbr3 | Carbonyl reductase 3 | | | **Regulation of metabolism** | | | 282 | 80 | 0.76 | 0.46 | * |
| 1380063_at | Ch25h | Cholesterol 25-hydroxylase | | | **Regulation of metabolism** | | | 559 | 490 | 9.68 | 9.83 | *# |
| 1373866_at | Coq10b | Coenzyme Q10 homolog B (S. cerevisiae) | | | **Regulation of metabolism** | | | 1602 | 1485 | 2.54 | 2.54 | *# |
| 1398710_at | Cyp2u1 | Cytochrome P450, family 2, subfamily u, polypeptide 1 | | | **Regulation of metabolism** | | | 417 | 403 | 0.54 | 0.57 | *# |
| 1389261_at | Dhrs13 | Dehydrogenase/reductase (SDR family) member 13 | | | **Regulation of metabolism** | | | 382 | 345 | 0.88 | 0.74 | # |
| 1368336_at | Fdx1 | Ferredoxin 1 | | | **Regulation of metabolism** | | | 3720 | 2354 | 1.63 | 1.82 | *# |
| 1376753_at | Fpgt | Fucose-1-phosphate guanylyltransferase | | | **Regulation of metabolism** | | | 221 | 291 | 0.82 | 0.78 | # |
| 1373838_at | Fut4 | Fucosyltransferase 4 | | | **Regulation of metabolism** | | | 766 | 640 | 0.73 | 0.65 | *# |
| 1387221_at | Gch | GTP cyclohydrolase 1 | | | **Regulation of metabolism** | | | 313 | 290 | 1.75 | 1.49 | *# |
| 1373513_at | Gch1 | GTP cyclohydrolase 1 | | | **Regulation of metabolism** | | | 229 | 196 | 1.89 | 1.66 | *# |
| 1370688_at, 1372523_at | Gclc | Glutamate-cysteine ligase, catalytic subunit | | | **Regulation of metabolism** | | | 527, 799 | 840, 1041 | 1.69 | 1.49 | *# |
| 1374903_at | Gcnt2 | Glucosaminyl (N-acetyl) transferase 2, I-branching enzyme | | | **Regulation of metabolism** | | | 275 | 368 | 1.50 | 1.28 | * |
| 1367867_at | Gfer | Growth factor, erv1 homolog (S. cerevisiae) | | | **Regulation of metabolism** | | | 715 | 425 | 0.77 | 0.84 | * |
| 1377761_at | Gfpt2 | Glutamine-fructose-6-phosphate transaminase 2 | | | **Regulation of metabolism** | | | 3707 | 4599 | 1.23 | 1.43 | # |
| 1367705_at, 1386908_at | Glrx1 | Glutaredoxin 1 (thioltransferase) | | | **Regulation of metabolism** | | |  |  | 1.41 | 1.32 | *# |
| 1367774_at | Gsta3 | Glutathione S-transferase A3 | | | **Regulation of metabolism** | | | 4674 | 3451 | 0.94 | 0.79 | # |
| 1387548_at | Has2 | Hyaluronan synthase 2 | | | **Regulation of metabolism** | | | 340 | 413 | 8.44 | 9.38 | *# |
| 1370239_at, 1375519_at, 1370240_x_at | Hba-a1 | Hemoglobin alpha, adult chain 1/2 | | | **Regulation of metabolism** | | | 4707, 464, 4242 | 10725, 1265, 10236 | 0.73 | 0.83 | * |
| 1367553_x_at1371102_x_at1371245_a_at | Hbb-b1 | Hemoglobin beta chain complex | | | **Regulation of metabolism** | | | 2152, 595, 1582 | 5010, 1523, 3358 | 0.67 | 0.75 | *# |
| 1369006_at, 1383519_at | Hk2 | Hexokinase 2 | | | **Regulation of metabolism** | | | 245, 637 | 546, 1114 | 1.31 | 1.48 | *# |
| 1375852_at | Hmgcr | 3-hydroxy-3-methylglutaryl-Coenzyme A reductase | | | **Regulation of metabolism** | | | 1110 | 2051 | 1.79 | 1.82 | *# |
| 1368878_at, 1388872_at | Idi1 | Isopentenyl-diphosphate delta isomerase | | | **Regulation of metabolism** | | | 3600, 1785 | 3111, 1817 | 1.57 | 1.60 | *# |
| 1367894_at | Insig1 | Insulin-induced gene 1 protein | | | **Regulation of metabolism** | | | 5919 | 4726 | 1.37 | 1.68 | *# |
| 1372524_at | Lpin1 | Lipin 1 | | | **Regulation of metabolism** | | | 259 | 458 | 0.72 | 0.78 | *# |
| 1376660_at, 1387570_at | Manea | Mannosidase, endo-alpha | | | **Regulation of metabolism** | | | 907, 152 | 1248, 308 | 1.50 | 1.43 | *# |
| 1371350_at, 1387737_at | Mat2a | Methionine adenosyltransferase II, alpha | | | **Regulation of metabolism** | | | 1928, 355 | 3333, 822 | 2.37 | 2.12 | *# |
| 1372198_at | Mepce | Methylphosphate capping enzyme | | | **Regulation of metabolism** | | | 563 | 674 | 0.77 | 0.72 | *# |
| 1367796_at | Mgat1 | Mannoside acetylglucosaminyltransferase 1 | | | **Regulation of metabolism** | | | 998 | 997 | 1.43 | 1.36 | *# |
| 1372808_at | Mthfd2 | Methylenetetrahydrofolate dehydrogenase (NAD+ dependent), methenyltetrahydrofolate cyclohydrolase | | | **Regulation of metabolism** | | | 1210 | 664 | 1.32 | 1.51 | *# |
| 1369200_at | Nt5e | 5' nucleotidase, ecto | | | **Regulation of metabolism** | | | 1071 | 1384 | 0.78 | 0.86 | * |
| 1368091_at | Oplah | 5-oxoprolinase (ATP-hydrolysing) | | | **Regulation of metabolism** | | | 250 | 402 | 0.85 | 0.75 | # |
| 1391530_a_at | Oxsm | 3-oxoacyl-ACP synthase, mitochondrial | | | **Regulation of metabolism** | | | 277 | 229 | 0.72 | 0.73 | * |
| 1389014_at | Pbef1 | Pre-B-cell colony enhancing factor 1 | | | **Regulation of metabolism** | | | 861 | 962 | 1.19 | 1.32 | # |
| 1370407_at | Pcyox1 | Prenylcysteine oxidase 1 | | | **Regulation of metabolism** | | | 631 | 777 | 0.85 | 0.77 | # |
| 1373854_at | Pdpr | Pyruvate dehydrogenase phosphatase regulatory subunit | | | **Regulation of metabolism** | | | 472 | 1433 | 0.78 | 0.87 | * |
| 1369029_at | Plscr1 | Phospholipid scramblase 1 | | | **Regulation of metabolism** | | | 574 | 516 | 1.66 | 1.36 | *# |
| 1368527_at | Ptgs2 | Prostaglandin-endoperoxide synthase 2 | | | **Regulation of metabolism** | | | 327 | 427 | 15.35 | 14.36 | *# |
| 1374680_at | Rdh13 | Retinol dehydrogenase 13 (all-trans and 9-cis) | | | **Regulation of metabolism** | | | 342 | 395 | 0.83 | 0.69 | # |
| 1382179_at | Rdh14 | Retinol dehydrogenase 14 (all-trans and 9-cis) | | | **Regulation of metabolism** | | | 885 | 701 | 0.82 | 0.71 | # |
| 1371774_at | Sat | Spermidine/spermine N1-acetyl transferase | | | **Regulation of metabolism** | | | 5125 | 3725 | 1.38 | 2.05 | *# |
| 1372876_at | Sephs2 | Selenophosphate synthetase 2 | | | **Regulation of metabolism** | | | 1462 | 893 | 0.84 | 0.77 | # |
| 1382500_at, 1386662_at, 1391607_at | Sesn2 | Sestrin 2 | | | **Regulation of metabolism** | | | 648, 366, 1002 | 550, 341, 722 | 1.91 | 1.65 | *# |
| 1387017_at | Sqle | Squalene epoxidase | | | **Regulation of metabolism** | | | 1848 | 2981 | 1.12 | 1.25 | # |
| 1372510_at, 1384331_at | Srxn1 | Sulfiredoxin 1 homolog (S. cerevisiae) | | | **Regulation of metabolism** | | | 1202, 309 | 615, 328 | 5.34 | 5.47 | *# |
| 1379910_at | Uap1 | UDP-N-acetylglucosamine pyrophosphorylase 1 | | | **Regulation of metabolism** | | | 948 | 932 | 1.63 | 2.73 | *# |
| 1387975_at | Ugcg | UDP-glucose ceramide glucosyltransferase | | | **Regulation of metabolism** | | | 1114 | 835 | 1.34 | 1.41 | *# |
| 1367938_at | Ugdh | UDP-glucose dehydrogenase | | | **Regulation of metabolism** | | | 4203 | 4723 | 1.21 | 1.35 | # |
| 1390628_at | Cpeb2 | Cytoplasmic polyadenylation element binding protein 2 | | | **RNA regulation** | | | 471 | 757 | 1.80 | 1.46 | *# |
| 1375484_at, 1385505_at | Cpeb3 | Cytoplasmic polyadenylation element binding protein 3 | | | **RNA regulation** | | | 239, 222 | 265, 365 | 1.31 | 1.18 | * |
| 1372223_at, 1393294_at | Cpeb4 | Cytoplasmic polyadenylation element binding protein 4 | | | **RNA regulation** | | | 1449, 914 | 2355, 1695 | 1.48 | 1.35 | *# |
| 1393166_at | Cstf3 | Cleavage stimulation factor, 3' pre-RNA, subunit 3 | | | **RNA regulation** | | | 252 | 376 | 1.22 | 1.33 | # |
| 1386535_at | Ddx3x | DEAD/H (Asp-Glu-Ala-Asp/His) box polypeptide 3, X-linked | | | **RNA regulation** | | | 621 | 1534 | 0.74 | 1.30 | *# |
| 1381075_at | Helz | Helicase with zinc finger | | | **RNA regulation** | | | 303 | 451 | 0.76 | 0.74 | *# |
| 1372693_at | Hnrpa1 | Heterogeneous nuclear ribonucleoprotein A1 | | | **RNA regulation** | | | 601 | 823 | 0.74 | 0.75 | *# |
| 1385487_at | Lsm1 | LSM1 homolog, U6 small nuclear RNA associated | | | **RNA regulation** | | | 1518 | 952 | 0.87 | 0.79 | # |
| 1395595_at | Mki67ip | Mki67 (FHA domain) interacting nucleolar phosphoprotein | | | **RNA regulation** | | | 410 | 498 | 1.38 | 1.26 | *# |
| 1373509_at | Nsun4 | NOL1/NOP2/Sun domain family, member 4 | | | **RNA regulation** | | | 374 | 507 | 1.13 | 1.32 | # |
| 1383929_at | Nufip2 | Nuclear fragile X mental retardation protein interacting protein 2 | | | **RNA regulation** | | | 550 | 840 | 0.72 | 0.71 | *# |
| 1392579_at | Obfc2a | Oligonucleotide/oligosaccharide-binding fold containing 2A | | | **RNA regulation** | | | 472 | 521 | 1.27 | 1.23 | * |
| 1390576_at, 1395129_at | Rbm15 | RNA binding motif protein 15 | | | **RNA regulation** | | | 612, 431 | 646, 270 | 1.34 | 1.58 | *# |
| 1371952_at, 1393569_at | Rbm18 | RNA binding motif protein 18 | | | **RNA regulation** | | | 2073, 716 | 1445, 532 | 1.24 | 1.31 | # |
| 1373730_at, 1377793_at | Rbm33 | RNA binding motif protein 33 | | | **RNA regulation** | | | 762, 174 | 700, 324 | 1.01 | 0.96 | # |
| 1382749_at | Rbm5 | RNA binding motif protein 5 | | | **RNA regulation** | | | 328 | 1415 | 0.83 | 0.70 | # |
| 1382287_at | Rnps1 | Ribonucleic acid binding protein S | | | **RNA regulation** | | | 567 | 678 | 1.27 | 1.11 | * |
| 1376110_at | Rpp25 | Ribonuclease P 25 subunit (human) | | | **RNA regulation** | | | 2567 | 1317 | 0.81 | 0.77 | # |
| 1393193_at | Rpusd2 | RNA pseudouridylate synthase domain containing 2 | | | **RNA regulation** | | | 349 | 336 | 0.73 | 0.65 | * |
| 1379358_at | Samd4a | Sterile alpha motif domain containing 4A | | | **RNA regulation** | | | 433 | 208 | 0.80 | 0.94 | * |
| 1388132_at | Sfpq | Splicing factor proline/glutamine rich (polypyrimidine tract binding protein associated) | | | **RNA regulation** | | | 766 | 1283 | 1.22 | 1.27 | # |
| 1387824_at | Sfrs12 | Splicing factor, arginine/serine-rich 12 | | | **RNA regulation** | | | 166 | 252 | 1.10 | 1.29 | # |
| 1372075_at | Sfrs6 | splicing factor, arginine/serine-rich 6 | | | **RNA regulation** | | | 2454 | 3007 | 1.36 | 1.26 | *# |
| 1393153_at | Tardbp | TAR DNA binding protein | | | **RNA regulation** | | | 388 | 298 | 0.58 | 0.58 | * |
| 1383660_at | Thoc4 | THO complex 4 | | | **RNA regulation** | | | 395 | 379 | 1.33 | 1.12 | * |
| 1379498_at | Trmt61b | tRNA methyltransferase 61 homolog B (S. cerevisiae) | | | **RNA regulation** | | | 380 | 267 | 0.80 | 0.71 | * |
| 1373708_at | Tut1 | Terminal uridylyl transferase 1, U6 snRNA-specific | | | **RNA regulation** | | | 294 | 286 | 0.79 | 0.78 | * |
| 1382972_at | Utp15 | UTP15, U3 small nucleolar ribonucleoprotein | | | **RNA regulation** | | | 341 | 418 | 1.23 | 1.35 | # |
| 1382175_at | Wtap | Wilms tumor 1 associated protein | | | **RNA regulation** | | | 2498 | 1584 | 0.81 | 0.78 | # |
| 1387870_at | Zfp36 | Zinc finger protein 36 | | | **RNA regulation** | | | 999 | 1255 | 3.65 | 3.35 | *# |
| 1369959_at | Zfp36l1 | Zinc finger protein 36, C3H type-like 1 | | | **RNA regulation** | | | 4821 | 4844 | 1.40 | 1.28 | *# |
| 1373106_at | Zfp36l2 | Zinc finger protein 36, C3H type-like 2 | | | **RNA regulation** | | | 2328 | 2154 | 1.75 | 1.61 | *# |
| 1390881_at | Abra | Actin-binding Rho activating protein | | | **Signalling/Trafficking** | | | 354 | 477 | 3.12 | 2.97 | *# |
| 1373843_at, 1382206_a_at 1385566_at | Akap2 | A kinase (PRKA) anchor protein 2 | | | **Signalling/Trafficking** | | | 4050, 681, 121 | 5706, 790, 207 | 2.35 | 1.92 | *# |
| 1371873_at | Anp32e | Acidic (leucine-rich) nuclear phosphoprotein 32 family, member E | | | **Signalling/Trafficking** | | | 1226 | 1369 | 0.89 | 0.76 | # |
| 1383091_at | Appbp2 | Amyloid beta precursor protein (cytoplasmic tail) binding protein 2 | | | **Signalling/Trafficking** | | | 758 | 782 | 1.31 | 1.43 | *# |
| 1368946_at, 1388813_at | Arf2 | ADP-ribosylation factor 2 | | | **Signalling/Trafficking** | | | 537, 750 | 508, 651 | 1.40 | 1.17 | * |
| 1390786_at | Arhgef2 | rho/rac guanine nucleotide exchange factor (GEF) 2 | | | **Signalling/Trafficking** | | | 1137 | 1178 | 1.16 | 1.32 | # |
| 1377750_at | Arhgef3 | Rho guanine nucleotide exchange factor (GEF) 3 | | | **Signalling/Trafficking** | | | 397 | 359 | 2.43 | 3.28 | *# |
| 1367960_at | Arl4 | ADP-ribosylation factor-like 4 | | | **Signalling/Trafficking** | | | 1581 | 1352 | 1.61 | 1.38 | *# |
| 1376275_at | Arl5b | ADP-ribosylation factor-like 5B | | | **Signalling/Trafficking** | | | 782 | 643 | 1.79 | 1.74 | *# |
| 1368260_at | Aurkb | Aurora kinase B | | | **Signalling/Trafficking** | | | 547 | 453 | 0.92 | 0.80 | # |
| 1389402_at | Axud1 | AXIN1 up-regulated 1 | | | **Signalling/Trafficking** | | | 375 | 314 | 8.31 | 8.97 | *# |
| 1382993_at | Bbc3 | Bcl-2 binding component 3 | | | **Signalling/Trafficking** | | | 1142 | 631 | 0.69 | 0.77 | *# |
| 1367752_at | Bcar1 | Breast cancer anti-estrogen resistance 1 | | | **Signalling/Trafficking** | | | 1077 | 695 | 1.26 | 1.31 | # |
| 1368118_at | Bcl10 | B-cell CLL/lymphoma 10 | | | **Signalling/Trafficking** | | | 2454 | 1654 | 1.28 | 1.24 | * |
| 1388742_at | Bcl2l11 | BCL2-like 11 | | | **Signalling/Trafficking** | | | 1168 | 1016 | 0.77 | 0.63 | *# |
| 1373494_at, 1389465_at | Bcr | Breakpoint cluster region | | | **Signalling/Trafficking** | | | 1559, 964 | 996, 984 | 2.41 | 2.66 | *# |
| 1372950_at, 1387448_at | Bet1l | Blocked early in transport 1 homolog (S. cerevisiae) like | | | **Signalling/Trafficking** | | | 1138, 477 | 682, 380 | 0.78 | 0.74 | *# |
| 1374493_at | Bmf | Bcl2 modifying factor | | | **Signalling/Trafficking** | | | 518 | 466 | 0.71 | 0.62 | *# |
| 1368050_at | Ccnl1 | Cyclin L1 | | | **Signalling/Trafficking** | | | 356 | 943 | 2.19 | 1.89 | *# |
| 1389157_at | Cdc42ep1 | CDC42 effector protein (Rho GTPase binding) 1 | | | **Signalling/Trafficking** | | | 1555 | 1024 | 0.83 | 0.79 | # |
| 1389145_at | Cdc42ep2 | CDC42 effector protein (Rho GTPase binding) 2 | | | **Signalling/Trafficking** | | | 1024 | 562 | 0.60 | 0.69 | * |
| 1375910_at | Cdc42ep3 | CDC42 effector protein (Rho GTPase binding) 3 | | | **Signalling/Trafficking** | | | 4839 | 3587 | 1.70 | 2.02 | *# |
| 1388730_at | Cdc42ep4 | CDC42 effector protein (Rho GTPase binding) 4 | | | **Signalling/Trafficking** | | | 1843 | 1314 | 0.78 | 0.72 | *# |
| 1387391_at, 1388674_at | Cdkn1a | Cyclin-dependent kinase inhibitor 1A | | | **Signalling/Trafficking** | | | 868, 4359 | 542, 2117 | 1.32 | 1.80 | *# |
| 1373812_at | Cdkn1b | Cyclin-dependent kinase inhibitor 1B | | | **Signalling/Trafficking** | | | 2407 | 2271 | 0.85 | 0.72 | # |
| 1377829_at | Cep57 | Centrosomal protein 57 | | | **Signalling/Trafficking** | | | 316 | 422 | 0.73 | 0.72 | *# |
| 1383764_at | Chic2 | Cysteine-rich hydrophobic domain 2 | | | **Signalling/Trafficking** | | | 428 | 349 | 1.25 | 1.14 | * |
| 1373425_at | Clk2 | CDC-like kinase 2 | | | **Signalling/Trafficking** | | | 351 | 467 | 0.84 | 0.76 | # |
| 1389368_at | Cnksr3 | Cnksr family member 3 | | | **Signalling/Trafficking** | | | 693 | 1075 | 1.59 | 1.83 | *# |
| 1368025_at | Ddit4 | DNA-damage-inducible transcript 4 | | | **Signalling/Trafficking** | | | 3051 | 2986 | 0.31 | 0.16 | *# |
| 1384525_at | Dock11 | Dedicator of cytokinesis 11 | | | **Signalling/Trafficking** | | | 384 | 671 | 0.88 | 0.79 | # |
| 1377982_at, 1373114_at | Dtx4 | Deltex 4 homolog (Drosophila) | | | **Signalling/Trafficking** | | | 439, 2189 | 407, 1381 | 0.77 | 0.76 | *# |
| 1368146_at, 1368147_at | Dusp1 | Dual specificity phosphatase 1 | | | **Signalling/Trafficking** | | | 2345, 131 | 2320, 237 | 3.55 | 2.68 | *# |
| 1394028_at | Dusp10 | Dual specificity phosphatase 10 | | | **Signalling/Trafficking** | | | 227 | 247 | 1.81 | 2.08 | *# |
| 1390962_at | Dusp11 | Dual specificity phosphatase 11 | | | **Signalling/Trafficking** | | | 424 | 396 | 0.74 | 0.86 | * |
| 1373324_at | Dusp14 | Dual specificity phosphatase 14 | | | **Signalling/Trafficking** | | | 433 | 274 | 1.30 | 1.44 | * |
| 1372354_at | Dusp16 | Dual specificity phosphatase 16 | | | **Signalling/Trafficking** | | | 465 | 395 | 1.30 | 1.46 | *# |
| 1377023_at | Dusp2 | Dual specificity phosphatase 2 | | | **Signalling/Trafficking** | | | 559 | 444 | 1.62 | 1.72 | *# |
| 1391324_at | Dusp27 | Dual specificity phosphatase 27 | | | **Signalling/Trafficking** | | | 1034 | 1761 | 1.26 | 1.23 | * |
| 1393119_at | Dusp4 | Dual specificity phosphatase 4 | | | **Signalling/Trafficking** | | | 1431 | 1412 | 1.80 | 1.96 | *# |
| 1368124_at | Dusp5 | Dual specificity phosphatase 5 | | | **Signalling/Trafficking** | | | 826 | 681 | 4.52 | 4.80 | *# |
| 1377064_at, 1387024_at | Dusp6 | Dual specificity phosphatase 6 | | | **Signalling/Trafficking** | | | 2693, 3778 | 1350, 2253 | 2.06 | 3.47 | *# |
| 1377992_at | Dusp7 | Dual specificity phosphatase 7 | | | **Signalling/Trafficking** | | | 966 | 614 | 0.71 | 0.70 | *# |
| 1372385_at | Dusp8 | Dual specificity phosphatase 8 | | | **Signalling/Trafficking** | | | 472 | 411 | 1.62 | 1.56 | *# |
| 1369736_at, 1371527_at | Emp1 | Epithelial membrane protein 1 | | | **Signalling/Trafficking** | | | 1028, 7831 | 1390, 4530 | 1.54 | 1.72 | *# |
| 1377213_at | Ern1 | Endoplasmic reticulum (ER) to nucleus signalling 1 | | | **Signalling/Trafficking** | | | 357 | 550 | 1.43 | 1.40 | *# |
| 1373093_at | Errfi1 | ERBB receptor feedback inhibitor 1 | | | **Signalling/Trafficking** | | | 1946 | 1963 | 5.15 | 5.23 | *# |
| 1378253_at | Exoc8 | Exocyst complex component 8 | | | **Signalling/Trafficking** | | | 461 | 528 | 0.78 | 0.75 | *# |
| 1389638_at | Fam160a2 | Family with sequence Similarity 160, member A2 | | | **Signalling/Trafficking** | | | 479 | 326 | 1.19 | 1.39 | # |
| 1382774_at | Frat1 | Frequently rearranged in advanced T-cell lymphomas | | | **Signalling/Trafficking** | | | 1053 | 587 | 0.76 | 0.60 | *# |
| 1368947_at | Gadd45a | Growth arrest and DNA-damage-inducible 45 alpha | | | **Signalling/Trafficking** | | | 2691 | 2147 | 1.44 | 1.74 | *# |
| 1372016_at | Gadd45b | Growth arrest and DNA-damage-inducible 45 beta | | | **Signalling/Trafficking** | | | 796 | 563 | 1.42 | 1.64 | # |
| 1388792_at | Gadd45g | Growth arrest and DNA-damage-inducible 45 gamma | | | **Signalling/Trafficking** | | | 1899 | 1419 | 2.53 | 2.59 | *# |
| 1382351_at | Gem | GTP binding protein | | | **Signalling/Trafficking** | | | 392 | 448 | 1.85 | 1.73 | # |
| 1390134_at | Gna13 | Guanine nucleotide binding protein, alpha 13 | | | **Signalling/Trafficking** | | | 1258 | 1011 | 0.71 | 0.76 | *# |
| 1383167_at | Golph3l | Golgi phosphoprotein 3-like | | | **Signalling/Trafficking** | | | 778 | 807 | 0.84 | 0.78 | # |
| 1387000_at | Gorasp1 | Golgi reassembly stacking protein 1 | | | **Signalling/Trafficking** | | | 548 | 407 | 0.78 | 0.82 | * |
| 1388587_at | Ier3 | Immediate early response 3 | | | **Signalling/Trafficking** | | | 4989 | 4246 | 1.49 | 1.66 | *# |
| 1387063_at | Ihpk2 | Inositol hexaphosphate kinase 2 | | | **Signalling/Trafficking** | | | 279 | 408 | 1.37 | 1.15 | * |
| 1373992_at | Iigp1 | Interferon inducible GTPase 1 | | | **Signalling/Trafficking** | | | 608 | 605 | 0.72 | 0.71 | # |
| 1382820_at | Ikbkap | Inhibitor of kappa light polypeptide enhancer in B-cells, kinase complex-associated protein | | | **Signalling/Trafficking** | | | 167 | 394 | 0.72 | 0.74 | # |
| 1371091_at | Irs2 | Insulin receptor substrate 2 | | | **Signalling/Trafficking** | | | 601 | 959 | 2.19 | 2.29 | *# |
| 1374462_at | Kifap3 | Kinesin-associated protein 3 | | | **Signalling/Trafficking** | | | 555 | 882 | 0.95 | 0.80 | # |
| 1369610_at | Lin7c | Lin-7 homolog C | | | **Signalling/Trafficking** | | | 219 | 285 | 1.32 | 1.20 | * |
| 1385238_at | Lman1 | Lectin, mannose-binding, 1 | | | **Signalling/Trafficking** | | | 1175 | 2277 | 0.86 | 0.78 | # |
| 1395316_at | Mageh1 | Melanoma antigen, family H, 1 | | | **Signalling/Trafficking** | | | 1134 | 928 | 0.94 | 0.79 | # |
| 1368871_at, 1375673_at | Map3k1 | Mitogen activated protein kinase kinase kinase 1 | | | **Signalling/Trafficking** | | | 1006, 431 | 1128, 676 | 0.69 | 0.63 | *# |
| 1372912_at | Map3k7ip1 | Mitogen-activated protein kinase kinase kinase 7 interacting protein 1 | | | **Signalling/Trafficking** | | | 855 | 779 | 0.75 | 0.73 | *# |
| 1395621_at | Mcart2 | Mitochondrial carrier triple repeat 2 | | | **Signalling/Trafficking** | | | 619 | 527 | 0.85 | 0.78 | # |
| 1370141_at, 1372520_at, 1373225_at | Mcl1 | Myeloid cell leukemia sequence 1 | | | **Signalling/Trafficking** | | | 1737, 5285, 3438 | 2383, 5372, 3427 | 1.44 | 1.44 | *# |
| 1372904_at, 1384900_at | Mobkl2b | MOB1, Mps One Binder kinase activator-like 2B (yeast) | | | **Signalling/Trafficking** | | | 1175, 208 | 668, 255 | 1.67 | 1.94 | *# |
| 1389072_at | Mtmr4 | Myotubularin related protein 4 | | | **Signalling/Trafficking** | | | 483 | 518 | 0.76 | 0.72 | *# |
| 1372236_at | Nod1 | Nucleotide-binding oligomerization domain containing 1 | | | **Signalling/Trafficking** | | | 756 | 675 | 0.89 | 0.75 | # |
| 1376282_at | Nuak1 | NUAK family, SNF1-like kinase, 1 | | | **Signalling/Trafficking** | | | 708 | 807 | 1.30 | 1.40 | *# |
| 1383614_at | Nuak2 | NUAK family, SNF1-like kinase, 2 | | | **Signalling/Trafficking** | | | 421 | 410 | 1.26 | 1.31 | # |
| 1372022_at | Nup50 | Nucleoporin 50 | | | **Signalling/Trafficking** | | | 300 | 329 | 0.82 | 0.77 | # |
| 1387058_at | Pctp | Phosphatidylcholine transfer protein | | | **Signalling/Trafficking** | | | 654 | 352 | 0.81 | 0.79 | # |
| 1389322_at | Pdcd7 | Programmed cell death protein 7 | | | **Signalling/Trafficking** | | | 690 | 642 | 0.74 | 0.69 | # |
| 1373052_at, 1377779_at | Pdcl3 | Phosducin-like 3 | | | **Signalling/Trafficking** | | | 2546, 1059 | 1916, 757 | 1.26 | 1.28 | *# |
| 1369070_at | Pex12 | Peroxisomal biogenesis factor 12 | | | **Signalling/Trafficking** | | | 324 | 369 | 0.90 | 0.77 | # |
| 1374638_at | Pex13 | Peroxisomal biogenesis factor 13 | | | **Signalling/Trafficking** | | | 1339 | 1419 | 1.25 | 1.20 | * |
| 1383960_at | Pex16 | Peroxisome biogenesis factor 16 | | | **Signalling/Trafficking** | | | 625 | 523 | 0.78 | 0.89 | * |
| 1384950_at, 1395655_at | Pi4k2b | Phosphatidylinositol 4-kinase type 2 beta | | | **Signalling/Trafficking** | | | 207, 937 | 224, 784 | 1.26 | 1.33 | *# |
| 1393499_at | Pik3ca | Phosphatidylinositol 3-kinase, catalytic, alpha polypeptide | | | **Signalling/Trafficking** | | | 548 | 841 | 0.83 | 0.75 | # |
| 1388525_at | Pik3ip1 | Phosphoinositide-3-kinase interacting protein 1 | | | **Signalling/Trafficking** | | | 914 | 1160 | 0.61 | 0.55 | *# |
| 1385800_at | Pim1 | Proviral integration site 1 | | | **Signalling/Trafficking** | | | 381 | 345 | 1.09 | 1.29 | # |
| 1367725_at | Pim3 | Serine/threonine-protein kinase pim-3 | | | **Signalling/Trafficking** | | | 1379 | 1130 | 1.45 | 1.33 | * |
| 1368982_at | Pkia | Protein kinase inhibitor, alpha | | | **Signalling/Trafficking** | | | 560 | 856 | 1.06 | 1.26 | # |
| 1373532_at | Plekhf1 | Pleckstrin homology domain containing, family F (with FYVE domain) member 1 | | | **Signalling/Trafficking** | | | 1134 | 1082 | 0.60 | 0.61 | *# |
| 1368106_at | Plk2 | Polo-like kinase 2 (Drosophila) | | | **Signalling/Trafficking** | | | 2079 | 2643 | 3.52 | 3.78 | *# |
| 1373108_at | Pp1r3c | Protein phosphatase 1, regulatory (inhibitor) subunit 3C | | | **Signalling/Trafficking** | | | 1278 | 1626 | 0.78 | 0.69 | # |
| 1371897_at | Ppapdc3 | Phosphatidic acid phosphatase type 2 domain containing 3 | | | **Signalling/Trafficking** | | | 862 | 725 | 0.74 | 0.77 | # |
| 1368910_at | Ppm2c | Protein phosphatase 2C, magnesium dependent, catalytic subunit | | | **Signalling/Trafficking** | | | 767 | 1108 | 1.43 | 1.44 | *# |
| 1374473_at | Ppp1r15b | Protein phosphatase 1, regulatory (inhibitor) subunit 15b | | | **Signalling/Trafficking** | | | 902 | 740 | 1.40 | 1.46 | # |
| 1384262_at | Ppp1r3b | Protein phosphatase 1, regulatory (inhibitor) subunit 3B | | | **Signalling/Trafficking** | | | 677 | 914 | 0.48 | 0.31 | *# |
| 1373656_at | Ppp1r3d | Protein phosphatase 1, regulatory subunit 3D | | | **Signalling/Trafficking** | | | 1205 | 1073 | 0.85 | 0.78 | # |
| 1373952_at | Prkag2 | Protein kinase, AMP-activated, gamma 2 non-catalytic subunit | | | **Signalling/Trafficking** | | | 555 | 437 | 0.83 | 0.78 | # |
| 1367727_at | Pscd2 | Pleckstrin homology, Sec7 and coiled-coil domains 2 | | | **Signalling/Trafficking** | | | 1766 | 1278 | 1.28 | 1.21 | * |
| 1370193_at | Ptp4a1 | Protein tyrosine phosphatase 4a1 | | | **Signalling/Trafficking** | | | 5151 | 3509 | 1.38 | 1.58 | *# |
| 1383232_at | Rab33b | RAB33B, member of RAS oncogene family | | | **Signalling/Trafficking** | | | 730 | 569 | 0.82 | 0.71 | # |
| 1391274_at | Rab3ip | RAB3A interacting protein | | | **Signalling/Trafficking** | | | 543 | 345 | 0.75 | 0.90 | * |
| 1367825_at | Ralgds | Ral guanine nucleotide dissociation stimulator | | | **Signalling/Trafficking** | | | 710 | 459 | 0.71 | 0.81 | * |
| 1386900_at | RAMP4 | Ribosome associated membrane protein 4 | | | **Signalling/Trafficking** | | | 3286 | 2081 | 1.16 | 1.31 | # |
| 1373955_at, 1396742_at | Ranbp5 | RAN binding protein 5 | | | **Signalling/Trafficking** | | | 1806, 59 | 3015, 407 | 1.26 | 1.34 | # |
| 1383322_at | Rasl11b | RAS-like family 11 member B | | | **Signalling/Trafficking** | | | 1434 | 1116 | 2.06 | 2.41 | *# |
| 1378484_at | Rasl12 | RAS-like, family 12 | | | **Signalling/Trafficking** | | | 535 | 442 | 0.59 | 0.61 | *# |
| 1373989_at | Rassf1 | Ras association (RalGDS/AF-6) domain family 1 | | | **Signalling/Trafficking** | | | 754 | 537 | 1.57 | 1.57 | *# |
| 1388686_at | Rcan1 | Regulator of calcineurin 1 | | | **Signalling/Trafficking** | | | 7512 | 5992 | 1.31 | 1.59 | *# |
| 1372875_at | Rcan3 | Regulator of calcineurin 3 | | | **Signalling/Trafficking** | | | 848 | 538 | 0.76 | 0.81 | * |
| 1373777_at | Rgs16 | Regulator of G-protein signaling 16 | | | **Signalling/Trafficking** | | | 790 | 429 | 1.55 | 1.33 | * |
| 1368144_at, 1387074_at | Rgs2 | Regulator of G-protein signaling 2 | | | **Signalling/Trafficking** | | | 1725, 3188 | 1175, 1977 | 4.21 | 5.66 | *# |
| 1369958_at | Rhob | Ras homolog gene family, member B | | | **Signalling/Trafficking** | | | 3705 | 2376 | 2.56 | 3.18 | *# |
| 1395699_at | Riok3 | RIO kinase 3 (yeast) | | | **Signalling/Trafficking** | | | 442 | 650 | 1.24 | 1.34 | # |
| 1381279_at | Ripk2 | Receptor (TNFRSF)-interacting serine-threonine kinase 2 | | | **Signalling/Trafficking** | | | 990 | 773 | 2.04 | 2.66 | *# |
| 1382148_at | Rit1 | Ras-like without CAAX 1 | | | **Signalling/Trafficking** | | | 633 | 487 | 0.71 | 0.84 | * |
| 1381533_at | Rnd1 | Rho family GTPase 1 | | | **Signalling/Trafficking** | | | 2128 | 1026 | 2.32 | 3.77 | *# |
| 1376066_at, 1377663_at, 1394077_at | Rnd3 | Rho family GTPase 3 | | | **Signalling/Trafficking** | | | 1421, 2391, 714 | 1203, 1483, 741 | 1.75 | 2.12 | *# |
| 1367862_at | Rrad | Ras-related associated with diabetes | | | **Signalling/Trafficking** | | | 1672 | 2283 | 0.73 | 0.64 | *# |
| 1382835_at | Rragd | Ras-related GTP binding D | | | **Signalling/Trafficking** | | | 768 | 669 | 0.92 | 0.77 | # |
| 1379285_at | Rtp4 | Receptor (chemosensory) transporter protein 4 | | | **Signalling/Trafficking** | | | 485 | 371 | 0.85 | 0.75 | # |
| 1389138_at | Sec22b | SEC22 vesicle trafficking protein homolog B (S. cerevisiae) | | | **Signalling/Trafficking** | | | 1837 | 1310 | 0.87 | 0.73 | # |
| 1380486_at | Sec22c | SEC22 vesicle trafficking protein-like C (S. cerevisiae) | | | **Signalling/Trafficking** | | | 307 | 226 | 0.80 | 0.88 | * |
| 1390249_at | Sept14 | Septin 14 | | | **Signalling/Trafficking** | | | 598 | 560 | 0.75 | 0.56 | *# |
| 1367802_at | Sgk | Serum/glucocorticoid regulated kinase | | | **Signalling/Trafficking** | | | 1985 | 2220 | 2.92 | 2.20 | *# |
| 1368605_at | Sh2b2 | SH2B adaptor protein 2 | | | **Signalling/Trafficking** | | | 599 | 353 | 0.82 | 0.73 | # |
| 1390298_at | Snag1 | Sorting nexin associated golgi protein 1 | | | **Signalling/Trafficking** | | | 2138 | 2297 | 1.17 | 1.25 | # |
| 1368596_at | Snf1lk | SNF1-like kinase | | | **Signalling/Trafficking** | | | 497 | 567 | 1.71 | 2.24 | *# |
| 1383653_at | Snx11 | Sorting nexin 11 | | | **Signalling/Trafficking** | | | 562 | 317 | 0.72 | 0.73 | * |
| 1390555_at | Socs5 | Suppressor of cytokine signaling 5 | | | **Signalling/Trafficking** | | | 1707 | 2091 | 1.24 | 1.30 | # |
| 1389460_at | Socs6 | Suppressor of cytokine signaling 6 | | | **Signalling/Trafficking** | | | 396 | 463 | 0.73 | 0.58 | *# |
| 1372027_at | Spata13 | Spermatogenesis associated 13 | | | **Signalling/Trafficking** | | | 300 | 328 | 0.80 | 0.86 | * |
| 1372633_at | Spg20 | Spastic paraplegia 20, spartin (Troyer syndrome) homolog | | | **Signalling/Trafficking** | | | 615 | 718 | 0.66 | 0.60 | *# |
| 1374864_at | Spry2 | Sprouty homolog 2 (Drosophila) | | | **Signalling/Trafficking** | | | 1742 | 1551 | 2.46 | 2.40 | *# |
| 1392529_at | Spry4 | Sprouty homolog 4 (Drosophila) | | | **Signalling/Trafficking** | | | 1636 | 1436 | 1.25 | 1.33 | # |
| 1391871_at | Stard13 | START domain containing 13 | | | **Signalling/Trafficking** | | | 613 | 886 | 1.32 | 1.28 | *# |
| 1372955_at | Stk38l | Serine/threonine kinase 38 like | | | **Signalling/Trafficking** | | | 443 | 710 | 1.14 | 1.34 | # |
| 1379732_at | Stx11 | Syntaxin 11 | | | **Signalling/Trafficking** | | | 903 | 651 | 1.38 | 1.35 | *# |
| 1382311_at | T2bp | TRAF2 binding protein | | | **Signalling/Trafficking** | | | 1462 | 899 | 1.70 | 1.29 | *# |
| 1382114_at | Tlk1 | Tousled-like kinase 1 | | | **Signalling/Trafficking** | | | 658 | 718 | 0.91 | 0.79 | # |
| 1379429_at | Tmed5 | Transmembrane emp24 protein transport domain containing 5 | | | **Signalling/Trafficking** | | | 575 | 923 | 1.31 | 1.14 | * |
| 1378572_at, 1380808_at, 1399052_at | Tollip | Toll interacting protein | | | **Signalling/Trafficking** | | | 354, 420, 1021 | 350, 411, 766 | 1.28 | 1.32 | *# |
| 1388649_at | Trappc10 | Trafficking protein particle complex 10 | | | **Signalling/Trafficking** | | | 193 | 196 | 1.52 | 1.47 | * |
| 1371019_at, 1391643_at | Trib1 | Tribbles homolog 1 | | | **Signalling/Trafficking** | | | 1357, 408 | 1036, 385 | 2.81 | 2.94 | *# |
| 1370694_at, 1370695_s_at 1386321_s_at | Trib3 | Tribbles homolog 3 (Drosophila) | | | **Signalling/Trafficking** | | | 660, 927, 1474 | 317, 301, 694 | 1.45 | 1.51 | *# |
| 1383981_at | Trp53bp2 | Transformation related protein 53 binding protein 2 | | | **Signalling/Trafficking** | | | 370 | 599 | 0.79 | 0.70 | *# |
| 1384163_at | Trp53inp1 | Transformation related protein 53 inducible nuclear protein 1 | | | **Signalling/Trafficking** | | | 1581 | 1935 | 1.71 | 1.46 | *# |
| 1387406_at | Uhmk1 | U2AF homology motif (UHM) kinase 1 | | | **Signalling/Trafficking** | | | 257 | 345 | 1.21 | 1.40 | # |
| 1389644_at | Wdr67 | WD repeat domain 67 | | | **Signalling/Trafficking** | | | 325 | 267 | 0.75 | 0.88 | * |
| 1370663_at, 1397409_s_at | Wee1 | Wee 1 homolog (S. pombe) | | | **Signalling/Trafficking** | | | 328, 534 | 441, 675 | 1.41 | 1.26 | *# |
| 1390996_at | Zfyve20 | Zinc finger, FYVE domain containing 20 | | | **Signalling/Trafficking** | | | 460 | 566 | 0.86 | 0.75 | # |
| 1389346_at | Adnp | Activity-dependent neuroprotective protein (3' end of 3' UTR) | | | **Transcriptional regulation** | | | 660 | 870 | 0.82 | 0.72 | # |
| 1397386_at | Aff4 | AF4/FMR2 family, member 4 | | | **Transcriptional regulation** | | | 308 | 620 | 1.43 | 1.54 | *# |
| 1367655_at | Ankrd1 | Ankyrin repeat domain 1 (cardiac muscle) | | | **Transcriptional regulation** | | | 7339 | 9018 | 0.67 | 0.63 | # |
| 1379311_at, 1380772_at, 1397437_at | Arid5a | AT rich interactive domain 5A (Mrf1 like) | | | **Transcriptional regulation** | | | 373, 186, 280 | 406, 149, 249 | 2.11 | 2.30 | *# |
| 1372964_at | Arid5b | AT rich interactive domain 5B (Mrf1 like) | | | **Transcriptional regulation** | | | 1352 | 1935 | 1.82 | 1.94 | *# |
| 1379085_at | Asxl1 | Additional sex combs like 1 (Drosophila) | | | **Transcriptional regulation** | | | 482 | 736 | 0.82 | 0.71 | # |
| 1369268_at | Atf3 | Activating transcription factor 3 | | | **Transcriptional regulation** | | | 827 | 475 | 11.99 | 13.91 | *# |
| 1395686_at | Atf7 | Activating transcription factor 7 | | | **Transcriptional regulation** | | | 410 | 340 | 1.31 | 1.17 | * |
| 1373287_at | Atoh8 | Atonal homolog 8 (Drosophila) | | | **Transcriptional regulation** | | | 1336 | 935 | 0.48 | 0.39 | *# |
| 1374433_at | Bach1 | BTB and CNC homology 1 | | | **Transcriptional regulation** | | | 1056 | 1241 | 1.45 | 1.35 | *# |
| 1374421_at | Baz1b | Bromodomain adjacent to zinc finger domain protein 1B | | | **Transcriptional regulation** | | | 834 | 1295 | 0.78 | 0.81 | * |
| 1385627_at, 1398482_at | Bcl3 | B-cell leukemia/lymphoma 3 | | | **Transcriptional regulation** | | | 715, 776 | 499, 455 | 0.51 | 0.48 | *# |
| 1379368_at | Bcl6 | B-cell leukemia/lymphoma 6 | | | **Transcriptional regulation** | | | 518 | 682 | 1.42 | 1.32 | *# |
| 1368511_at | Bhlhb3 | Basic helix-loop-helix domain containing, class B3 | | | **Transcriptional regulation** | | | 1133 | 1098 | 0.82 | 0.76 | # |
| 1373467_at | Btaf1 | BTAF1 RNA polymerase II, B-TFIID transcription factor-associated, 170kDa (Mot1 homolog, S. cerevisiae) | | | **Transcriptional regulation** | | | 251 | 520 | 1.37 | 1.43 | *# |
| 1386994_at, 1386995_at | Btg2 | B-cell translocation gene 2, anti-proliferative | | | **Transcriptional regulation** | | | 1407, 1509 | 1101, 1127 | 7.18 | 7.67 | *# |
| 1377869_at | Ccrn4l | Carbon catabolite repression 4 protein homolog | | | **Transcriptional regulation** | | | 437 | 455 | 10.17 | 11.01 | *# |
| 1374540_at | Cdca7 | Cell division cycle associated 7 | | | **Transcriptional regulation** | | | 426 | 443 | 0.73 | 0.73 | *# |
| 1368813_at, 1387343_at | Cebpd | CCAAT/enhancer binding protein (C/EBP), delta | | | **Transcriptional regulation** | | | 1081, 3816 | 981, 2512 | 0.50 | 0.46 | *# |
| 1371038_at | Cebpg | CCAAT/enhancer binding protein (C/EBP), gamma | | | **Transcriptional regulation** | | | 376 | 423 | 1.31 | 1.31 | *# |
| 1374408_at, 1386229_at | Cir | CBF1 interacting corepressor | | | **Transcriptional regulation** | | | 776, 407 | 774, 546 | 0.84 | 0.76 | # |
| 1367601_at, 1367602_at | Cited2 | Cbp/p300-interacting transactivator, with Glu/Asp-rich carboxy-terminal domain, 2 | | | **Transcriptional regulation** | | | 1861, 1899 | 1314, 2091 | 4.59 | 5.03 | *# |
| 1373190_at | Cnot4 | CCR4-NOT transcription complex, subunit 4 | | | **Transcriptional regulation** | | | 381 | 488 | 0.77 | 0.80 | * |
| 1378925_at | Crem | cAMP responsive element modulator | | | **Transcriptional regulation** | | | 429 | 318 | 1.99 | 2.01 | *# |
| 1368321_at | Egr1 | Early growth response 1 | | | **Transcriptional regulation** | | | 6553 | 3193 | 2.56 | 5.49 | *# |
| 1387306_a_at1398266_a_at | Egr2 | Early growth response 2 | | | **Transcriptional regulation** | | | 749, 102 | 464, 107 | 5.01 | 6.69 | *# |
| 1369545_at, 1392791_at | Egr3 | Early growth response 3 | | | **Transcriptional regulation** | | | 177, 314 | 179, 172 | 16.21 | 28.36 | *# |
| 1387442_at | Egr4 | Early growth response 4 | | | **Transcriptional regulation** | | | 10 | 9 | 78.10 | 68.89 | *# |
| 1393058_at | Eid2 | EP300 interacting inhibitor of differentiation 2 | | | **Transcriptional regulation** | | | 654 | 509 | 0.75 | 0.80 | * |
| 1391338_at | Eid2b | EP300 interacting inhibitor of differentiation 2B | | | **Transcriptional regulation** | | | 280 | 252 | 0.68 | 0.70 | * |
| 1389680_at | Ell2 | Elongation factor RNA polymerase II 2 | | | **Transcriptional regulation** | | | 418 | 539 | 1.25 | 1.36 | *# |
| 1399059_at | Ezh1 | Enhancer of zeste homolog 1 (Drosophila) | | | **Transcriptional regulation** | | | 551 | 561 | 0.85 | 0.77 | # |
| 1377648_at | Fam120b | Family with sequence Similarity 120B | | | **Transcriptional regulation** | | | 230 | 296 | 0.87 | 0.76 | # |
| 1386643_at, 1393843_at | Fem1b | Feminization 1 homolog b | | | **Transcriptional regulation** | | | 379, 297 | 431, 302 | 1.41 | 1.19 | * |
| 1393722_at | Fem1c | Fem-1 homolog c (C.elegans) | | | **Transcriptional regulation** | | | 243 | 361 | 1.49 | 1.32 | *# |
| 1389404_at | Fkhl18 | Forkhead-like 18 (Drosophila) | | | **Transcriptional regulation** | | | 619 | 385 | 0.66 | 0.72 | *# |
| 1375043_at | Fos | FBJ murine osteosarcoma viral oncogene homolog | | | **Transcriptional regulation** | | | 343 | 241 | 12.80 | 12.50 | *# |
| 1373759_at | FosB | FBJ osteosarcoma oncogene B | | | **Transcriptional regulation** | | | 42 | 41 | 165.65 | 113.08 | *# |
| 1368489_at | Fosl1 | Fos-like antigen 1 | | | **Transcriptional regulation** | | | 391 | 295 | 5.31 | 6.08 | *# |
| 1373035_at, 1383860_at, 1387530_a_at | Fosl2 | Fos-like antigen 2 | | | **Transcriptional regulation** | | | 1915, 227, 158 | 1742, 159, 175 | 1.95 | 2.03 | *# |
| 1377347_at | Foxj3 | Forkhead box J3 | | | **Transcriptional regulation** | | | 1649 | 1634 | 0.93 | 0.80 | # |
| 1376593_at | Foxo3 | Forkhead box O3 | | | **Transcriptional regulation** | | | 1526 | 1643 | 0.83 | 0.72 | # |
| 1388067_a_at | Gmeb2 | Glucocorticoid modulatory element binding protein 2 | | | **Transcriptional regulation** | | | 662 | 719 | 1.50 | 1.27 | * |
| 1376159_at | Gzf1 | GDNF-inducible zinc finger protein 1 | | | **Transcriptional regulation** | | | 413 | 592 | 0.77 | 0.82 | * |
| 1387036_at | Hes1 | Hairy and enhancer of split 1 (Drosophila) | | | **Transcriptional regulation** | | | 1839 | 1854 | 0.50 | 0.48 | *# |
| 1386080_at | Hey1 | Hairy/enhancer-of-split related with YRPW motif 1 | | | **Transcriptional regulation** | | | 522 | 459 | 0.73 | 0.69 | *# |
| 1384515_at | Hey2 | Hairy/enhancer-of-split related with YRPW motif 2 | | | **Transcriptional regulation** | | | 792 | 834 | 0.78 | 0.77 | *# |
| 1393629_at | Hlx1 | H2.0-like homeo box 1 (Drosophila) | | | **Transcriptional regulation** | | | 616 | 411 | 0.71 | 0.60 | * |
| 1387028_a_at | Id1 | Inhibitor of DNA binding 1 | | | **Transcriptional regulation** | | | 6337 | 3549 | 0.67 | 0.71 | *# |
| 1375120_at, 1394022_at | Id4 | Inhibitor of DNA binding 4 | | | **Transcriptional regulation** | | | 441, 803 | 178, 711 | 1.80 | 1.92 | *# |
| 1367795_at | Ifrd1 | Interferon-related developmental regulator 1 | | | **Transcriptional regulation** | | | 1465 | 1197 | 4.07 | 4.27 | *# |
| 1386550_at | Ikzf5 | IKAROS family zinc finger 5 | | | **Transcriptional regulation** | | | 341 | 423 | 1.27 | 1.19 | * |
| 1376758_at | Ing1 | Inhibitor of growth family, member 1 | | | **Transcriptional regulation** | | | 1456 | 829 | 0.77 | 0.92 | * |
| 1376812_at | Ing5 | Inhibitor of growth family, member 5 | | | **Transcriptional regulation** | | | 370 | 325 | 1.34 | 1.31 | * |
| 1368073_at | Irf1 | Interferon regulatory factor 1 | | | **Transcriptional regulation** | | | 1014 | 920 | 0.45 | 0.37 | *# |
| 1374627_at | Irf9 | Interferon regulatory factor 9 | | | **Transcriptional regulation** | | | 480 | 430 | 0.77 | 0.64 | *# |
| 1390776_at, 1394251_x_at | Irx3 | Iroquois related homeobox 3 (Drosophila) | | | **Transcriptional regulation** | | | 1510, 564 | 1685, 663 | 0.76 | 0.63 | *# |
| 1386823_at | Irx5 | Iroquois homeobox protein 5 | | | **Transcriptional regulation** | | | 367 | 356 | 0.65 | 0.54 | * |
| 1383448_at | Isgf3g | Interferon dependent positive acting transcription factor 3 gamma | | | **Transcriptional regulation** | | | 889 | 832 | 0.85 | 0.73 | # |
| 1393093_at | Jarid1c | Jumonji, AT rich interactive domain 1C (Rbp2 like) | | | **Transcriptional regulation** | | | 237 | 283 | 1.13 | 1.31 | # |
| 1369788_s_at 1374404_at, 1389528_s_at | Jun | Jun oncogene | | | **Transcriptional regulation** | | | 724, 406, 1313 | 919, 449, 1432 | 3.28 | 2.69 | *# |
| 1387788_at | Junb | Jun-B oncogene | | | **Transcriptional regulation** | | | 1913 | 1731 | 1.63 | 1.77 | *# |
| 1390000_at | Kdm6b | KDM1 lysine (K)-specific demethylase 6B | | | **Transcriptional regulation** | | | 1160 | 1138 | 1.45 | 1.48 | *# |
| 1370066_at | Keap1 | Kelch-like ECH-associated protein 1 | | | **Transcriptional regulation** | | | 906 | 713 | 0.75 | 0.76 | * |
| 1379914_at | Klf11 | Kruppel-like factor 11 | | | **Transcriptional regulation** | | | 663 | 522 | 0.73 | 0.59 | *# |
| 1368249_at, 1381396_s_at | Klf15 | Kruppel-like factor 15 | | | **Transcriptional regulation** | | | 1239, 305 | 941, 300 | 0.48 | 0.41 | *# |
| 1375248_at, 1376569_at, 1386041_a_at 1394068_x_at | Klf2 | Kruppel-like factor 2 | | | **Transcriptional regulation** | | | 497, 1009, 165, 155 | 609, 673, 141, 137 | 3.29 | 2.69 | *# |
| 1378332_at, 1389479_at | Klf3 | Kruppel-like factor 3 | | | **Transcriptional regulation** | | | 1072, 1974 | 706, 1924 | 0.70 | 0.66 | *# |
| 1387260_at | Klf4 | Kruppel-like factor | | | **Transcriptional regulation** | | | 1823 | 1242 | 4.17 | 4.14 | *# |
| 1368363_at, 1394039_at | Klf5 | Kruppel-like factor 5 | | | **Transcriptional regulation** | | | 279, 237 | 325, 391 | 2.95 | 3.24 | *# |
| 1387060_at, 1388986_at, 1395557_at | Klf6 | Kruppel-like factor 6 | | | **Transcriptional regulation** | | | 2062, 1774, 697 | 959, 1895, 755 | 4.03 | 4.30 | *# |
| 1370209_at, 1371864_at | Klf9 | Kruppel-like factor 9 | | | **Transcriptional regulation** | | | 2500, 2370 | 1630, 2292 | 1.32 | 1.20 | * |
| 1376632_at | Lmcd1 | LIM and cysteine-rich domains 1 | | | **Transcriptional regulation** | | | 1239 | 1212 | 2.45 | 3.39 | *# |
| 1372535_at | LOC366431 | Similar to RIKEN cDNA 2210012G02 | | | **Transcriptional regulation** | | | 463 | 315 | 0.82 | 0.73 | # |
| 1382108_at | Mafb | v-maf musculoaponeurotic fibrosarcoma oncogene family, protein B (avian) | | | **Transcriptional regulation** | | | 435 | 456 | 0.82 | 0.80 | # |
| 1380229_at | Maff | v-maf musculoaponeurotic fibrosarcoma oncogene family, protein F (avian) | | | **Transcriptional regulation** | | | 644 | 235 | 1.44 | 2.36 | *# |
| 1372211_at | Mafk | v-maf musculoaponeurotic fibrosarcoma oncogene family, protein K (avian) | | | **Transcriptional regulation** | | | 1414 | 967 | 1.71 | 1.87 | *# |
| 1376196_a_at | Med4 | Mediator complex subunit 4 | | | **Transcriptional regulation** | | | 1238 | 1034 | 0.80 | 0.84 | * |
| 1373189_at | Mkl1 | Megakaryoblastic leukemia (translocation) 1 | | | **Transcriptional regulation** | | | 324 | 415 | 0.84 | 0.76 | # |
| 1374687_at | Mkl2 | MKL/myocardin-like 2 | | | **Transcriptional regulation** | | | 691 | 1245 | 0.81 | 0.78 | # |
| 1375929_at | Mnt | Max binding protein | | | **Transcriptional regulation** | | | 903 | 765 | 0.80 | 0.71 | *# |
| 1387732_at | Mterf | Mitochondrial transcription termination factor 1 | | | **Transcriptional regulation** | | | 784 | 785 | 0.64 | 0.67 | *# |
| 1376077_at, 1389789_at | Mxd1 | Max dimerization protein | | | **Transcriptional regulation** | | | 391, 661 | 332, 497 | 1.23 | 1.28 | *# |
| 1368308_at | Myc | Myelocytomatosis viral oncogene homolog (avian) | | | **Transcriptional regulation** | | | 702 | 634 | 3.68 | 4.75 | *# |
| 1376648_at | Mycn | v-myc myelocytomatosis viral related oncogene, neuroblastoma derived (avian) | | | **Transcriptional regulation** | | | 514 | 596 | 0.83 | 0.72 | # |
| 1368488_at | Nfil3 | Nuclear factor, interleukin 3 regulated | | | **Transcriptional regulation** | | | 645 | 559 | 3.81 | 3.27 | *# |
| 1389538_at | Nfkbia | Nuclear factor of kappa light chain gene enhancer in B-cells inhibitor, alpha | | | **Transcriptional regulation** | | | 2013 | 954 | 0.75 | 0.64 | * |
| 1378032_at | Nfkbiz | Nuclear factor of kappa light polypeptide gene enhancer in B-cells inhibitor, zeta | | | **Transcriptional regulation** | | | 741 | 893 | 3.90 | 3.88 | *# |
| 1368376_at | Nr0b2 | Nuclear receptor subfamily 0, group B, member 2 | | | **Transcriptional regulation** | | | 344 | 311 | 0.67 | 0.53 | *# |
| 1385638_at, 1389554_at | Nr2f2 | Nuclear receptor subfamily 2, group F, member 2 | | | **Transcriptional regulation** | | | 299, 1354 | 275, 927 | 0.85 | 0.76 | # |
| 1386935_at | Nr4a1 | Nuclear receptor subfamily 4, group A, member 1 | | | **Transcriptional regulation** | | | 574 | 635 | 19.74 | 18.48 | *# |
| 1369007_at | Nr4a2 | Nuclear receptor subfamily 4, group A, member 2 | | | **Transcriptional regulation** | | | 96 | 95 | 5.23 | 5.96 | *# |
| 1369067_at, 1393389_at | Nr4a3 | Nuclear receptor subfamily 4, group A, member 3 | | | **Transcriptional regulation** | | | 76, 86 | 85, 136 | 23.75 | 30.72 | *# |
| 1385667_x_at | Pbx1 | Pre-B-cell leukemia homeobox 1 | | | **Transcriptional regulation** | | | 382 | 388 | 0.80 | 0.77 | # |
| 1374141_at | Pcif1 | PDX1 C-terminal inhibiting factor 1 | | | **Transcriptional regulation** | | | 460 | 441 | 0.87 | 0.79 | # |
| 1374032_at, 1394014_at | Phf12 | PHD finger protein 12 | | | **Transcriptional regulation** | | | 571, 268 | 603, 360 | 0.84 | 0.71 | # |
| 1376931_at, 1379408_at | Phf20 | PHD finger protein 20 | | | **Transcriptional regulation** | | | 477, 315 | 384, 345 | 0.78 | 0.92 | * |
| 1387122_at | Plagl1 | Pleiomorphic adenoma gene-like 1 | | | **Transcriptional regulation** | | | 934 | 1519 | 1.26 | 1.40 | # |
| 1375504_at | Polg2 | Polymerase (DNA directed), gamma 2, accessory subunit | | | **Transcriptional regulation** | | | 388 | 372 | 0.87 | 0.76 | # |
| 1371822_at | Polr3d | Polymerase (RNA) III (DNA directed) polypeptide D | | | **Transcriptional regulation** | | | 1574 | 1147 | 1.14 | 1.33 | # |
| 1393743_at | Ppargc1a | Peroxisome proliferator-activated receptor gamma, coactivator 1 alpha | | | **Transcriptional regulation** | | | 495 | 717 | 0.76 | 0.70 | *# |
| 1390531_at | Pric285 | Peroxisomal proliferator-activated receptor A interacting complex 285 | | | **Transcriptional regulation** | | | 219 | 356 | 1.27 | 1.48 | *# |
| 1376672_at | Prox1 | Prospero-related homeobox 1 | | | **Transcriptional regulation** | | | 545 | 761 | 0.85 | 0.74 | # |
| 1377747_at, 1391899_at | Purb | Purine-rich element binding protein B | | | **Transcriptional regulation** | | | 3528, 255 | 2526, 175 | 1.36 | 1.44 | *# |
| 1368176_at, 1373579_at | Rara | Retinoic acid receptor, alpha | | | **Transcriptional regulation** | | | 985, 1000 | 639, 635 | 0.83 | 0.78 | # |
| 1389748_at | Runx1t1 | Runt-related transcription factor 1; translocated to, 1 | | | **Transcriptional regulation** | | | 679 | 595 | 0.86 | 0.77 | # |
| 1383034_at | Rybp | RING1 and YY1 binding protein | | | **Transcriptional regulation** | | | 395 | 317 | 1.26 | 1.38 | *# |
| 1389437_at | Sall2 | Sal-like 2 (Drosophila) | | | **Transcriptional regulation** | | | 395 | 423 | 0.73 | 0.50 | *# |
| 1393338_at | Scx | Scleraxis | | | **Transcriptional regulation** | | | 883 | 867 | 0.78 | 0.89 | * |
| 1372417_at | Sertad1 | SERTA domain containing 1 | | | **Transcriptional regulation** | | | 1804 | 870 | 1.96 | 1.97 | *# |
| 1375279_at | Sertad2 | SERTA domain containing 2 | | | **Transcriptional regulation** | | | 2695 | 1578 | 1.11 | 1.27 | # |
| 1379621_at | Sertad3 | SERTA domain containing 3 | | | **Transcriptional regulation** | | | 461 | 352 | 0.75 | 0.81 | * |
| 1372376_at | Setd2 | SET domain containing 2 | | | **Transcriptional regulation** | | | 396 | 713 | 0.85 | 0.77 | # |
| 1372347_at | Skil | SKI-like | | | **Transcriptional regulation** | | | 1991 | 2699 | 1.48 | 1.55 | *# |
| 1388524_at | Smad5 | MAD homolog 5 (Drosophila) | | | **Transcriptional regulation** | | | 797 | 850 | 0.90 | 0.76 | # |
| 1394025_at | Smad6 | SMAD family member 6 | | | **Transcriptional regulation** | | | 1410 | 1033 | 0.60 | 0.53 | *# |
| 1368896_at | Smad7 | MAD homolog 7 (Drosophila) | | | **Transcriptional regulation** | | | 1127 | 845 | 0.77 | 0.59 | *# |
| 1393262_at | Smad9 | MAD homolog 9 (Drosophila) | | | **Transcriptional regulation** | | | 448 | 555 | 0.79 | 0.64 | *# |
| 1371767_at | Smarcd1 | SWI/SNF related, matrix associated, actin dependent regulator of chromatin, subfamily d, member 1 | | | **Transcriptional regulation** | | | 288 | 241 | 1.12 | 1.26 | # |
| 1373219_at | Snai1 | Snail homolog 1 (Drosophila) | | | **Transcriptional regulation** | | | 1861 | 1250 | 0.23 | 0.28 | *# |
| 1383589_at | Snai2 | Snail homolog 2 (Drosophila) | | | **Transcriptional regulation** | | | 762 | 500 | 0.67 | 0.60 | * |
| 1367790_at | Snd1 | Staphylococcal nuclease domain containing 1 | | | **Transcriptional regulation** | | | 1299 | 2517 | 0.78 | 0.89 | * |
| 1383210_at | Sox11 | SRY-box containing gene 11 | | | **Transcriptional regulation** | | | 340 | 264 | 1.33 | 1.51 | *# |
| 1373860_at, 1375123_at | Sox4 | SRY-box containing gene 4 | | | **Transcriptional regulation** | | | 3115, 639 | 2372, 1005 | 0.87 | 0.76 | # |
| 1374829_at, 1388842_at, 1395378_at | Srf | Serum response factor | | | **Transcriptional regulation** | | | 579, 4065, 135 | 393, 2603, 126 | 1.91 | 2.31 | *# |
| 1382350_at | Stat6 | Signal transducer and activator of transcription 6 | | | **Transcriptional regulation** | | | 350 | 282 | 0.78 | 0.92 | * |
| 1373238_at, 1397852_at | Tada1l | Transcriptional adaptor 1 (HFI1 homolog, yeast) like | | | **Transcriptional regulation** | | | 536, 452 | 478, 433 | 0.77 | 0.72 | # |
| 1388782_at | Tcf21 | Transcription factor 21 | | | **Transcriptional regulation** | | | 2761 | 1776 | 1.31 | 1.21 | * |
| 1386435_at | Tcfap4 | Transcription factor AP4 | | | **Transcriptional regulation** | | | 482 | 468 | 0.88 | 0.78 | # |
| 1390681_at | Tgif2 | TGFB-induced factor homeobox 2 | | | **Transcriptional regulation** | | | 644 | 449 | 0.78 | 0.75 | *# |
| 1385641_at | Tnfaip3 | Tumor necrosis factor, alpha-induced protein 3 | | | **Transcriptional regulation** | | | 801 | 1101 | 3.20 | 2.35 | *# |
| 1382240_at | Trim33 | Tripartite motif-containing 33 | | | **Transcriptional regulation** | | | 316 | 571 | 0.81 | 0.77 | # |
| 1391173_at | Trps1 | Trichorhinophalangeal syndrome I (human) | | | **Transcriptional regulation** | | | 239 | 364 | 0.91 | 0.76 | # |
| 1378165_at, 1387750_at | Twist1 | Twist gene homolog 1 (Drosophila) | | | **Transcriptional regulation** | | | 805, 116 | 456, 99 | 3.90 | 4.54 | *# |
| 1371131_a_at | Txnip | Thioredoxin interacting protein | | | **Transcriptional regulation** | | | 4822 | 5221 | 0.75 | 0.56 | *# |
| 1373941_at | Yeats2 | YEATS domain containing 2 | | | **Transcriptional regulation** | | | 355 | 499 | 0.83 | 0.74 | # |
| 1395901_at | Yy1 | YY1 transcription factor | | | **Transcriptional regulation** | | | 756 | 856 | 0.84 | 0.80 | # |
| 1373771_at | Zbtb34 | Zinc finger and BTB domain containing 34 | | | **Transcriptional regulation** | | | 374 | 396 | 0.80 | 0.77 | *# |
| 1379439_at | Zbtb44 | Zinc finger and BTB domain containing 44 | | | **Transcriptional regulation** | | | 325 | 226 | 1.41 | 1.21 | * |
| 1385929_at | Zfp11 | Zinc finger protein 11 | | | **Transcriptional regulation** | | | 316 | 301 | 0.72 | 0.67 | * |
| 1393150_at | Zfp161 | Zinc finger protein 161 | | | **Transcriptional regulation** | | | 986 | 730 | 0.75 | 0.80 | * |
| 1376628_at | Zfp189 | Zinc finger protein 189 | | | **Transcriptional regulation** | | | 309 | 369 | 1.27 | 1.17 | * |
| 1386424_at | Zfp212 | Zinc finger protein 212 | | | **Transcriptional regulation** | | | 1014 | 924 | 0.83 | 0.74 | # |
| 1372205_at | Zfp278 | Zinc finger protein 278 | | | **Transcriptional regulation** | | | 499 | 483 | 0.76 | 0.59 | *# |
| 1382642_at, 1385869_at | Zfp281 | Zinc finger protein 281 | | | **Transcriptional regulation** | | | 708, 600 | 900, 977 | 2.22 | 2.44 | *# |
| 1389674_at | Zfp282 | Zinc finger protein 282 | | | **Transcriptional regulation** | | | 330 | 352 | 0.66 | 0.77 | *# |
| 1385379_at | Zfp287 | Zinc finger protein 287 | | | **Transcriptional regulation** | | | 222 | 279 | 0.72 | 0.77 | # |
| 1393127_at | Zfp358 | zinc finger protein 358 | | | **Transcriptional regulation** | | | 985 | 814 | 0.72 | 0.85 | * |
| 1378049_at | Zfp362 | Zinc finger protein 362 | | | **Transcriptional regulation** | | | 1126 | 822 | 0.87 | 0.75 | # |
| 1381065_at | Zfp383 | Zinc finger protein 383 | | | **Transcriptional regulation** | | | 539 | 808 | 0.74 | 0.60 | *# |
| 1381607_at | Zfp407 | Zinc finger protein 407 | | | **Transcriptional regulation** | | | 175 | 314 | 0.83 | 0.72 | # |
| 1370984_at | Zfp46 | Zinc finger protein 46 | | | **Transcriptional regulation** | | | 754 | 735 | 0.54 | 0.53 | *# |
| 1391507_at | Zfp467 | Zinc finger protein 467 | | | **Transcriptional regulation** | | | 367 | 378 | 0.48 | 0.51 | *# |
| 1386721_at, 1393990_at | Zfp503 | Zinc finger protein 503 | | | **Transcriptional regulation** | | | 1054, 923 | 953, 952 | 0.74 | 0.79 | *# |
| 1373583_at | Zfp518b | Zinc finger protein 518B | | | **Transcriptional regulation** | | | 370 | 560 | 1.20 | 1.29 | # |
| 1389366_at | Zfp553 | Zinc finger protein 553 | | | **Transcriptional regulation** | | | 490 | 465 | 0.51 | 0.54 | *# |
| 1375859_a_at | Zfp568 | Zinc finger protein 568 | | | **Transcriptional regulation** | | | 779 | 1248 | 1.33 | 1.28 | *# |
| 1385059_at, 1389685_at, 1398536_at | Zfp655 | Zinc finger protein 655 | | | **Transcriptional regulation** | | |  |  | 1.45 | 1.55 | # |
| 1385236_at | Zfp687 | Zinc finger protein 687 | | | **Transcriptional regulation** | | | 432 | 401 | 0.80 | 0.79 | # |
| 1389445_at | Zfp688 | Zinc finger protein 688 | | | **Transcriptional regulation** | | | 640 | 324 | 0.72 | 0.74 | *# |
| 1374944_at | Zfp691 | Zinc finger protein 691 | | | **Transcriptional regulation** | | | 340 | 271 | 0.49 | 0.58 | * |
| 1373680_at | Zfp697 | Zinc finger protein 697 | | | **Transcriptional regulation** | | | 389 | 426 | 2.00 | 2.26 | *# |
| 1374258_at | Zfp764 | Zinc finger protein 764 | | | **Transcriptional regulation** | | | 255 | 186 | 0.68 | 0.80 | * |
| 1372699_at | Zfp775 | Zinc finger protein 775 | | | **Transcriptional regulation** | | | 252 | 189 | 0.53 | 0.45 | * |
| 1377600_at | Zfp777 | Zinc finger protein 777 | | | **Transcriptional regulation** | | | 470 | 311 | 0.76 | 0.83 | * |
| 1384432_at | Zfp96 | Zinc finger protein 96 | | | **Transcriptional regulation** | | | 242 | 222 | 0.55 | 0.46 | * |
| 1385773_at | Zkscan1 | Zinc finger with KRAB and SCAN domains 1 | | | **Transcriptional regulation** | | | 209 | 304 | 0.78 | 0.78 | # |
| 1374127_at | Zkscan6 | Zinc finger with KRAB and SCAN domains 6 | | | **Transcriptional regulation** | | | 275 | 261 | 0.72 | 0.77 | * |
| 1376917_at, 1390945_at | Znf292 | Zinc finger protein 292 | | | **Transcriptional regulation** | | | 183, 200 | 364, 398 | 0.75 | 0.65 | # |
| 1390448_at | Abhd13 | Abhydrolase domain containing 13 | | | **Unknown function** | | | 731 | 623 | 1.28 | 1.30 | * |
| 1385701_at | Ahnak2 | AHNAK nucleoprotein 2 | | | **Unknown function** | | | 121 | 551 | 0.79 | 0.73 | # |
| 1397525_at | Alkbh5 | AlkB, alkylation repair homolog 5 (E. coli) | | | **Unknown function** | | | 692 | 461 | 0.95 | 0.79 | # |
| 1372069_at | Ankrd15 | Ankyrin repeat domain 15 | | | **Unknown function** | | | 1553 | 2390 | 1.40 | 1.53 | *# |
| 1386065_at | Ankrd57 | Ankyrin repeat domain 57 | | | **Unknown function** | | | 312 | 269 | 2.26 | 2.09 | *# |
| 1367614_at | Anxa1 | Annexin A1 | | | **Unknown function** | | | 4772 | 4962 | 1.78 | 1.67 | *# |
| 1387068_at | Arc | Activity regulated cytoskeletal-associated protein | | | **Unknown function** | | | 224 | 256 | 5.59 | 5.14 | *# |
| 1383246_at | Armc7 | Armadillo repeat containing 7 | | | **Unknown function** | | | 444 | 373 | 0.73 | 0.79 | *# |
| 1389230_at | Arrdc3 | Arrestin domain containing 3 | | | **Unknown function** | | | 2272 | 2632 | 0.86 | 0.79 | # |
| 1372956_at | Bat4 | HLA-B associated transcript 4 | | | **Unknown function** | | | 483 | 436 | 0.75 | 0.70 | * |
| 1381838_at | Bivm | Basic, immunoglobulin-like variable motif-containing protein | | | **Unknown function** | | | 374 | 316 | 0.80 | 0.76 | # |
| 1374853_at | Bod1 | Biorientation of chromosomes in cell division 1 | | | **Unknown function** | | | 2061 | 1068 | 0.82 | 0.74 | # |
| 1389029_at | Brd3 | Bromodomain containing 3 | | | **Unknown function** | | | 1632 | 1360 | 0.78 | 0.76 | *# |
| 1389568_at | Calhm2 | Calcium homeostasis modulator 2 | | | **Unknown function** | | | 779 | 628 | 0.79 | 0.67 | *# |
| 1383545_at | Ccdc127 | Coiled-coil domain containing 127 | | | **Unknown function** | | | 711 | 393 | 0.76 | 0.85 | * |
| 1393951_at | Ccdc9 | Coiled-coil domain containing 9 | | | **Unknown function** | | | 435 | 430 | 1.26 | 1.06 | * |
| 1374139_at | Cdr2 | Cerebellar degeneration-related 2 | | | **Unknown function** | | | 1145 | 609 | 1.62 | 2.11 | *# |
| 1383738_at | Cep68 | Centrosomal protein 68kDa | | | **Unknown function** | | | 698 | 457 | 0.80 | 0.79 | * |
| 1374416_at | Chchd8 | Coiled-coil-helix-coiled-coil-helix domain containing 8 | | | **Unknown function** | | | 904 | 453 | 0.72 | 0.63 | *# |
| 1373525_at, 1379666_at | Dcun1d3 | DCN1, defective in cullin neddylation 1, domain containing 3 | | | **Unknown function** | | | 436, 334 | 327, 296 | 1.98 | 2.09 | *# |
| 1379425_at | Dem1 | Defects in morphology 1 homolog (S. cerevisiae) | | | **Unknown function** | | | 329 | 191 | 0.67 | 0.77 | * |
| 1374718_at | Dtx3l | Deltex 3-like (Drosophila) | | | **Unknown function** | | | 306 | 436 | 0.85 | 0.74 | # |
| 1377938_at | Fam100a | Family with sequence Similarity 100, member A | | | **Unknown function** | | | 1614 | 963 | 1.72 | 1.67 | *# |
| 1389085_at | Fam101b | Family with sequence Similarity 101, member B | | | **Unknown function** | | | 667 | 524 | 0.75 | 0.87 | * |
| 1379356_at, 1380696_at | Fam102b | Family with sequence Similarity 102, member B | | | **Unknown function** | | | 1161, 1306 | 866, 520 | 1.43 | 1.43 | *# |
| 1383295_at | Fam110a | Family with sequence Similarity 110, member A | | | **Unknown function** | | | 214 | 162 | 1.94 | 1.73 | * |
| 1385251_at | Fam110c | Family with sequence Similarity 110, member C | | | **Unknown function** | | | 124 | 76 | 4.54 | 3.82 | *# |
| 1383155_at | Fam117b | Family with sequence Similarity 117, member B | | | **Unknown function** | | | 686 | 647 | 0.90 | 0.79 | # |
| 1372621_at | Fam176b | Family with sequence Similarity 176, member B | | | **Unknown function** | | | 3659 | 1474 | 0.80 | 0.72 | # |
| 1391003_at | Fam178a | Family with sequence Similarity 178, member A | | | **Unknown function** | | | 841 | 823 | 0.79 | 0.90 | * |
| 1394695_at, 1394745_at, 1394940_at | Fam46a | Family with sequence Similarity 46, member A | | | **Unknown function** | | | 262, 157, 1813 | 335, 263, 1549 | 1.63 | 1.52 | *# |
| 1373341_at | Fam53a | Family with sequence Similarity 53, member A | | | **Unknown function** | | | 549 | 408 | 0.84 | 0.74 | # |
| 1391741_a_at | Fam78a | Family with sequence Similarity 78, member A | | | **Unknown function** | | | 491 | 344 | 0.67 | 0.58 | *# |
| 1382267_at | Fjx1 | Four jointed box 1 (Drosophila) | | | **Unknown function** | | | 249 | 187 | 1.42 | 1.48 | *# |
| 1372526_at | Flcn | Folliculin | | | **Unknown function** | | | 2922 | 2903 | 0.79 | 0.72 | *# |
| 1392618_at | Frmd4a | FERM domain containing 4A | | | **Unknown function** | | | 1121 | 1406 | 0.84 | 0.78 | # |
| 1386420_at | Harbi1 | Harbinger transposase derived 1 | | | **Unknown function** | | | 496 | 545 | 0.73 | 0.83 | * |
| 1391429_at | Hfe2 | Hemochromatosis type 2 (juvenile) homolog | | | **Unknown function** | | | 321 | 363 | 0.87 | 0.78 | # |
| 1376076_at | Hig2 | Hypoxia-inducible protein 2 | | | **Unknown function** | | | 1556 | 934 | 0.57 | 0.70 | *# |
| 1381919_at | Hps6 | Hermansky-Pudlak syndrome 6 | | | **Unknown function** | | | 389 | 220 | 0.55 | 0.49 | * |
| 1372389_at | Ier2 | Immediate early response 2 | | | **Unknown function** | | | 1177 | 637 | 3.02 | 3.92 | *# |
| 1389355_at, 1389882_at | Ier5 | Immediate early response 5 | | | **Unknown function** | | | 1987, 77 | 2068, 99 | 4.53 | 5.07 | *# |
| 1389675_at, 1391026_at | Ier5l | Immediate early response 5-like | | | **Unknown function** | | | 944, 1820 | 637, 1234 | 0.32 | 0.34 | *# |
| 1390104_at | Irgq | Immunity-related GTPase family, Q | | | **Unknown function** | | | 734 | 723 | 0.76 | 0.73 | # |
| 1391356_at | Iws1 | IWS1 homolog (S. cerevisiae) | | | **Unknown function** | | | 796 | 996 | 1.39 | 1.21 | * |
| 1393793_at | Kbtbd11 | Kelch repeat and BTB (POZ) domain containing 11 | | | **Unknown function** | | | 607 | 578 | 0.72 | 0.63 | *# |
| 1380449_at | Klhdc5 | Kelch domain containing 5 | | | **Unknown function** | | | 413 | 331 | 0.77 | 0.63 | *# |
| 1383906_at | Lincr | Lung inducible neuralized-related C3HC4 RING finger protein | | | **Unknown function** | | | 557 | 330 | 1.31 | 1.28 | * |
| 1395472_at | Lrrc17 | Leucine rich repeat containing 17 | | | **Unknown function** | | | 1619 | 1489 | 0.86 | 0.77 | # |
| 1372405_at | Lrrc68 | Leucine rich repeat containing 68 | | | **Unknown function** | | | 468 | 277 | 1.10 | 1.31 | # |
| 1376436_at, 1384584_at | Lysmd3 | LysM, putative peptidoglycan-binding, domain containing 3 | | | **Unknown function** | | | 900, 509 | 598, 572 | 1.60 | 1.33 | *# |
| 1372060_at | Lysmd4 | LysM, putative peptidoglycan-binding, domain containing 4 | | | **Unknown function** | | | 704 | 467 | 0.54 | 0.52 | *# |
| 1376957_at | Lzic | Leucine zipper and CTNNBIP1 domain containing | | | **Unknown function** | | | 308 | 220 | 0.80 | 0.87 | * |
| 1377696_at | Mesdc1 | Mesoderm development candidate 1 | | | **Unknown function** | | | 1474 | 955 | 1.19 | 1.39 | # |
| 1389911_at | Metrnl | Meteorin, glial cell differentiation regulator-like | | | **Unknown function** | | | 2664 | 1329 | 1.41 | 1.50 | *# |
| 1390063_at | Mfap3 | Microfibrillar-associated protein 3 | | | **Unknown function** | | | 480 | 522 | 0.87 | 0.78 | # |
| 1389541_at | Mfhas1 | Malignant fibrous histiocytoma amplified sequence 1 | | | **Unknown function** | | | 1701 | 1442 | 0.87 | 0.80 | # |
| 1379059_at | Micall2 | MICAL-like 2 | | | **Unknown function** | | | 372 | 367 | 0.77 | 0.64 | *# |
| 1373496_at | Mkrn1 | Makorin ring finger protein 1 | | | **Unknown function** | | | 503 | 380 | 0.80 | 0.68 | # |
| 1371692_at | Mllt11 | Myeloid/lymphoid or mixed-lineage leukemia (trithorax homolog, Drosophila); translocated to, 11 | | | **Unknown function** | | | 2399 | 1400 | 1.28 | 1.86 | *# |
| 1388453_at | Myadm | Myeloid-associated differentiation marker | | | **Unknown function** | | | 4234 | 4303 | 1.31 | 1.42 | *# |
| 1370174_at | Myd116 | Myeloid differentiation primary response gene 116 | | | **Unknown function** | | | 1968 | 1132 | 2.11 | 2.41 | *# |
| 1377955_at | Orai2 | ORAI calcium release-activated calcium modulator 2 | | | **Unknown function** | | | 492 | 340 | 0.71 | 0.63 | *# |
| 1380553_at | Osgin2 | Oxidative stress induced growth inhibitor family member 2 | | | **Unknown function** | | | 229 | 254 | 1.30 | 1.25 | * |
| 1384254_at | Otud1 | OTU domain containing 1 | | | **Unknown function** | | | 1223 | 1602 | 5.50 | 6.08 | *# |
| 1390814_at, 1399081_at | Peli1 | Pellino homolog 1 (Drosophila) | | | **Unknown function** | | | 980, 542 | 741, 522 | 2.21 | 1.69 | *# |
| 1368303_at | Per2 | Period homolog 2 (Drosophila) | | | **Unknown function** | | | 230 | 361 | 1.84 | 1.80 | # |
| 1390514_at | Pfaap5 | Phosphonoformate immuno-associated protein 5 | | | **Unknown function** | | | 1118 | 1250 | 1.25 | 1.11 | * |
| 1392534_at | Pmepa1 | Prostate transmembrane protein, androgen induced 1 (protein coding region) | | | **Unknown function** | | | 3258 | 1852 | 1.17 | 1.35 | # |
| 1394736_at | Popdc2 | Popeye domain containing 2 | | | **Unknown function** | | | 944 | 954 | 1.23 | 1.28 | # |
| 1398508_at | Pqcp | polyglutamine-containing protein | | | **Unknown function** | | | 2310 | 2082 | 0.73 | 0.64 | *# |
| 1378978_a_at1392593_a_at | Prr12 | Proline rich 12 | | | **Unknown function** | | | 324, 232 | 335, 286 | 0.73 | 0.73 | *# |
| 1376348_at | Ptcd2 | Pentatricopeptide repeat domain 2 | | | **Unknown function** | | | 379 | 412 | 0.79 | 0.83 | * |
| 1389800_at | Pwwp2b | PWWP domain containing 2b | | | **Unknown function** | | | 342 | 252 | 0.75 | 0.69 | *# |
| 1389254_at | Qser1 | Glutamine and serine rich 1 | | | **Unknown function** | | | 839 | 881 | 0.84 | 0.78 | # |
| 1374545_at | Rkhd2 | Ring finger and KH domain containing 2 | | | **Unknown function** | | | 2813 | 3039 | 1.30 | 1.15 | * |
| 1374906_at | Rnf113a1 | Ring finger protein 113A1 | | | **Unknown function** | | | 713 | 491 | 0.78 | 0.81 | * |
| 1389790_at | Rnf169 | Ring finger protein 169 | | | **Unknown function** | | | 450 | 465 | 0.73 | 0.66 | *# |
| 1373284_at | Sav1 | Salvador homolog 1 (Drosophila) | | | **Unknown function** | | | 1534 | 1091 | 0.87 | 0.78 | # |
| 1393959_at | Sbno1 | Sno, strawberry notch homolog 1 (Drosophila) | | | **Unknown function** | | | 515 | 609 | 0.75 | 0.85 | * |
| 1385291_a_at | Sc65 | Synaptonemal complex protein SC65 | | | **Unknown function** | | | 643 | 445 | 0.86 | 0.79 | # |
| 1372248_at | Sesn1 | Sestrin 1 | | | **Unknown function** | | | 577 | 564 | 0.56 | 0.54 | *# |
| 1384899_at | Spin4 | Spindlin family, member 4 | | | **Unknown function** | | | 199 | 321 | 1.69 | 1.47 | *# |
| 1382349_at | Tbccd1 | TBCC domain containing 1 | | | **Unknown function** | | | 320 | 190 | 0.74 | 0.88 | * |
| 1390832_at | Tmcc3 | Transmembrane and coiled-coil domain family 3 | | | **Unknown function** | | | 1355 | 984 | 0.65 | 0.60 | *# |
| 1382010_at | Tmem107 | Transmembrane protein 107 | | | **Unknown function** | | | 583 | 417 | 0.79 | 0.92 | * |
| 1372935_at | Tmem119 | Transmembrane protein 119 homolog | | | **Unknown function** | | | 673 | 471 | 0.78 | 0.66 | *# |
| 1379683_at | Tmem136 | Transmembrane protein 136 | | | **Unknown function** | | | 568 | 553 | 0.84 | 0.72 | # |
| 1390042_at | Tmem140 | Transmembrane protein 140 | | | **Unknown function** | | | 849 | 543 | 0.77 | 0.74 | *# |
| 1374484_at | Tmem39a | Transmembrane protein 39a | | | **Unknown function** | | | 1038 | 928 | 1.44 | 1.41 | *# |
| 1376571_at | Tmem42 | Transmembrane protein 42 | | | **Unknown function** | | | 758 | 442 | 0.81 | 0.70 | # |
| 1388773_at | Tnfaip2 | Tumor necrosis factor, alpha-induced protein 2 | | | **Unknown function** | | | 1320 | 1182 | 0.84 | 0.75 | # |
| 1389865_at | Toe1 | Target of EGR1, member 1 (nuclear) | | | **Unknown function** | | | 219 | 329 | 0.86 | 0.80 | # |
| 1374731_at | Trim25 | Tripartite motif protein 25 | | | **Unknown function** | | | 2323 | 2622 | 0.80 | 0.74 | *# |
| 1374228_at, 1397527_at | Trim47 | Tripartite motif protein 47 | | | **Unknown function** | | | 3316, 464 | 2522, 561 | 0.81 | 0.72 | # |
| 1391027_at | Trim65 | Tripartite motif-containing 65 | | | **Unknown function** | | | 366 | 297 | 0.67 | 0.58 | *# |
| 1376340_a_at | Tssc4 | Tumor suppressing subtransferable candidate 4 | | | **Unknown function** | | | 1048 | 811 | 0.86 | 0.79 | # |
| 1397349_at | Ubxd6 | UBX domain containing 6 | | | **Unknown function** | | | 243 | 266 | 1.31 | 1.12 | * |
| 1385755_at | Vezf1 | Vascular endothelial zinc finger 1 | | | **Unknown function** | | | 823 | 774 | 0.80 | 0.80 | * |
| 1382679_at | Wdr43 | WD-repeat protein 43 | | | **Unknown function** | | | 291 | 341 | 1.26 | 1.19 | * |
| 1395974_at | Wdr81 | WD repeat domain 81 | | | **Unknown function** | | | 367 | 394 | 0.77 | 0.81 | * |
| 1371729_at | Ypel5 | Yippee-like 5 (Drosophila) | | | **Unknown function** | | | 1759 | 1362 | 0.85 | 0.76 | # |
| 1385699_at | Ythdf1 | YTH domain family 1 | | | **Unknown function** | | | 621 | 521 | 1.27 | 1.37 | *# |
| 1372414_at, 1382199_at | Zcchc14 | Zinc finger, CCHC domain containing 14 | | | **Unknown function** | | | 1030, 614 | 1148, 773 | 0.85 | 0.75 | # |
| 1388672_at | Zcchc24 | zinc finger, CCHC domain containing 24 | | | **Unknown function** | | | 2471 | 1538 | 0.82 | 0.80 | # |
| 1371816_at | Zcchc3 | Zinc finger, CCHC domain containing 3 | | | **Unknown function** | | | 751 | 793 | 0.86 | 0.78 | # |
| 1373767_at | Zfand2a | Zinc finger, AN1-type domain 2A | | | **Unknown function** | | | 766 | 636 | 2.02 | 1.52 | *# |
| 1375130_at, 1388868_at | Zfand5 | Zinc finger, AN1-type domain 5 | | | **Unknown function** | | | 357, 4667 | 827, 4425 | 2.02 | 1.63 | *# |
| 1376465_at | Zfp704 | Zinc finger protein 704 | | | **Unknown function** | | | 712 | 699 | 0.85 | 0.74 | # |
| 1372030_at | Zfyve21 | Zinc finger, FYVE domain containing 21 | | | **Unknown function** | | | 2217 | 1167 | 0.83 | 0.77 | # |
